# Antimitotic Naphthalene Sulfonamides Are Potent Antitumor Agents Acting Differently from Colchicine

**DOI:** 10.3390/pharmaceutics18060733

**Published:** 2026-06-13

**Authors:** Miguel Marín, Raúl Fuentes-Martín, Baldomero Sánchez, Laura Gallego-Yerga, Rafael Peláez

**Affiliations:** 1 Laboratorio de Química Orgánica y Farmacéutica, Departamento de Ciencias Farmacéuticas, Universidad de Salamanca, Campus Miguel de Unamuno, E-37007 Salamanca, Spain; mmarin@usal.es (M.M.); raulfuentes@usal.es (R.F.-M.); u130953@usal.es (B.S.); gallego@usal.es (L.G.-Y.); 2 Instituto de Investigación Biomédica de Salamanca (IBSAL), Facultad de Farmacia, Universidad de Salamanca, Campus Miguel de Unamuno, E-37007 Salamanca, Spain; 3 Centro de Investigación de Enfermedades Tropicales de la Universidad de Salamanca (CIETUS), Facultad de Farmacia, Universidad de Salamanca, Campus Miguel de Unamuno, E-37007 Salamanca, Spain

**Keywords:** tubulin, colchicine-binding site, sulfonamides, cancer, microtubule-targeting agents

## Abstract

**Background/Objectives:** Microtubule-targeting agents represent a pillar of cancer chemotherapy; however, their clinical utility is constrained by significant toxicity, pharmacokinetic instability, and susceptibility to multidrug resistance transporters. This study aimed to explore the impact of replacing substituted phenyl rings with a naphthalene moiety in sulfonamide-based colchicine-site ligands, with the goal of identifying new antiproliferative candidates with improved profiles. **Methods:** We designed, synthesized, and evaluated a library of 35 naphthalene sulfonamides bearing varied aryl groups and sulfonamide nitrogen substituents. We assessed the antiproliferative activity against multiple cancer cell lines. Mechanistic studies, including fluorescence microscopy, cell cycle analysis, and cell death assays, were performed to evaluate the effect of these compounds on microtubule polymerization dynamics and cell fate. Molecular docking and in silico pharmacokinetic profiling were carried out to support the proposed binding mode at the colchicine site and to assess drug-likeness. **Results:** Exclusively, compounds bearing a trimethoxyphenyl group showed antiproliferative activity in the submicromolar range, thus identifying it as a structural requirement. The most potent compound (**2**) reached double-digit nanomolar IC_50_ values (67–104 nM) across multiple cancer cell lines. Microscopy confirmed intracellular disruption of microtubule polymerization. Unlike colchicine, these compounds did not induce canonical mitotic arrest but instead triggered apoptotic cell death. In silico analyses supported binding at the colchicine site and revealed favorable predicted pharmacokinetic properties. **Conclusions:** The naphthalene sulfonamides described herein demonstrate potent antiproliferative activity through a distinct mechanism compared to colchicine, and their favorable in silico profiles position them as promising candidates for further development as antitumor agents.

## 1. Introduction

Targeting the polymerization–depolymerization dynamics of microtubules, assembled from repeating units of α- and β-tubulin heterodimers, is a well-established chemotherapeutic strategy. Several clinically approved drugs rely on this mechanism of action to inhibit tumor growth, including paclitaxel, docetaxel, vincristine, and eribulin [1,2]. Paclitaxel and docetaxel are examples of polymerizing agents, which induce microtubule assembly by shifting the equilibrium of tubulin from the soluble form toward the polymerized form. Vincristine and eribulin are depolymerizing agents that disrupt the proper alignment of tubulin dimers, thereby promoting microtubule disassembly or preventing their assembly altogether. Binders to the colchicine-site in tubulin belong to this latter group. Some examples of molecules that bind to the colchicine site include colchicine itself, the benzimidazoles (such as albendazole and mebendazole, which are well-known antiparasitic molecules), combretastatin A-4 (CA-4), and ABT-751 [3]. Ligands targeting the colchicine-binding site offer several advantages over other tubulin-binding drugs such as taxanes or Vinca alkaloids. They are small molecules that are easier to synthesize and often less prone to multidrug resistance (MDR) [3,4]. Moreover, they are reported to act as vascular disrupting agents, affecting the capillaries that supply blood to tumors [5].

**Figure 1:**
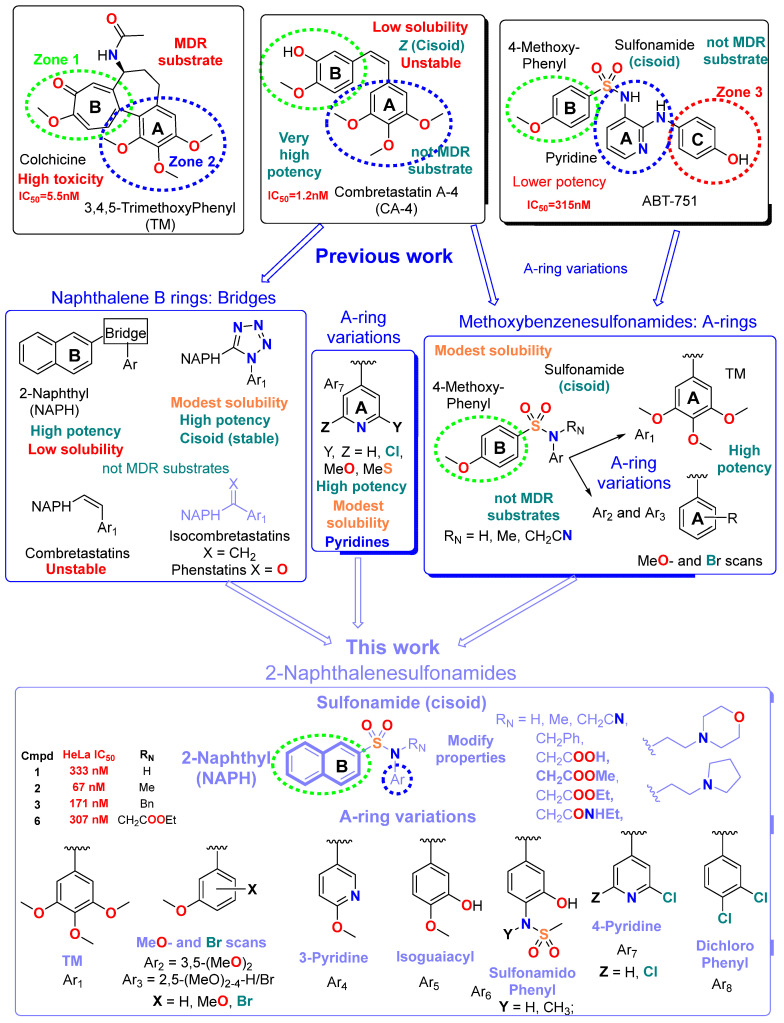
Rationale for the design of this work. The reference colchicine-site ligands are shown above, together with their IC_50_ values in HeLa cells. The middle panel shows the previous results that led to this proposal. For each compound, the structural elements that are mentioned in the main text are indicated, along with the favorable (in green) and undesired (in red or orange) characteristics. The four most active compounds resulting from this work are shown, together with their IC_50_ values in HeLa cells.

The colchicine site is located at the interfacial surface of the αβ tubulin dimers, mostly buried within the β subunit [6]. This site can be subdivided into three sub-pockets, known as Zones I, II, and III [7]. Most colchicine ligands bind to only two of these three zones, but a few, such as ABT-751, are capable of binding to all three [8]. Ligands that bind to Zones I and II usually exhibit higher antitumor potency. To do so, the binding moieties are connected by bridges of different lengths (typically of zero to four atoms) that favor a cisoid disposition [9]: 0-atom bridges (as in colchicine), 1-atom bridges (as in phenstatins or isocombretastatins), 2-atom bridges (as in combretastatins, azoles, or sulfonamides), and bridges with three (as chalcones) or more atoms. Sulfonamide bridges present some characteristics that make them highly advantageous for colchicine-site ligand binding to Zones I and II [4]: they can form various hydrogen bonds, possess a good hydrophobic–hydrophilic balance that can be modified by substituents on the amide nitrogen, and favor the desired *cisoid* arrangement (Figure 1). Zone I occupies the interdimer interface and binds a variety of moieties, such as substituted phenyl rings, such as the *p*-methoxyphenyl rings of CA-4 or ABT-751, the tropolone of colchicine, or fused ring systems such as indoles, carbazoles, or naphthalenes [10]. Zone II occupies a central position in the colchicine binding site, and one of the most common moieties that bind within is a 3,4,5-trimethoxyphenyl ring, which has been associated with high potency. Specifically, the central methoxy group has been associated with both direct and water-mediated hydrogen-bonding interactions with a cysteine amino acid sidechain (βC241) [10]. Successful substitution of the trimethoxyphenyl ring with other moieties, such as pyridine rings, as in ABT-751, or differently substituted alkoxybenzenes, has also been reported [9].

We have previously shown that replacing the *p*-methoxyphenyl ring (ring B) binding at zone I by 5-indole moieties yields highly potent phenstatins, isocombretastatins [11], combretastatins [12], and sulfonamides [13,14]. Further, we have extended this substitution to the fused ring system of 2-naphthalene moieties in phenstatins [15], isocombretastatins [16], combretastatins [17], and azoles [18], with good results. Here, we studied the effect of a naphthalene moiety in sulfonamides, giving naphthalenesulfonamides (Figure 1). Differently substituted phenyls and pyridines [19], which have been shown to afford highly potent colchicine-site binders, especially methoxybenzenesulfonamides, were explored for occupying Zone II: from the trimethoxyphenyl (Ar_1_) [20] to other methoxyphenyl rings (Ar_2_ and Ar_3_) [21], differently substituted pyridines (Ar_4_ and Ar_7_) [22,23], and novel dichlorophenyl (Ar_8_), sulfonamidophenyl (Ar_6_), and isoguaiacyl rings (Ar_5_). Different substitutions known to yield highly potent sulfonamides were introduced at the sulfonamide nitrogen, such as methyl, cyanomethyl, alkoxycarbonylmethyls, and alkylaminocarbonylmethyls, along with larger substituents to explore interactions with the interfacial space and modify pharmacokinetic properties. We evaluated the antiproliferative effect of the naphthalenesulfonamides against several tumorigenic human cancer cell lines, their sensitivity to efflux by MDR pumps, their effects on the cell cycle, and the induction of cell death. We assessed, using immunostaining experiments, the effects of the most antiproliferative compounds on the microtubules within cells, and we studied their binding interactions with tubulin through docking calculations.

## 2. Methods

### 2.1. Chemistry

#### 2.1.1. General Chemical Techniques

All commercially available reagents were used as bought, without additional purification. Dry solvents were maintained over molecular sieves before use. Thin-layer chromatography (TLC) was carried out on precoated silica gel polyester plates (0.25 mm thickness) with a UV fluorescence indicator 254 (Polychrom SI F254, Merck, Darmstadt, Germany). Flash chromatography was conducted using silica gel columns (Kieselgel 40, 0.040–0.063 mm; Merck, Darmstadt, Germany) using the solvent gradients indicated in each case. Preparative chromatography separations were also performed using the specified mobile phase gradients. Crude yields refer to the isolated material after aqueous workup, before further purification; in all cases, ^1^H NMR analysis confirmed the target compound as the major component. NMR spectra were recorded in CDCl_3_, CD_3_OD, and DMSO-d_6_ on a Bruker SY (Bruker, Billerica, MA, USA) spectrometer operating at 400 MHz for ^1^H and 100 MHz for ^13^C. DEPT-135 and DEPT-90 experiments were used to distinguish between C, CH, CH_2_, and CH_3_ signals. Chemical shifts (δ) are reported in parts per million (ppm) relative to the residual solvent signals. Melting points of crystallized compounds were determined using a Cole-Parmer MEL-TEMP model 1101D (Cole-Parmer, Vernon Hills, IL, USA) apparatus and are uncorrected. High-resolution mass spectrometry (HRMS) analyses were performed using a hybrid QSTAR XL quadrupole/time-of-flight (QTOF) (Applied Biosystems/MDS SCIEX, Foster City, CA, USA) mass spectrometer.

#### 2.1.2. General Procedures

- General procedure (A) for the synthesis of sulfonamides.

To a solution of naphthalene-2-sulfonyl chloride (1.0 eq) in CH_2_Cl_2_, the corresponding aniline (1.0 to 1.1 eq) and 250 µL of pyridine were added at room temperature, with constant stirring under an atmosphere of argon. The reaction was controlled by TLC, and after completion, it was poured onto ice and washed twice with 2 M HCl, twice with a 5% NaHCO_3_ solution, and finally with brine until a neutral pH was reached. The resulting solution was dried over anhydrous sodium sulfate, filtered, and vacuum concentrated.

Synthesis of ***N-*(3,4,5-trimethoxyphenyl)naphthalene-2-sulfonamide (1)**.

Following general procedure A, 303 mg of naphthalene-2-sulfonyl chloride (303 mg, 1.120 mmol) and 204 mg of 3,4,5-trimethoxyaniline (204 mg, 1.120 mmol) were stirred for 30 h to give sulfonamide **1**. Yield, crude: 87.4% (364 mg); crystals (mixture of Hexane and CH_2_Cl_2_): 44.4% (207 mg).

**^1^H-NMR (CDCl_3_):** 8.35 (1H, *d*, *J* = 1.6 Hz); 7.88–7.92 (3H, *m*); 7.75 (1H, *dd*, *J* = 2.0 Hz; 8.8 Hz); 7.58–7.66 (2H, *m*); 6.55 (NH, *s*); 6.29 (2H, *s*); 3.75 (3H, *s*); 3.68 (6H, *s*) ppm. **^13^C-NMR (CD_3_OD):** 153.4 (2C); 135.7 (C); 135.6 (C); 134.9 (C); 132.5 (C); 132.0 (C); 129.4 (CH); 129.2 (CH); 129.0 (CH); 129.0 (CH); 127.9 (CH); 127.6 (CH); 122.3 (CH); 99.7 (2CH); 60.9 (CH_3_); 56.1 (2CH_3_) ppm. 1C not found. **M.p.:** 162 °C. **HRMS:** C_19_H_19_NO_5_S. Calculated: 396.0876 (M + Na^+^); found: 396.0875 (M + Na^+^).

Synthesis of ***N-*(3,5-dimethoxyphenyl)naphthalene-2-sulfonamide (11)**.

Following general procedure A, naphthalene-2-sulfonyl chloride (673 mg, 2.969 mmol) and 3,5-dimethoxyaniline (500 mg, 3.266 mmol) were stirred for 3 days to give sulfonamide **11**. Yield, crude: 98.7% (1003 mg); crystals (mixture of Hexane and CH_2_Cl_2_): 46.6% (475 mg).

**^1^H-NMR (CDCl_3_):** 8.41 (1H, *s*); 7.87–7.93 (3H, *m*); 7.76 (1H, *dd*, *J* = 2.0 Hz; 8.8 Hz); 7.57–7.65 (2H, *m*); 6.57 (NH, *s*); 6.26 (2H, *d*, *J* = 2.2 Hz); 6.16 (1H, *t*, *J* = 2.2 Hz); 3.68 (6H, *s*) ppm. **^13^C-NMR (CDCl_3_):** 161.2 (2C); 138.4 (C); 135.8 (C); 134.9 (C); 132.0 (C); 129.5 (CH); 129.3 (CH); 129.1 (CH); 129.0 (CH); 127.9 (CH); 127.5 (CH); 122.3 (CH); 99.0 (2CH); 97.2 (CH); 60.9 (CH_3_); 55.4 (2CH_3_) ppm. **M.p.:** 115 °C. **HRMS:** C_18_H_17_NO_4_S. Calculated: 344.0951 (M + H^+^); found: 344.0947 (M + H^+^).

Synthesis of ***N-*(2,5-dimethoxyphenyl)naphthalene-2-sulfonamide (14)**.

Following general procedure A, naphthalene-2-sulfonyl chloride (445 mg, 1.963 mmol) and 2,5-dimethoxyaniline (330 mg, 2.159 mmol) were stirred for 20 h to give sulfonamide **14**. Yield, crude: 86.5% (583 mg); crystals (mixture of Hexane and CH_2_Cl_2_): 42.3% (285 mg).

**^1^H-NMR (CDCl_3_):** 8.37 (1H, *d*, *J* = 1.6 Hz); 7.84–7.91 (3H, *m*); 7.75 (1H, *dd*, *J* = 1.6 Hz; 8.4 Hz); 7.55–7.63 (2H, *m*); 7.21 (1H, *d*, *J* = 3.4 Hz); 7.14 (NH, *s*); 6.61 (1H, *d*, *J* = 8.8 Hz); 6.51 (1H, *dd*, *J* = 3.2 Hz; 8.8 Hz); 3.74 (3H, *s*); 3.53 (3H, *s*) ppm. **^13^C-NMR (CDCl_3_):** 153.8 (C); 143.7 (C); 136.0 (C); 134.8 (C); 131.9 (C); 129.2 (CH); 129.1 (CH); 128.9 (CH); 128.8 (CH); 127.8 (CH); 127.5 (CH); 126.5 (C); 122.3 (CH); 111.5 (CH); 109.6 (CH); 107.4 (CH); 56.1 (CH_3_); 55.7 (CH_3_) ppm. **M.p.:** 159 °C. **HRMS:** C_18_H_17_NO_4_S. Calculated: 366.0770 (M + Na^+^); found: 366.0769 (M + Na^+^).

Synthesis of ***N-*(6-methoxypyridin-3-yl)naphthalene-2-sulfonamide (18)**.

Following general procedure A, naphthalene-2-sulfonyl chloride (1050 mg, 4.632 mmol) and 6-methoxypyridin-3-amine (632 mg, 5.095 mmol) were stirred for 24 h to give sulfonamide **18**. Yield, crude: 73.4% (1068 mg); crystals (mixture of Hexane and CH_2_Cl_2_): 8.5% (124 mg).

**^1^H-NMR (CDCl_3_):** 8.30 (1H, *s*); 7.88 (1H, *d*, *J* = 8.4 Hz); 7.82–7.86 (2H, *m*); 7.76 (1H, *d*, *J* = 7.2 Hz); 7.61 (1H, *t*, *J* = 7.6 Hz); 7.55 (1H, *t*, *J* = 8.0 Hz); 7.42 (1H, *dd*, *J* = 1.6 Hz; 7.6 Hz); 7.33 (NH, *bs*); 6.60 (1H, *d*, *J* = 8.4 Hz); 3.81 (3H, *s*) ppm. **^13^C-NMR (CDCl_3_):** 162.5 (C); 142.7 (CH); 135.9 (CH); 135.4 (C); 134.9 (C); 132.0 (C); 129.6 (CH); 129.3 (CH); 129.0 (CH); 129.0 (CH); 127.9 (CH); 127.6 (CH); 126.5 (C); 122.2 (CH); 111.1 (CH); 53.7 (CH_3_) ppm. **M.p.:** 147 °C. **HRMS:** C_16_H_14_N_2_O_3_S. Calculated: 315.0798 (M + H^+^); found: 315.0795 (M + H^+^).

Synthesis of ***N-*(3-hydroxy-4-methoxyphenyl)naphthalene-2-sulfonamide (21)**.

Following general procedure A, naphthalene-2-sulfonyl chloride (250 mg, 1.103 mmol) and 5-amino-2-methoxyphenol (170 mg, 1.221 mmol) were stirred for 24 h to give sulfonamide **21**. Yield, crude: 79.5% (289 mg); crystals (mixture of Hexane, CH_2_Cl_2_, and MeOH): 14.0% (51 mg).

**^1^H-NMR (CDCl_3_):** 8.32 (1H, *s*); 7.86–7.90 (3H, *m*); 7.71 (1H, *dd*, *J* = 1.6 Hz; 8.8 Hz); 7.56–7.65 (2H, *m*); 6.65–6.68 (2H, *m*); 6.58 (1H, *dd*, *J* = 2.4 Hz; 8.8 Hz); 6.28 (NH, *bs*); 5.54 (OH, *bs*); 3.81 (3H, *s*) ppm. **^13^C-NMR (CD_3_OD):** 146.5 (C); 145.6 (C); 136.5 (C); 134.8 (C); 132.0 (C);130.5 (C); 128.7 (CH); 128.7 (CH); 128.4 (CH); 128.0 (CH); 127.5 (CH); 127.1 (CH); 122.2 (CH); 113.8 (CH); 111.4 (CH); 110.5 (CH); 55.1 (CH_3_) ppm. **M.p.:** 190 °C. **HRMS:** C_17_H_15_NO_4_S. Calculated: 330.0790 (M + H^+^); found: 330.0790 (M + H^+^).

Synthesis of ***N-*(3-methoxy-4-(methylsulfonamido)phenyl)naphthalene-2-sulfonamide (22)**.

Following general procedure A, naphthalene-2-sulfonyl chloride (150 mg, 0.662 mmol) and *N*-(4-amino-2-methoxyphenyl)methanesulfonamide (**P2**) (157 mg, 0.726 mmol) were stirred for 48 h to give sulfonamide **22**. Yield, crude: 94.4% (254 mg); crystals (mixture of Hexane, CH_2_Cl_2_, and MeOH): 57.2% (134 mg).

**^1^H-NMR (CDCl_3_):** 8.35 (NH, *bs*); 7.88–7.93 (3H, *m*); 7.73 (1H, *d*, *J* = 9.2 Hz); 7.58–7.67 (2H, *m*); 7.30 (1H, *d*, *J* = 8.4 Hz); 6.89 (1H, *d*, *J* = 1.6 Hz); 6.61 (2H, *d*, *J* = 8.0 Hz); 6.49 (1H, *dd*, *J* = 2.0 Hz; 8.8 Hz); 3.81 (3H, *s*); 2.86 (3H, *s*) ppm. **^13^C-NMR (DMSOd_6_):** 153.8 (C); 137.0 (C); 136.8 (C); 134.7 (C); 132.0 (C); 130.0 (CH); 129.7 (CH); 129.5 (CH); 128.6 (CH); 128.3 (CH); 128.2 (CH); 127.7 (CH); 122.5 (CH); 122.1 (C); 112.2 (CH); 104.4 (CH); 56.1 (CH_3_); 40.4 (CH_3_) ppm. **M.p.:** 195 °C. **HRMS:** C_18_H_18_N_2_O_5_S_2_. Calculated: 424.0995 (M + NH_4_^+^); found: 424.0992 (M + NH_4_^+^).

Synthesis of ***N-*(3-methoxy-4-(*N-*methylmethylsulfonamido)phenyl)naphthalene-2-sulfonamide (24)**.

Following general procedure A, naphthalene-2-sulfonyl chloride (110 mg, 0.485 mmol) and *N-*(4-amino-2-methoxyphenyl)*-N-*methylmethanesulfonamide (**P4**) (120 mg, 0.522 mmol) were stirred for 48 h to give sulfonamide **24**. Yield, crude: 58.8% (120 mg); crystals (mixture of Hexane and CH_2_Cl_2_): 18.1% (37 mg).

**^1^H-NMR (CDCl_3_):** 8.39 (1H, *s*); 7.88–7.94 (3H, *m*); 7.76 (1H, *dd*, *J* = 2.4 Hz; 9.2 Hz); 7.59–7.67 (2H, *m*); 7.15 (1H, *d*, *J* = 8.8 Hz); 6.86 (1H, *d*, *J* = 2.4 Hz); 6.77 (NH, *bs*); 6.47 (1H, *dd*, *J* = 2.4 Hz; 8.4 Hz); 3.81 (3H, *s*); 3.16 (3H, *s*); 2.88 (3H, *s*) ppm. **^13^C-NMR (CDCl_3_):** 156.5 (C); 138.0 (C); 136.0 (C); 135.0 (C);132.8 (CH); 132.0 (C); 129.6 (CH); 129.3 (CH); 129.1 (CH); 128.9 (CH); 128.0 (CH); 127.7 (CH); 125.7 (C); 122.1 (CH); 113.1 (CH); 105.0 (CH); 55.6 (CH_3_); 38.1 (CH_3_); 37.5 (CH_3_) ppm. **M.p.:** 184 °C. **HRMS:** C_19_H_20_N_2_O_5_S_2_. Calculated: 443.0706 (M + Na^+^); found: 443.0702 (M + Na^+^).

Synthesis of ***N-*(2-chloropyridin-4-yl)naphthalene-2-sulfonamide (26)**.

Following general procedure A, naphthalene-2-sulfonyl chloride (104 mg, 0.458 mmol) and 2-chloropyridin-4-amine (65 mg, 0.505 mmol) were stirred for 5 days to give sulfonamide **26**. Yield, crude: 39.0% (57 mg); crystals (mixture of Hexane and CH_2_Cl_2_): 19.9% (29 mg).

**^1^H-NMR (CDCl_3_):** 8.55 (1H, *s*); 8.16 (1H, *d*, *J* = 5.6 Hz); 7.93 (2H, *t*, *J* = 8.8 Hz); 7.85 (2H, *t*, *J* = 8.0 Hz); 7.65 (1H, *t*, 8.0 Hz); 7.60 (1H, *t*, *J* = 8.0 Hz); 7.18 (1H, *bs*); 7.08 (1H, *dd*, *J* = 2.0 Hz; *J* = 6.0 Hz) ppm. **^13^C-NMR (CDCl_3_):** 152.1 (C); 150.1 (CH); 147.2 (C); 135.2 (C); 135.2 (C); 131.9 (C); 130.1 (CH); 129.6 (CH); 129.4 (CH); 129.2 (CH); 128.0 (CH); 128.0 (CH); 121.6 (CH); 112.3 (CH); 111.4 (CH) ppm. **M.p.:** 167 °C. **HRMS:** C_15_H_11_ClN_2_O_2_S. Calculated: 319.0303 (M + H^+^); found: 319.0301 (M + H^+^).

Synthesis of ***N-*(2,6-dichloropyridin-4-yl)naphthalene-2-sulfonamide (30)**.

Following general procedure A, naphthalene-2-sulfonyl chloride (110 mg, 0.485 mmol) and 2,6-dichloropyridin-4-amine (87 mg, 0.534 mmol) were stirred for 5 days to give sulfonamide **30**. Yield, crude: 15.0% (26 mg); crystals (mixture of Hexane and CH_2_Cl_2_): 7.6% (13 mg).

**^1^H-NMR (CDCl_3_):** 8.50 (1H, *d*, *J* = 1.2 Hz); 8.06 (1H, *d*, *J* = 8.8 Hz); 7.98–8.02 (2H, *m*); 7.90 (1H, *dd*, *J* = 1.2 Hz; *J* = 8.8 Hz); 7.76 (1H, *t*, *J* = 7.6 Hz); 7.69 (1H, *t*, *J* = 7.6 Hz); 7.02 (2H, *d*, *J* = 0.8 Hz); 3.64 (NH, *s*) ppm. **^13^C-NMR (DMSOd):** 150.7 (2C); 145.9 (C); 135.7 (C); 134.6 (C); 131.9 (CH); 131.3 (CH); 130.8 (CH); 130.4 (C); 130.3 (CH); 128.6 (CH); 128.6 (CH); 126.5 (CH); 123.0 (2CH) ppm. **M.p.:** 200 °C. **HRMS:** C_15_H_10_Cl_2_N_2_O_2_S. Calculated: 350.9767 (M-H^+^); found: 350.9765 (M-H^+^).

Synthesis of ***N-*****(3,4-dichlorophenyl)naphthalene-2-sulfonamide (32)**.

Following general procedure A, naphthalene-2-sulfonyl chloride (1.017 mg, 4.480 mmol) and 3,4-dichloroaniline (846 mg, 5.220 mmol) were stirred for 5 days to give sulfonamide **32**. Yield, crude: 92.0% (1.451 mg); crystals (mixture of Hexane and CH_2_Cl_2_): 72.2% (1.139 mg).

**^1^H-NMR (CDCl_3_):** 8.31 (1H, *d*, *J* = 2.0 Hz); 7.85–7.87 (2H, *m*); 7.82 (1H, *d*, *J* = 8.4 Hz); 7.65 (1H, *dd*, *J* = 2.0 Hz; 8.4 Hz); 7.52–7.61 (2H, *m*); 7.20 (1H, *d*, *J* = 8.8 Hz); 7.17 (1H, *d*, *J* = 2.4 Hz); 6.88 (1H, *dd*, *J* = 2.4 Hz; 8.8 Hz). **^13^C-NMR (CDCl_3_):** 136.0 (C); 135.2 (C); 135.1 (C); 133.2 (C); 132.0 (C); 130.9 (CH); 129.9 (CH); 129.4 (CH); 129.3 (CH); 129.1 (C); 129.1 (CH); 127.9 (CH); 127.8 (CH); 122.8 (CH); 121.8 (CH); 120.3 (CH) ppm. **M.p.:** 140 °C. **HRMS:** C_16_H_11_Cl_2_NO_2_S. Calculated: 349.9815 (M-H^+^); found: 349.9814 (M-H^+^).

- General synthetic procedure (B) for the methylation of sulfonamide using Me_2_SO_4_.

To a solution of the sulfonamide (1.0 eq) in a CH_2_Cl_2_/H_2_O solution (1:1), and at a temperature of 0 °C, Me_2_SO_4_ (6.0 eq), catalytic amounts of tetrabutylammonium bromide, and 100 mg of crushed sodium hydroxide were added with continuous stirring. The reaction was monitored by TLC until completion. The crude reaction mixture was then poured onto ice and washed with brine until a neutral pH was obtained. The resulting solution was dried over anhydrous sodium sulfate, filtered, and vacuum concentrated.

Synthesis of ***N-*methyl*-N-*(3,4,5-trimethoxyphenyl)naphthalene-2-sulfonamide (2)**.

Following general procedure B, *N-*(3,4,5-trimethoxyphenyl)naphthalene-2-sulfonamide (**1**) (250 mg, 0.670 mmol) was treated with Me_2_SO_4_ (446 mg, 4.017 mmol) and stirred for 24 h to give sulfonamide **2**. Yield, crude: 76.3% (198 mg); crystals (mixture of Hexane and CH_2_Cl_2_): 56.6% (147 mg).

**^1^H-NMR (CDCl_3_):** 8.21 (1H, *d*, *J* = 1.2 Hz); 7.91 (3H, *d*, *J* = 7.6 Hz); 7.56–7.67 (3H, *m*); 6.25 (2H, *s*); 3.82 (3H, *s*); 3.62 (6H, *s*); 3.18 (3H, *s*) ppm. **^13^C-NMR (CDCl_3_):** 153.0 (2C); 137.6 (C); 137.1 (C); 134.8 (C); 133.6 (C); 132.0 (C); 129.4 (CH); 129.2 (CH); 128.9 (CH); 128.7 (CH); 127.9 (CH); 127.6 (CH); 123.4 (CH); 104.7 (2x CH); 60.9 (CH_3_); 56,1 (2CH_3_); 38.7 (CH_3_) ppm. **M.p.:** 143 °C. **HRMS:** C_20_H_21_NO_5_S. Calculated: 410.1030 (M + Na^+^); found: 410.1030 (M + Na^+^).

Synthesis of ***N-*(3,5-dimethoxyphenyl)-*N-*methylnaphthalene-2-sulfonamide (12)**.

Following general procedure B, *N-*(3,5-dimethoxyphenyl)naphthalene-2-sulfonamide (**11**) (148 mg, 0.431 mmol) was treated with Me_2_SO_4_ (326 mg, 2.586 mmol) and stirred for 20 h to give sulfonamide **12**. Yield, crude: 97.3% (144 mg); flash chromatography with Hexane/EtOAc (8:2): 64.2% (95 mg).

**^1^H-NMR (CDCl_3_):** 8.24 (1H, *s*); 7.88–7.93 (3H, *m*); 7.54–7.65 (3H, *m*); 6.26 (2H, *d*, *J* = 1.6 Hz); 6.16 (1H, *t*, *J* = 1.6 Hz); 3.70 (6H, *s*); 3.19 (3H, *s*) ppm. **^13^C-NMR (CDCl_3_):** 160.6 (2C); 143.3 (C); 134.9 (C); 133.7 (C); 132.0 (C);129.2 (CH); 129.2 (CH); 128.9 (CH); 128.8 (CH); 127.9 (CH); 127.5 (CH); 123.2 (CH); 105.0 (2CH); 99.7 (CH); 55.4 (2CH_3_); 38.4 (CH_3_) ppm. **HRMS:** C_19_H_19_NO_4_S. Calculated: 380.0927 (M + Na^+^); found: 380.0920 (M + Na^+^).

- General synthetic procedure (C) for the methylation with MeI of the sulfonamide.

To a solution of the sulfonamide (1.0 eq) dissolved in CH_3_CN at room temperature, MeI (2.0 eq) and Cs_2_CO_3_ (1.5 to 4.0 eq) were added, with continuous stirring. The reaction was controlled by TLC until completion. The crude reaction mixture was then poured onto ice, diluted with a large volume of CH_2_Cl_2_, and washed with brine until a neutral pH was reached. The resulting solution was dried over anhydrous sodium sulfate, filtered, and vacuum concentrated.

Synthesis of ***N-*(2-methoxy-4-nitrophenyl)*-N-*methylmethanesulfonamide (P2)**.

Following general procedure C, *N-*(2-methoxy-4-nitrophenyl)methanesulfonamide (**P1**) (340 mg, 1.381 mmol) was treated with MeI (389 mg, 2.762 mmol) and Cs_2_CO_3_ (389 mg, 2.762 mmol) and stirred for 5 days to give precursor **P2**. Yield, crude: 80.7% (290 mg); no further purification.

**^1^H-NMR (CDCl_3_):** 7.82 (1H, *dd*, *J* = 2.4 Hz; 8.8 Hz); 7.80 (1H, *d*, *J* = 2.4 Hz); 7.50 (1H, *d*, *J* = 8.0 Hz); 4.00 (3H, *s*); 3.26 (3H, *s*); 2.96 (3H, *s*) ppm. **^13^C-NMR (CDCl_3_):** 156.3 (C); 148.1 (C); 135.2 (C); 132.6 (CH); 116.3 (CH); 107.2 (CH); 56.3 (CH_3_); 38.4 (CH_3_); 37.4 (CH_3_) ppm.

Synthesis of ***N-*(2,5-dimethoxyphenyl)-*N-*methylnaphthalene-2-sulfonamide (15)**.

Following general procedure C, *N-*(2,5-dimethoxyphenyl)naphthalene-2-sulfonamide (**14**) (200 mg, 0.582 mmol) was treated with MeI (165 mg, 1.165 mmol) and Cs_2_CO_3_ (190 mg, 0.582 mmol) and stirred for 3 days to give sulfonamide **15**. Yield, crude: 51.0% (106 mg); crystals (mixture of Hexane and CH_2_Cl_2_): 21.6% (45 mg).

**^1^H-NMR (CDCl_3_):** 8.26 (1H, *s*); 7.90–7.92 (3H, *m*); 7.72 (1H, *dd*, *J* = 1.6 Hz; 8.4 Hz); 7.56–7.64 (2H, *m*); 6.93 (1H, *d*, *J* = 2.4 Hz); 6.83 (1H, *dd*, *J* = 3.2 Hz; 9.2 Hz); 6.67 (1H, *d*, *J* = 2.8 Hz); 3.75 (3H, *s*); 3.25 (3H, *s*); 3.15 (3H, *s*) ppm. **^13^C-NMR (CD_3_OD):** 153.8 (C); 147.3 (C); 142.4 (C); 137.5 (C); 134.0 (C); 132.4 (C); 128.4 (CH); 127.7 (CH); 127.3 (CH); 127.0 (CH); 126.4 (CH); 126.3 (CH); 123.6 (CH); 111.5 (CH); 108.6 (CH); 104.0 (CH); 55.3 (CH_3_); 54.4 (CH_3_); 47.6 (CH_3_) ppm. **M.p.:** 117 °C. **HRMS:** C_19_H_19_NO_4_S. Calculated: 380.0927 (M + Na^+^); found: 380.0918 (M + Na^+^).

Synthesis of ***N-*(6-methoxypyridin-3-yl)*-N-*methylnaphthalene-2-sulfonamide (19)**.

Following general procedure C, *N-*(6-methoxypyridin-3-yl)naphthalene-2-sulfonamide (**18**) (100 mg, 0.318 mmol) was treated with MeI (90 mg, 0.636 mmol) and Cs_2_CO_3_ (155 mg, 0.477 mmol) and stirred for 48 h to give sulfonamide **19**. Yield, crude: quantitative (108 mg); preparative chromatography with Hexane/EtOAc (7:3): 54.6% (57 mg).

**^1^H-NMR (CDCl_3_):** 8.18 (1H, *bs*); 7.89 (3H,d, *J* = 8.4 Hz); 7.85 (1H, *bs*); 7.63 (1H, *t*, *J* = 7.6 Hz); 7.58 (1H, *t*, *J* = 7.6 Hz); 7.52 (1H, *d*, *J* = 8.8 Hz); 7.30 (1H, *dd*, *J* = 1.6 Hz; *J* = 8.8 Hz); 6.64 (1H, *dd*, *J* = 1.2 Hz; *J* = 8.4 Hz); 3.88 (3H, *s*); 3.19 (3H,s) ppm. **^13^C-NMR (CDCl_3_):** 163.0 (C); 145.3 (CH); 137.6 (CH); 134.9 (C); 133.5 (C); 132.0 (C); 131.8 (C); 129.2 (CH); 129.2 (CH); 129.2 (CH); 129.0 (CH); 127.9 (CH); 127.6 (CH); 123.0 (CH); 110.9 (CH); 53.7 (CH_3_); 38.6 (CH_3_) ppm. **HRMS:** C_16_H_14_N_2_O_3_S. Calculated: 329.0954 (M + H^+^); found: 329.0947 (M + H^+^).

Synthesis of ***N-*(3-methoxy-4-(methylsulfonamido)phenyl)*-N-*methylnaphthalene-2-sulfonamide (23)**.

Following general procedure C, *N-*(3-methoxy-4-(methylsulfonamido)phenyl)naphthalene-2-sulfonamide (**22**) (155 mg, 0.381 mmol) was treated with MeI (65 mg, 0.458 mmol) and Cs_2_CO_3_ (250 mg, 0.767 mmol) and stirred for 48 h to give sulfonamide **23**. Yield, crude: 64.3% (103 mg); preparative chromatography with CH_2_Cl_2_/MeOH (98:2): 11.9% (19 mg).

**^1^H-NMR (CDCl_3_):** 8.20 (1H, *d*, *J* = 1.2 Hz); 7.89–7.92 (3H, *m*); 7.66 (1H, *dt*, *J* = 1.2 Hz; 6.4 Hz); 7.59 (1H, *dt*, *J* = 1.6 Hz; 6.8 Hz); 7.51 (1H, *dd*, *J* = 2.0 Hz; 8.4 Hz); 7.36 (1H, *d*, *J* = 8.8 Hz); 6.94 (1H, *d*, *J* = 2.0 Hz); 6.80 (NH, sa); 6.44 (1H, *dd*, *J* = 2.4 Hz; 8.4 Hz); 3.82 (3H, *s*); 3.19 (3H, *s*); 2.96 (3H, *s*) ppm. **^13^C-NMR (CDCl_3_):** 149.2 (C); 138.8 (C); 134.9 (C); 133.4 (C); 132.0 (C);129.2 (CH); 129.2 (CH); 129.0 (CH); 129.0 (CH); 128.0 (CH); 127.6 (CH); 125.4 (C); 123.1 (CH); 120.0 (CH); 118.2 (CH); 111.4 (CH); 56.0 (CH_3_); 39.3 (CH_3_); 38.4 (CH_3_) ppm. **HRMS:** C_19_H_20_N_2_O_5_S_2_. Calculated: 421.0886 (M + H^+^); found: 421.0885 (M + H^+^).

Synthesis of ***N-*(3-methoxy-4-(*N-*methylmethylsulfonamido)phenyl)*-N-*methylnaphthalene-2-sulfonamide (25)**.

Following general procedure C, *N-*(3-methoxy-4-(*N-*methylmethylsulfonamido)phenyl)naphthalene-2-sulfonamide (**24**) (77 mg, 0.189 mmol) was treated with MeI (130 mg, 0.916 mmol) and Cs_2_CO_3_ (123 mg, 0.379 mmol) and stirred for 5 days to give sulfonamide **25**. Yield, crude: 92.5% (76 mg); preparative chromatography with CH_2_Cl_2_/MeOH (98:2): 74.3% (61 mg).

**^1^H-NMR (CDCl_3_):** 8.21 (1H, *d*, *J* = 0.8 Hz); 7.92 (2H, *d*, *J* = 8.4 Hz); 7.60–7.68 (2H, *m*); 7.52 (1H, *dd*, *J* = 2.0 Hz; 8.8 Hz); 7.22 (1H, *d*, *J* = 8.0 Hz); 7.01 (1H, *d*, *J* = 2.0 Hz); 6.43 (1H, *dd*, *J* = 2.4 Hz; 8.0 Hz); 3.84 (3H, *s*); 3.25 (3H, *s*); 3.19 (3H, *s*); 2.94 (3H, *s*) ppm. **^13^C-NMR (CDCl_3_):** 156.0 (C); 142.3 (C); 134.9 (C); 133.4 (C); 132.3 (CH); 132.0 (C); 129.3 (CH); 129.2 (CH); 129.0 (CH); 129.0 (CH); 128.1 (C); 127.9 (CH); 127.6 (CH); 123.0 (CH); 117.5 (CH); 112.3 (CH); 55.7 (CH_3_); 38.2 (CH_3_); 37.6 (CH_3_) ppm. **HRMS:** C_20_H_22_N_2_O_5_S_2_. Calculated: 435.1043 (M + H^+^); found: 435.1038 (M + H^+^).

Synthesis of ***N-*(2-chloropyridin-4-yl)*-N-*methylnaphthalene-2-sulfonamide (27)** and **2-chloro-1-methyl-4-(naphthalene-2-sulfonamido)pyridin-1-ium (28)**.

Following general procedure C, *N-*(2-chloropyridin-4-yl)naphthalene-2-sulfonamide (**26**) (103 mg, 0.323 mmol) was treated with MeI (91 mg, 0.646 mmol) and Cs_2_CO_3_ (158 mg, 0.482 mmol) and stirred for 48 h to give sulfonamide **27** and **28** in a proportion 6:4. Yield, crude: 96.0% (103 mg); preparative chromatography with Hexane/EtOAc (7:3):

***N-*(2-chloropyridin-4-yl)*-N-*methylnaphthalene-2-sulfonamide (27):** 54.0% (58 mg).

**^1^H-NMR (CDCl_3_):** 8.25–8.28 (2H, *m*); 7.90–7.96 (3H, *m*); 7.61–7.69 (2H, *m*); 7.50 (1H, *dd*, *J* = 2.0 Hz; 8.8 Hz); 7.20–7.22 (2H, *m*); 3.28 (3H, *s*) ppm. **^13^C-NMR (CDCl_3_):** 152.0 (C); 150.7 (C); 149.7 (CH); 134.9 (C); 133.1 (C); 131.7 (C); 129.4 (CH); 129.2 (CH); 129.1 (CH); 128.9 (CH); 127.7 (CH); 127.7 (CH); 121.7 (CH); 117.2 (CH); 116.1 (CH); 36.3 (CH_3_) ppm. **HRMS:** C_16_H_13_ClN_2_O_2_S. Calculated: 333.0459 (M + H^+^); found: 333.0458 (M-H^+^).

**2-chloro-1-methyl-4-(naphthalene-2-sulfonamido)pyridin-1-ium (28):** 39.0% (42 mg).

**^1^H-NMR (CDCl_3_):** 8.27 (1H, *s*); 8.24 (1H, *dd*, *J* = 0.8 Hz; 5.6 Hz); 7.88–7.94 (3H, *m*); 7.59–7.65 (2H, *m*); 7.49 (1H, *dd*, *J* = 1.6 Hz; 8.8 Hz); 7.18–7.20 (2H, m); 3.27 (3H, *s*) ppm. **^13^C-NMR (CDCl_3_):** 162.8 (C); 142.4 (C); 141.9 (CH); 140.1 (C); 134.4 (C); 132.2 (C); 129.1 (CH); 128.9 (CH); 128.0 (CH); 127.8 (CH); 127.0 (CH); 126.8 (CH); 122.9 (CH); 116.1 (CH); 115.5 (CH); 43.2 (CH_3_) ppm. **HRMS:** C_16_H_13_ClN_2_O_2_S. Calculated: 333.0459 (M + H^+^); found: 333.0462 (M + H^+^).

Synthesis of ***N-*(2,6-dichloropyridin-4-yl)*-N-*methylnaphthalene-2-sulfonamide (31)**.

Following general procedure C, N-(6-methoxypyridin-3-yl)naphthalene-2-sulfonamide (**30**) (100 mg, 0.318 mmol) was treated with MeI (90 mg, 0.636 mmol) and Cs_2_CO_3_ (155 mg, 0.477 mmol) and stirred for 48 h to give sulfonamide **31**. Yield, crude: 78.2% (127 mg); crystals (MeOH): 9.9% (16 mg).

**^1^H-NMR (CDCl_3_):** 8.33 (1H, *s*); 7.92–7.99 (3H, *m*); 7.64–7.72 (2H, *m*); 7.53 (1H, *d*, *J* = 8.0 Hz); 7.23 (2H, *d*, *J* = 1.6 Hz); 3.30 (3H, *s*) ppm. **^13^C-NMR (CDCl_3_):** 152.4 (C); 151.1 (C); 135.2 (C); 133.2 (C); 131.9 (C); 129.9 (CH); 129.6 (CH); 129.4 (CH); 129.3 (CH); 128.1 (CH); 128.0 (CH); 121.7 (CH); 115.6 (2CH); 36.5 (CH_3_) ppm. **M.p.:** 152 °C. **HRMS:** C_16_H_12_Cl_2_N_2_O_2_S. Calculated: 367.0067 (M + H^+^); found: 367.0067 (M + H^+^).

Synthesis of ***N-*****(3,4-dichlorophenyl)-*N-*methylnaphthalene-2-sulfonamide (33)**.

Following general procedure C, *N-*(3,4-dichlorophenyl)naphthalene-2-sulfonamide (**32**) (150 mg, 0.426 mmol) was treated with MeI (20 mg, 1.446 mmol) and Cs_2_CO_3_ (180 mg, 0.554 mmol) and stirred for 4 days to give sulfonamide **32**. Yield, crude: 77.5% (121 mg); crystals (MeOH): 37.8% (58 mg).

**^1^H-NMR (CDCl_3_):** 8.21 (1H, *d*, *J* = 1.2 Hz); 7.90–7.95 (3H, *m*); 7.61–7.70 (2H, *m*); 7.47 (1H, *dd*, *J* = 2.0 Hz; 8.8 Hz); 7.36 (1H, *d*, *J* = 8.8 Hz); 7.23 (1H, *d*, *J* = 2.4 Hz); 7.00 (1H, *dd*, *J* = 2.4 Hz; 8.4 Hz); 3.18 (3H, *s*). **^13^C-NMR (CDCl_3_):** 141.0 (C); 135.0 (C); 133.0 (C); 132.7 (C); 132.0 (C); 131.4 (C); 130.4 (CH); 129.3 (CH); 129.2 (CH); 129.2 (CH); 129.1 (CH); 128.4 (CH); 128.0 (CH); 127.7 (CH); 125.7 (CH); 122.8 (CH); 38.0 (CH_3_) ppm. **M.p.:** 165 °C. **HRMS:** C_17_H_13_Cl_2_NO_2_S. Calculated: 387.9936 (M + Na^+^); found: 387.9932 (M + Na^+^).

- General synthetic procedure (D) for the *N-*alkylation of the sulfonamide using Cs_2_CO_3_.

To a solution of the sulfonamide (1.0 eq) dissolved in CH_3_CN at room temperature, the corresponding halogenated derivative (1.0 to 3.0 eq), Cs_2_CO_3_ (1.0 to 2.0 eq), and a catalytic amount of KI were added, with continuous stirring. The reaction was monitored by TLC until completion. The crude reaction mixture was then poured onto ice, diluted with a large volume of CH_2_Cl_2_, and washed with brine until a neutral pH was reached. The resulting solution was dried over anhydrous sodium sulfate, filtered, and vacuum concentrated.

Synthesis of ***N-*(cyanomethyl)*-N-*(3,4,5-trimethoxyphenyl)naphthalene-2-sulfonamide (3)**.

Following general procedure D, *N-*(3,4,5-trimethoxyphenyl)naphthalene-2-sulfonamide (**1**) (300 mg, 0.803 mmol) was treated with 2-chloroacetonitrile (181 mg, 2.410 mmol) and Cs_2_CO_3_ (524 mg, 0.803 mmol) and stirred for 24 h to give sulfonamide **3**. Yield, crude: 75.0% (249 mg); crystals (mixture of Hexane and EtOAc): 60.0% (192 mg).

**^1^H-NMR (CDCl_3_):** 8.30 (1H, *d*, *J* = 1.6 Hz); 7.98 (1H, *d*, *J* = 8.8 Hz); 7.93 (1H, *d*, *J* = 8.8 Hz); 7.60–7.74 (4H, *m*); 6.34 (2H, *s*); 4.61 (2H, *s*); 3.83 (3H, *s*); 3.60 (6H, *s*) ppm. **^13^C-NMR (CDCl_3_):** 153.5 (2C); 138.9 (C); 135.1 (C); 134.2 (C); 133.6 (C); 131.9 (C); 129.9 (CH); 129.4 (CH); 129.3 (CH); 127.9 (CH); 127.9 (CH); 122.8 (CH); 115.0 (C); 106.1 (2CH); 60.9 (CH_3_); 56.0 (2CH_3_); 40.0 (CH_2_) ppm. **M.p.:** 174 °C. **HRMS:** C_21_H_20_N_2_O_5_S. Calculated: 413.1166 (M + H^+^); found: 413.1160 (M + H^+^).

Synthesis of **ethyl *N-*(naphthalen-2-ylsulfonyl)-*N-*(3,4,5-trimethoxyphenyl)glycinate (6)**.

Following general procedure D, *N-*(3,4,5-trimethoxyphenyl)naphthalene-2-sulfonamide (**1**) (482 mg, 1.290 mmol) was treated with ethyl bromoacetate (649 mg, 3.900 mmol) and Cs_2_CO_3_ (524 mg, 0.803 mmol) and stirred for 24 h to give sulfonamide **6**. Yield, crude: 84.5% (499 mg); crystals (mixture of Hexane and EtOAc): 61.1% (362 mg).

**^1^H-NMR (CDCl_3_):** 8.27 (1H, *d*, *J* = 1.6 Hz); 7.89–7.95 (3H, *m*); 7.75 (1H, *dd*, *J* = 2.0 Hz; 8.4 Hz); 7.64 (1H, *dd*, *J* = 1.6 Hz; 8.0 Hz); 7.60 (1H, *dd*, *J* = 1.6 Hz; 8.0 Hz); 6.41 (2H, *s*); 4.43 (2H, *s*); 4.16 (2H, *c*, *J* = 7.2 Hz; 1.4 Hz); 3.81 (3H, *s*); 3.60 (6H, *s*); 1.23 (3H, *t*, *J* = 7.2 Hz). **^13^C-NMR (CDCl_3_):** 168.8 (C); 153.1 (2C); 138.1 (C); 135.8 (C); 135.2 (C); 134.8 (C); 131.9 (C); 129.3 (CH); 129.1 (CH); 128.9 (CH); 128.8 (CH); 127.8 (CH); 127.6 (CH); 123.1 (CH); 106.7 (2CH); 61.5 (CH_2_); 60.8 (CH_3_); 56.0 (2CH_3_); 53.1 (CH_2_); 14.1 (CH_3_) ppm. **M.p.:** 167 °C. **HRMS:** C_23_H_25_NO_7_S. Calculated: 482.1244 (M + Na^+^); found: 482.1237 (M + Na^+^).

Synthesis of ***N-*(2-morpholinoethyl)-*N-*(3,4,5-trimethoxyphenyl)naphthalene-2-sulfonamide (10)**.

Following general procedure D, *N-*(3,4,5-trimethoxyphenyl)naphthalene-2-sulfonamide (**1**) (254 mg, 0.680 mmol) was treated with 4-(2-chloroethyl)morpholine (204 mg, 1.360 mmol) and Cs_2_CO_3_ (443 mg, 1.360 mmol) and stirred for 5 days sulfonamide **10**. Yield, crude: 64.6% (214 mg); crystals (MeOH): 30.8% (102 mg).

**^1^H-NMR (CDCl_3_):** 8.23 (1H, *s*); 7.90–7.94 (3H, *m*); 7.59–7.70 (3H, *m*); 6.22 (2H, *s*); 3.84 (3H, *s*); 3.70 (6H, *m*); 3.60 (6H, *s*); 2.48 (2H, *m*) ppm. **^13^C-NMR (CDCl_3_):** 153.0 (2C); 138.0 (C); 135.2 (C); 134.7 (C); 134.7 (C); 132.0 (C); 129.1 (2CH); 128.9 (CH); 128.9 (C); 127.8 (CH); 127.6 (CH); 123.2 (CH); 106.7 (2CH); 66.9 (2CH_2_); 60.9 (CH_3_); 56.8 (CH_2_); 56.0 (2CH_3_); 53.6 (2CH_2_); 48.3 (CH_2_) ppm. **M.p.:** 128 °C. **HRMS:** C_25_H_30_N_2_O_6_S. Calculated: 487.1897 (M + H^+^); found: 487.1884 (M + H^+^).

Synthesis of ***N-*(cyanomethyl)*-N-*(6-methoxypyridin-3-yl)naphthalene-2-sulfonamide (20)**.

Following general procedure D, *N-*(6-methoxypyridin-3-yl)naphthalene-2-sulfonamide (**18**) (98 mg, 0.313 mmol) was treated with 2-chloroacetonitrile (0.031 mg, 0.407 mmol) and Cs_2_CO_3_ (152 mg, 0.469 mmol) and stirred for 4 days to give sulfonamide **20**. Yield, crude: 87.7% (97 mg); flash chromatography with Hexane/EtOAc (1:1): 58.0% (192 mg).

**^1^H-NMR (CDCl_3_):** 8.29 (1H, *s*); 7.93–8.00 (4H, *m*); 7.61–7.71 (3H, *m*); 7.39 (1H, *dd*, *J* = 2.4 Hz; 8.4 Hz); 6.70 (1H, *d*, *J* = 8.8 Hz); 4.62 (3H, *s*); 3.92 (2H, *s*). **^13^C-NMR (CD_3_OD):** 164.0 (C); 147.3 (CH); 139.1 (CH); 135.2 (C); 134.3 (C); 132.0 (C); 129.5 (CH); 129.4 (CH); 129.2 (CH); 129.1 (C); 128.9 (CH); 127.7 (CH); 127.6 (CH); 122.2 (CH); 115.4 (C); 111.1 (CH); 53.2 (CH_3_); 39.3 (CH_2_) ppm. **HRMS:** C_18_H_16_N_3_O_3_S. Calculated: 354.0907 (M + H^+^); found: 354.0898 (M + H^+^).

Synthesis of ***N-*(2-chloropyridin-4-yl)*-N-*(cyanomethyl)naphthalene-2-sulfonamide (29)**.

Following general procedure D, *N-*(2-chloropyridin-4-yl)naphthalene-2-sulfonamide (**26**) (136 mg, 0.427 mmol) was treated with 2-chloroacetonitrile (42 mg, 0.555 mmol) and Cs_2_CO_3_ (208 mg, 0.640 mmol) and stirred for 5 days to give sulfonamide **29**. Yield, crude: 91.0% (139 mg); crystals (mixture of Hexane and EtOAc): 25.5% (39 mg).

**^1^H-NMR (CDCl_3_):** 8.40 (1H, *d*, *J* = 1.6 Hz); 8.34 (1H, *d*, *J* = 5.6 Hz); 7.97 (2H, *d*, *J* = 8.8 Hz); 7.92 (1H, *d*, *J* = 7.6 Hz); 7.63–7.73 (3H, *m*); 7.37 (1H, *d*, *J* = 2.0 Hz); 7.22 (1H, *dd*, *J* = 1.6 Hz; 5.6 Hz); 4.72 (2H, *s*) ppm. **M.p.:** 152 °C. **HRMS:** C_17_H_12_ClN_3_O_2_S. Calculated: 380.0231 (M + H^+^); found: 380.0225 (M + H^+^).

Synthesis of **3-(*N-*(3,4-dichlorophenyl)naphthalene-2-sulfonamido)propanamide (34)**.

Following general procedure D, *N-*(3,4-dichlorophenyl)naphthalene-2-sulfonamide (**32**) (177 mg, 0.503 mmol) was treated with 3-chloropropanamide (181 mg, 1.005 mmol) and Cs_2_CO_3_ (213 mg, 0.654 mmol) and stirred for 6 days to give sulfonamide **8**. Yield, crude: 98.6% (210 mg); crystals (MeOH): 46.5% (99 mg).

**^1^H-NMR (CDCl_3_):** 8.38 (1H, *s*); 7.78–7.95 (3H, *m*); 7.72 (1H, *dd*, *J* = 1.6 Hz; 8.4 Hz); 7.60–7.67 (2H, *m*); 7.28 (1H, *s*); 6.95 (1H, *dd*, *J* = 2.0; 9.2 Hz); 6.70 (1H, *s*); 3.81 (2H, *t*, *J* = 6.4 Hz); 2.70 (2H, *t*, *J* = 6.4 Hz). **^13^C-NMR (CDCl_3_):** 172.8 (C); 136.7 (C); 135.6 (C); 135.0 (C); 133.0 (C); 132.0 (C); 130.8 (CH); 129.7 (CH); 129.3 (CH); 129.1 (CH); 128.9 (CH); 128.4 (C); 127.9 (CH); 127.7 (CH); 122.4 (CH); 121.9 (CH); 120.0 (CH); 39.7 (CH_2_); 38.7 (CH_2_) ppm. **M.p.:** 179 °C. **HRMS:** C_19_H_16_Cl_2_N_2_O_3_S. Calculated: 445.0151 (M + Na^+^); found: 445.0149 (M + Na^+^).

Synthesis of ***N-*(3,4-dichlorophenyl)-*N-*(2-morpholinoethyl)naphthalene-2-sulfonamide (35)**.

Following general procedure D, *N-*(3,4-dichlorophenyl)naphthalene-2-sulfonamide (**32**) (198 mg, 0.562 mmol) was treated with 4-(2-chloroethyl)morpholine (170 mg, 1.124 mmol) and Cs_2_CO_3_ (238 mg, 0.731 mmol) and stirred for 6 days to give sulfonamide **35**. Yield, crude: quantitative (262 mg); crystals (MeOH): 34.9% (91 mg).

**^1^H-NMR (CDCl_3_):** 8.22 (1H, *d*, *J* = 2.0 Hz); 7.91–7.95 (3H, *m*); 7.62–7.69 (2H, *m*); 7.58 (1H, *dd*, *J* = 2.0 Hz; 8.8 Hz); 7.35 (1H, *d*, *J* = 8.8 Hz); 7.26–7.27 (1H, *m*); 6.93 (1H, *dd*, *J* = 2.4 Hz; 8.4 Hz); 3.68 (2H, *t*, *J* = 6.8 Hz); 3.61 (4H, *t*, *J* = 4.8 Hz); 2.45 (2H, *t*, *J* = 6.8 Hz); 2.38 (4H, *t*, *J* = 4.8 Hz). **^13^C-NMR (DMSOd_6_):** 139.6 (C); 135.2 (C); 134.9 (C); 132.2 (C); 131.5 (CH); 131.4 (CH); 131.1 (C); 131.0 (CH); 129.9 (CH); 129.6 (CH); 129.4 (CH); 129.6 (CH); 128.3 (CH); 128.2 (CH); 123.0 (CH); 66.5 (2CH_2_); 56.8 (CH_2_); 53.3 (2CH_2_); 47.6 (CH_2_) ppm. 1C not found. **M.p.:** 183 °C. **HRMS:** C_22_H_22_Cl_2_N_2_O_3_S. Calculated: 465.0801 (M + H^+^); found: 465.0797 (M + H^+^).

- General synthetic procedure (E) for benzylation.

To a solution of the sulfonamide (1.0 eq) dissolved in DMF at room temperature, (bromomethyl)benzene (2.0 eq) and K_2_CO_3_ (2.7 eq) were added, with continuous stirring. The reaction was monitored by TLC until completion. The crude reaction mixture was then poured onto ice, diluted with a large volume of CH_2_Cl_2_, and washed with brine until a neutral pH was reached. The resulting solution was dried over anhydrous sodium sulfate, filtered, and vacuum concentrated.

Synthesis of ***N-*benzyl*-N-*(3,4,5-trimethoxyphenyl)naphthalene-2-sulfonamide (4)**.

Following general procedure E, *N-*(3,4,5-trimethoxyphenyl)naphthalene-2-sulfonamide (**1**) (201 mg, 0.538 mmol) was treated with (bromomethyl)benzene (130 mg, 1.077 mmol) and K_2_CO_3_ (200 mg, 1.449 mmol) and stirred for 20 h to give sulfonamide **4**. Yield, crude: 71.0% (177 mg); crystals (mixture of Hexane and CH_2_Cl_2_): 30.5% (76 mg).

**^1^H-NMR (CDCl_3_):** 8.30 (1H, *s*); 7.97 (1H, *d*, *J* = 8.0 Hz); 7.94 (2H, *d*, *J* = 7.6 Hz); 7.73 (1H, *dd*, *J* = 2.0 Hz; 8.8 Hz); 7.61–7.68 (2H, *m*); 7.21–7.25 (5H, *m*); 6.07 (2H, *s*); 3.78 (3H, *s*); 3.50 (6H, *s*) ppm. **^13^C-NMR (CDCl_3_):** 152.9 (2C); 137.8 (C); 136.0 (C); 135.7 (C); 134.8 (C); 134.4 (C); 132.1 (C); 129.2 (2CH); 129.0 (CH); 128.9 (CH); 128.9 (CH); 128.8 (CH); 128.4 (CH); 127.9 (2CH); 127.8 (CH); 127.7 (CH); 123.1 (CH); 106.8 (2CH); 60.8 (CH_3_); 55.9 (2CH_3_); 55.5 (CH_2_) ppm. **M.p.:** 186 °C. **HRMS:** C_26_H_25_NO_5_S. Calculated: 486.1346 (M + Na^+^); found: 486.1341 (M + Na^+^).

Synthesis of ***N-*benzyl-*N-*(3,5-dimethoxyphenyl)naphthalene-2-sulfonamide (13)**.

Following general procedure E, *N-*(3,5-dimethoxyphenyl)naphthalene-2-sulfonamide (**11**) (122 mg, 0.355 mmol) was treated with (bromomethyl)benzene (86 mg, 0.711 mmol) and K_2_CO_3_ (132 mg, 0.959 mmol) and stirred for 20 h to give sulfonamide **13**. Yield, crude: quantitative (154 mg); crystals (mixture of Hexane and CH_2_Cl_2_): 80.6% (124 mg).

**^1^H-NMR (CDCl_3_):** 8.32 (1H, *d*, *J* = 1.2 Hz); 7.91–7.96 (3H, *m*); 7.58–7.71 (3H, *m*); 7.20–7.28 (5H, *m*); 6.26 (2H, *t*, *J* = 2.2 Hz); 6.16 (1H, *d*, *J* = 2.2 Hz); 4.75 (2H, *s*); 3.56 (6H, *s*) ppm. **^13^C-NMR (CDCl_3_):** 160.5 (2C); 140.7 (C); 136.0 (C); 135.7 (C); 134.8 (C); 132.1 (C); 129.3 (CH); 129.1 (CH); 129.1 (CH); 128.8 (CH); 128.5 (2CH); 128.4 (2CH); 127.9 (CH); 127.7 (CH); 127.5 (CH); 123.1 (CH); 107.3 (2CH); 100.3 (CH); 55.3 (2CH_3_); 55.1 (CH_2_) ppm. **M.p.:** 159 °C. **HRMS:** C_25_H_23_NO_4_S. Calculated: 434.1421 (M + H^+^); found: 434.1417 (M + H^+^).

- General synthetic procedure (F) for saponification.

To a solution of the sulfonamide (1.0 eq) dissolved in MeOH at room temperature, KOH (6 eq) was added, with continuous stirring. The reaction was monitored by TLC until completion. The crude reaction mixture was then poured onto ice, diluted with a large volume of CH_2_Cl_2_, and washed with brine until a neutral pH was reached. The resulting solution was dried over anhydrous sodium sulfate, filtered, and vacuum concentrated.

Synthesis of ***N-*(naphthalen-2-ylsulfonyl)-*N-*(3,4,5-trimethoxyphenyl)glycine (5)**.

Following general procedure F, ethyl *N-*(naphthalen-2-ylsulfonyl*)-N*-(3,4,5-trimethoxyphenyl)glycinate (**6**) (201 mg, 0.438 mmol) was stirred with KOH (147 mg, 2.628 mmol) for 24 h to give sulfonamide **5**. Yield, crude: 66.2% (125 mg); crystals (Acetone): 21.7% (41 mg).

**^1^H-NMR (CDCl_3_):** 8.28 (1H, *s*); 7.91–7.96 (3H, *m*); 7.73 (1H, *d*, *J* = 8.4 Hz); 7.59–7.68 (2H, *m*); 6.38 (2H, *s*); 4.47 (2H, *s*); 3.81 (3H, *s*); 3.68 (6H, *s*). **^13^C-NMR (DMSOd):** 170.6 (C); 153.0 (2C); 137.6 (C); 136.2 (C); 135.7 (C); 134.9 (C); 132.1 (C); 129.7 (CH); 129.6 (CH); 129.5 (CH); 129.2 (CH); 128.2 (CH); 128.1 (CH); 123.3 (CH); 106.8 (2CH); 60.5 (CH_3_); 56.2 (2CH_3_); 53.0 (CH_2_) ppm. **M.p.:** 252 °C. **HRMS:** C_21_H_21_NO_7_S. Calculated: 430.0966 (M-H^+^); found: 430.0965 (M-H^+^).

- Synthetic procedure (G) for aminolysis of esters.

To a solution of the sulfonamide (1.0 eq) dissolved in MeOH at room temperature, an excess of NH_3_ (10 eq) was added, with continuous stirring. The reaction was controlled by TLC until completion. The crude reaction mixture was then poured onto ice, diluted with a large volume of CH_2_Cl_2_, and washed with brine until a neutral pH was reached. The resulting solution was dried over anhydrous sodium sulfate, filtered, and vacuum concentrated.

Synthesis of ***N-*(2-oxopropyl)-*N-*(3,4,5-trimethoxyphenyl)naphthalene-2-sulfonamide (7)**.

Following general procedure G, ethyl *N-*(naphthalen-2-ylsulfonyl*)-N*-(3,4,5-trimethoxyphenyl)glycinate (**6**) (102 mg, 222 mmol) reacted with an excess of NH_3_ for 5 days to give **7**. Yield, crude: 90.1% (86 mg); crystals (MeOH): 29.3% (28 mg).

**^1^H-NMR (CDCl_3_):** 8.27 (1H, *d*, *J* = 2.0 Hz); 7.95–7.90 (3H, *m*); 7.75 (1H, *dd*, *J* = 2.0 Hz; 8.4 Hz); 7.67–7.58 (2H, *m*); 6.40 (2H, *s*); 4.43 (2H, *s*); 3.80 (3H, *s*); 3.71 (3H, *s*); 3.60 (6H, *s*). **^13^C-NMR (CDCl_3_):** 169.4 (C); 153.2 (2C); 138.2 (C); 135.8 (C); 135.1 (C); 134.9 (C); 131.9 (C); 129.4 (CH); 129.2 (CH); 129.0 (CH); 128.9 (CH); 127.8 (CH); 127.6 (CH); 123.2 (CH); 106.7 (2CH); 60.8 (CH_3_); 56.0 (2CH_3_); 53.4 (CH_2_); 52.4 (CH_3_) ppm. **M.p.:** 214 °C. **HRMS:** C_22_H_23_NO_7_S. Calculated: 468.1087 (M + Na^+^); found: 468.1084 (M + Na^+^).

- General synthetic procedure (H) for amide coupling of *N*-sulfonyl glycine derivatives.

To a solution of the sulfonamide (1.0 eq) dissolved in CH_2_Cl_2_ at room temperature, ethylamine (1.1 eq), DMAP (4 eq), and *N-*Ethyl*-N′-*(3-dimethylaminopropyl)carbodiimide hydrochloride (EDC) (8 eq) were added and allowed to heat until 55 °C, with continuous stirring. The reaction was controlled by TLC until completion. Then it was poured into ice and washed twice with 2 M HCl, twice with a 5% NaHCO_3_ solution, and finally with brine until a neutral pH was reached. The resulting solution was dried over anhydrous sodium sulfate, filtered, and vacuum concentrated.

Synthesis of ***N-*ethyl-2-(*N-*(3,4,5-trimethoxyphenyl)naphthalene-2-sulfonamido)acetamide (8)**.

Following general procedure H, *N-*(naphthalen-2-ylsulfonyl)-*N-*(3,4,5-trimethoxyphenyl)glycine (**5**) (91 mg, 0.210 mmol), ethylamine (79 mg, 0.230 mmol), DMAP (103 mg, 0.839 mmol), and EDC (322 mg, 1.680 mmol) were stirred for 5 h to give sulfonamide **8**. Yield, crude: 50.9% (49 mg); crystals (mixture of Hexane and EtOAc): 19.7% (19 mg).

**^1^H-NMR (CDCl_3_):** 8.20 (1H, *s*); 7.95–7.90 (3H, *m*); 7.70–7.63 (2H, *m*); 7.59 (1H, *dd*, *J* = 2.0 Hz; 8.4 Hz); 6.22 (2H, *s*); 4.17 (2H, *s*); 3.81 (3H, *s*); 3.59 (6H, *s*); 3.33 (2H, *c*, *J* = 7.2 Hz); 1.15 (3H, *t*, *J* = 7.2 Hz). **^13^C-NMR (CDCl_3_):** 167.5 (C); 153.5 (2C); 135.3 (C); 135.2 (C); 133.6 (C); 132.1 (C); 129.9 (CH); 129.5 (CH); 129.4 (CH); 129.3 (CH); 128.1 (CH); 123.2 (CH); 105.7 (2CH); 61.1 (CH_2_); 56.3 (2CH_3_); 55.4 (CH_3_); 34.7 (CH_2_); 15.0 (CH_3_) ppm. 1C not found. **M.p.:** 159 °C. **HRMS:** C_23_H_26_N_2_O_6_S. Calculated: 481.1404 (M + Na^+^); found: 481.1396 (M + Na^+^).

- General synthetic procedure (I) for *N-*alkylation of the sulfonamide using KOH.

To a solution of the sulfonamide (1.0 eq) dissolved in CH_3_CN at room temperature, 1-(2-chloroethyl)pyrrolidine (2 eq) and KOH (3.3 eq) were added, with continuous stirring. The reaction was controlled by TLC until completion. The crude reaction mixture was then poured onto ice, diluted with a large volume of CH_2_Cl_2_, and washed with brine until a neutral pH was reached. The resulting solution was dried over anhydrous sodium sulfate, filtered, and vacuum concentrated.

Synthesis of ***N-*(2-(pyrrolidin-1-yl)ethyl)*-N-*(3,4,5-trimethoxyphenyl)naphthalene-2-sulfonamide (9)**.

Following general procedure I, *N-*(3,4,5-trimethoxyphenyl)naphthalene-2-sulfonamide (**1**) (200 mg, 0.536 mmol) was stirred with 1-(2-chloroethyl)pyrrolidine (143 mg, 1.071 mmol) and KOH (100 mg, 1.774 mmol) for 3 days to give sulfonamide **9**. Yield, crude: 66.6% (168 mg); crystals (CH_2_Cl_2_): 16.3% (41 mg).

**^1^H-NMR (CDCl_3_):** 8.23 (1H, *s*); 7.90–7.94 (3H, *m*); 7.59–7.68 (3H, *m*); 6.23 (2H, *s*); 3.84 (3H, *s*); 3.73 (2H, *bs*); 3.59 (6H, *s*); 2.68 (2H, *bs*); 2.58 (4H, *bs*); 1.79 (4H, *bs*) ppm. **^13^C-NMR (CDCl_3_):** 153.0 (2C); 137.9 (C); 135.1 (C); 134.7 (C); 134.7 (C); 131.9 (C); 129.1 (CH); 129.1 (CH); 128.8 (CH); 128.8 (CH); 127.8 (CH); 127.6 (CH); 123.2 (CH); 106.5 (2CH); 60.8 (CH_3_); 55.9 (2CH_3_); 54.4 (CH_2_); 54.1 (2CH_2_); 50.3 (CH_2_); 23.5 (2CH_2_) ppm. **M.p.:** 117 °C. **HRMS:** C_26_H_25_NO_5_S. Calculated: 471.1948 (M + H^+^); found: 471.1950 (M + H^+^).

- General synthetic procedure (J) for bromination.

To a solution of the sulfonamide (1.0 eq) dissolved in CH_2_Cl_2_ at room temperature, *N-*Bromosuccinimide (1.2 eq) was added, with continuous stirring. The reaction was controlled by TLC until completion. The crude reaction mixture was then poured onto ice, diluted with a large volume of CH_2_Cl_2_, and washed with brine until a neutral pH was reached. The resulting solution was dried over anhydrous sodium sulfate, filtered, and vacuum concentrated.

Synthesis of ***N-*(4-bromo-2,5-dimethoxyphenyl)naphthalene-2-sulfonamide (16)**.

Following general procedure J, *N-*(2,5-dimethoxyphenyl)naphthalene-2-sulfonamide (**14**) (164 mg, 0.478 mmol) was stirred with NBS (103 mg, 0.573 mmol) for 20 h to give sulfonamide **16**. Yield, crude: quantitative (201 mg); crystals (MeOH): 27.7% (56 mg).

**^1^H-NMR (CDCl_3_):** 8.34 (1H, *d*, *J* = 1.6 Hz); 7.85–7.90 (3H, *m*); 7.71 (1H, *dd*, *J* = 2.0 Hz; 8.4 Hz); 7.57–7.63 (2H, *m*); 7.28 (1H, *s*); 7.05 (NH, *s*); 6.86 (1H, *s*); 3.87 (3H, *s*); 3.51 (3H, *s*) ppm. **^13^C-NMR (CDCl_3_):** 150.3 (C); 143.9 (C); 135.7 (C); 134.9 (C); 131.9 (C); 129.2 (CH); 129.2 (CH); 129.0 (CH); 128.8 (CH); 127.9 (CH); 127.6 (CH); 125.7 (C); 122.2 (CH); 115.9 (CH); 106.6 (C); 106.1 (CH); 56.9 (CH_3_); 56.3 (CH_3_) ppm. **M.p.:** 174 °C. **HRMS:** C_18_H_16_BrNO_4_S. Calculated: 443.9876 (M + Na^+^); found: 443.9875 (M + Na^+^).

Synthesis of ***N-*(4-bromo-2,5-dimethoxyphenyl)-*N-*methylnaphthalene-2-sulfonamide (17)**.

Following general procedure J, *N-*(2,5-dimethoxyphenyl)-*N-*methylnaphthalene-2-sulfonamide (**15**) (120 mg, 0.336 mmol) was stirred with NBS (73 mg, 0.403 mmol) for 20 h to give sulfonamide **17**. Yield, crude: 86.6% (127 mg); crystals (mixture of Hexane and CH_2_Cl_2_): 22.5% (33 mg).

**^1^H-NMR (CDCl_3_):** 8.25 (1H, *d*, *J* = 1.6 Hz); 7.90–7.93 (3H, *m*); 7.58–7.70 (3H, *m*); 6.96 (1H, *s*); 6.93 (1H, *s*); 3.83 (3H, *s*); 3.25 (3H, *s*); 3.08 (3H, *s*) ppm. **^13^C-NMR (CDCl_3_):** 150.5 (C); 149.8 (C); 136.2 (C); 134.7 (C); 132.0 (C); 129.2 (CH); 128.6 (CH); 128.5 (CH); 128.5 (C); 128.5 (CH); 127.8 (CH); 127.3 (CH); 123.3 (CH); 116.6 (CH); 116.0 (CH); 111.7 (C); 56.8 (CH_3_); 55.3 (CH_3_); 37.9 (CH_3_) ppm. **M.p.:** 138 °C. **HRMS:** C_19_H_18_BrNO_4_S. Calculated: 458.0032 (M + Na^+^); found: 458.0025 (M + Na^+^).

- General synthetic procedure (K) for mesylation.

To a solution of the amine (1.0 eq) dissolved in CH_2_Cl_2_ at room temperature, MeSO_2_Cl (1.15 eq) and 0.25 mL of pyridine were added, with continuous stirring. The reaction was controlled by TLC until completion. The crude reaction mixture was then poured onto ice, diluted with a large volume of CH_2_Cl_2_, and then washed twice with 2M HCl, twice with 5% NAHCO_3_, and finally with brine until a neutral pH was reached. The resulting solution was dried over anhydrous sodium sulfate, filtered, and vacuum concentrated.

Synthesis of ***N-*(2-methoxy-4-nitrophenyl)methanesulfonamide (P1)**.

Following the general procedure K, 2-methoxy-4-nitroaniline (1000 mg, 5.945 mmol) was treated with MeSO_2_Cl (783 mg, 6.837 mmol) and stirred for 3 days to give precursor **P1**. Yield, crude: 85.7% (1254 mg); crystals (mixture of Hexane and EtOAc): 43.5% (637 mg).

**^1^H-NMR (DMSOd):** 9.58 (NH, *bs*); 7.87 (1H, *dd*, *J* = 2.4 Hz; 9.2 Hz); 7.80 (1H, *d*, *J* = 2.4 Hz); 7.55 (1H, *d*, *J* = 9.2 Hz); 3.94 (3H, *s*); 3.14 (3H, *s*) ppm. **^13^C-NMR (CDCl_3_):** 147.8 (C); 143.8 (C); 132.8 (C); 117.6 (CH); 116.4 (CH); 106.1 (CH); 56.6 (CH_3_); 40.2 (CH_3_) ppm. **M.p.:** 131 °C.

- General synthetic procedure (L) for reduction of nitro groups using FeCl_3_ and Zn.

To a solution of the nitro compound (1.0 eq) dissolved in a mixture of H_2_O/acetone at room temperature, Zn (10 eq), FeCl_3_ (4 eq), and a few drops of HCl were added, with continuous stirring. The reaction was controlled by TLC, and after completion, the reaction crude was filtered over Celite^®^ (Fisher Scientific, Hampton, NH, USA), abundant CH_2_Cl_2_ was added, and it was washed with brine until neutral pH was reached. The resulting solution was dried over anhydrous sodium sulfate, filtered, and vacuum concentrated.

Synthesis of ***N-*(4-amino-2-methoxyphenyl)methanesulfonamide (P3)**.

Following general procedure L, *N-*(2-methoxy-4-nitrophenyl)methanesulfonamide (**P1**) (62 mg, 0.252 mmol) was reduced using Zn (160 mg, 2.518 mmol) and FeCl_3_ (160 mg, 0.986 mmol) for 4 days to give precursor **P3**. Yield, crude: 93.6% (51 mg); no further purification.

**^1^H-NMR (DMSOd):** 8.36 (NH, *bs*); 6.81 (1H, *d*, *J* = 8.4 Hz); 6.25 (1H, *d*, *J* = 2.0 Hz); 6.08 (1H, *dd*, *J* = 2.4 Hz; 8.8 Hz); 5.16 (NH_2_, *bs*); 3.97 (3H, *s*); 2.77 (3H, *s*) ppm. **^13^C-NMR (DMSOd):** 155.6 (C); 149.6 (C); 130.5 (CH); 113.5 (C); 106.0 (CH); 98.0 (CH); 55.6 (CH_3_); 40.1 (CH_3_) ppm. **HRMS:** C_8_H_12_N_2_O_3_S. Calculated: 217.0641 (M + H^+^); found: 217.0638 (M + H^+^).

Synthesis of ***N-*(4-amino-2-methoxyphenyl)*-N-*methylmethanesulfonamide (P4)**.

Following general procedure L, *N-*(2-methoxy-4-nitrophenyl)*-N-*methylmethanesulfonamide (**P2**) (270 mg, 1.037 mmol) was reduced using Zn (670 mg, 10.373 mmol) and FeCl_3_ (670 mg, 4.149 mmol) for 5 days to give precursor **P4**. Yield, crude: 79.6% (190 mg); no further purification.

**^1^H-NMR (CDCl_3_):** 7.12 (1H, *d*, *J* = 8.8 Hz); 6.24 (1H, *dd*, *J* = 1.2 Hz; 8.0 Hz); 6.23 (1H, *s*); 3.82 (3H, *s*); 3.67 (NH_2_, *bs*); 3.21 (3H, *s*); 2.89 (3H, *s*) ppm. **^13^C-NMR (CDCl_3_):** 156.8 (C); 148.2 (C); 133.1 (CH); 119.5 (C); 107.2 (CH); 98.7 (CH); 55.2 (CH_3_); 37.9 (CH_3_); 37.7 (CH_3_) ppm.

### 2.2. Biology

#### 2.2.1. Cell Culture Conditions

The cell lines were from ATTC (Manassas, VA, USA). HeLa (cervix epithelioid carcinoma), A172 (glioblastoma), U-87 MG (glioblastoma), U-118 MG (glioblastoma), MCF7 (breast adenocarcinoma), HEK293 (human embryonic kidney cells), HUH-7 (hepatocellular carcinoma), and A549 (lung carcinoma) were grown in Dulbecco’s modified Eagle’s medium (DMEM) (Gibco, Waltham, MA, USA) supplemented with 10% (*v*/*v*) heat-inactivated fetal bovine serum (HIFBS, Sigma-Aldrich, St. Louis, MO, USA), 2 mM L-glutamine, 100 μg/mL streptomycin, and 100 U/mL penicillin (Gibco, Waltham, MA, USA). HT-29 (colon carcinoma) cell line was grown in Roswell Park Memorial Institute 1640 (RPMI 1640) (Gibco, Waltham, MA, USA) supplemented with 10% HIFBS, 100 μg/mL streptomycin, and 100 U/mL penicillin. The cells were maintained at 37 °C, 95% humidity, and 5% CO_2_.

#### 2.2.2. Cell Growth Inhibition Assay

The effect of the compounds on the proliferation of tumoral cell lines was measured at 72 h using the MTT reagent (Sigma-Aldrich, St. Louis, MO, USA), following the manufacturer’s guidelines.

The cells were seeded in 96-well plates (100 µL/well) in complete DMEM or RPMI 1640 medium, depending on the line (see above), at 37 °C, 95% humidity, and 5% CO_2_ atmosphere and were allowed to adhere overnight. The concentrations used were: 40,000 cells/mL (MCF-7 cells), 30,000 cells/mL (HT-29, U-87 MG, U-118 MG, and HUH-7 cells), 20,000 cells/mL (A172, A549, and HeLa cells), and 15,000 cells/mL (HEK-293 cells). These concentrations were used as they achieve a 95% confluence at the end of the experiment in healthy controls. Compounds were then added (10 µL/well) at the desired final concentrations. Untreated cells and those treated with the maximum concentration of DMSO served as negative controls, with no differences observed between the two. Colchicine at 10 µM was used as a positive control. For compounds showing antiproliferative effects in the initial screening at 1 µm, their IC_50_ value was determined. The experiments were performed in three biological replicates, each one with three technical replicates. The IC_50_ values were determined using GraphPad Prism for Windows (GraphPad version 10.0.0 Software, Boston, MA, USA, www.graphpad.com).

To assess the sensitivity of compounds to MDR efflux pumps, we used the HT-29 cell line. The IC_50_ values were determined using the same procedure in both the presence and absence of verapamil (final concentration, 10 µM).

#### 2.2.3. Cell Cycle Analysis

HeLa (40,000 cells/mL), U-87 MG (50,000 cells/mL), and HT-29 (50,000 cells/mL) cells were seeded in 6-well plates (2 mL/well) and incubated in complete DMEM or RPMI 1640 medium (see above) at 37 °C and 5% CO_2_ atmosphere and were allowed to adhere overnight. Then the cells were treated with compounds **1** and **2** or colchicine at 600 nM, 100 nM, and 20 nM, respectively. As a negative control, cells were incubated in the presence of 0.1% DMSO (untreated cells). Cells were recovered at 24, 48, and 72 h following treatment and fixed overnight in ethanol/PBS (7:3) at 4 °C. The day before measurement, the collected cells were rinsed twice with PBS, suspended in a mixture of PBS, 0.1 mg/mL RNase A (Sigma-Aldrich, St. Louis, MO, USA), 40 µg/mL propidium iodide (Sigma-Aldrich, St. Louis, MO, USA), and 0.1% Triton, and then they were incubated overnight. A BD Accuri™ C6 Plus Flow Cytometer (BD Biosciences, Franklin Lakes, NJ, USA) cytometer was used to analyze the samples, and BD Accuri™ C6 Software (version 1.0.264.21) was used for data analysis.

#### 2.2.4. Apoptotic Cell Death Quantification

HeLa (40,000 cells/mL), U-87 MG (50,000 cells/mL), and HT-29 (50,000 cells/mL) cells were seeded in 12-well plates (1 mL/well) and incubated in complete DMEM or RPMI 1640 medium (see above) at 37 °C and 5% CO_2_ atmosphere and were allowed to adhere overnight. Then the cells were treated with compounds **1** and **2** or colchicine at 600 nM, 100 nM, and 20 nM, respectively. As a negative control, cells were incubated in the presence of 0.1% DMSO (Untreated cells). 72 h after treatment, cell populations were quantified using an Annexin V-FITC/PI apoptosis detection kit (Immunostep, Salamanca, Spain) according to the manufacturer’s instructions; briefly, cells were harvested, centrifugated, resuspended in the Annexin V binding buffer, and incubated in darkness with Annexin V-FITC/PI for 15 min at room temperature. The samples were analyzed the same day by using a BD Accuri™ C6 Plus Flow Cytometer (BD Biosciences), and the acquired data were analyzed via BD Accuri™ C6 Software (version 1.0.264.21).

#### 2.2.5. Confocal Microscopy

HeLa (40,000 cells) and U-87 MG (50,000 cells) cells were seeded onto poly-L-lysine–coated (0.01%) square glass coverslips (22 mm^2^), placed in 6-well plates (one per well), and allowed to adhere overnight in complete DMEM or RPMI 1640 medium (as indicated above) at 37 °C in a 5% CO_2_ atmosphere. The cells were incubated with compounds **1** and **2** at 600 nM and 100 nM, respectively. As a negative control, cells were incubated in the presence of 0.1% DMSO. After 24 h of drug treatment incubation, the medium was removed, coverslips were rinsed with PBS, fixed with 4% formaldehyde in PBS for 10 min, permeabilized with an ice-cold PBS solution with 0.25% Triton X-100 (Sigma-Aldrich, St. Louis, MO, USA) for 10 min, blocked with a solution of PBS with 10% BSA for 30 min, and rinsed once more with PBS. Samples were incubated for 2 h with a mouse monoclonal anti-α-tubulin antibody (Sigma-Aldrich, St. Louis, MO, USA) diluted 1:200 in PBS. After rinsing with PBS, they were further incubated for 1 h in the dark with Alexa Fluor 488 goat anti-mouse IgG secondary antibody (Molecular Probes, Invitrogen, Eugene, OR, USA) diluted 1:400 in PBS. The coverslips were then washed again with PBS, and mounted with a drop of ProLong™ Gold Antifade Mountant containing DAPI (ThermoFisher, Waltham, MA, USA) for nuclear staining. The samples were imaged using a LEICA SP5 microscope DMI-6000V model (Leica Microsystems, Wetzlar, Germany) coupled to a computer running LEICA LAS AF software.

### 2.3. In Silico Studies

#### 2.3.1. Conformational Analysis

The virtual structures of the synthesized naphthalene sulfonamides were built, and conformers were generated using the conformational search engine at the semiempirical (AM1) level. The obtained structures were energy minimized with RB3LYP DFT calculations at the 6-31G(D) level in vacuum and in water using the Spartan’08 software package (Wavefunction, Inc., Irvine, CA, USA, 2008).

#### 2.3.2. Docking Studies

The virtual compounds were docked into the colchicine site of tubulin by applying ensemble docking procedures, as previously described [13,18]. Briefly, the compounds were drawn and transformed into 3D structures in Marvin. The protein conformational space was accounted for using a set of different X-ray structures of tubulin complexes with structurally diverse colchicine-site ligands available in the PDB [24], which represent different site configurations due to adjustment to the ligand. A total of 139 downloaded structures were restricted to a single tubulin dimer, the ligands in the binding site were removed, and the proteins were cleaned to satisfy the requirements of the docking procedures. When waters bridging the ligands and the protein were present in the binding site, different models were generated with or without them. Six previously generated models from a molecular dynamics simulation were also included, giving a total of 153 binding sites for the docking runs. The refined models were aligned within Chimera to best superimpose the colchicine sites. We ran docking experiments in parallel with AutoDock 4.2 using the Lamarckian genetic algorithm (LGA) 100–300 times for a maximum of 2.5 million energy evaluations, 150 individuals, a maximum of 27,000 generations, and 10 runs per ligand with PLANTS 1.2 [25] in default settings. The outputs from the docking programs were semiautomatically processed using in-house scripts and KNIME pipelines [26]. In-house-generated KNIME pipelines were used to automatically allocate every docking pose in the colchicine-site subzones (A, B, C, and D). Z-scores were obtained from the programs’ scores to allow comparisons of the different scoring scales. The common docking pose for the two software sharing the best Z-scores was taken as the consensus prediction of the binding mode. RMSDs were calculated with LigRMSD v1.0 [27] between every docked pose and model scaffolds with no substituents and with colchicine-binding-site ligands representative of binders occupying different subzones. Analysis of the docked poses was done with Chimera [28], OpenEye [29], and JADOPPT [30].

#### 2.3.3. Calculation of Physicochemical Properties, Pharmacokinetic Parameters, and Metabolic Transformations

The structures of the synthesized molecules were drawn using ChemDraw 25.5.0.5789 (Revvity Signals Software, Waltham, MA, USA, 2024), and their SMILES codes were obtained. We used the SwissADME web tool [31] to calculate different physicochemical properties and pharmacokinetic parameters. Selected properties were further compared, such as the fraction of sp3 carbons, topological surface area (TPSA), different lipophilicity (logP), solubility estimates, GI absorption, sensitivity to glycoprotein efflux pumps (P-gp substrate), and Bioavailability Score. Additionally, we examined the sites of metabolic transformations using the SOMP and FAME3 web tools [32,33].

## 3. Results and Discussion

### 3.1. Chemical Synthesis

**Scheme 1:**
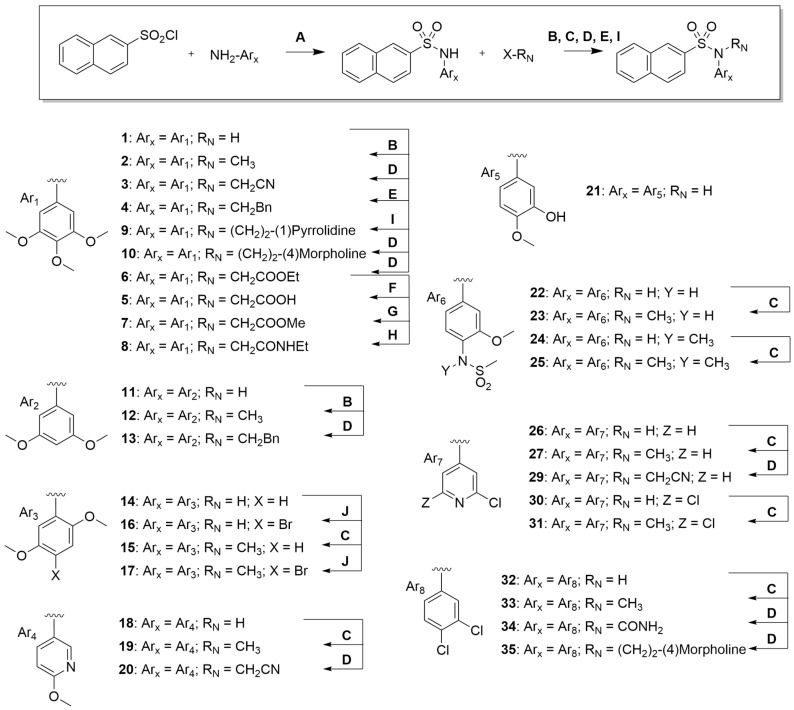
Synthesis of sulfonamides. Reagents and conditions: A: CH_2_Cl_2_, pyridine (yields 15 to 99%); B: Me_2_SO_4_, tetrabutylammonium bromide, NaOH, CH_2_Cl_2_/H_2_O (yields 76 to 97%); C: MeI, Cs_2_CO_3_, CH_3_CN (yields 51% to quantitative); D: halogenated derivative, Cs_2_CO_3_, CH_3_CN (yields 65% to quantitative); E: (bromomethyl)benzene, K_2_CO_3_, DMF (yields 71% to quantitative); F: KOH, MeOH (yield 66%); G: NH_3_, MeOH (yield 90%); H: Ethylamine, EDC, DMAP, CH_2_Cl_2_ (yield 51%); I: 1-(2-chloroethyl)pyrrolidine, KOH, CH_3_CN (yield 67%); J: NBS, CH_2_Cl_2_ (yields 87% to quantitative).

A total of 35 sulfonamides belonging to eight families according to the A rings and with different substituents on the sulfonamide nitrogen were synthesized (Scheme 1). Sulfonamides were formed by reacting naphthalene-2-sulfonyl chloride with the corresponding anilines or aminopyridines (Condition A, compounds **1**, **11**, **14**, **18**, **21**, **22**, **24**, **26**, **30**, and **32**) in good yields. However, solubility issues with the chloropyridamines reduced the yields for compounds **26** and **30**.

The synthetic route to the *N-*(4-methoxy-3-methanesulfonamidophenyl) family (Ar_6_) began with the anilines **P3** and **P4** (Scheme S1). Mesylation of 2-methoxy-4-nitroaniline gave sulfonamide **P1**, whose methylation under basic catalysis gave derivative **P2**. Reduction of nitro-compounds **P1** and **P2** to anilines **P3** and **P4**, respectively, was achieved with FeCl_3_/Zn.

The sulfonamide nitrogen was alkylated to explore the effects of *N*-substituents of different sizes, shapes, and properties on the SAR. Methylation was performed with dimethyl sulfate under phase-transfer conditions (Condition B, compounds **2** and **12**) or with MeI under basic (Cs_2_CO_3_) catalysis (Condition C, compounds **15**, **19**, **23**, **25**, **27**, **31**, and **33**). The reactions proceeded in good yields, most above 70%. Methylation of compound **26** gave the methylated sulfonamide methylpyridinium **28**. Other *N*-alkylations under basic (Cs_2_CO_3_) catalysis (Condition D, compounds **3**, **6**, **10**, **20**, **29**, **34**, and **35**) or KOH (Condition I, compound **9**) were also performed, along with benzylations (Condition E, Compounds **4** and **13**). Ethyl ester **6** was saponified with alcoholic KOH to carboxylic acid **5** (Condition F). Attempts to form amides from ethyl ester **6** using aqueous ammonia as the nucleophile in refluxing methanol resulted in the formation of methyl ester **7** (Condition G). Ethylamide **8** was obtained from acid **5** with ethylamine, DMAP, and EDC (Condition H). Compounds **16** and **17** were obtained from **14** and **15**, respectively, by bromination using *N-*bromosuccinimide (NBS) in excellent yields (Condition J).

### 3.2. Biology

#### 3.2.1. Naphthalene Sulfonamides Are Potent Antiproliferative Agents with Limited Tolerance for the Aniline Ring

The antiproliferative effect after 72 h of treatment with the naphthalene sulfonamides on a group of human tumorigenic cell lines was assessed using the MTT method. This panel included HeLa (cervix epithelioid carcinoma), U-118 MG and A172 (glioblastoma), and HT-29 (colon adenocarcinoma). For those compounds that at 1 μM inhibit cell proliferation in more than 40% of the untreated control in three independent experiments, the antiproliferative effects were measured at different concentrations to calculate the IC_50_ values (drug concentration that inhibits 50% of cell proliferation compared to the untreated control). Additional antiproliferative assays for treatments with compounds **1**, **2**, and **3** were performed on MCF7 (human breast adenocarcinoma), U-87 MG (human glioblastoma), HEK-293 (human embryonic kidney), HUH-7 (human hepatoma), and A549 (human lung adenocarcinoma) cells. CA-4, colchicine, and ABT-751 were used as positive controls representative of colchicine-site ligands, while paclitaxel was included as a different drug affecting the microtubules (Table 1 and Table S1).

Of all the proposed A-ring substitutions, only the 3,4,5-trimethoxyphenyl ring (TM, Ar_1_) and, although less potently, the 4-bromo-2,5-dimethoxyphenyl ring (4-Br-25DM, Ar_3_) achieve IC_50_ values in the submicromolar range. Compound **1**, the *N*-(3,4,5-trimethoxyphenyl) naphthalene sulfonamide scaffold, shows high antiproliferative potency against all tested cell lines, most of them between 171–430 nM. Compound **2**, featuring a methyl substituent at the sulfonamide nitrogen, achieves the best values in every cell line except for A172, where it is outperformed by compound **3**, which bears a cyanomethyl group at the sulfonamide nitrogen. Compound **2** reaches double-digit nanomolar range values in HeLa, U-118MG, and HEK-293. Compounds **1** and **3** display similar cytotoxicity values to each other and are comparable or superior to the reference sulfonamide ABT-751. Compounds **4** (with a benzyl group), **6** (with an ethoxycarbonylmethyl group), and **7** (with a methoxycarbonylmethyl group) also show good cytotoxicity values, particularly against the U-118 MG cell line. Compound **10**, with a positively charged 2-(4-morpholinyl)ethyl *N*-substituent, exhibits very good activity against the HeLa cells, achieving similar IC_50_ values to compound **3** and even surpassing **1**, but its activity drops off entirely in the HT-29, U-118 MG, and A172 lines, being the only compound to show this trend. Its activity against HeLa cells suggests that it can interact with tubulin, and the loss of activity against other cell lines might be due to differences in uptake. Compound **17**, with a 4-bromo-2,5-dimethoxyphenyl A ring (Ar_3_), is the only active compound without a TM ring. It does not show activity against HeLa cells, but achieves modest IC_50_ values against HT-29 and U-118 MG.

With respect to the substituent on the sulfonamide nitrogen, a methyl group appears to be the most favorable for activity, with compound **2** showing the highest potency across all the cell lines, except for A172, where its activity significantly drops to a similar IC_50_ value as that of **1**. The HT-29 cell line, a cell line with intrinsic resistance to Combretastatin A-4 and related compounds, shows the most significant sensitivity increase upon methylation, a 4-fold magnitude (104 nM for the methylated analog **2** versus 413 nM for the unsubstituted sulfonamide **1**). Methylation of the sulfonamide nitrogen in the 4-bromo-2,5-dimethoxyphenyl series (**17**) also affords the best results, as it is the only one to achieve submicromolar potency against HT-29 and U-118 MG cells, but not for HeLa. Increasing the size and the polarity of the substituent seems to be detrimental, except in the case of the morpholinylethyl substituted compound **10**, and exclusively against HeLa cells. The unsubstituted sulfonamide **1** shows similar IC_50_ values to the cyanomethyl derivative **3**, both worse than the methyl derivative 2, thus suggesting that the methyl binds to a small and mostly hydrophobic pocket that cannot accommodate as well polar or larger moieties. In good agreement, larger or more polar substituents such as the alkoxycarbonyls **6** and **7** or the benzyl derivative **4** result in lower potencies. In line with this, the 2-naphthyl sulfonamide **5d** reported by Wang et al. [34], bearing a 4-methoxybenzyl group on the nitrogen, showed considerably weaker activity (IC_50_ = 5.72 µM on MCF-7 and >30 µM on A549) than our submicromolar benzyl derivative **4**. This indicates that the interfacial space of tubulin is flexible and adaptable to medium-sized substituents.

In summary, the main structure–activity relationships (SAR) established from these data are: (I) the 3,4,5-trimethoxyphenyl A-ring is essential for submicromolar activity, with compound **17** being the sole active exception outside this series; (II) *N*-methylation is the most favorable sulfonamide nitrogen substitution, with compound **2** achieving double-digit nanomolar potency in multiple cell lines; and (III) larger or more polar *N*-substituents progressively reduce potency, with strongly ionizable or bulky groups being poorly tolerated, except for the morpholinylethyl derivative **10**, which retains HeLa-specific activity.

A head-to-head comparison of the potencies of the naphthalene sulfonamides with their paired 4-methoxyphenyl sulfonamides [20,21] (Table S2) reveals quite different responses depending on the aniline ring. For the 3,4,5-trimethoxyanilides (Ar_1_), both show similar potencies regardless of the substituent on the sulfonamide nitrogen, except in the HT-29 cells, which seem less resistant to the naphthalene sulfonamides. For the rest of the anilines (Ar_2_ to Ar_8_), the naphthalene sulfonamide is a worse pairing than the 4-methoxyphenyl sulfonamide. This is most evident for the 4-bromo-2,5-dimethoxyaniline family, which, in combination with the p-methoxyphenyl, results in potencies in the double-digit nanomolar range, whereas the combination with naphthalene fails to yield submicromolar potencies. This different behavior seems unlikely to arise from different uptakes upon A-ring changing, as such changes should similarly affect all series. Instead, it suggests that the differences arise from the sulfonamide binding to tubulin. We have previously shown that B-rings affect A-ring performance and vice versa, and that this coupling varies depending on the nature of the bridge joining the aromatic rings. This effect seems to arise from a more sterically demanding in-plane left upper quadrant (positions 7 and 8) of the naphthalene, which pushes the molecule toward the A-ring site walls and results in more stringent binding requirements for the anilines. This is a subtle effect, as the only slightly less sterically demanding indole [14] (Table S2) results in more potent compounds and a less stringent structure–activity relationship for the A rings. The higher potency of the indoles seems to arise not only from a better fit but also from electronic effects, as the dimethylaminophenyl analogs do not perform as well [35].

#### 3.2.2. Antiproliferative Naphthalene Sulfonamides Are Not Substrates of the MDR Pumps

The multidrug-resistant (MDR) phenotype is the most frequent clinical resistance mechanism against drugs that target microtubules [36]. This phenotype is due to the expression of MDR efflux pumps, which remove drugs from the cells, thus reducing their effectiveness [37,38,39]. To evaluate if every compound is a substrate of MDR efflux proteins, their potency against the HT-29 colorectal cell line was compared in the presence and absence of 10 µM verapamil, a P-GP 1 and MRP1 [40,41,42] inhibitor that had no antiproliferative effect at this concentration as a single treatment. If the drug is a substrate of the MDR proteins inhibited by verapamil, the cotreatment should decrease the IC_50_ value due to an increase in the effective intracellular drug concentration. The effect of the verapamil cotreatment was quantified as the relative sensitization (RS) of the cells upon cotreatment with verapamil, a percentual measure of the IC_50_ value change: RS (%) = ((IC_50_ cotreatment/IC_50_ without verapamil) × 100). The lower the percentage, the higher the effect of the cotreatment with verapamil and therefore the sensitivity of the compound to MDR pumps inhibited by verapamil. No active compound showed a significant (greater than 20%) decrease in the IC_50_ value with the verapamil cotreatment, and none of the compounds lacking submicromolar potency reached it upon cotreatment with verapamil (Table 2 and Table S1), thus indicating that they are not substrates of the MDR pumps and thus rendered inactive by efflux. Colchicine, a known MDR substrate, shows a greater than 40% decrease. These results suggest that this new family of compounds is not affected by MDR efflux.

#### 3.2.3. Naphthalene Sulfonamides Produce a Different Effect in the Cell Cycle than Colchicine

**Figure 2:**
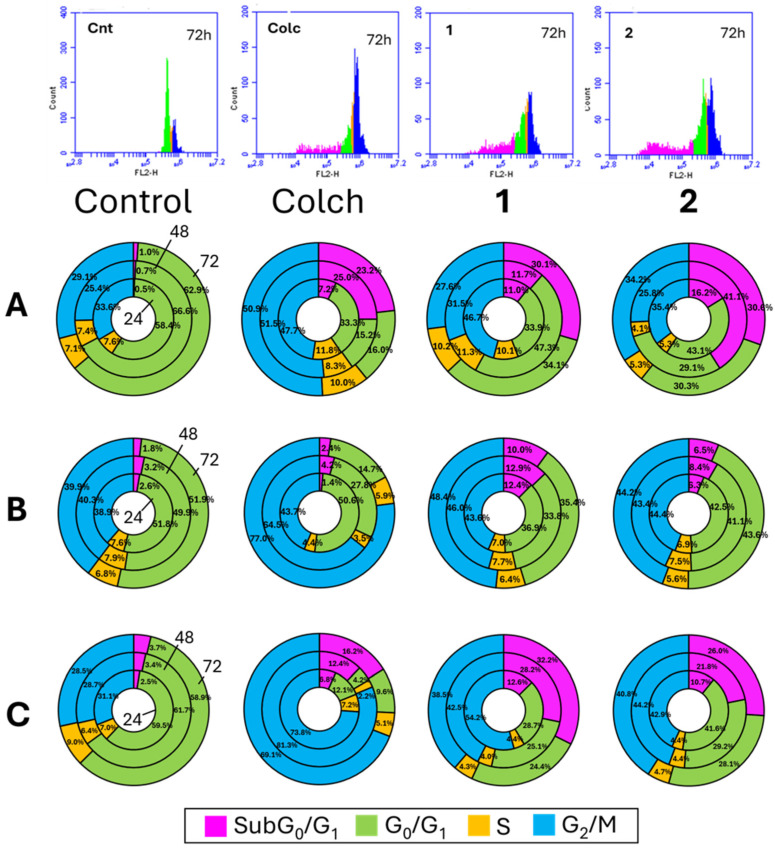
Cell-cycle profiles of HeLa (**A**), U-87 MG (**B**), and HT-29 (**C**) cells after 24, 48, or 72 h of treatment with colchicine (20 nM), compound **1** (600 nM), and **2** (100 nM). SubG_0_/G_1_, pink; G_0_/G_1_, green; S, yellow; and G_2_/M, blue. Untreated control cells were run in parallel.

To further evaluate the effect of the lead compounds **1** and **2**, we studied their effect on the cell-cycle populations at different time points employing flow cytometry. The effect was assessed in HeLa, U-87 MG, and HT-29 cell lines at 24 h, 48 h, and 72 h post-treatment, and colchicine served as a reference tubulin inhibitor (Figure 2 and Figures S1–S3, Table S3). The assay relies on the ability of propidium iodide (PI) to intercalate into DNA, enabling the differentiation of cell populations based on fluorescence intensity, and thus, differences in DNA content within the cells. Cells with a 2N DNA content are categorized as belonging to the G_0_/G_1_ phases, whereas those with 4N DNA are assigned to the G_2_/M phases. Cells with intermediate DNA amounts are placed in the S phase, and those with less than 2N DNA are classified into the SubG_0_/G_1_ population, indicative of apoptotic cells [43]. Compound **1** was tested at 600 nM while compound **2** was tested at 100 nM. Initial attempts to assay compound **1** at lower concentrations reported minimal or indistinguishable effects compared to the control.

The populations of HeLa cells in the untreated controls remain similar at the 24, 48, and 72 h: G_0_/G_1_ (58.4–66.6%), G_2_/M (25.4–33.6%), S (7.1–7.6%), and SubG_0_/G_1_ (<1%). Colchicine induces a sustained G_2_/M arrest (47.7–51.5–50.9%) and a progressive rise of the populations of cells at SubG_0_/G_1_ up to the 48 h time point (7.2–25.0–23.2%), both withdrawing cells from the G_0_/G_1_ populations (33.3–15.2–16.0%). Compounds **1** and **2** show a similar pattern of cell-cycle populations but with slightly different time evolution. Neither compound causes a mitotic arrest as pronounced as that observed for colchicine, although **1** induces a substantial increase in the G_2_/M population with respect to the control at 24 h (46.7%), which then decreases to control levels at 48 and 72 h (31.5% and 27.6%, respectively), similar to the three time points for **2** (35.4–25.8–34.2%). This different time course is also observed for the SubG_0_/G_1_ populations, which remain steady for the first two time points to finally increase (11.0–11.7–30.1%) for **1**, whereas for **2** it increases at 24 h, peaks at 48 h, and slightly decreases at 72 h (16.2–41.1–30.6%). These SubG_0_/G_1_ populations are larger in general than those observed for colchicine and are accompanied by varying reductions in the G_0_/G_1_ populations for **1** (33.9–47.3–34.1%) and **2** (35.4–25.8–34.2%) to account for a common total around 90% for the sum of the three populations. Compounds **1** and **2** do not induce the permanent G_2_/M arrest observed with colchicine but instead significantly increase the levels of apoptotic (SubG_0_/G_1_) cells.

The cell populations for U-87 MG are similar in the untreated control at the three time points: G_0_/G_1_ (49.9–51.9%), G_2_/M (38.9–40.3%), S (6.8–7.9%), and SubG_0_/G_1_ (1.8–3.2%). Compared with HeLa, there are substantially larger G_2_/M populations and complementary lower G_0_/G_1_ populations. In this cell line, colchicine progressively arrests cells at G_2_/M population (43.7–64.5–77.0%) but without substantially increasing the SubG_0_/G_1_ populations as observed for HeLa cells. For these cells, similar effects are achieved with **1** and **2**: faint sustained increases in the G_2_/M populations (43.6–46.0–48.4% and 44.4–43.4–44.2%, respectively) and steady significant SubG_0_/G_1_ increments (12.4–12.9–10.0% and 6.3–8.4–6.5%) when compared with the control, larger for **1** than for **2**. As observed in HeLa cells, **1** and **2** do not induce the prototypical strong mitotic arrests of colchicine, and their primary effect is to increase the SubG_0_/G_1_ population.

Similar populations’ ranges for the control HT-29 cells were obtained along the whole experiment: G_0_/G_1_ (58.9–61.7%), G_2_/M (28.5–31.1%), S (6.4–9.0%), and SubG_0_/G_1_ (2.5–3.7%), values like those of HeLa cells, but with slightly higher values of the SubG_0_/G_1_ populations. Colchicine quickly and sustainably arrests cells in the G_2_/M population (73.8–81.3–69.1%), accompanied by a progressive increase in the apoptotic SubG_0_/G_1_ populations (6.8–12.4–16.2%) and a drastic G_0_/G_1_ decrease (12.1–4.2–9.6%). The effects of **1** and **2** are similar, except for slight differences: **1** arrests more cells at G_2_/M after 24 h, to slightly decrease later on (54.2–42.5–38.5%), while the G_2_/M levels for **2** remain stable throughout the experiment (42.9–44.2–40.8%), in both cases showing lower G_2_/M arrests than cells treated with colchicine. The temporal increase in apoptotic cells is similar for both compounds, though higher for **1** (12.6–28.2–32.2%) than for **2** (10.7–21.8–26.0%), both sets significantly larger than those for colchicine. The effects of the compounds once again differ from those observed with colchicine, causing weak G_2_/M arrests but largely increasing the apoptotic SubG_0_/G_1_ populations instead.

Overall, colchicine shows different effects in the three cell lines, thus justifying their selection. In turn, **1** and **2** exhibit similar effects to each other and are different from colchicine, with **1** impacting more on U-87 MG and HT-29 cell populations and **2** on HeLa. Compared with colchicine, both compounds produce smaller increases in the G_2_/M population and markedly greater increases in the SubG_0_/G_1_ population, in both cases at the expense of the G_0_/G_1_ fraction. Although these differences could partly reflect the distinct concentrations used relative to each compound’s IC_50_, the consistency of this pattern across all three cell lines suggests an intrinsic mechanistic distinction rather than a purely concentration-dependent effect.

#### 3.2.4. Naphthalene Sulfonamides Induce Apoptotic Death 72 h Post-Treatment

**Figure 3:**
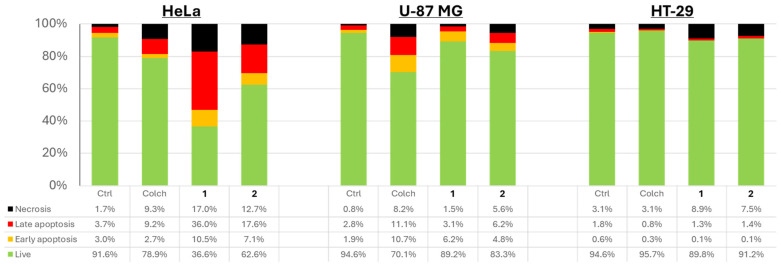
Annexin V-FITC/PI apoptosis profiles of HeLa, U-87 MG, and HT-29 cells after 72 h of treatment with colchicine (20 nM), compound **1** (600 nM), and compound **2** (100 nM). Untreated control cells were run in parallel.

Aiming to better understand the significant increase of cells in the SubG_0_/G_1_ populations induced by sulfonamides **1** and **2**, we studied the cell death induced in the same three cell lines used for cell cycle analysis after 72 h of treatment with **1** (600 nM) and **2** (100 nM) using bidimensional flow cytometry experiments by means of a double staining with fluorescein isothiocyanate-labeled Annexin V (AnV) and propidium iodide (PI) to elucidate the differences in the increments in the SubG_0_/G_1_ populations (Figure 3). Apoptotic cells translocate phosphatidylserine to the outer leaflet of the cell membrane and become Annexin V positive, and cells with damaged membranes allow PI uptake and become PI^+^. According to this dual labeling, cells were classified in four populations: healthy cells (PI^−^, AnV^−^), early apoptotic cells (PI^−^, AnV^+^), late apoptotic cells (PI^+^, AnV^+^), or necrotic cells (PI^+^, AnV^−^) [44].

Treatments of HeLa cells with **1** and **2** induce cell death substantially above the levels of the untreated control and even of those of colchicine-treated cells, particularly of the late apoptotic and necrotic ones. Compound **1** shows the most significant effects, reaching 36.0% of late apoptotic cells and a total of 46.4% of all apoptotic cells. Compound **2** has more modest effects, with 17.6% late apoptotic and 24.7% total apoptotic cells. The obtained results and the cell cycle results are in good qualitative agreement.

In U-87 MG cells, the effects of the compounds are more moderate but still significant compared to the untreated control: the percentage of healthy cells decreases with both compounds, accompanied by increases in other populations. In this case, **1** only shows a clear difference in the early apoptosis population, while **2** increases the two apoptotic and the necrotic populations. Colchicine exhibits a stronger effect than the compounds in this cell line, increasing all three affected populations to a similar extent. These effects do not correlate with the findings from cell cycle analysis, where colchicine almost does not change the SubG_0_/G_1_ populations compared to the control, and the naphthalene sulfonamides induce significant SubG_0_/G_1_ apoptotic populations.

For HT-29 cells, the populations of untreated and colchicine-treated cells are very similar. For the treatments with **1** and **2,** there is only a modest increase in the necrotic populations, in qualitative agreement with the results of the cell cycle analysis.

For the three cell lines, substantially different effects on the extent and stages of apoptotic induction were observed for each compound. The death induction results align with the cell cycle results for the HeLa and HT-29, but differ for U-87 MG cells. HeLa is the most sensitive cell line in this assay, followed by U-87 MG, and HT-29 shows the lowest sensitivity, in good agreement with its natural resistant phenotype to colchicine-site ligands. For all the treatments, the major populations are the late apoptotic or the necrotic (apoptotic cells often end up undergoing necrosis in cell cultures), which suggests an early onset of the apoptotic processes, as shown in the cell cycle assays.

#### 3.2.5. Naphthalene Sulfonamides Disrupt the Microtubule Network 24 h Post-Treatment

To further explore the effect of these compounds on the microtubule network, we performed immunostaining experiments. HeLa and U-87 MG cells treated with compound **1** (600 nM) and compound **2** (100 nM) for 24 h were stained with anti-alpha-tubulin antibodies and with DAPI for nuclei staining.

**Figure 4:**
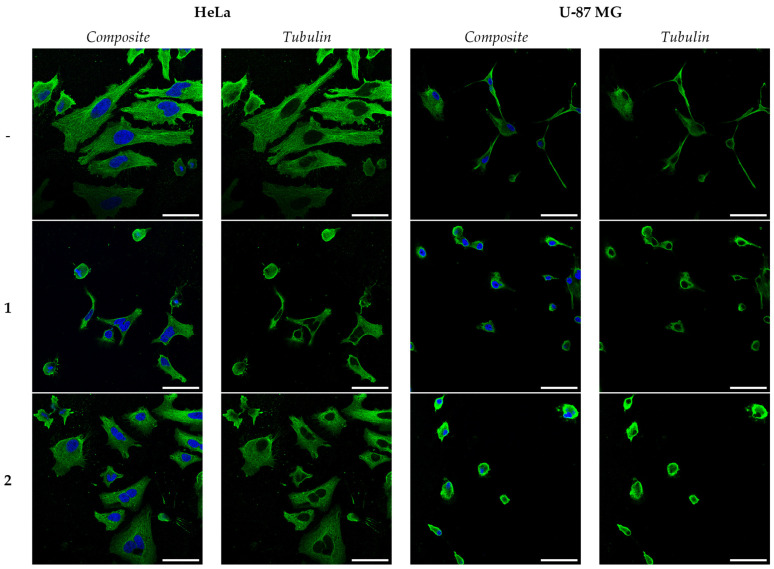
Confocal microscopy images of HeLa (**left**) and U-87 MG (**right**) showing the effect of the compounds **1** and **2** or 0.1% DMSO (−) after 24 h treatments. Nuclei were stained in blue with DAPI and α-Tubulin in green. The scale bar measures 50 µm.

The two compounds result in noticeable losses of microtubule network integrity in both cell lines. Treated HeLa cells lose their normal morphology and become rounded, with smaller cytoplasm and multilobed nuclei (Figure 4). The effects of **1** are more pronounced than those of **2**, in agreement with the cell cycle assays, where **1** showed the greatest effect at 24 h, while for **2**, they were greater later. In the case of U-87 MG cells, treatment with both compounds disrupts the triangular-star morphology, adopting a round shape with large accumulations of diffuse mass mainly clustered around the nuclei.

These findings suggest that these compounds effectively affect the microtubules inside the cell, consistent with their proposed mechanism of action as antimitotic agents.

### 3.3. In Silico Studies

#### 3.3.1. Conformational Analysis

*N*-(hetero)Aryl naphthalene sulfonamides are flexible compounds due to the freely rotating sulfonamide (S-N) bond and the 2-naphthyl-sulfur and (hetero)aryl-nitrogen single bonds. Their conformational preferences are a relevant factor that contributes to the potency, as binding to the different subpocket combinations of the colchicine site of tubulin occurs in different relative rings dispositions: binding to the AB rings requires a cisoid disposition of the sulfonamide bridge that places the two aryl rings in a close, non-coplanar arrangement whereas binding to the AC sub-pockets requires a more extended disposition of the aryl rings, as provided by transoid sulfonamide configurations. Considering that the limited docking scoring functions might not adequately account for such conformational issues, we performed DFT calculations of the conformational preferences of the ligands, quantified as the energy differences (Δ_relE_) between the transoid and cisoid conformations. The cisoid conformations are preferred over the transoid ones, thus favoring binding to the BA zones. The substitutions on the sulfonamide nitrogen increase, especially for the *N*-(3,4,5-trimethoxyphenyl) and *N*-(3,5-dimethoxyphenyl) derivatives, the energy difference being in favor of the cisoid conformations. Changes in the conformational preferences might affect the potencies of the ligands positively or negatively.

#### 3.3.2. Docking Experiments

**Figure 5:**
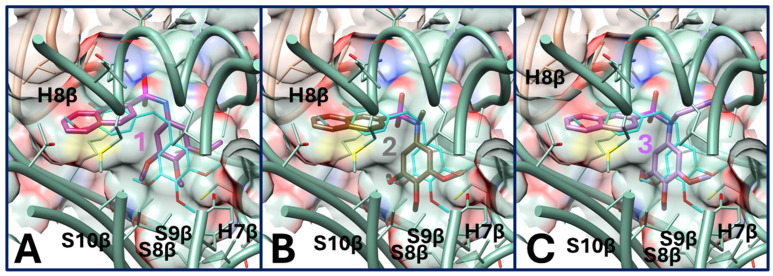
Docking poses for compounds **1** (**A**), **2** (**B**), and **3** (**C**) in complex with tubulin (PDB ID code 5GON). The tubulin secondary structural elements and interacting sidechains are shown. For comparison, combretastatin A-4 (CA-4) is shown with carbons in cyan.

Ensemble docking experiments [24] were used to study the binding modes and placement (Figure 5) of the compounds at the different sub-pockets (sub-sites A, B, and C) [7,9] of the colchicine site of tubulin. Colchicine-site ligands can be quite different in size and nature and bind to different zones [4,7,9]. To further increase the binding multiverse, similar moieties can bind to different sub-pockets depending on other ligand components or their geometrical arrangements. The flexibility of the colchicine site has been accounted for in the docking experiments by a group of experimental structures of tubulin in complexes with structurally dissimilar ligands that sample diverse protein dispositions and ligand pharmacophore configurations: 147 X-ray crystal structures [24] and 6 models from an earlier MD simulation [11] combined with flexible ligand docking [45].

Two docking programs were used in the docking experiments: AutoDock 4.2 [46] and PLANTS [25]. Their different scoring functions explore the sites differently, and the poses showing similar binding and the best combined scores were selected as the binding mode(s) for each docked ligand. Every pose was semiautomatically assigned to the different sub-pockets by: (1) measuring the shortest distance to the geometrical center of the subpocket, as defined by the pharmacophores derived from the X-ray structures [45]; (2) the RMSDs to model scaffolds putting phenyl rings on the subpocket centers; and (3) the RMSDs with combretastatin A-4 (AB), MI-181 (AC), or ABT-751 (ABC) in complex with tubulin (PDB IDs 5LYJ, 4YJ2 and 3HKC, respectively). The energy scores from each software were converted to relative scales between 0 (worst) and 1 (best) and to Z-scores, so that a fair comparison between them is possible for every pose. The pair of poses with the best combined Z-values for similarly binding poses from the two software packages was chosen as the consensus binding mode. Control experiments with ligands of known X-ray structures in complex with tubulin correctly retrieved the experimental poses [4].

The antiproliferative naphthalene sulfonamides occupy the BA sub-pockets of the colchicine site (Figure 5), with the naphthalene in the B zone and the 3,4,5-trimethoxyphenyl ring (TMP) in the A zone, arranged in a cisoid disposition by the sulfonamide bridge. In this series, the naphthalene runs with its long axis parallel to that of helix H8β and stacked behind Asn258β (H8β) and Val191α (H5α). Its H4, H5 edge makes hydrophobic contacts with Met259β (H8β), while H8 contacts the ε methylene of Lys352β (S9β). In addition, the aromatic plane forms hydrophobic interactions with Ala316β (S8β) and the β-methylene group of Lys352β (S9β). The TMP tight-fits in zone A, with its methoxylated edge taking up the hydrophobic cavity enclosed by the sidechains of Ala316β (S8), Ile318β (S9), Lys352β (S9β), Ala354β (S9β), and Ile378β (S10β) with Leu248β (Loop H7β-H8β) and Ala250β (H7β), and Leu248β (H8β) covering the sides of the ring plane and hydrogen bonding with the methoxy group to the thiol group of Cys241β (H7β). For differently substituted A rings, the docking poses require the displacement of the naphthalene moiety with respect to the methoxyphenyl ring of combretastatin A4, and in some instances (e.g., for the chloropyridines or the methylsulfonamido phenyls), the naphthalene occupies in a skewed disposition the A zone, due to a substantially worse fit of the other A rings to the A pocket, thus suggesting a worse fitting consistent with the loss of antiproliferative potency activity observed.

The ensemble docking approach selects the most favorable ligand poses along with the preferred binding sites. The PDB IDs most frequently retrieved by AD4 are 5JVD and 5LYJ, the complexes of tubulin with a chalcone analogue of combretastatin A-4 and combretastatin A-4 itself, while for PLANTS it is 5GON, the β-lactam analogue of combretastatin A-4. All these sites host ligands with a tightly fitting trimethoxyphenyl ring in the A zone and a methoxyphenyl ring in the B zone, thus confirming a fit to these adjusted sites and the formal equivalence of the naphthalene to the methoxyphenyl ring. We previously noted that the two software select differently, probably owing to the different scoring functions, thus validating the use of as many as possible protein models (ensemble docking strategy) and consensus scoring. The docking results support the design hypothesis that the active ligands target zones A-B for the naphthalene sulfonamides and that the naphthalene moieties impose severe restrictions on the structural variations tolerated in the A rings.

#### 3.3.3. Properties

The SwissADME web tool [31] was applied to calculate relevant physicochemical properties and pharmacokinetic parameters of the compounds and estimate their drug-likeness (Table S4 and Document S1). Compounds featuring the 3,4,5-trimethoxyphenyl ring exhibited intermediate values compared to the other A-rings tested. Compounds containing chloro groups tend to show decreases in Csp^3^, TPSA, and solubility, whereas compounds with methoxypyridines and (2-methoxy)benzene methanesulfonamide exhibited the highest solubilities. Within the trimethoxyphenyl family, none of the compounds are predicted to cross the blood-brain barrier; however, all are expected to have high gastrointestinal absorption and moderate solubility. Among them, compound **4**, with a benzyl substitution at the bridge, shows the lowest solubility, while those with a carboxylic chain substitution display the highest. Nevertheless, the effect of the substitution is relatively modest. The Csp^3^ fraction values range between 0.15 and 0.36, close to the desired value of 0.25. Additionally, all trimethoxyphenyl compounds exceed or approach a TPSA value of 75 Å^2^, which is associated with reduced toxicity issues [47], and are considered non-substrates of P-GP except for compound **4**. Although compound 4 showed a 15% reduction in IC_50_ value upon cotreatment with verapamil, it was classified as a non-substrate according to our criteria. Overall, these results suggest that the molecules are good candidates for antitumor treatment.

The potential sites of metabolic transformations were predicted using SOMP (to model the action of CYP3A4, CYP2D6, CYP2C19, CYP2C9, CYP1A2, and UGT) [32] and FAME3 (within NERDD) [33] (Figure S4 and Document S2). SOMP analysis identified two primary CYP metabolic liabilities: O-demethylation of methoxy groups (ΔP ≈ 0.55–0.70) and cleavage of substituent chains at the sulfonamide nitrogen, particularly methyl substitutions (ΔP ≈ 0.50–0.80). No significant differences were observed across CYP enzymes or methoxy positions, except for methoxypyridine and (2-methoxy)benzenesulfonamide derivatives, which consistently showed slightly lower values. FAME3 corroborated these findings, also identifying methoxy groups as the primary metabolic sites. However, it revealed greater variability among substitution patterns, assigning lower values to methoxyls in 3,5-dimethoxyphenyl derivatives and higher values to those in 2,5-dimethoxyphenyl analogs.

## 4. Conclusions

Colchicine-site ligands are promising antitumor drugs, but their toxicity, poor solubility, and instability remain unresolved issues, thus requiring the discovery of novel compounds. To address these challenges, we have explored the *N*-aryl 2-naphthalenesulfonamide scaffold in the search for new colchicine-site ligands. We synthesized and evaluated 35 compounds with different substitution patterns on the A ring and in the nitrogen of sulfonamide that have been shown to modulate the activity in related sulfonamides. Antiproliferative assays on a broad group of human cancer cell lines revealed that naphthalenesulfonamide serves as a promising scaffold, yielding compounds with activity in the double-digit nanomolar range. These results allowed us to establish structure–activity relationships: (1) the 3,4,5-trimethoxyphenyl ring appears essential for activity within this compound family; (2) substitution of the sulfonamide nitrogen with a methyl is the most favorable, followed by the cyanomethyl group, with larger substituents reducing the potency; (3) different nitrogen substitutions exhibit varying effects across cancer cell lines, with, for example, notable potency observed for cyanomethyl substitution in glioblastoma cell lines (U-118 MG and A172). Experiments using a P-glycoprotein inhibitor demonstrated that these compounds are not substrates of P-gp efflux pumps, a common cause of cancer cell resistance to taxanes and vinca alkaloids.

To study the mechanism of action, we examined the impact of the most potent compounds on the cell cycle at 24, 48, and 72 h. Unlike colchicine, which induces a strong and sustained G_2_/M arrest, compounds **1** and **2** produce only a modest increase in the G_2_/M population and instead markedly elevate the SubG_0_/G_1_ apoptotic fraction, indicating that these compounds trigger cell death through a distinct mechanistic pathway despite sharing the same binding site. The results from cell death mechanism assays using isothiocyanate-labeled Annexin V show that the compounds mostly induce late apoptotic responses and necrosis, a common endpoint of the apoptosis machinery in cultured cells.

Microscopy assays using immunostaining in HeLa and U-87 MG cells confirm the ability of the compounds to disrupt microtubule networks, thus reinforcing the proposed antitubulin mechanism of action through binding to the colchicine site. Ensemble docking experiments support binding at the colchicine site with the naphthalene system occupying the B zone and provide a rationale for the stringent requirement of a trimethoxyphenyl ring, thus expanding knowledge of the structural requirements governing sulfonamide binding to the colchicine site. The naphthalene sulfonamides are potent antimitotic agents with a distinct binding mode that results in cellular responses different from those induced by colchicine upon treatment of human tumor cells and therefore constitute a promising family for the further development of novel antitumor compounds.

## Figures and Tables

**Table 1:** IC_50_ values for antiproliferative activity against the tumorigenic cell lines HeLa, HT-29, U-118 MG, A172, U-87 MG, MCF7, HUH-7, A549, and the non-tumorigenic HEK-293. IC_50_ values are given in nM. Standard errors of the mean (SEM) are shown in Table S1. TM = 3,4,5-trimethoxyphenyl; DM = dimethoxyphenyl; Ms = Mesyl = methanesulfonyl = CH_3_SO_2_−; Pyrr = pyrrolidin-1-yl; Morph = morpholin-4-yl. Cpd = compound. The potency of the compounds is highlighted by green background colors whose darkness increase with potency.

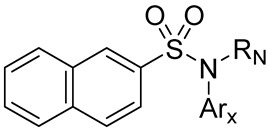												
Cpd	A Ring	Ar	R_N_	HeLa	HT-29	U118	A172	U87	MCF7	HEK	HUH-7	A549
**1**	TM	Ar_1_	H	333	413	186	434	512	305	171	798	595
**2**	TM	Ar_1_	CH_3_	67	104	93	431	132	102	74	222	159
**3**	TM	Ar_1_	CH_2_CN	171	268	195	231	269	206	154		
**4**	TM	Ar_1_	Bn	423	355	333	304					
**5**	TM	Ar_1_	CH_2_COOH	>1000	>1000	>1000	>1000					
**6**	TM	Ar_1_	CH_2_COOEt	307	335	167	630		324			
**7**	TM	Ar_1_	CH_2_COOMe	543	201	230	616					
**8**	TM	Ar_1_	CH_2_CONHEt	>1000		2650	>1000					
**9**	TM	Ar_1_	(CH_2_)_2_-(1)Pyrr	>1000	>1000	>1000						
**10**	TM	Ar_1_	(CH_2_)_2_-(4)Morph	168	>1000	1120	>1000					
**11**	3,5-DM	Ar_2_	H	>1000	>1000	>1000						
**12**	3,5-DM	Ar_2_	CH_3_	>1000	>1000	>1000						
**13**	3,5-DM	Ar_2_	Bn	>1000	>1000	>1000						
**14**	2,5-DM	Ar_3_	H	>1000	>1000	>1000						
**15**	2,5-DM	Ar_3_	CH_3_	>1000	>1000	>1000						
**16**	4-Br-2,5-DM	Ar_3_	H	>1000	>1000	>1000						
**17**	4-Br-2,5-DM	Ar_3_	CH_3_	>1000	718	520						
**18**	6-MeO-3-Pyridyl	Ar_4_	H	>1000	>1000	>1000	>1000					
**19**	6-MeO-3-Pyridyl	Ar_4_	CH_3_	>1000	>1000	>1000	>1000					
**20**	6-MeO-3-Pyridyl	Ar_4_	CH_2_CN	>1000	>1000	>1000	>1000					
**21**	3-OH-4-MeOPh	Ar_5_	H	1710	>1000	>1000	>1000					
**22**	3-MeO-4-MsNHPh	Ar_6_	H	>1000	>1000	>1000	>1000					
**23**	3-MeO-4-MsNHPh	Ar_6_	CH_3_	>1000	>1000	>1000	>1000					
**24**	3-MeO-4-MsNMePh	Ar_6_	H	>1000	>1000	>1000	>1000					
**25**	3-MeO-4-MsNMePh	Ar_6_	CH_3_	>1000	>1000	>1000	>1000					
**26**	2-Cl-4-Pyridyl	Ar_7_	H	>1000	>1000	>1000	>1000					
**27**	2-Cl-4-Pyridyl	Ar_7_	CH_3_	>1000	>1000	>1000	>1000					
**28**	2-Cl-*1*-Me-Pyridin- 1-ium-4-yl	Ar_7_	CH_3_	>1000	>1000	>1000	>1000					
**29**	2-Cl-4-Pyridyl	Ar_7_	CH_2_CN	>1000	>1000	>1000	>1000					
**30**	2,6-Cl_2_-4-Pyridyl	Ar_7_	H	>1000	>1000	>1000	>1000					
**31**	2,6-Cl_2_-4-Pyridyl	Ar_7_	CH_3_	>1000	>1000	>1000	>1000					
**32**	3,4-Cl_2_Ph	Ar_8_	H	>1000	>1000	>1000	>1000					
**33**	3,4-Cl_2_Ph	Ar_8_	CH_3_	>1000	>1000	>1000	>1000					
**34**	3,4-Cl_2_Ph	Ar_8_	CH_2_CONH_2_	>1000	>1000	>1000	>1000					
**35**	3,4-Cl_2_Ph	Ar_8_	(CH_2_)_2_-(4)Morph	>1000	>1000	>1000	>1000					
												
	Colchicine			5.5	9.1	8.9	20.9	11.9	15.2	8.0	39.5	39.6
	Paclitaxel			2.5	1.3	3.5	4.2	6.6	4.4	2.5	-	-
	ABT-751			315	1110	244	346	830	180	475	705	523
	CA-4			1.2	748	1.2	6.5	-	1.0	-	-	-

**Table 2:** IC_50_ values of antiproliferative activity in HT-29 cells co-treated with 10 μM verapamil (VPM) and without cotreatment. The IC_50_ values are given in nM. Standard errors of the mean are shown in Table S1. RS (%) = relative sensitization of the HT-29 cells upon cotreatment with verapamil.

Compound	HT-29	HT-29 + VPM	RS (%)
**1**	413	428	104
**2**	104	147	141
**3**	268	254	95
**4**	355	303	85
**5**	>1000	>1000	-
**6**	335	276	82
**7**	201	236	117
**9–35**	>1000	>1000	-
Colchicine	9.1	5.3	58

## Data Availability

The data underlying this study are available in the published article and its Supplementary Materials. Crude data are available from the authors upon request.

## Supplementary Materials

The following supporting information can be downloaded at: https://www.mdpi.com/article/10.3390/pharmaceutics18060733/s1, Scheme S1: Synthesis of aniline precursors P3 and P4. Reagents and conditions; Table S1: Antiproliferative activities; Table S2: A comparison of the antiproliferative potency (IC_50_) of naphthyl, 4-methoxyphenyl and 1-methyl-5-indolyl sulfonamides; Table S3: Values of the cell-cycle populations; Figure S1: Cell cycle histograms of HeLa cells; Figure S2: Cell cycle histograms of U-87 MG cells; Figure S3: Cell cycle histograms of HT-29 cells; Table S4: Selected physicochemical, pharmacokinetic, and properties calculated with SwissADME; Figure S4: Values obtained for the compounds 1, 2, and 3 from SOMP and FAME3 Software; Spectra: Spectra of the compounds 1–35 and P1–P4; Document S1: Results of SwissADME; Document S2: Results of SOMP and FAME3.

## Abbreviations

The following abbreviations are used in this manuscript:

CA-4	Combretastatin A-4
MDR	Multidrug resistance
DMAP	Dimethyl amino pyridine
EDC	*N-*Ethyl*-N′-*(3-dimethylaminopropyl)carbodiimide hydrochloride
NBS	*N-*bromosuccinimide
DMF	Dimethyl formamide
DMSO	Dimethyl sulfoxide
MTT	3-[4,5-dimethylthiazol-2-yl]-2,5-diphenyltetrazolium bromide
IC_50_	Inhibitory concentration 50
SEM	Standard errors of the mean
TM	3,4,5-trimethoxyphenyl
DM	Dimethoxyphenyl
Ms	Mesyl
Methanesulfonyl	CH_3_SO_2_−
Pyrr	Pyrrolidin-1-yl
Morph	Morpholin-4-yl
Cpd	Compound
P-gp	P-glycoprotein
RS	Relative sensitization
PI	Propidium iodide
AnV	Fluorescein isothiocyanate-labeled Annexin V
DAPI	4′,6-diamidino-2-phenylindole
PDB	Protein Data Bank
TPSA	Total polar surface area
TLC	Thin-layer chromatography
RPMI	Roswell Park Memorial Institute
DMEM	Dulbecco’s modified Eagle’s medium
HIFBS	Heat-inactivated fetal bovine serum
HRMS	High-resolution mass spectrometry
M.p.	Melting point
