# Supplementary material for: Antimitotic Naphthalene Sulfonamides Are Potent Antitumor Agents Acting Differently from Colchicine
Source: Pharmaceutics. 2026 Jun 13;18(6):733. doi: 10.3390/pharmaceutics18060733 (PMC13306225; doi:10.3390/pharmaceutics18060733)
Supplement: Supplementary file 1 [file pharmaceutics-18-00733-s001.zip › Pharmaceutics_MM Suppl_rev1.pdf]

Antimitotic Naphthalene Sulfonamides are potent antitumor agents acting differently from colchicine

Miguel Marín [1,2,3], Raúl Fuentes-Martín [1,2,3], Baldomero Sánchez [1,2,3], Laura Gallego-Yerga [1,2,3], Rafael Peláez [1,2,3,*]

Scheme S1: Synthesis of aniline precursors **P3** and **P4**. Reagents and conditions

Table S1: Antiproliferative activities

Table S2: A comparison of the antiproliferative potency (IC_{50}) of naphthyl, 4-methoxyphenyl and 1-methyl-5-indolyl sulfonamides

Table S3: Values of the cell cycle populations

Figure S1: Cell cycle histograms of HeLa cells

Figure S2: Cell cycle histograms of U-87 MG cells

Figure S3: Cell cycle histograms of HT-29 cells

Table S4: Selected physicochemical, pharmacokinetic, and properties calculated with SwissADME

Figure S4: Values obtained for the compounds **1**, **2**, and **3** from SOMP and FAME3 Software

Spectra: Spectra of the compounds **1-35** and **P1-P4**

Document 1: Results of SwissADME

Document 2: Results of SOMP and FAME3

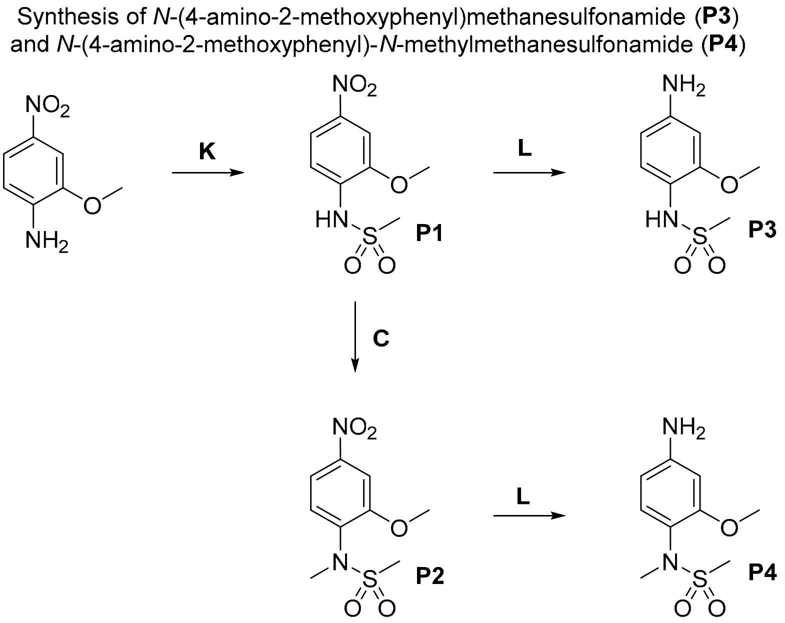

Scheme S1: Synthesis of aniline precursors P3 and P4. Reagents and conditions: **C**: MeI, Cs_2CO_3, CH_3CN; (yield 81%); **K**: $MeSO_2Cl$, CH_2Cl_2, pyr; (yield 86%); **L**: $FeCl_3$ and Zn, H_2O/Acetone, HCl; (yields 80 to 94%).

Table S1: Antiproliferative activity (IC_{50}) and the Standard error of the mean (SEM) in the different cell lines used in this work. IC_{50} values are in nM. N.d.: not determined

Compound	HeLa	HT-29	HT-29 + verap	U118	A172	U87	MCF7	HEK	HUH-7	A549
**1**	333 ± 31	413 ± 59	428 ± 68	186 ± 75	434 ± 155	512 ± 92	305 ± 76	171 ± 16	798 ± 177	595 ± 99
**2**	67 ± 27	104 ± 21	147 ± 37	93 ± 29	431 ± 170	132 ± 52	102 ± 27	74 ± 5	222 ± n.d.	159 ± 35
**3**	171 ± 56	268 ± 48	254 ± 35	195 ± 51	231 ± 31	269 ± 51	206 ± 21	154 ± 12		
**4**	423 ± 88	355 ± 63	303 ± 59	333 ± 179	304 ± 97					
**5**	>1000	>1000	>1000	>1000	>1000					
**6**	307	335 ± 22	276 ± 9	167±46	630 ± 144		324 ± 160			
**7**	543	201 ± 31	236 ± 24	230±93	616 ± 121					
**8**	>1000			2650 ± n.d.	>1000					
**9**	>1000	>1000	>1000	>1000						
**10**	168	>1000	>1000	1120 ± n.d.	>1000					
**11**	>1000	>1000	>1000	>1000						
**12**	>1000	>1000	>1000	>1000						
**13**	>1000	>1000	>1000	>1000						
**14**	>1000	>1000	>1000	>1000						
**15**	>1000	>1000	>1000	>1000						
**16**	>1000	>1000	>1000	>1000						
**17**	>1000	718 ± 35	678 ± 35	520 ± n.d.						
**18**	>1000	>1000	>1000	>1000	>1000					
**19**	>1000	>1000	>1000	>1000	>1000					
**20**	>1000	>1000	>1000	>1000	>1000					
**21**	1710 ± n.d.	>1000	>1000	>1000	>1000					
**22**	>1000	>1000	>1000	>1000	>1000					
**23**	>1000	>1000	>1000	>1000	>1000					
**24**	>1000	>1000	>1000	>1000	>1000					
**25**	>1000	>1000	>1000	>1000	>1000					
**26**	>1000	>1000	>1000	>1000	>1000					
**27**	>1000	>1000	>1000	>1000	>1000					
**28**	>1000	>1000	>1000	>1000	>1000					
**29**	>1000	>1000	>1000	>1000	>1000					
**30**	>1000	>1000	>1000	>1000	>1000					
**31**	>1000	>1000	>1000	>1000	>1000					
**32**	>1000	>1000	>1000	>1000	>1000					
**33**	>1000	>1000	>1000	>1000	>1000					
**34**	>1000	>1000	>1000	>1000	>1000					
**35**	>1000	>1000	>1000	>1000	>1000					
Colchicine	5.5 ± 0.6	9.1 ± 1.1	5.3 ± 0.5	8.9 ± 3.9	20.9 ± 0.9	11.9 ± 3.4	15.2 ± 2.9	8.0 ± 0.5	39.5 ± 19.9	39.6 ± 10.7
Paclitaxel	2.5 ± 0.6	1.3 ± 1.2		3.5 ± 2.2	4.2 ± 1.1	6.6 ± 0.8	4.4 ± 1.0	2.5 ± 0.7	-	-
ABT-751	315 ± 22	1110 ± 971		244 ± 29	346 ± 96	830 ± 68	180 ± 32	475 ± 22	705 ± 530	523 ± 83
CA-4	1.2 ± 0.3	748 ± 598		1.2 ± 0.5	6.5 ± 0.5	-	1.0 ± 0.4	-	-	-

Table S2: A comparison of the antiproliferative potency (IC_{50}) of naphthyl (Naphth) (this work), 4-methoxyphenyl (MeOPh) [20, 21] and 1-methyl-5-indolyl (MeIND) [14] sulfonamides against HeLa, HT-29, and MCF7. IC_{50} values are in nM.

N			Ring	R_N	HeLa			HT-29			MCF7		
**Naphth**	**MeOPh [19, 20]**	**MeIND [14]**			**Naphth**	**MeOPh**	**MeIND**	**Naphth**	**MeOPh**	**MeIND**	**Naphth**	**MeOPh**	**MeIND**
**1**	**1a**	**4c**		H	173	240	10	413	897	51	305	375	
**2**	**2a**	**5c**		CH_3	90	71	2.4	104	143	4.3	102	127	
**3**	**6a**	**5c**	3,4,5-TM	CH_2CN	171	143	14	268	230	48	206	275	
**4**	**9a**			Bn	423	750		355	>1000			830	
**5**	**8a**	**8**		CH_2COOH	>1000	>1000	>1000	>1000	>1000	>1000		>1000	
**6**	**7a**	**7**		CH_2COOEt	307	217	28.8	335	237	53	324	335	
**11**	**1b**	**21**		H	>1000	>1000	>1000	>1000	>1000	>1000		>1000	
**12**	**2b**	**22**	3,5-DMP	CH_3	>1000	877	20	>1000	>1000	43		765	
**13**	**6b**	**26**		Bn	>1000	>1000	273	>1000	>1000	370		>1000	
**14**	**21b**	**35**	2,5-DMP	H	>1000	227	320	>1000	187	898		350	
**15**	**22b**	**36**		CH_3	>1000	177	169	>1000	250	613		153	
**16**	**23b**		4-Br-	H	>1000	45		>1000	72			25	
**17**	**24b**		-2,5-DMP	CH_3	>1000	33		718	123			19	
Colç					5.5			9.1			15.2		
Pacli					2.5			1.3			4.4		
ABT					315			1110			180		
CA-4					1.2			748			1.0		

Table S3: Values of the cell cycle populations (SubG_0/G_1, G_0/G_1, S, and G_2/M) after 24, 48, or 72 hours of treatment with colchicine (20 nM) or compounds **1** (600 nM) and **2** (100 nM) in HeLa, U-87 MG, and HT-29. Negative control cells were run in parallel.

Treatment	Time	HeLa				U-87 MG				HT-29			
		Sub G_0/G_1	G_0/G_1	S	G_2/M	Sub G_0/G_1	G_0/G_1	S	G_2/M	Sub G_0/G_1	G_0/G_1	S	G_2/M
Control	24h	0.5%	58.4%	7.6%	33.6%	2.6%	51.8%	7.6%	38.9%	2.5%	59.5%	7.0%	31.1%
	48h	0.7%	66.6%	7.4%	25.4%	3.2%	49.9%	7.9%	40.3%	3.4%	61.7%	6.4%	28.7%
	72h	1.0%	62.9%	7.1%	29.1%	1.8%	51.9%	6.8%	39.9%	3.7%	58.9%	9.0%	28.5%
Colchicine	24h	7.2%	33.3%	11.8%	47.7%	1.4%	50.6%	4.4%	43.7%	6.8%	12.1%	7.2%	73.8%
	48h	25.0%	15.2%	8.3%	51.5%	4.2%	27.8%	3.5%	64.5%	12.4%	4.2%	2.2%	81.3%
	72h	23.2%	16.0%	10.0%	50.9%	2.4%	14.7%	5.9%	77.0%	16.2%	9.6%	5.1%	69.1%
**1**	24h	11.0%	33.9%	10.1%	46.7%	12.4%	36.9%	7.0%	43.6%	12.6%	28.7%	4.4%	54.2%
	48h	11.7%	47.3%	11.3%	31.5%	12.9%	33.8%	7.7%	46.0%	28.2%	25.1%	4.0%	42.5%
	72h	30.1%	34.1%	10.2%	27.6%	10.0%	35.4%	6.4%	48.4%	32.2%	24.4%	4.3%	38.5%
**2**	24h	16.2%	43.1%	5.3%	35.4%	6.3%	42.5%	6.9%	44.4%	10.7%	41.6%	4.4%	42.9%
	48h	41.1%	29.1%	4.1%	25.8%	8.4%	41.1%	7.5%	43.4%	21.8%	29.2%	4.4%	44.2%
	72h	30.6%	30.3%	5.3%	34.2%	6.5%	43.6%	5.6%	44.2%	26.0%	28.1%	4.7%	40.8%

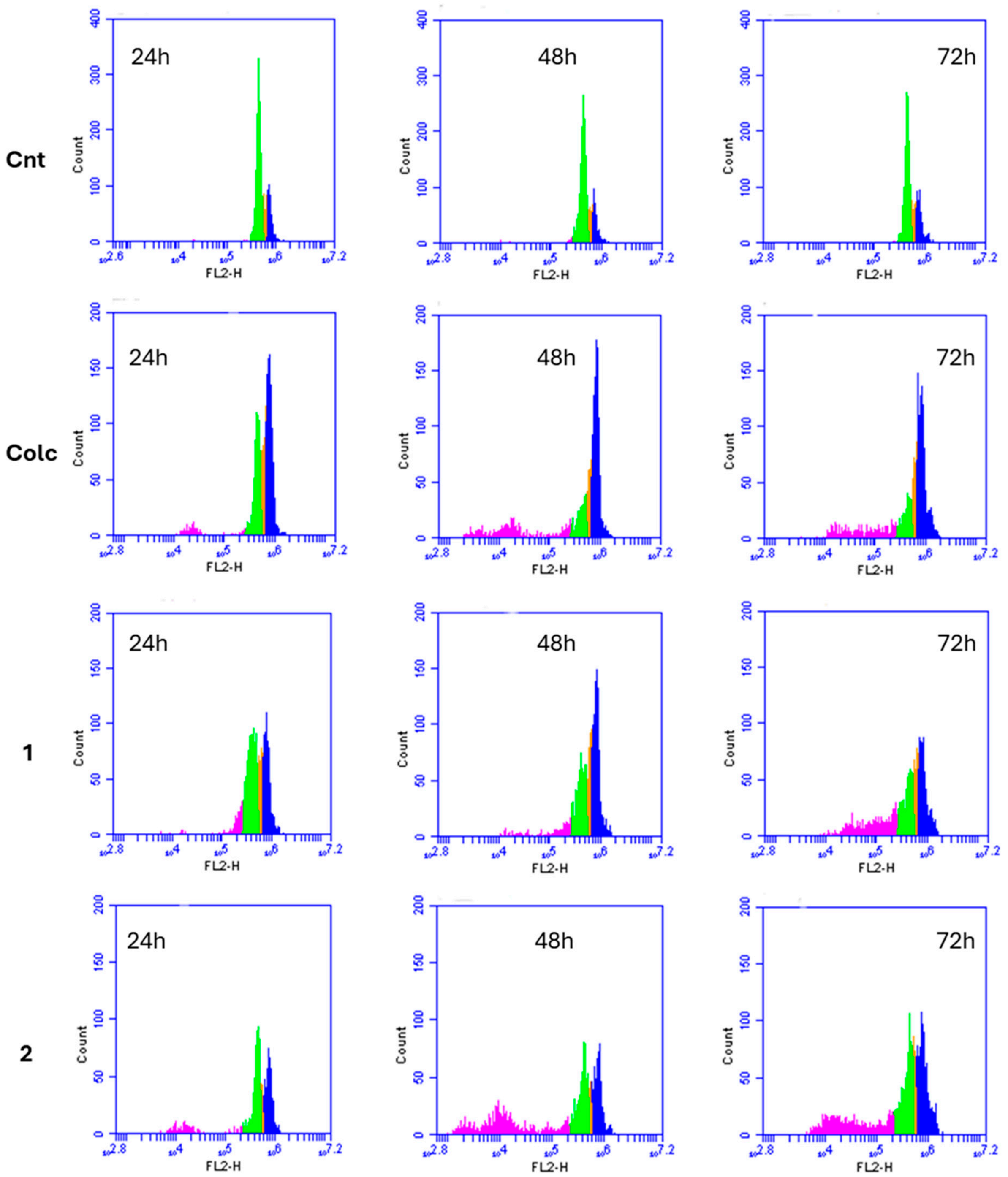

Figure S1: Cell cycle histograms of HeLa cells after 24, 48, or 72 hours of treatment with colchicine (20 nM) or compounds **1** (600 nM) and **2** (100 nM). Untreated control cells were run in parallel.

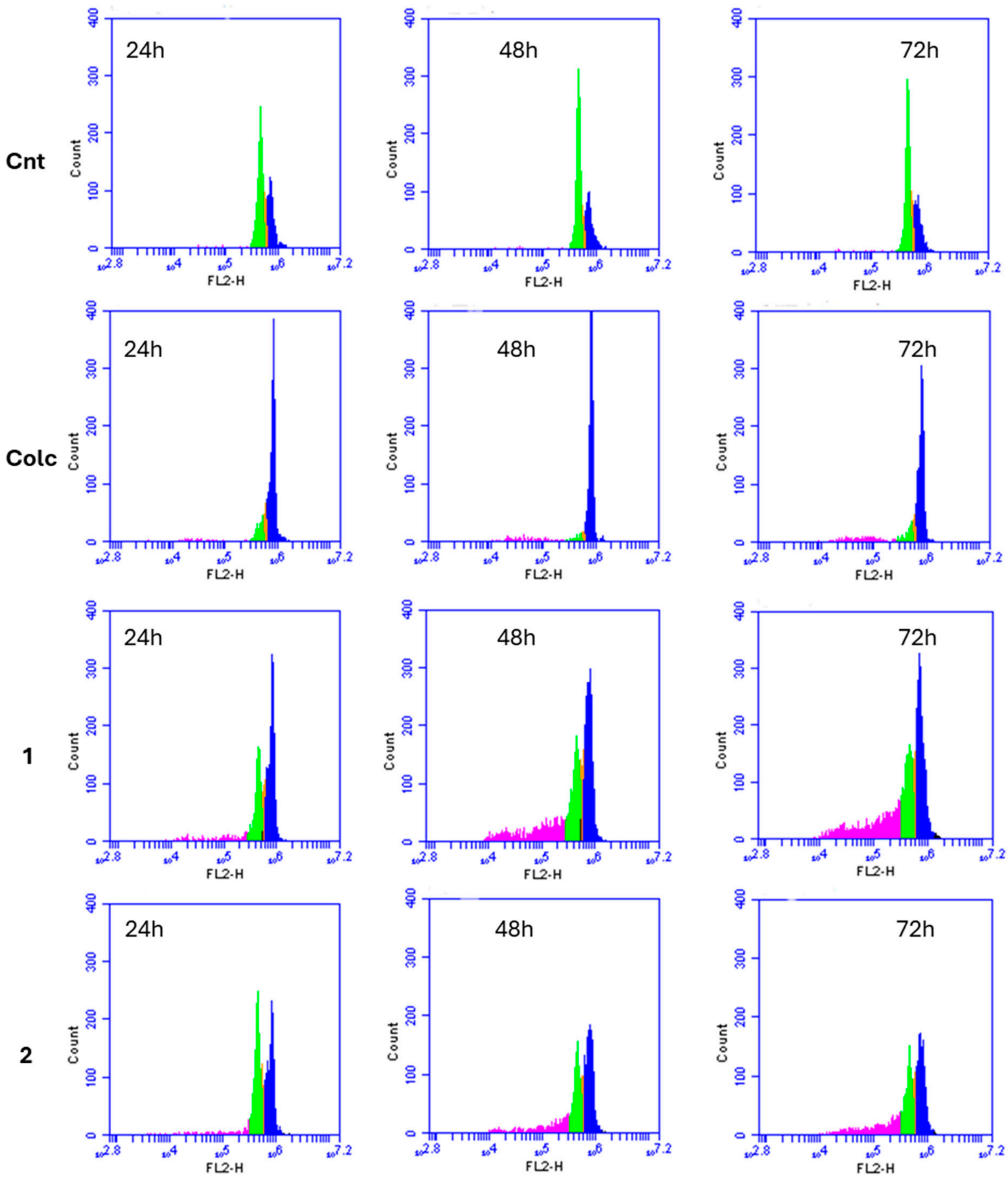

Figure S2: Cell cycle histograms of U-87 MG cells after 24, 48, or 72 hours of treatment with colchicine (20 nM) or compounds **1** (600 nM) and **2** (100 nM). Untreated control cells were run in parallel.

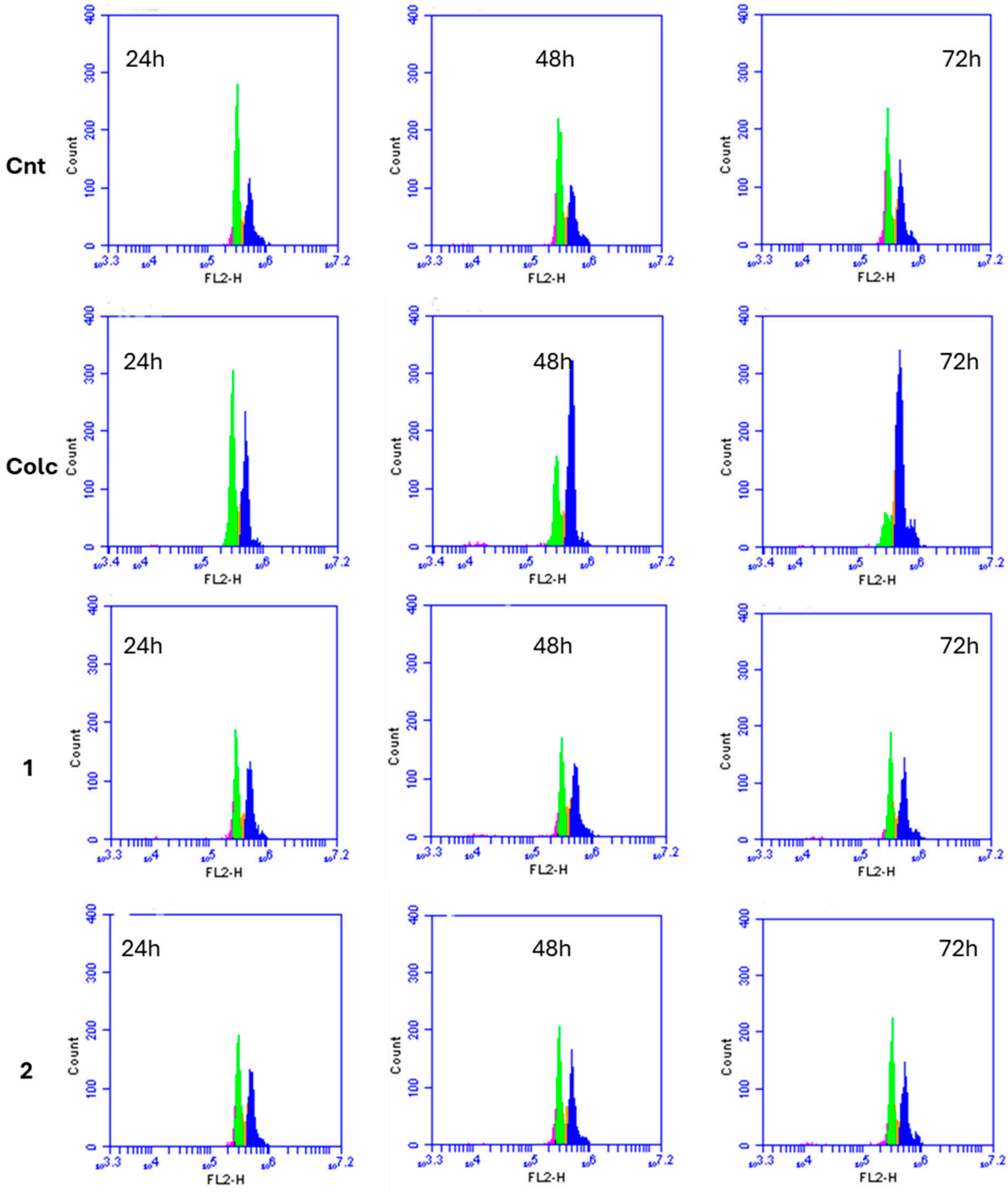

Figure S3: Cell cycle histograms of HT-29 cells after 24, 48, or 72 hours of treatment with colchicine (20 nM) or compounds **1** (600 nM) and **2** (100 nM). Untreated control cells were run in parallel.

Table S4: Selected physicochemical, pharmacokinetic, and properties calculated with SwissADME. The complete table can be found in Document S1.

Compound	Fraction Csp3	TPSA	iLOGP	Consensus Log P	ESOL Log S	ESOL Class	Ali Log S	Ali Class	Silicos-IT LogSw	Silicos-IT class	GI absorption	BBB permeant	Pgp substrate	Bioavailability Score
**1**	0,16	82,24	2,95	3,17	-4,49	Moderately soluble	-5,02	Moderately soluble	-6,89	Poorly soluble	High	No	No	0,55
**2**	0,2	73,45	3,13	3,32	-4,67	Moderately soluble	-5,03	Moderately soluble	-6,55	Poorly soluble	High	No	No	0,55
**3**	0,19	97,24	3,06	3,1	-4,6	Moderately soluble	-5,31	Moderately soluble	-6,64	Poorly soluble	High	No	No	0,55
**4**	0,15	73,45	3,57	4,5	-6,01	Poorly soluble	-6,58	Poorly soluble	-9,02	Poorly soluble	High	No	Yes	0,55
**5**	0,19	110,75	2,59	2,77	-4,52	Moderately soluble	-5,39	Moderately soluble	-5,91	Moderately soluble	High	No	No	0,56
**6**	0,26	99,75	3,43	3,45	-4,97	Moderately soluble	-5,88	Moderately soluble	-6,99	Poorly soluble	High	No	No	0,55
**7**	0,23	99,75	3,31	3,15	-4,73	Moderately soluble	-5,5	Moderately soluble	-6,6	Poorly soluble	High	No	No	0,55
**8**	0,26	102,55	3,58	3,14	-4,6	Moderately soluble	-5,34	Moderately soluble	-7,3	Poorly soluble	High	No	No	0,55
**9**	0,36	76,69	4,29	3,78	-5,24	Moderately soluble	-5,64	Moderately soluble	-7,34	Poorly soluble	High	No	No	0,55
**10**	0,36	85,92	4,22	3,2	-4,79	Moderately soluble	-4,95	Moderately soluble	-7,07	Poorly soluble	High	No	No	0,55
**11**	0,11	73,01	2,8	3,06	-4	Moderately soluble	-4,17	Moderately soluble	-6,79	Poorly soluble	High	No	No	0,55
**12**	0,16	64,22	3,17	3,39	-4,61	Moderately soluble	-4,86	Moderately soluble	-6,45	Poorly soluble	High	Yes	No	0,55
**13**	0,12	64,22	3,79	4,6	-5,94	Moderately soluble	-6,41	Poorly soluble	-8,92	Poorly soluble	High	No	Yes	0,55
**14**	0,11	73,01	3,14	3,23	-4,31	Moderately soluble	-4,67	Moderately soluble	-6,79	Poorly soluble	High	No	No	0,55
**15**	0,16	64,22	3,15	3,38	-4,61	Moderately soluble	-4,86	Moderately soluble	-6,45	Poorly soluble	High	Yes	No	0,55
**16**	0,11	73,01	3,39	3,87	-5,33	Moderately soluble	-5,58	Moderately soluble	-7,58	Poorly soluble	High	No	No	0,55
**17**	0,16	64,22	3,4	3,98	-5,51	Moderately soluble	-5,58	Moderately soluble	-7,24	Poorly soluble	High	No	No	0,55
**18**	0,06	76,67	2,22	2,55	-3,91	Soluble	-4,2	Moderately soluble	-6,3	Poorly soluble	High	Yes	No	0,55
**19**	0,12	67,88	2,82	2,78	-4,09	Moderately soluble	-4,2	Moderately soluble	-5,97	Moderately soluble	High	Yes	No	0,55
**20**	0,11	91,67	2,13	2,43	-4	Moderately soluble	-4,49	Moderately soluble	-6,06	Poorly soluble	High	No	No	0,55
**21**	0,06	84,01	1,89	2,74	-4,22	Moderately soluble	-4,75	Moderately soluble	-6,09	Poorly soluble	High	No	No	0,55
**22**	0,11	118,33	2,32	2,47	-4,06	Moderately soluble	-4,77	Moderately soluble	-6,97	Poorly soluble	Low	No	No	0,55
**23**	0,16	109,54	2,64	2,65	-4,25	Moderately soluble	-4,78	Moderately soluble	-6,63	Poorly soluble	High	No	Yes	0,55
**24**	0,16	109,54	2,59	2,64	-4,25	Moderately soluble	-4,78	Moderately soluble	-6,63	Poorly soluble	High	No	Yes	0,55
**25**	0,2	100,75	2,8	2,8	-4,44	Moderately soluble	-4,78	Moderately soluble	-6,29	Poorly soluble	High	No	Yes	0,55
**26**	0	67,44	1,99	2,97	-4,44	Moderately soluble	-4,69	Moderately soluble	-6,79	Poorly soluble	High	Yes	No	0,55
**27**	0,06	58,65	2,72	3,23	-4,62	Moderately soluble	-4,69	Moderately soluble	-6,45	Poorly soluble	High	Yes	No	0,55
**28**	0,12	49,64	-1,25	2,33	-4,77	Moderately soluble	-4,64	Moderately soluble	-6,01	Poorly soluble	High	Yes	Yes	0,55
**29**	0,06	82,44	2,2	2,91	-4,53	Moderately soluble	-4,98	Moderately soluble	-6,54	Poorly soluble	High	No	No	0,55
**30**	0	67,44	2,21	3,57	-5,24	Moderately soluble	-5,69	Moderately soluble	-7,38	Poorly soluble	High	No	No	0,55
**31**	0,06	58,65	2,96	3,83	-5,41	Moderately soluble	-5,69	Moderately soluble	-7,05	Poorly soluble	High	Yes	No	0,55
**32**	0	54,55	2,39	4,21	-5,48	Moderately soluble	-5,83	Moderately soluble	-7,76	Poorly soluble	High	No	No	0,55
**33**	0,06	45,76	3,18	4,48	-5,66	Moderately soluble	-5,84	Moderately soluble	-7,42	Poorly soluble	High	No	No	0,55
**34**	0,06	88,85	1,97	3,39	-5,07	Moderately soluble	-5,65	Moderately soluble	-6,99	Poorly soluble	High	No	No	0,55
**35**	0,27	58,23	4,08	4,3	-5,75	Moderately soluble	-5,75	Moderately soluble	-7,94	Poorly soluble	High	Yes	No	0,55

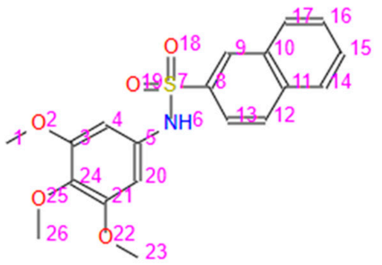

1	CYP3A4		CYP2D6		CYP2C19		CYP2C9		CYP1A2		UGT		Probability	SOM	Fame score
	Rang	Delta P	Rang	Delta P	Rang	Delta P	Rang	Delta P	Rang	Delta P	Rang	Delta P			
C.1	2	0.563	1	0.732	1	0.750	2	0.673	1	0.521	7	-0.235	0.36	No	0.72
O.2	24	-0.925	23	-0.928	19	-0.711	20	-0.886	23	-0.844	3	-0.100	0.00	No	0.64
N.6	26	-0.986	26	-0.991	26	-0.989	26	-0.952	26	-0.980	1	0.383	0.24	No	0.64
C.15	4	0.274	4	0.218	4	0.308	4	0.067	4	0.275	14	-0.485	0.08	No	0.60
O.22	25	-0.925	24	-0.928	20	-0.711	22	-0.886	24	-0.844	4	-0.100	0.00	No	0.64
C.23	3	0.563	2	0.732	2	0.750	3	0.673	2	0.521	8	-0.235	0.36	No	0.72
O.25	23	-0.905	21	-0.902	18	-0.649	24	-0.897	22	-0.803	2	-0.075	0.01	No	0.69
C.26	1	0.577	3	0.727	3	0.712	1	0.699	3	0.397	9	-0.248	0.44	Yes	0.73

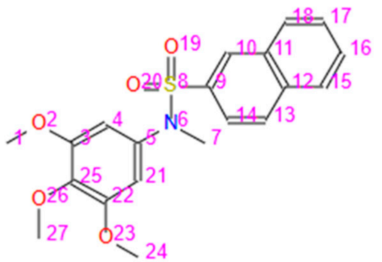

2															
	Rang	Delta P	Rang	Delta P	Rang	Delta P	Rang	Delta P	Rang	Delta P	Rang	Delta P			
C.1	3	0.605	1	0.753	1	0.748	3	0.609	1	0.503	9	-0.250	0.37	No	0.69
O.2	26	-0.947	26	-0.965	25	-0.855	24	-0.942	26	-0.937	3	-0.101	0.01	No	0.62
N.6	24	-0.809	24	-0.949	27	-0.878	9	-0.385	24	-0.836	1	0.116	0.19	No	0.61
C.7	1	0.644	4	0.532	4	0.642	1	0.757	3	0.437	7	-0.158	0.14	No	0.57
O.23	27	-0.947	27	-0.965	26	-0.855	25	-0.942	27	-0.937	4	-0.101	0.01	No	0.62
C.24	4	0.605	2	0.753	2	0.748	4	0.609	2	0.503	10	-0.250	0.37	No	0.69
O.26	25	-0.929	25	-0.954	24	-0.820	27	-0.950	25	-0.915	2	-0.076	0.01	No	0.69
C.27	2	0.617	3	0.750	3	0.705	2	0.642	4	0.367	11	-0.263	0.42	Yes	0.71

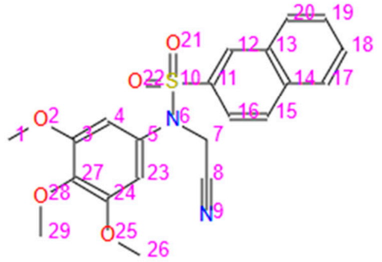

3															
	Rang	Delta P	Rang	Delta P	Rang	Delta P	Rang	Delta P	Rang	Delta P	Rang	Delta P			
C.1	2	0.600	1	0.720	1	0.738	2	0.675	1	0.483	11	-0.186	0.39	No	0.64
O.2	28	-0.894	28	-0.938	27	-0.805	26	-0.852	28	-0.883	3	-0.086	0.01	No	0.58
N.6	26	-0.735	27	-0.935	29	-0.857	15	-0.490	26	-0.793	1	0.106	0.20	No	0.57
C.7	8	0.154	6	-0.062	4	0.265	7	0.001	10	-0.094	10	-0.184	0.18	No	0.54
S.10	10	-0.016	12	-0.406	8	0.023	4	0.129	9	-0.087	17	-0.315	0.02	No	0.58
C.18	4	0.323	4	0.198	5	0.260	5	0.095	4	0.222	18	-0.375	0.08	No	0.54
O.25	29	-0.894	29	-0.938	28	-0.805	27	-0.852	29	-0.883	4	-0.086	0.01	No	0.58
C.26	3	0.600	2	0.720	2	0.738	3	0.675	2	0.483	12	-0.186	0.39	No	0.64
O.28	27	-0.865	26	-0.916	26	-0.761	29	-0.863	27	-0.850	2	-0.064	0.01	No	0.64
C.29	1	0.611	3	0.716	3	0.696	1	0.699	3	0.355	13	-0.193	0.63	Yes	0.66

Figure S4: Values obtained for the compounds **1**, **2**, and **3** from SOMP and FAME3 Software. The complete table can be found in Document S2.

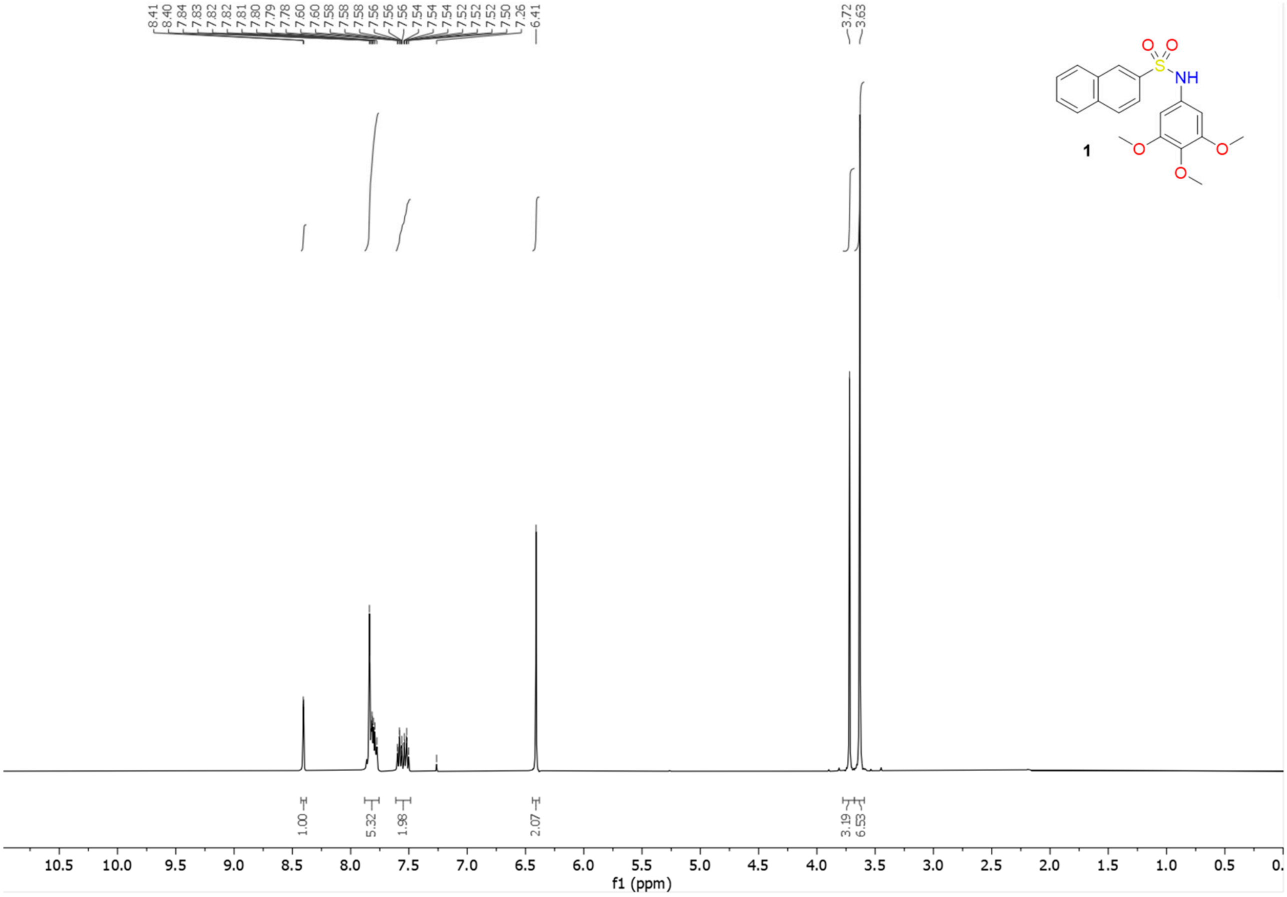
8.41
8.40
7.84
7.83
7.82
7.82
7.81
7.80
7.79
7.78
7.60
7.60
7.58
7.58
7.58
7.56
7.56
7.56
7.54
7.54
7.54
7.52
7.52
7.52
7.50
7.26
6.41
3.72
3.63
NH
1
1.00
5.32
1.98
2.07
3.19
6.53
f1 (ppm)

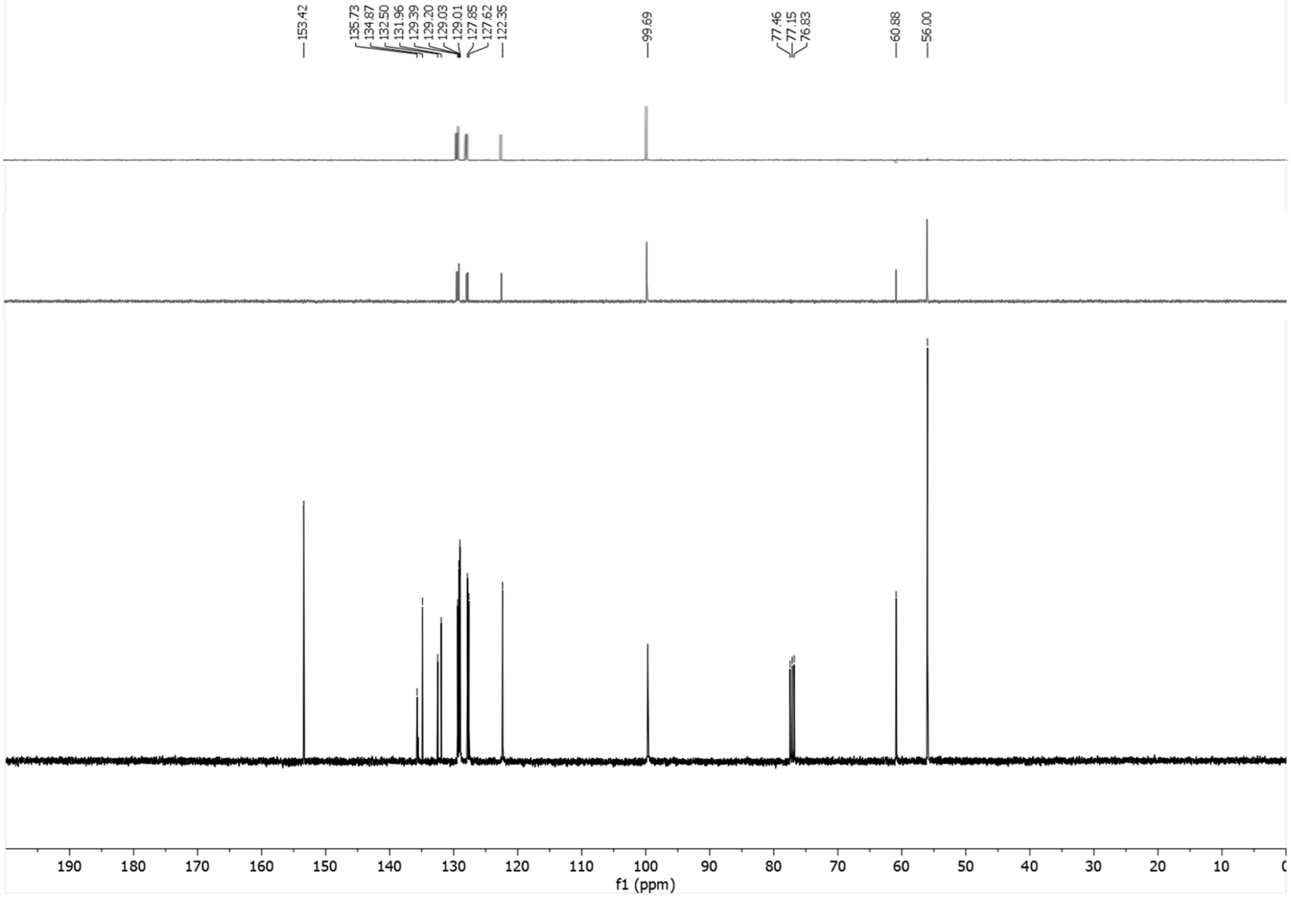
153.42
135.73
134.87
132.50
131.96
129.39
129.20
129.03
129.01
127.85
127.62
122.35
99.69
77.46
77.15
76.83
60.88
56.00
f1 (ppm)

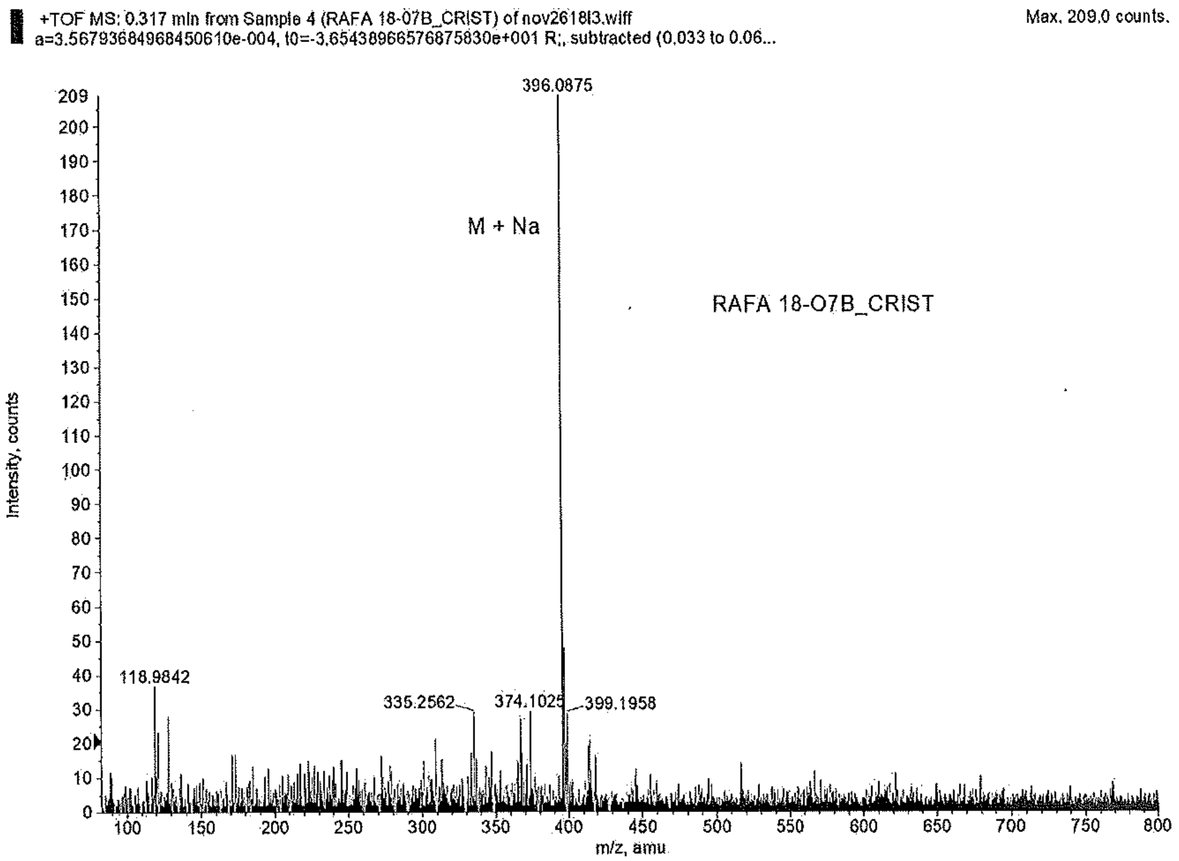

^{1}H, ^{13}C NMR and HRMS spectra of ***N*-(3,4,5-trimethoxyphenyl)naphthalene-2-sulfonamide (1)**

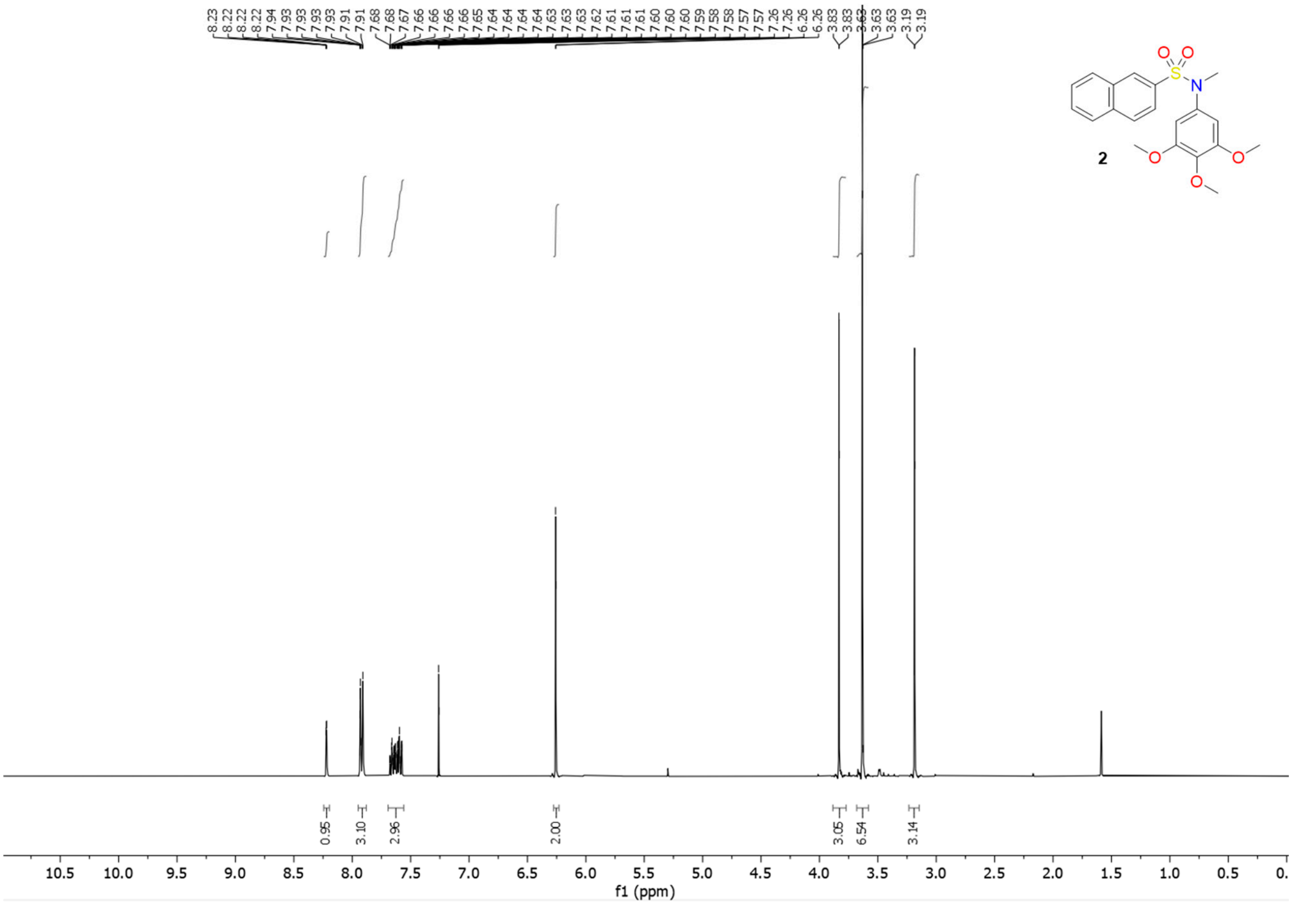
8.23 8.22 8.22 8.22 7.94 7.93 7.93 7.93 7.93 7.91 7.91 7.68 7.68 7.67 7.66 7.66 7.66 7.65 7.64 7.64 7.64 7.64 7.63 7.63 7.63 7.63 7.62 7.61 7.61 7.61 7.60 7.60 7.60 7.59 7.58 7.58 7.57 7.57 7.26 7.26 6.26 6.26 3.83 3.83 3.63 3.63 3.63 3.19 3.19
2
0.95
3.10
2.96
2.00
3.05
6.54
3.14
10.5 10.0 9.5 9.0 8.5 8.0 7.5 7.0 6.5 6.0 5.5 5.0 4.5 4.0 3.5 3.0 2.5 2.0 1.5 1.0 0.5
f1 (ppm)

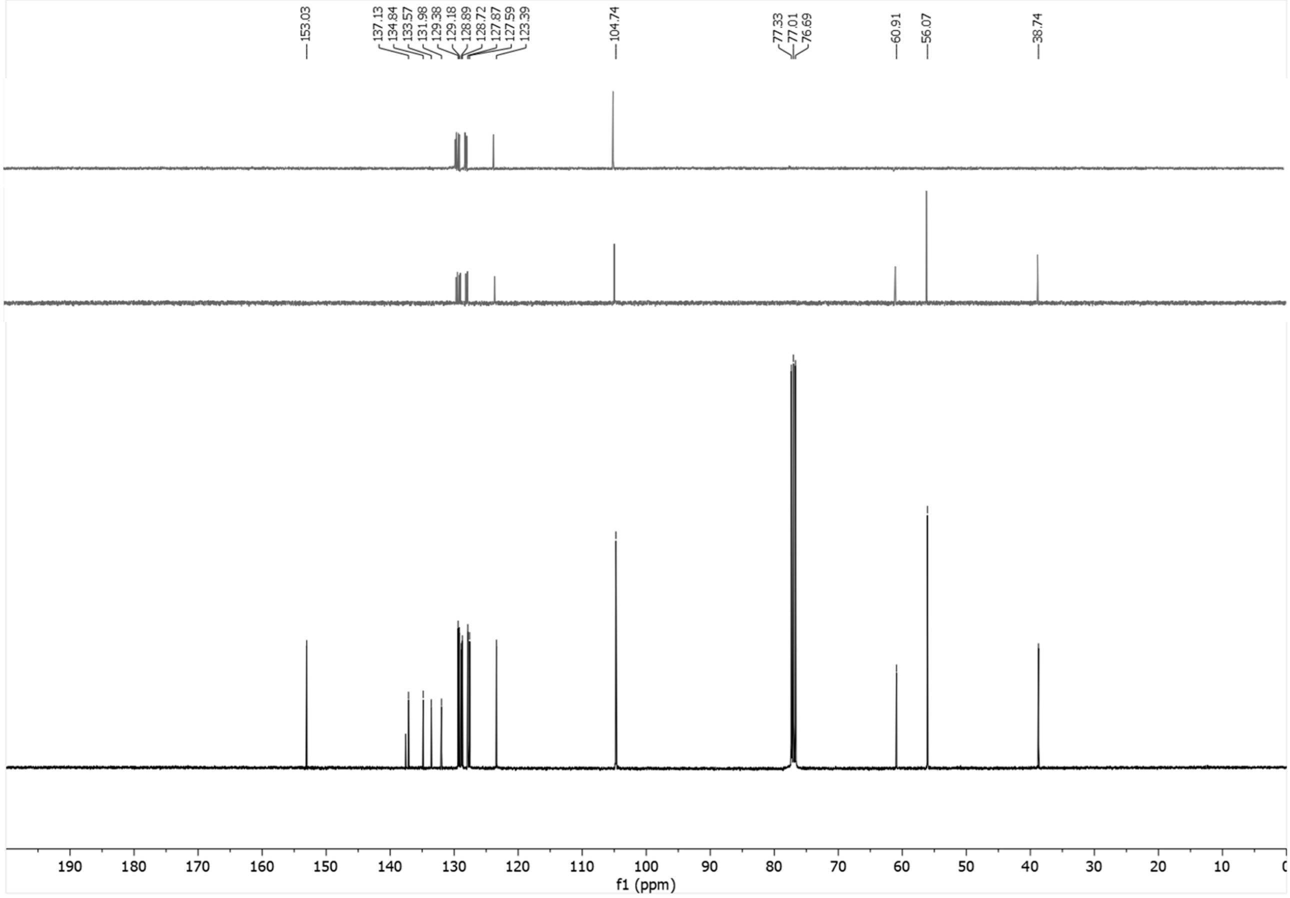
153.03
137.13 134.84 133.57 131.98 129.38 129.18 128.89 128.72 127.87 127.59 123.39
104.74
77.33 77.01 76.69
60.91
56.07
38.74
190 180 170 160 150 140 130 120 110 100 90 80 70 60 50 40 30 20 10
f1 (ppm)

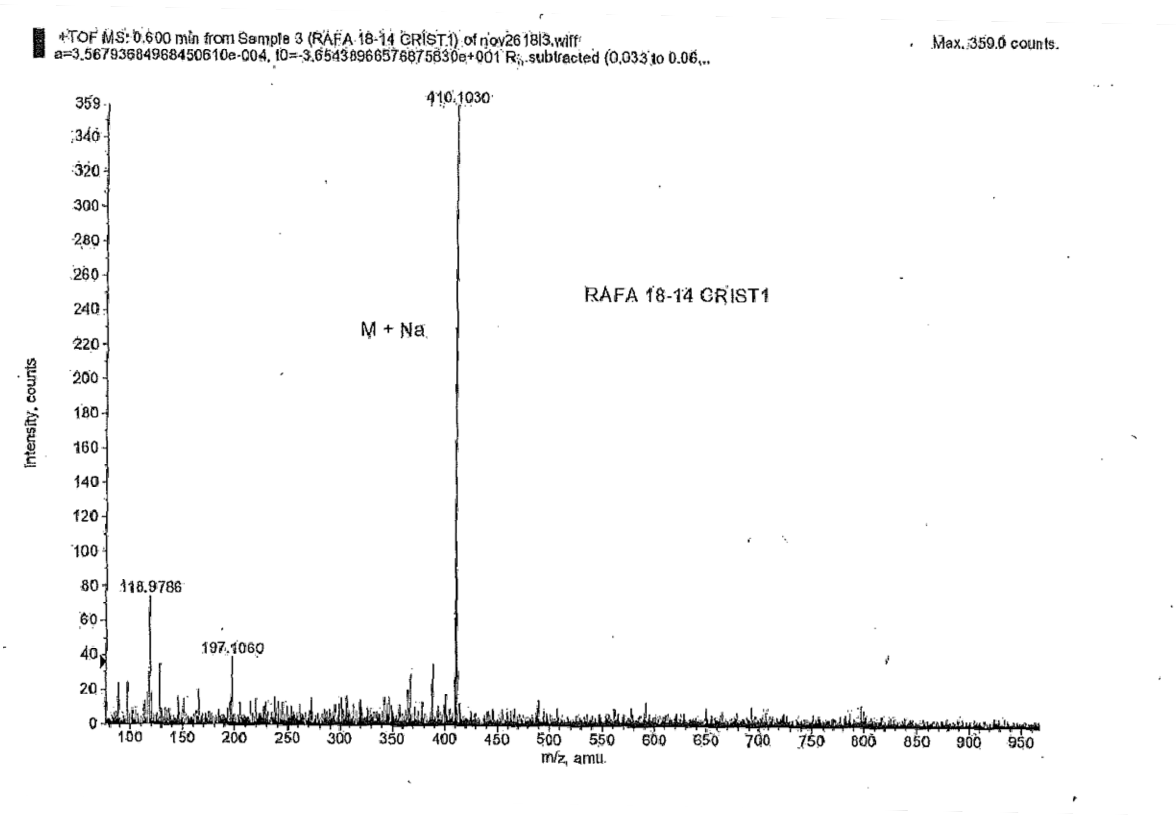

^{1}H, ^{13}C NMR and HRMS spectra of ***N*-methyl-*N*-(3,4,5-trimethoxyphenyl)naphthalene-2-sulfonamide (2)**

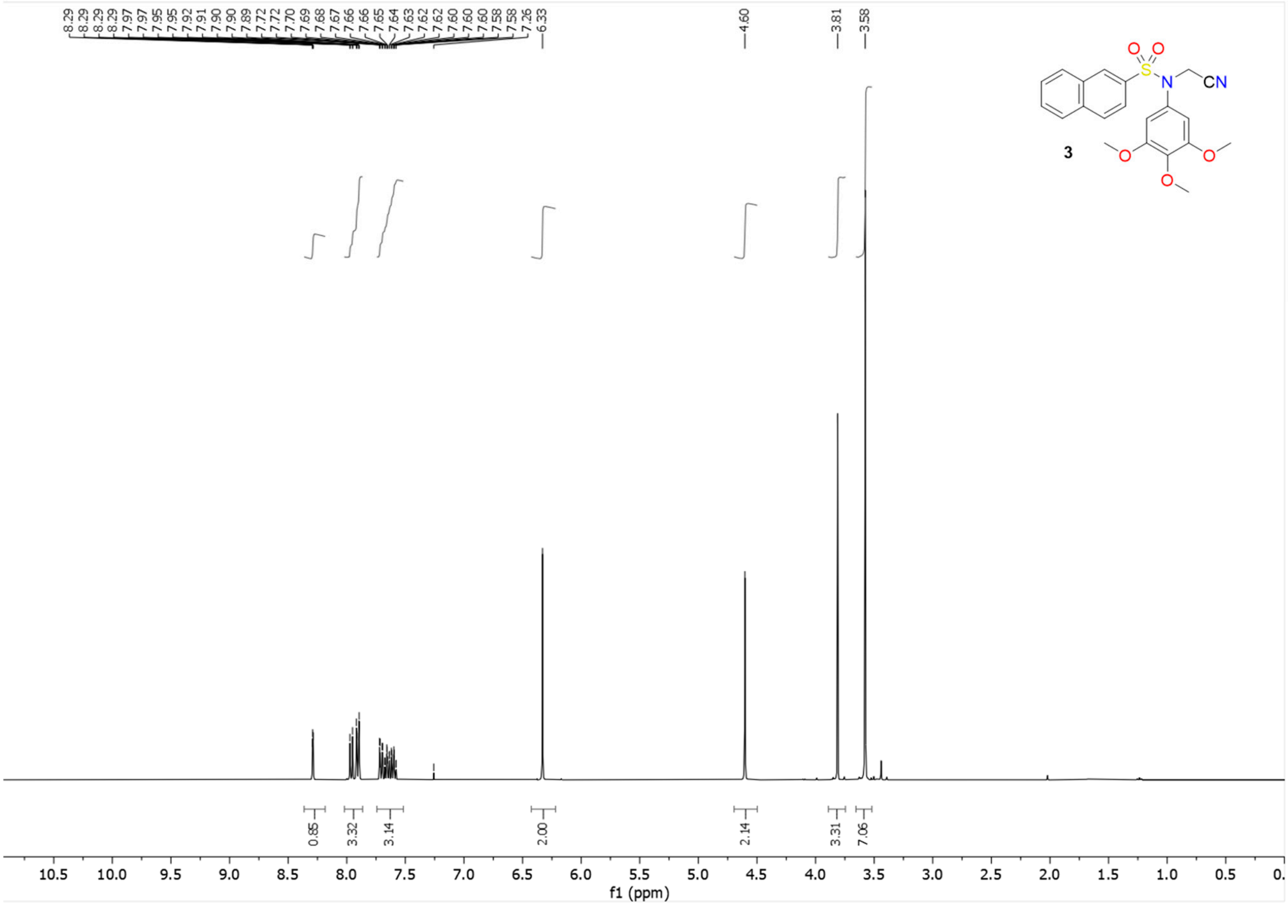
8.29
8.29
8.29
8.29
7.97
7.97
7.95
7.95
7.92
7.91
7.90
7.90
7.89
7.72
7.72
7.70
7.69
7.68
7.67
7.66
7.66
7.65
7.64
7.63
7.62
7.62
7.60
7.60
7.60
7.58
7.58
7.26
6.33
4.60
3.81
3.58
3
CN
0.85
3.32
3.14
2.00
2.14
3.31
7.06
f1 (ppm)

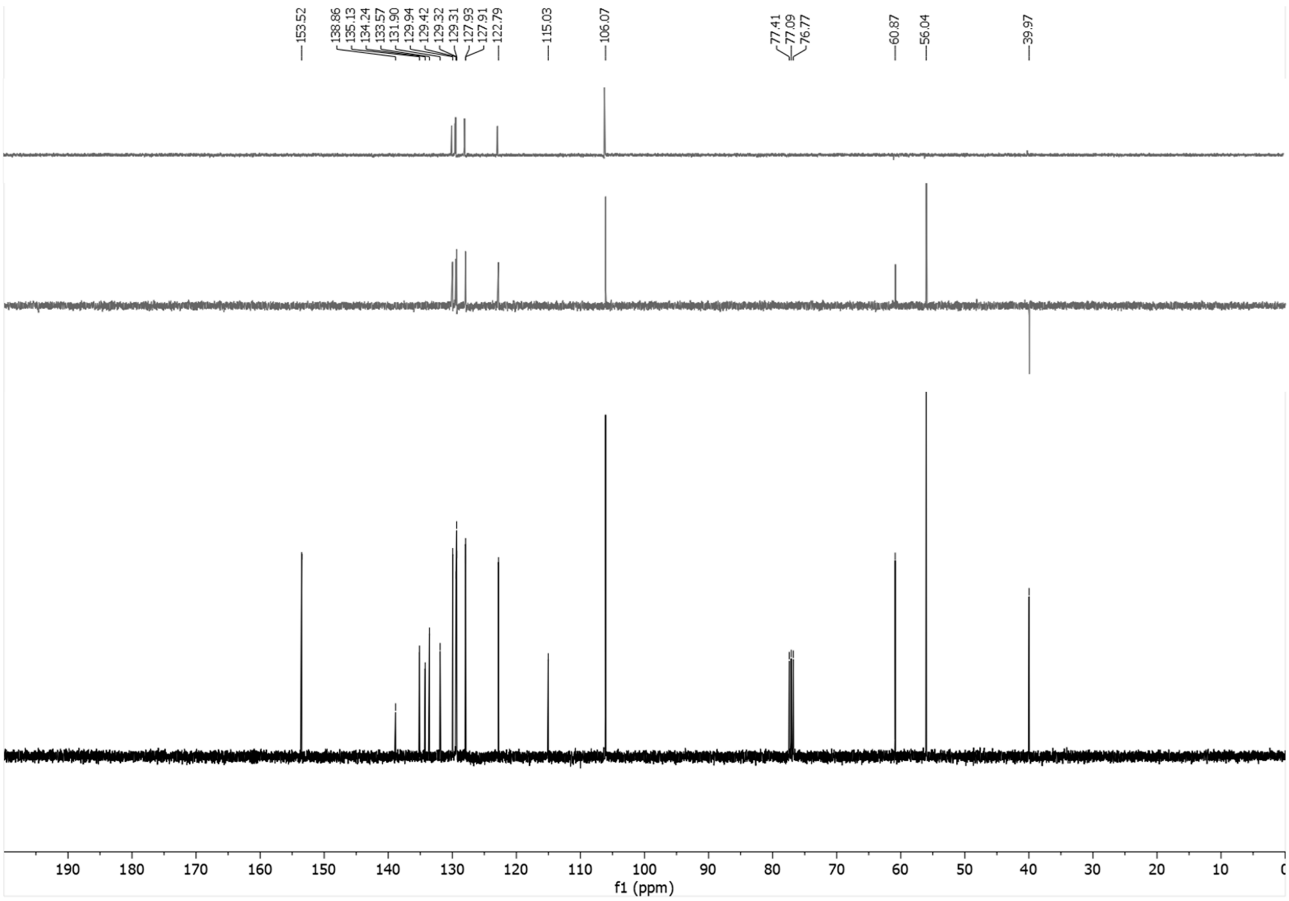

153.52
138.86
135.13
134.24
133.57
131.90
129.94
129.42
129.32
129.31
127.93
127.91
122.79
115.03
106.07
77.41
77.09
76.77
60.87
56.04
39.97
190 180 170 160 150 140 130 120 110 100 90 80 70 60 50 40 30 20 10 0
f1 (ppm)

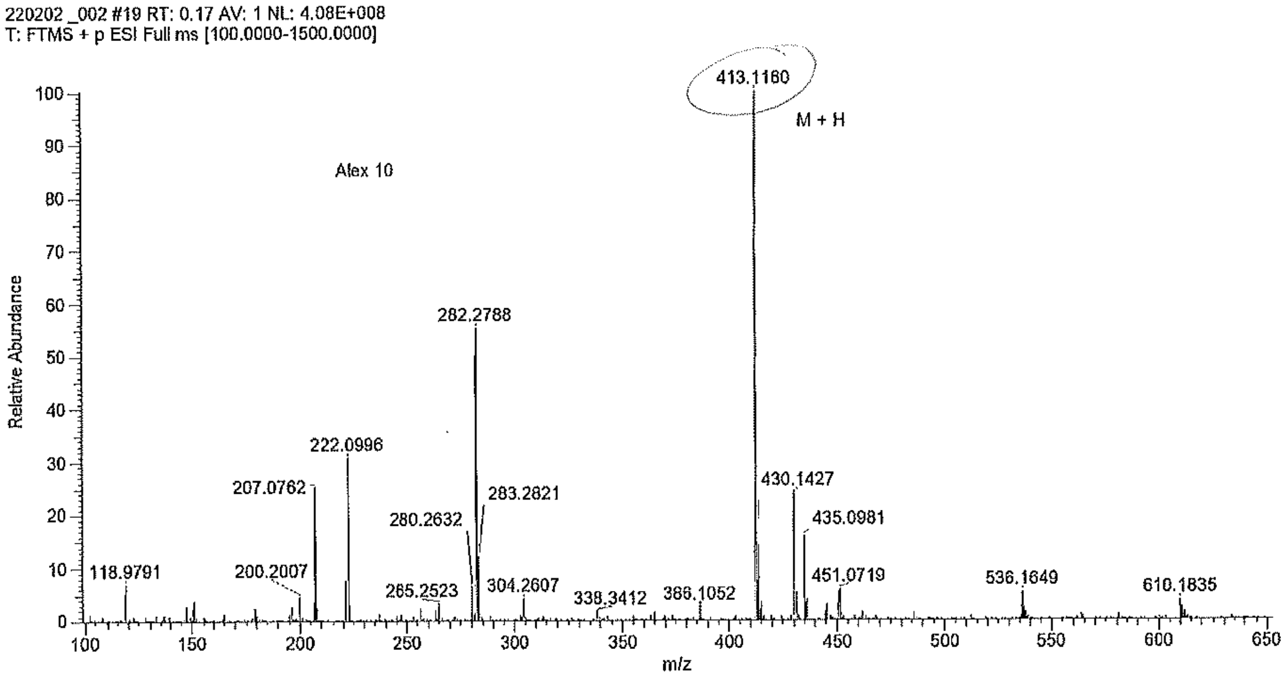

^{1}H, ^{13}C NMR and HRMS spectra of ***N*-(cyanomethyl)-*N*-(3,4,5-trimethoxyphenyl)naphthalene-2-sulfonamide (3)**

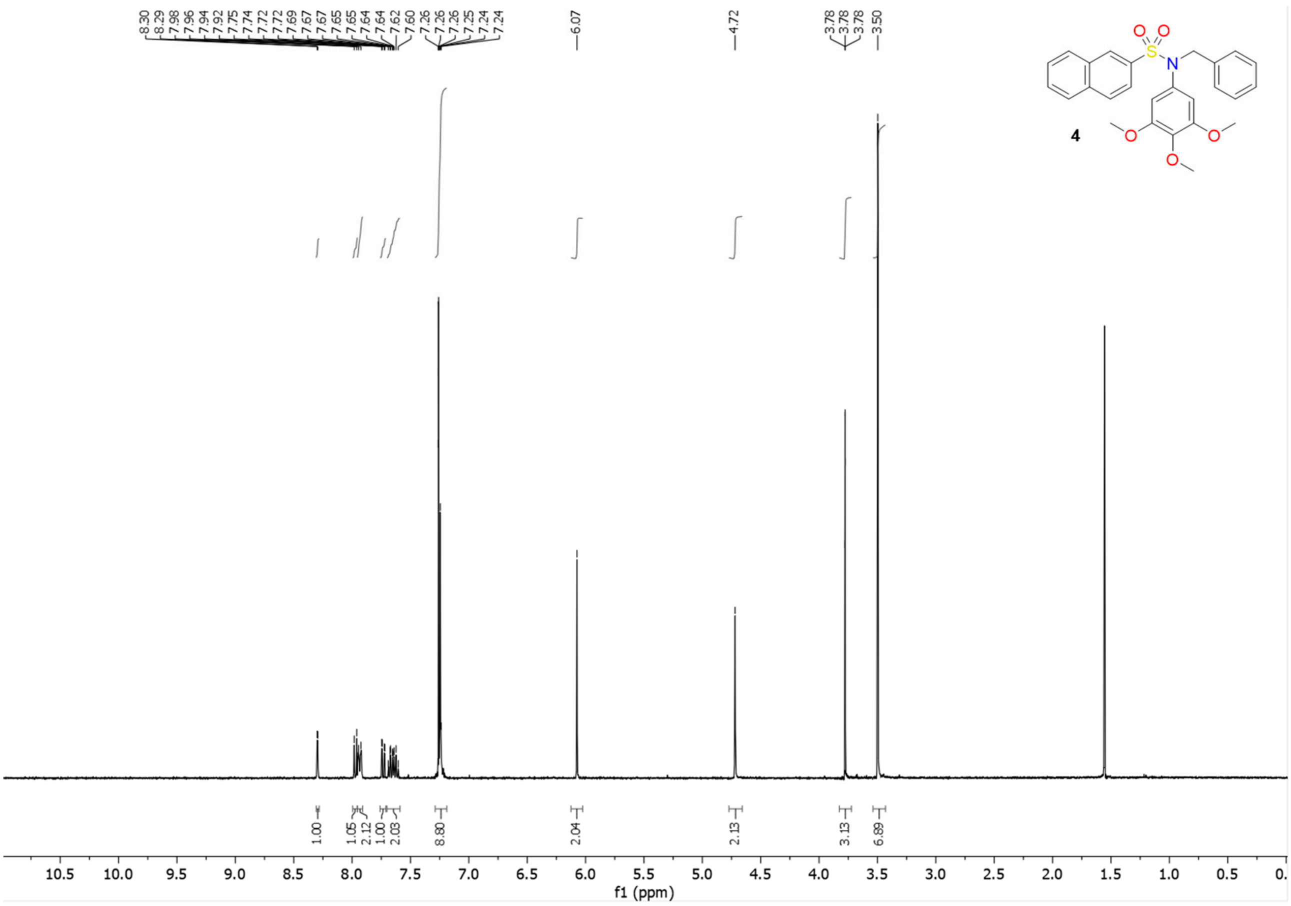
8.30 8.29 7.98 7.96 7.94 7.92 7.75 7.74 7.72 7.72 7.69 7.67 7.67 7.65 7.65 7.64 7.64 7.62 7.60 7.26 7.26 7.26 7.25 7.24 7.24
6.07
4.72
3.78 3.78 3.78
3.50
4
1.00 1.05 2.12 1.00 2.03 8.80 2.04 2.13 3.13 6.89
f1 (ppm)

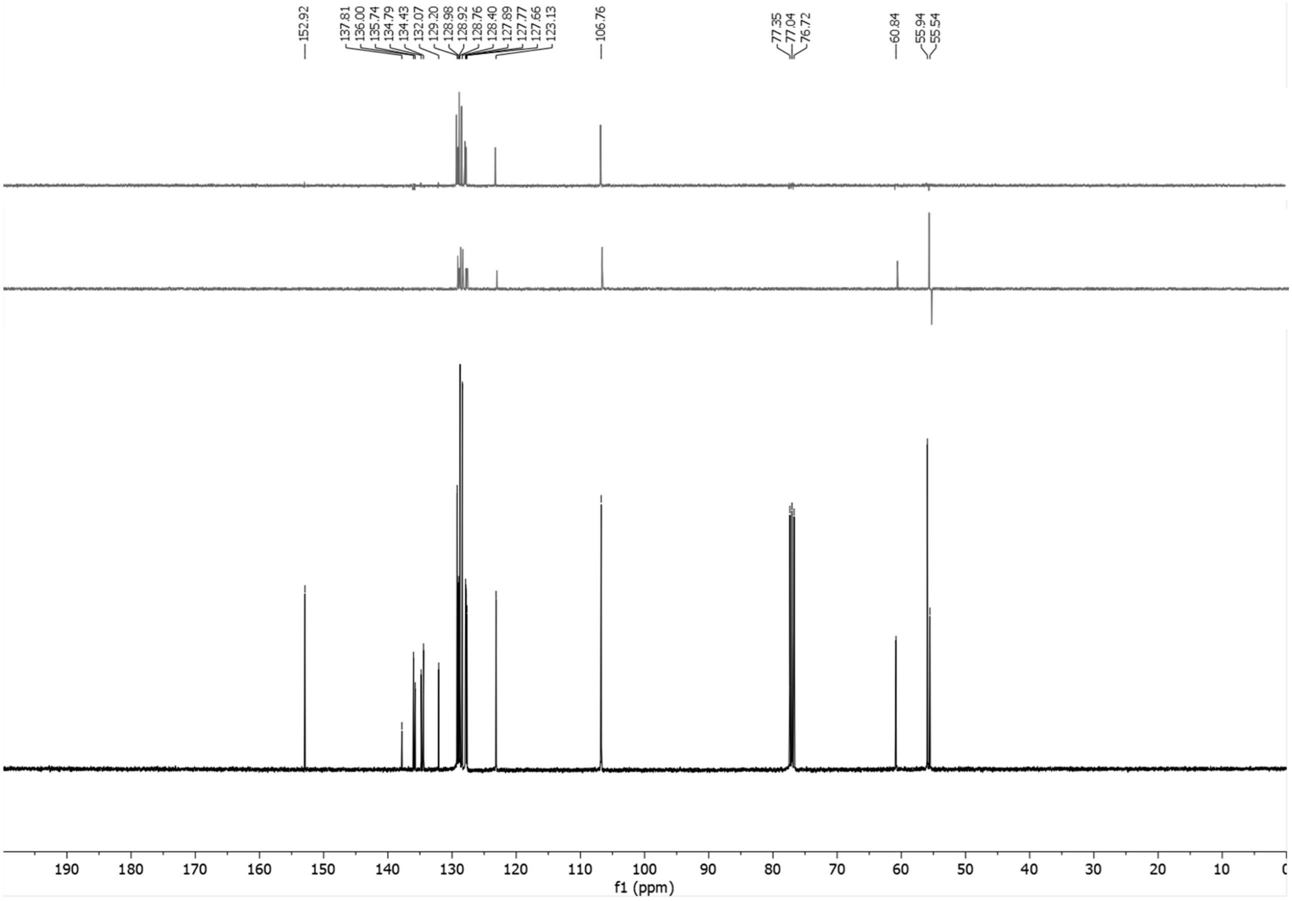
152.92
137.81 136.00 135.74 134.79 134.43 132.07 129.20 128.98 128.92 128.76 128.40 127.89 127.77 127.66 123.13
106.76
77.35 77.04 76.72
60.84
55.94 55.54
f1 (ppm)

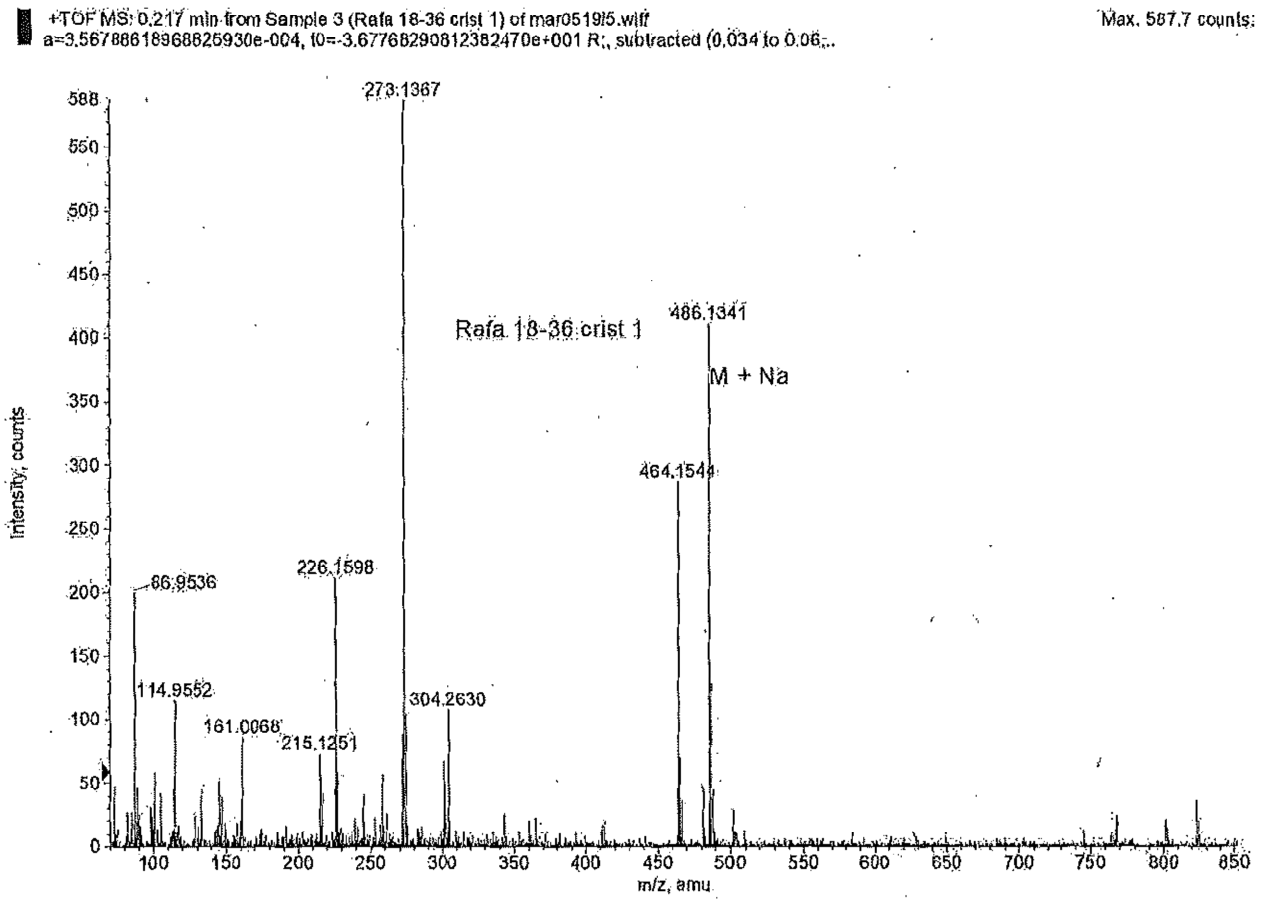

^{1}H, ^{13}C NMR and HRMS spectra of ***N*-benzyl-*N*-(3,4,5-trimethoxyphenyl)naphthalene-2-sulfonamide (4)**

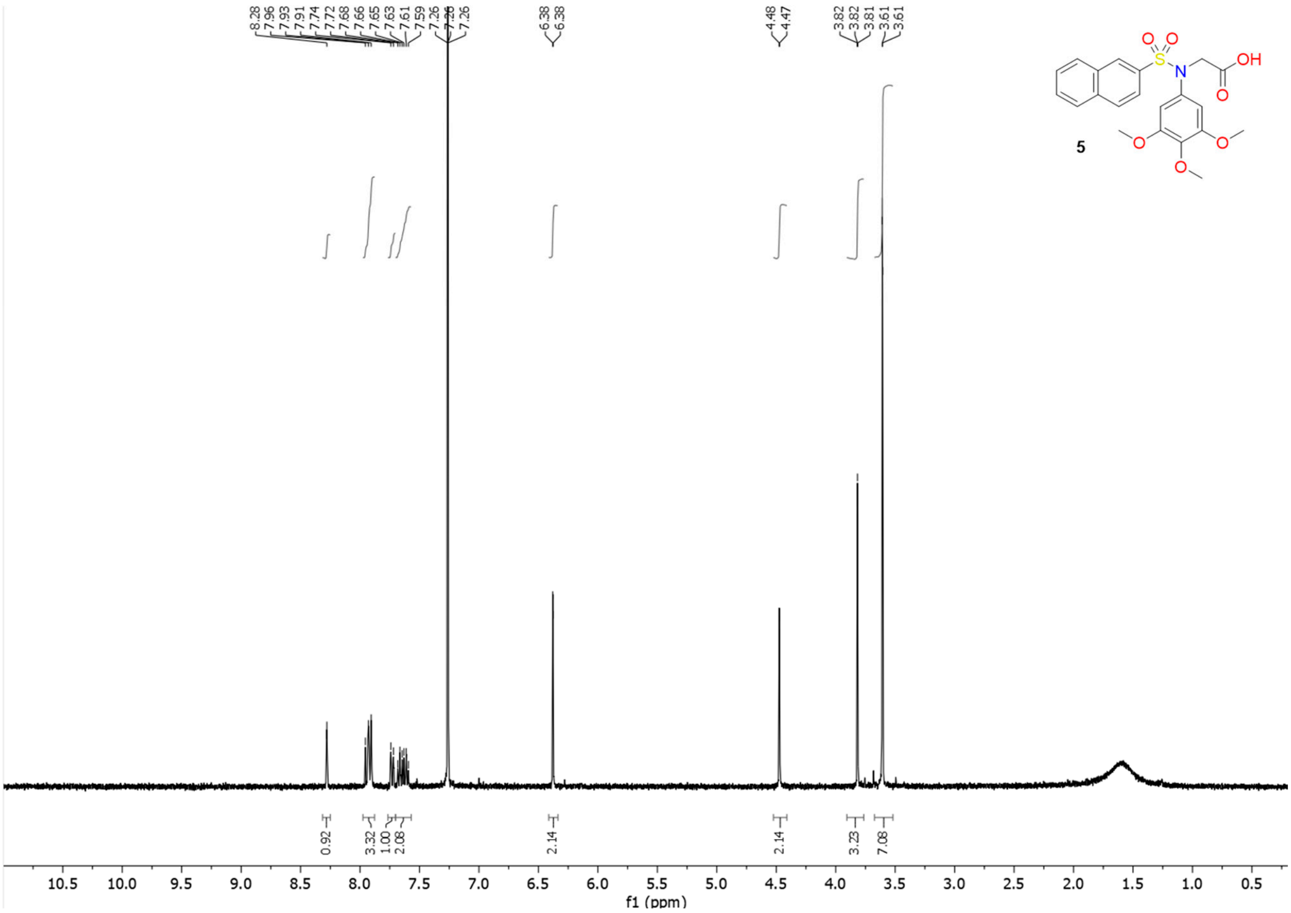
8.28 7.96 7.93 7.91 7.74 7.72 7.68 7.66 7.65 7.63 7.61 7.59 7.26 7.26 7.26
6.38 6.38
4.48 4.47
3.82 3.82 3.81 3.61 3.61
OH
O
S
O
O
N
O
O
O
5
0.92 3.32 1.00 2.08 2.14 2.14 3.23 7.08
10.5 10.0 9.5 9.0 8.5 8.0 7.5 7.0 6.5 6.0 5.5 5.0 4.5 4.0 3.5 3.0 2.5 2.0 1.5 1.0 0.5
f1 (ppm)

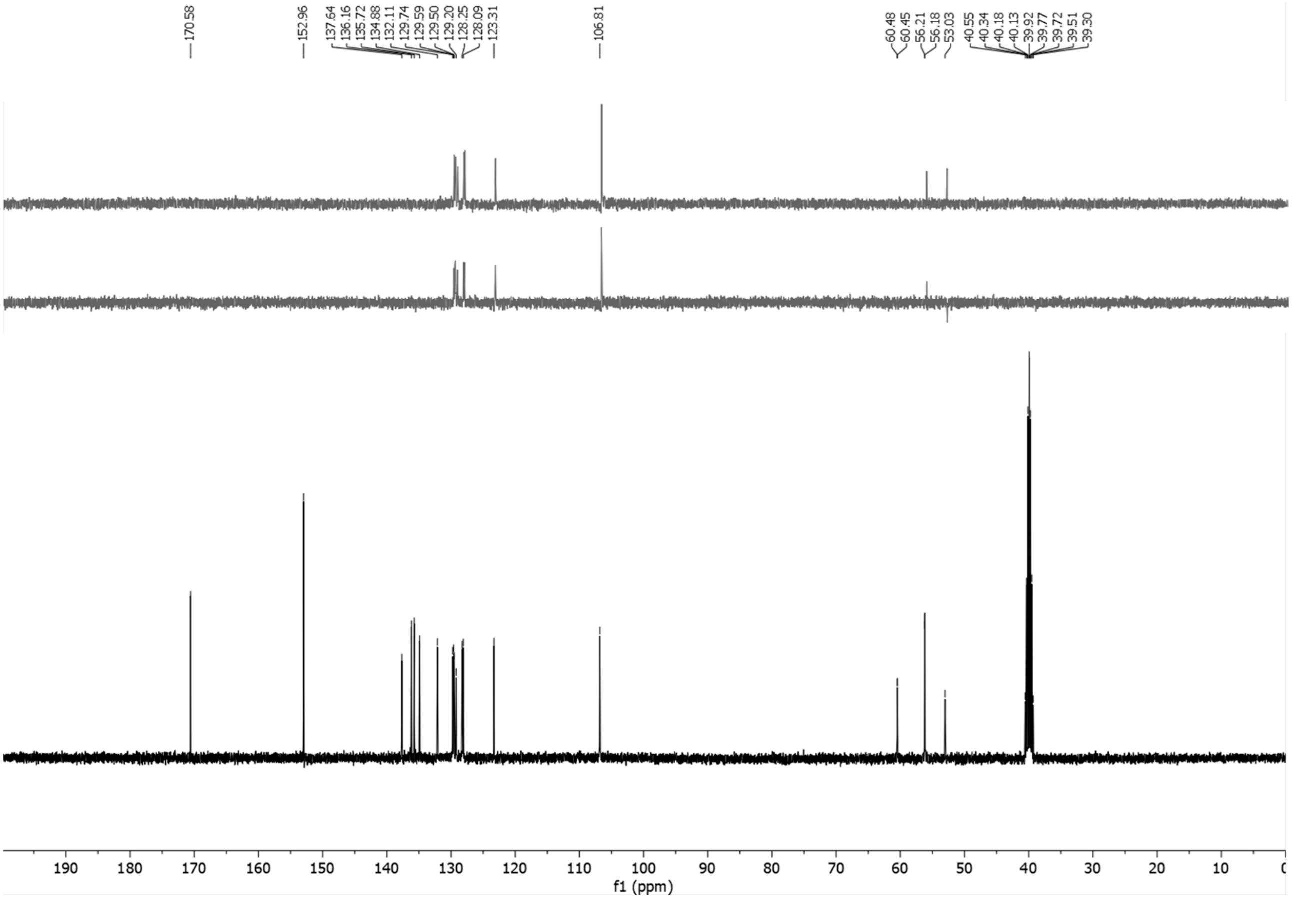
170.58
152.96
137.64 136.16 135.72 134.88 132.11 129.74 129.59 129.50 129.20 128.25 128.09 123.31
106.81
60.48 60.45 56.21 56.18 53.03 40.55 40.34 40.18 40.13 39.92 39.77 39.72 39.51 39.30
190 180 170 160 150 140 130 120 110 100 90 80 70 60 50 40 30 20 10
f1 (ppm)

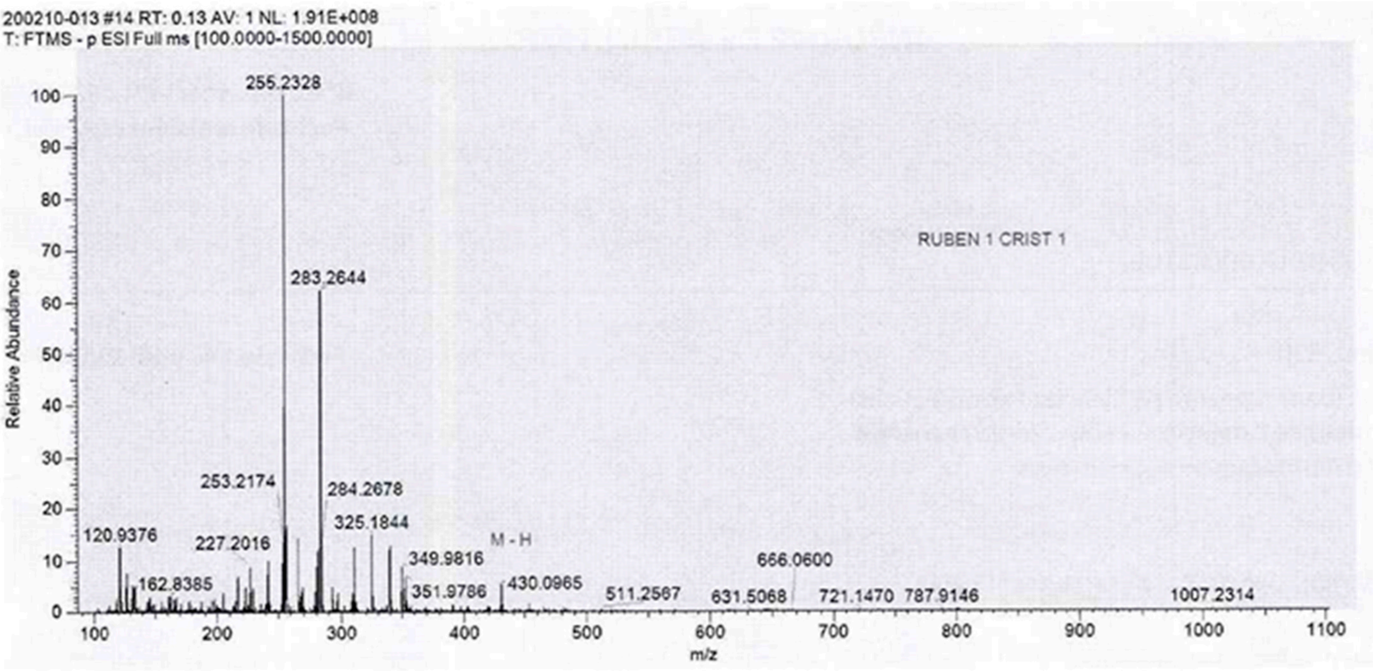

^{1}H, ^{13}C NMR and HRMS spectra of ***N*-(naphthalen-2-ylsulfonyl)-*N*-(3,4,5-trimethoxyphenyl)glycine (5)**

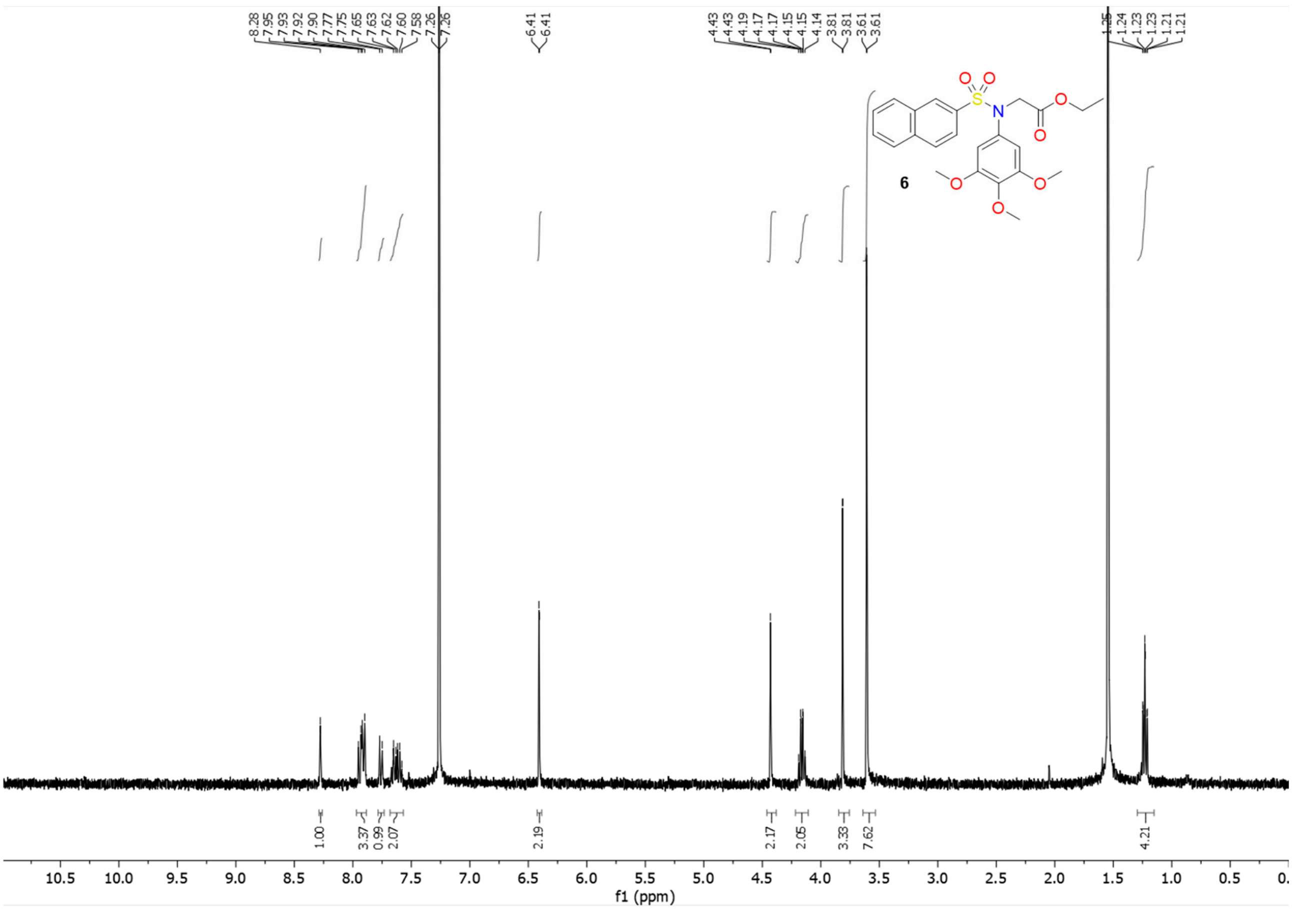
8.28
7.95
7.93
7.92
7.90
7.77
7.75
7.65
7.63
7.62
7.60
7.58
7.26
7.26
6.41
6.41
4.43
4.43
4.19
4.17
4.17
4.15
4.15
4.14
3.81
3.81
3.61
3.61
1.25
1.24
1.23
1.23
1.21
1.21
6
1.00
3.37
0.99
2.07
2.19
2.17
2.05
3.33
7.62
4.21
f1 (ppm)

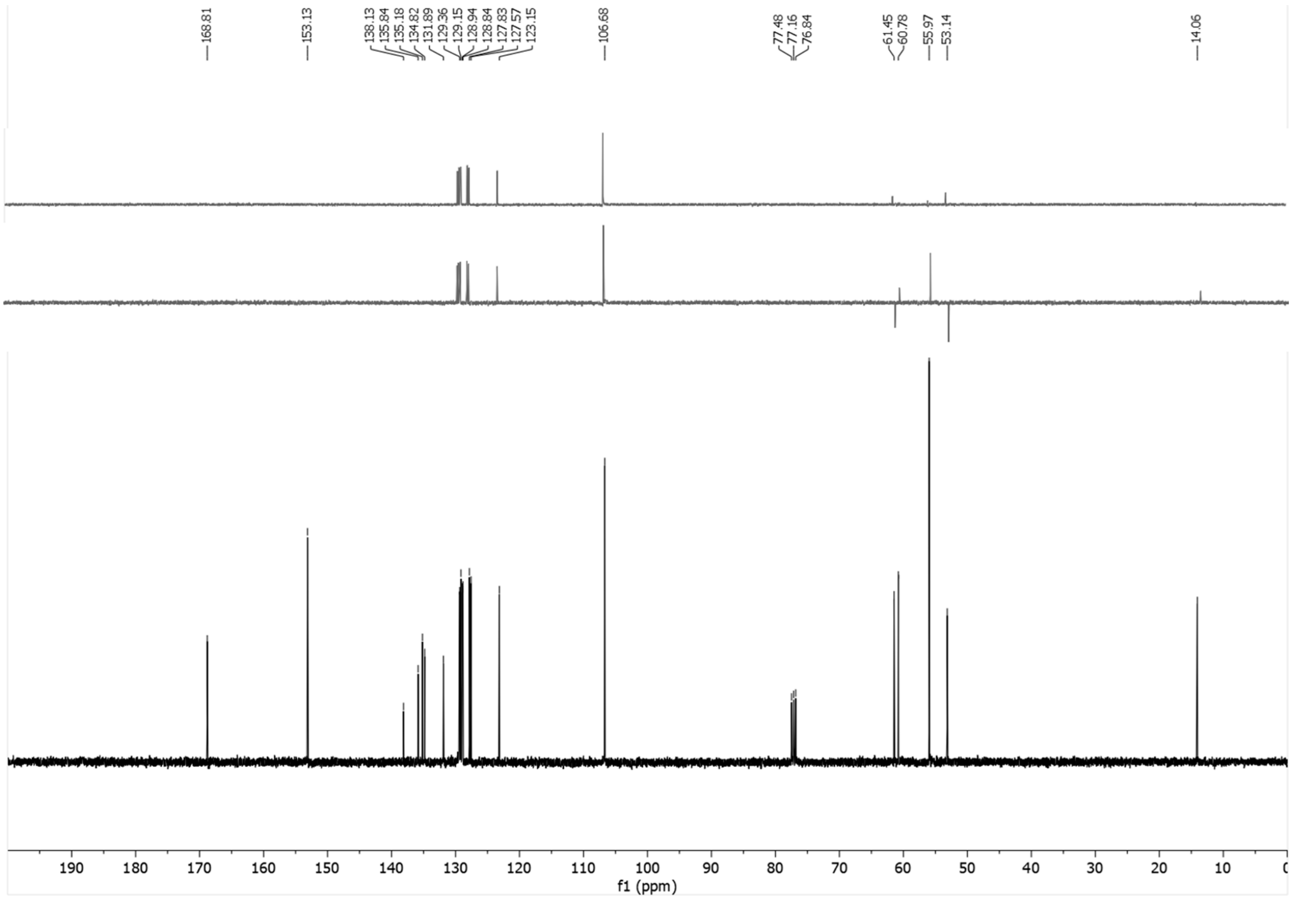
168.81
153.13
138.13
135.84
135.18
134.82
131.89
129.36
129.15
128.94
128.84
127.83
127.57
123.15
106.68
77.48
77.16
76.84
61.45
60.78
55.97
53.14
14.06
f1 (ppm)

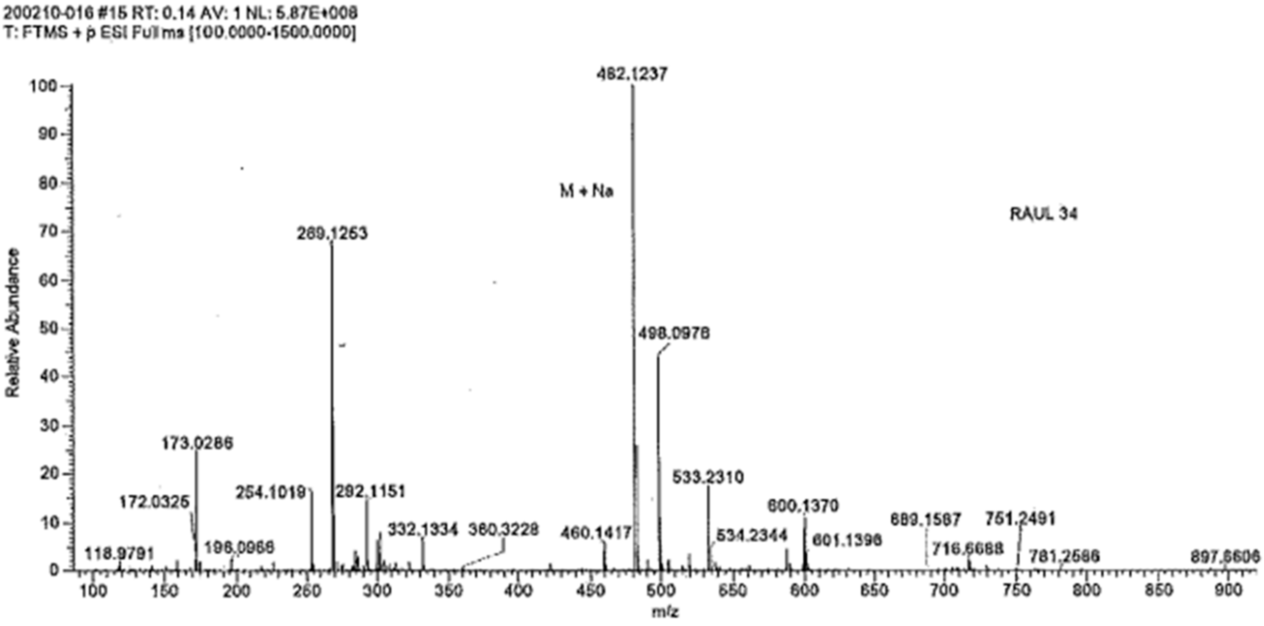

^{1}H, ^{13}C NMR and HRMS spectra of **ethyl *N*-(naphthalen-2-ylsulfonyl)-*N*-(3,4,5-trimethoxyphenyl)glycinate (6)**

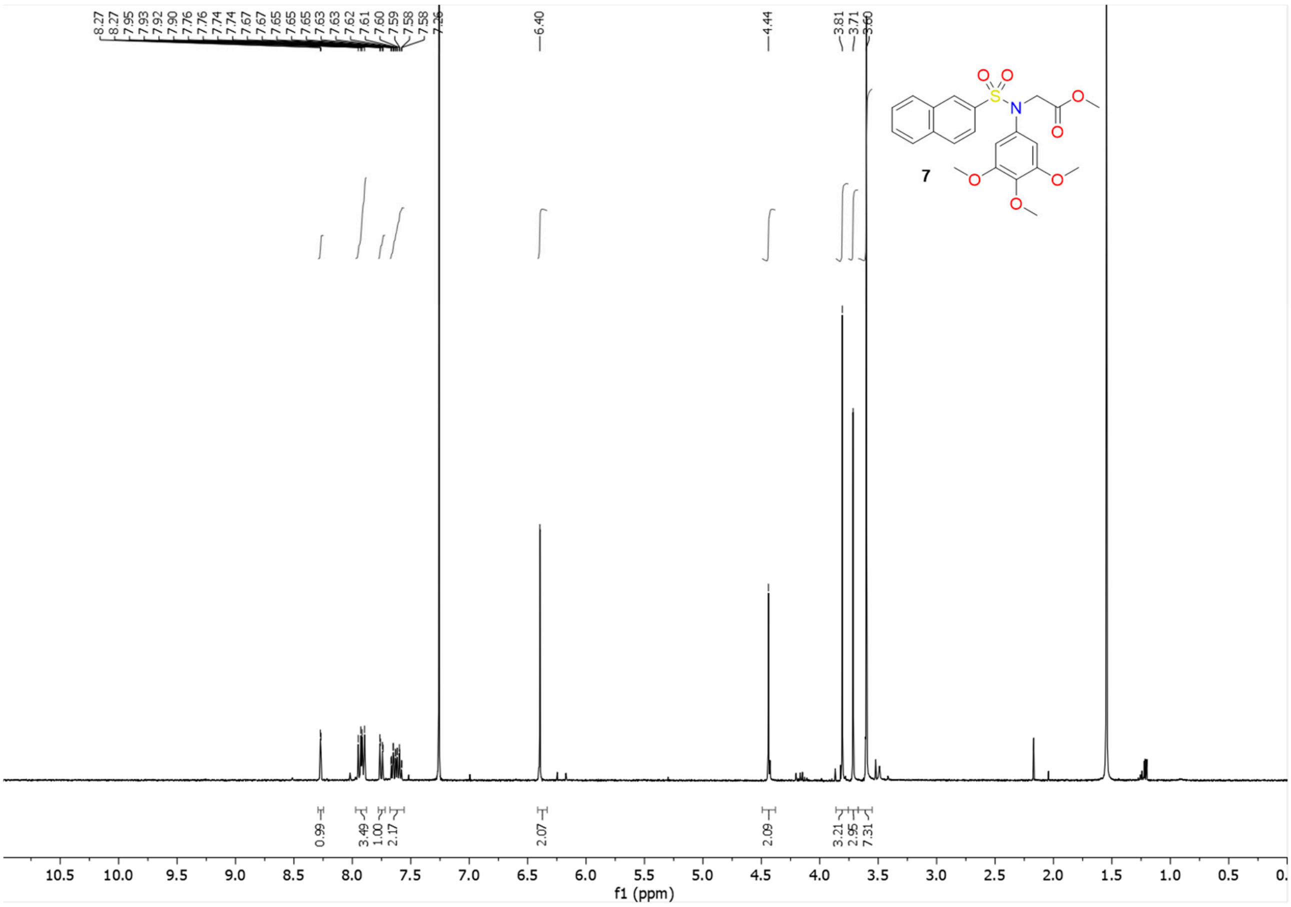
8.27 8.27 7.95 7.93 7.92 7.90 7.76 7.76 7.74 7.74 7.67 7.67 7.65 7.65 7.65 7.63 7.63 7.62 7.62 7.61 7.60 7.59 7.58 7.58 7.26
6.40
4.44
3.81 3.71 3.60
7
0.99 3.49 1.00 2.17 2.07 2.09 3.21 2.95 7.31
f1 (ppm)

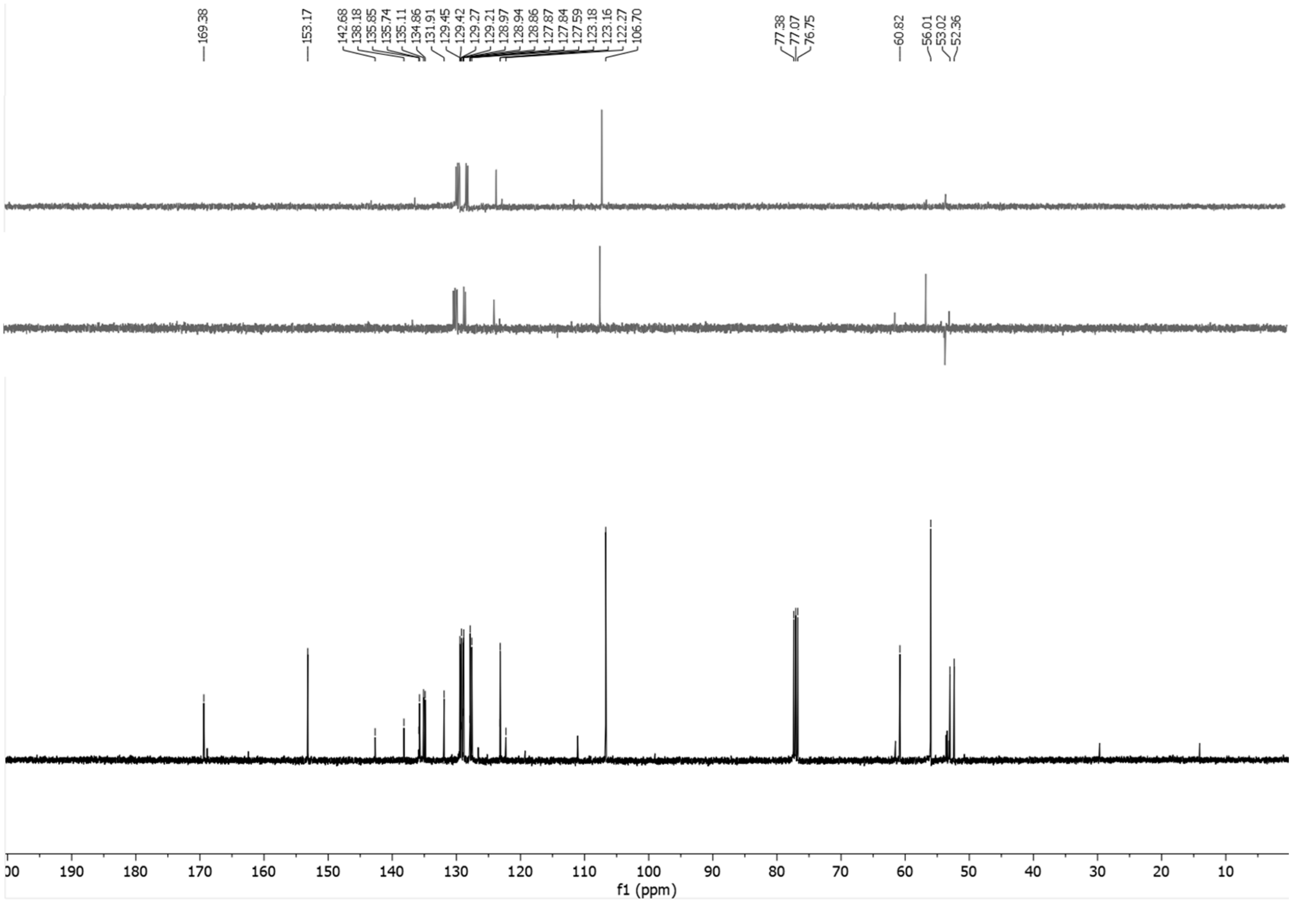
169.38
153.17
142.68 138.18 135.85 135.74 135.11 134.86 131.91 129.45 129.42 129.27 129.21 128.97 128.94 128.86 127.87 127.84 127.59 123.18 123.16 122.27 106.70
77.38 77.07 76.75
60.82 56.01 53.02 52.36
f1 (ppm)

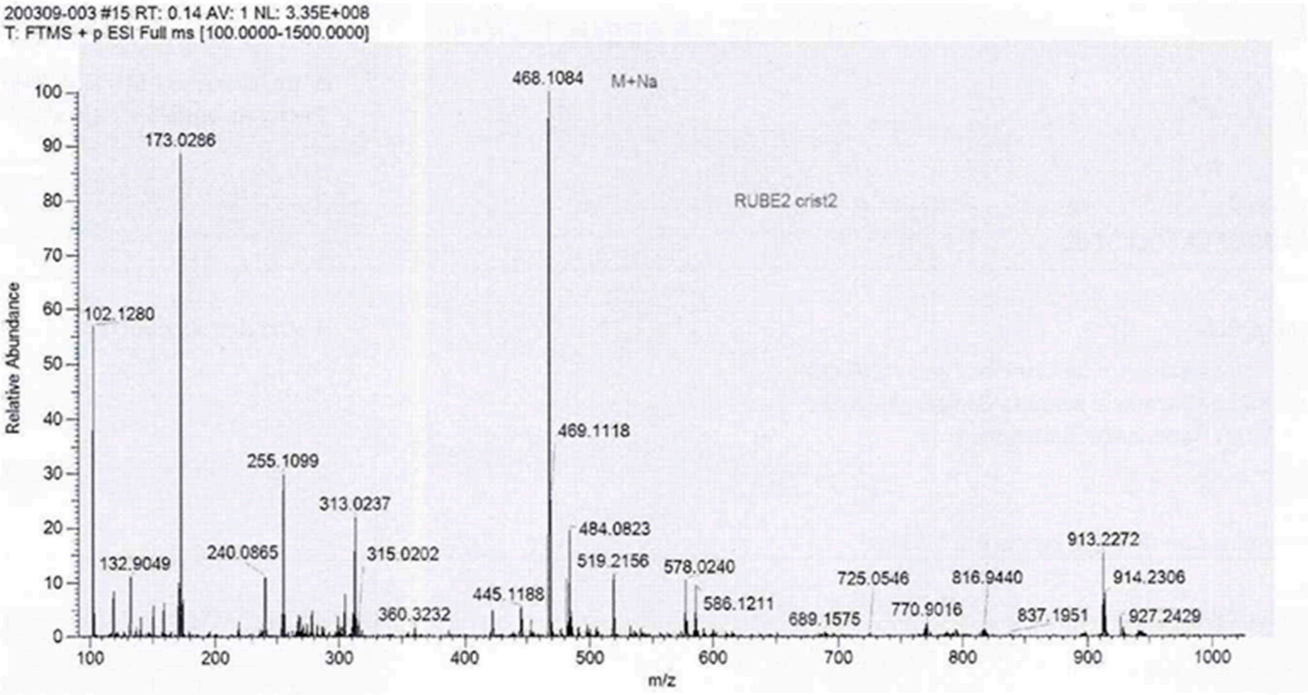

^{1}H, ^{13}C NMR and HRMS spectra of ***N*-(2-oxopropyl)-*N*-(3,4,5-trimethoxyphenyl)naphthalene-2-sulfonamide (7)**

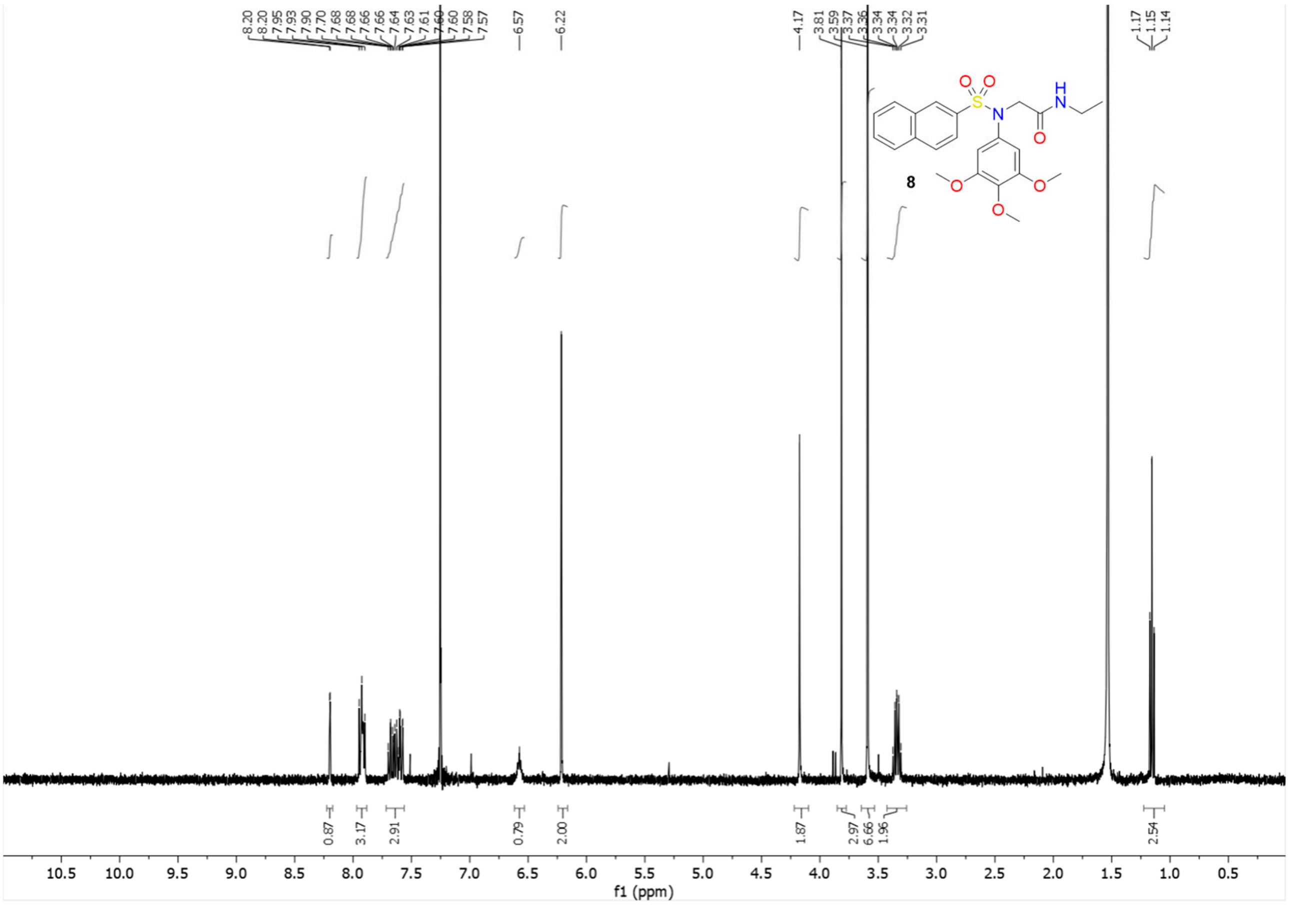

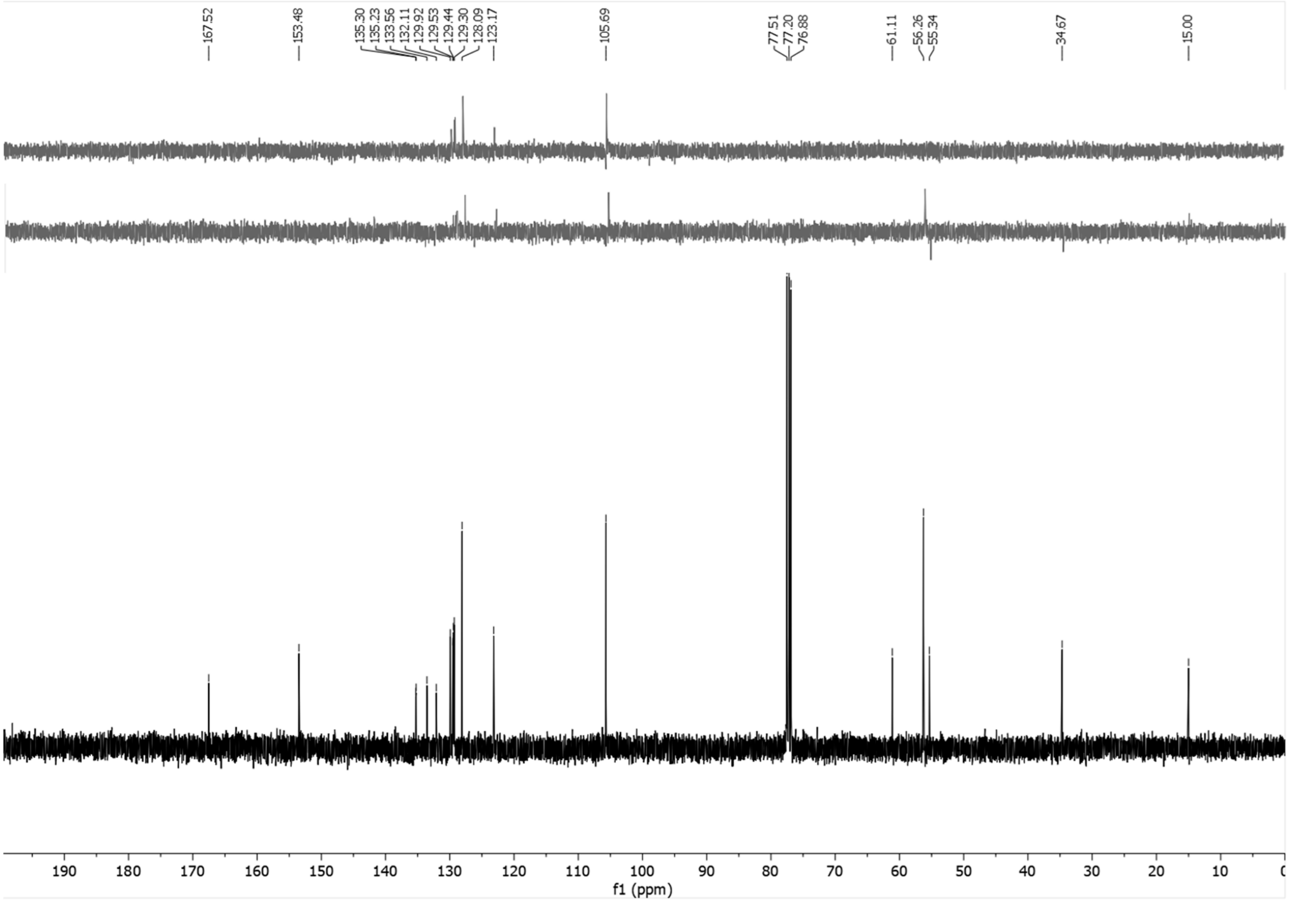

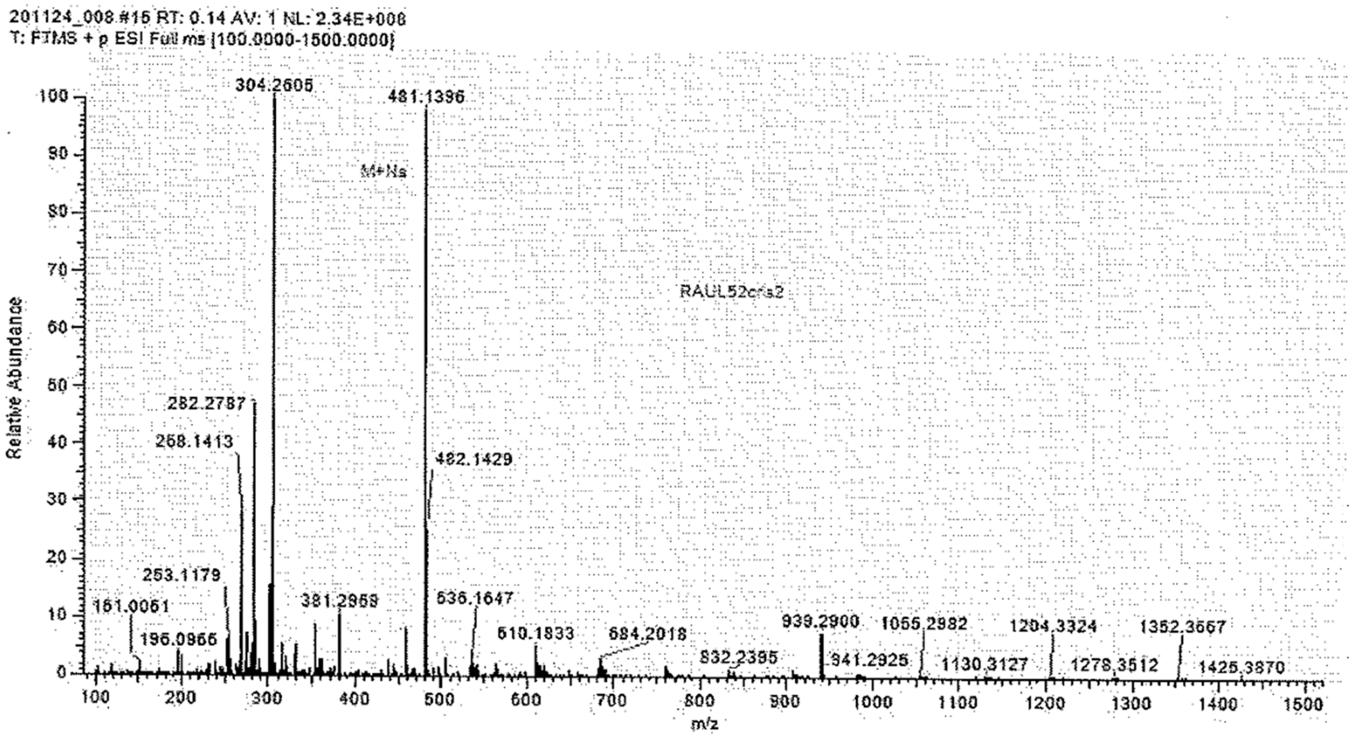

^{1}H, ^{13}C NMR and HRMS spectra of ***N*-ethyl-2-(*N*-(3,4,5-trimethoxyphenyl)naphthalene-2-sulfonamido)acetamide (8)**

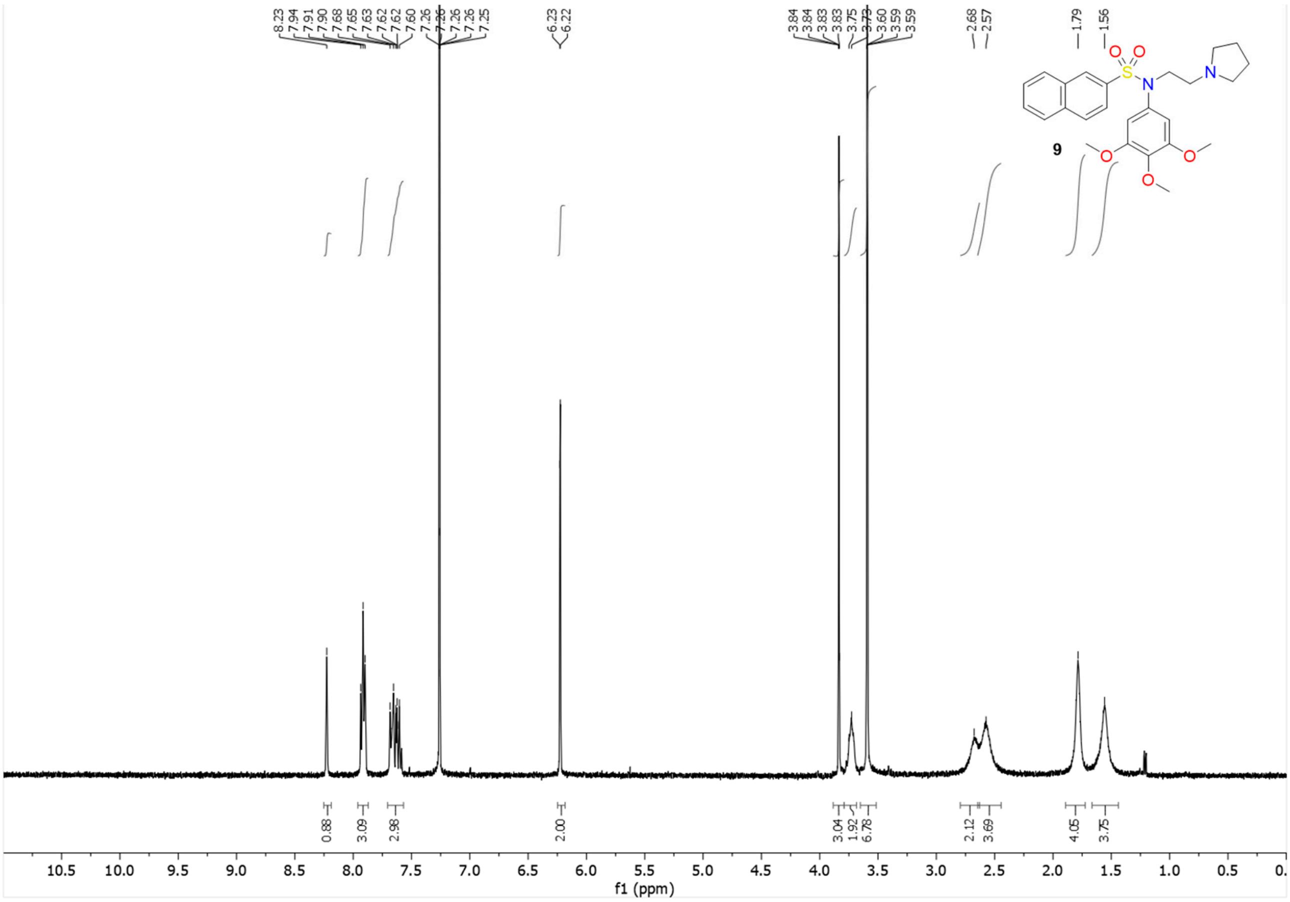
8.23
7.94
7.91
7.90
7.68
7.65
7.63
7.62
7.62
7.60
7.26
7.26
7.26
7.25
6.23
6.22
3.84
3.84
3.83
3.83
3.75
3.60
3.59
3.59
2.68
2.57
1.79
1.56
9
0.88
3.09
2.98
2.00
3.04
1.92
6.78
2.12
3.69
4.05
3.75
10.5
10.0
9.5
9.0
8.5
8.0
7.5
7.0
6.5
6.0
5.5
5.0
4.5
4.0
3.5
3.0
2.5
2.0
1.5
1.0
0.5
f1 (ppm)

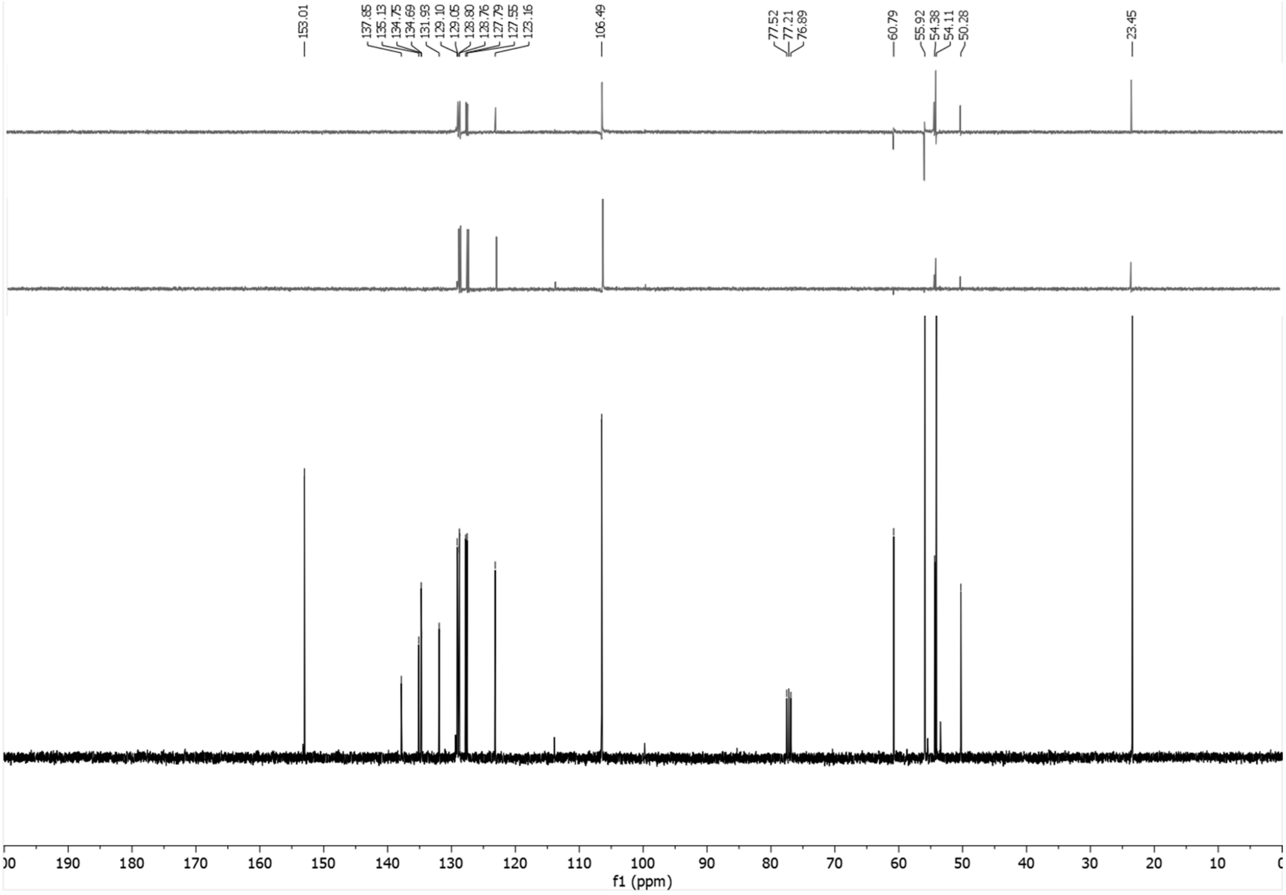
153.01
137.85
135.13
134.75
134.69
131.93
129.10
129.05
128.80
128.76
127.79
127.55
123.16
106.49
77.52
77.21
76.89
60.79
55.92
54.38
54.11
50.28
23.45
190
180
170
160
150
140
130
120
110
100
90
80
70
60
50
40
30
20
10
f1 (ppm)

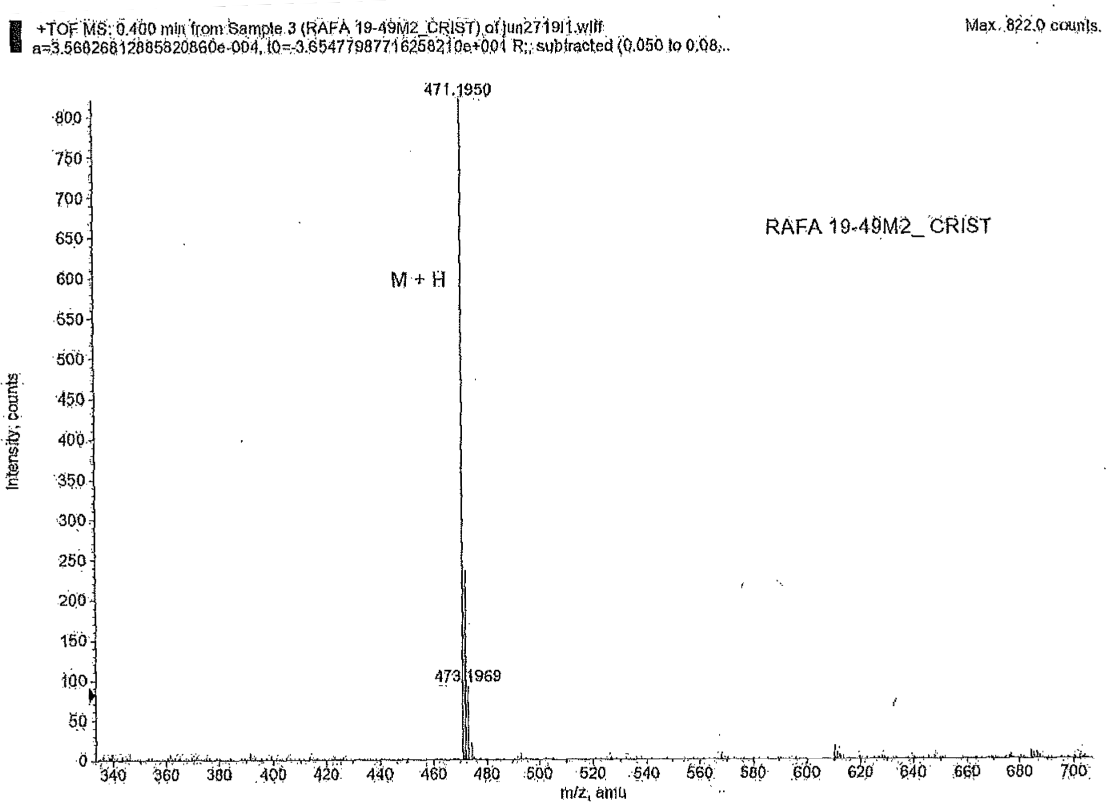

^1H, ^{13}C NMR and HRMS spectra of ***N*-(2-(pyrrolidin-1-yl)ethyl)-*N*-(3,4,5-trimethoxyphenyl)naphthalene-2-sulfonamide (9)**

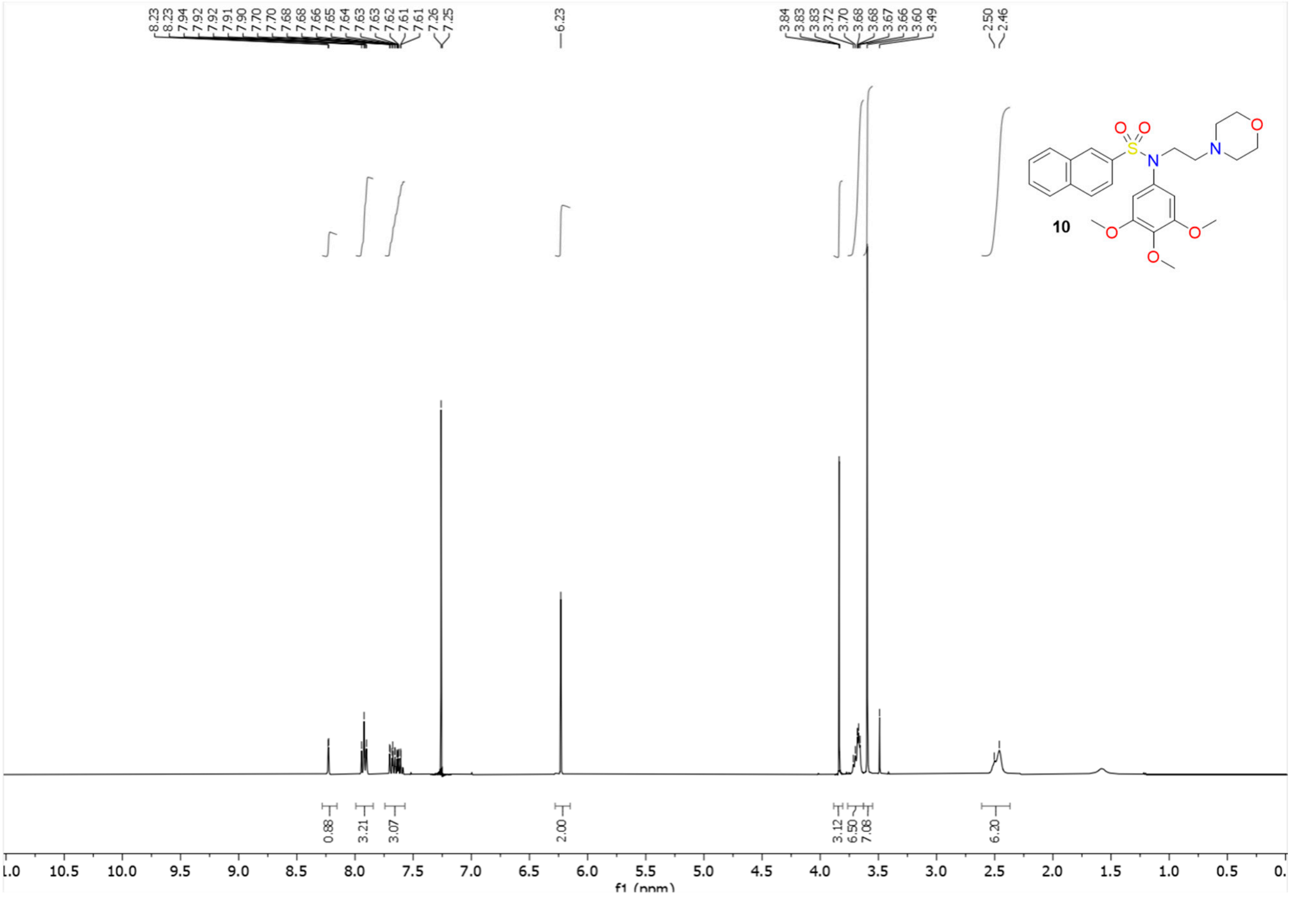
8.23 8.23 7.94 7.92 7.92 7.91 7.90 7.70 7.70 7.68 7.68 7.66 7.65 7.64 7.63 7.63 7.62 7.61 7.61 7.26 7.25
6.23
3.84 3.83 3.83 3.72 3.70 3.68 3.68 3.67 3.66 3.60 3.49
2.50 2.46
10
0.88 3.21 3.07 2.00 3.12 6.50 7.08 6.20
f1 (ppm)

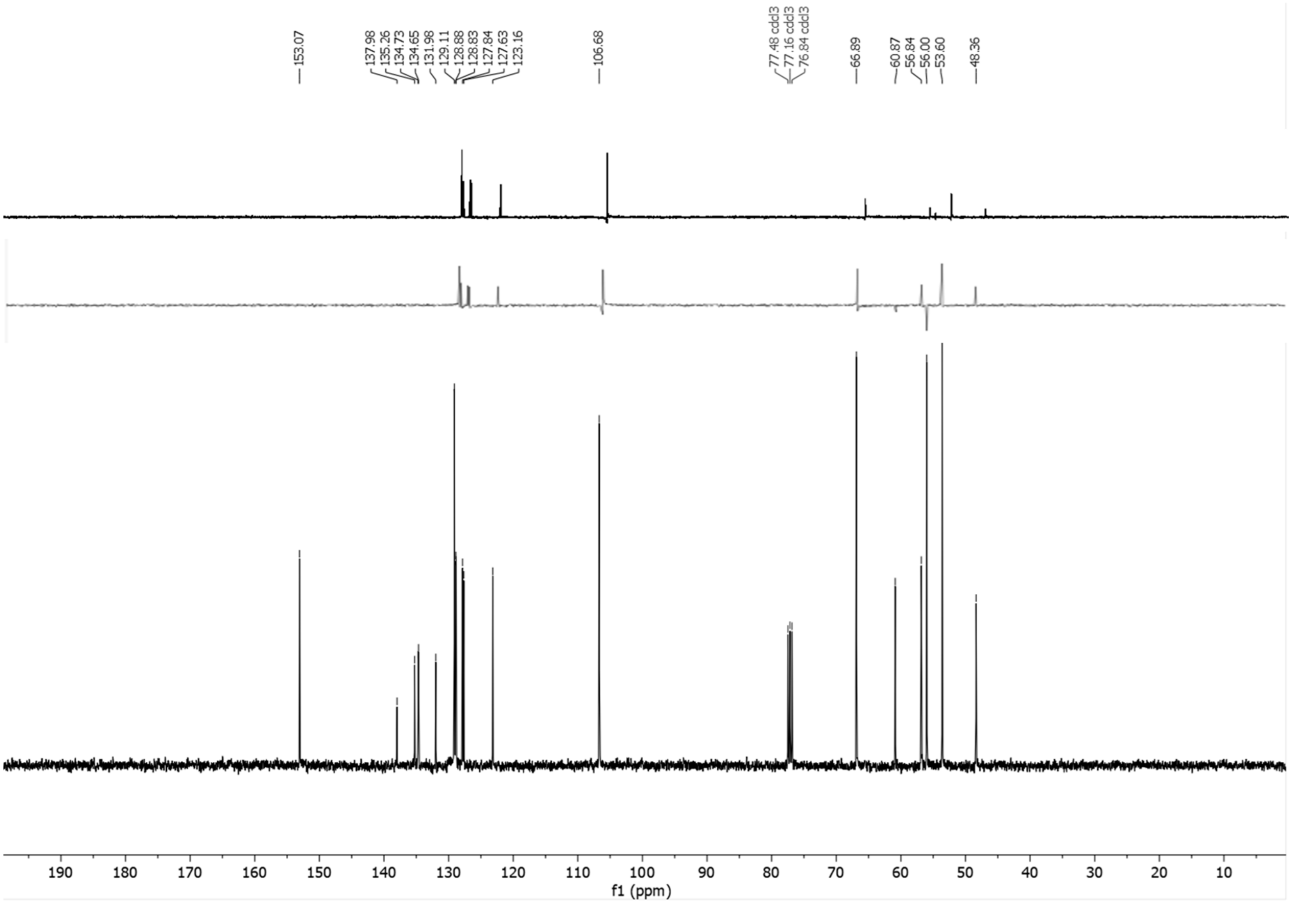
153.07
137.98 135.26 134.73 134.65 131.98 129.11 128.88 128.83 127.84 127.63 123.16
106.68
77.48 cdcl3 77.16 cdcl3 76.84 cdcl3
66.89
60.87 56.84 56.00 53.60
48.36
f1 (ppm)

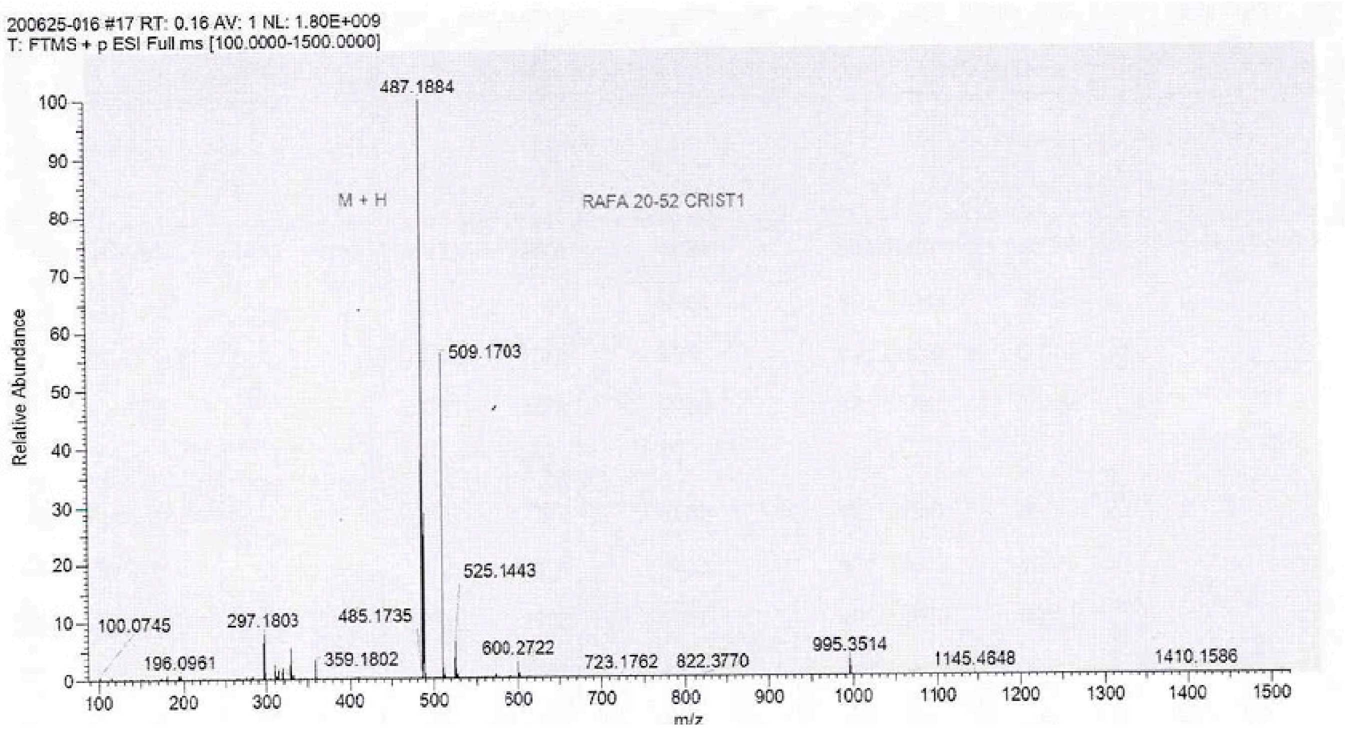

^{1}H, ^{13}C NMR and HRMS spectra of ***N*-(2-morpholinoethyl)-*N*-(3,4,5-trimethoxyphenyl)naphthalene-2-sulfonamide (10)**

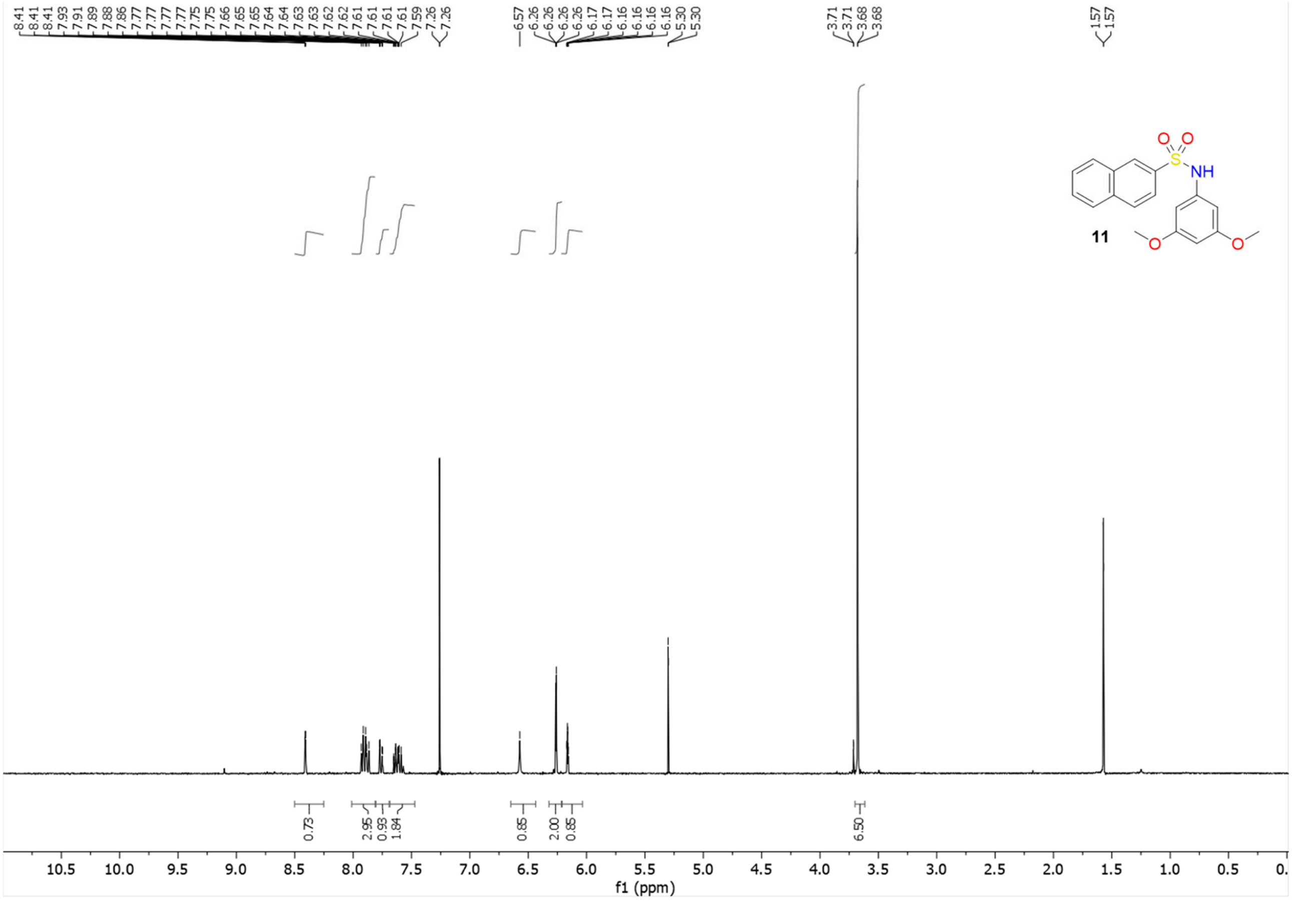
8.41 8.41 8.41 7.93 7.91 7.89 7.88 7.86 7.77 7.77 7.77 7.77 7.75 7.75 7.66 7.65 7.65 7.64 7.64 7.63 7.63 7.62 7.62 7.61 7.61 7.61 7.61 7.61 7.59 7.26 7.26
6.57 6.26 6.26 6.26 6.26 6.17 6.17 6.16 6.16 6.16 6.16 5.30 5.30
3.71 3.71 3.68 3.68
1.57 1.57
NH
O
S
11
0.73
2.95 0.93 1.84
0.85
2.00 0.85
6.50
10.5 10.0 9.5 9.0 8.5 8.0 7.5 7.0 6.5 6.0 5.5 5.0 4.5 4.0 3.5 3.0 2.5 2.0 1.5 1.0 0.5
f1 (ppm)

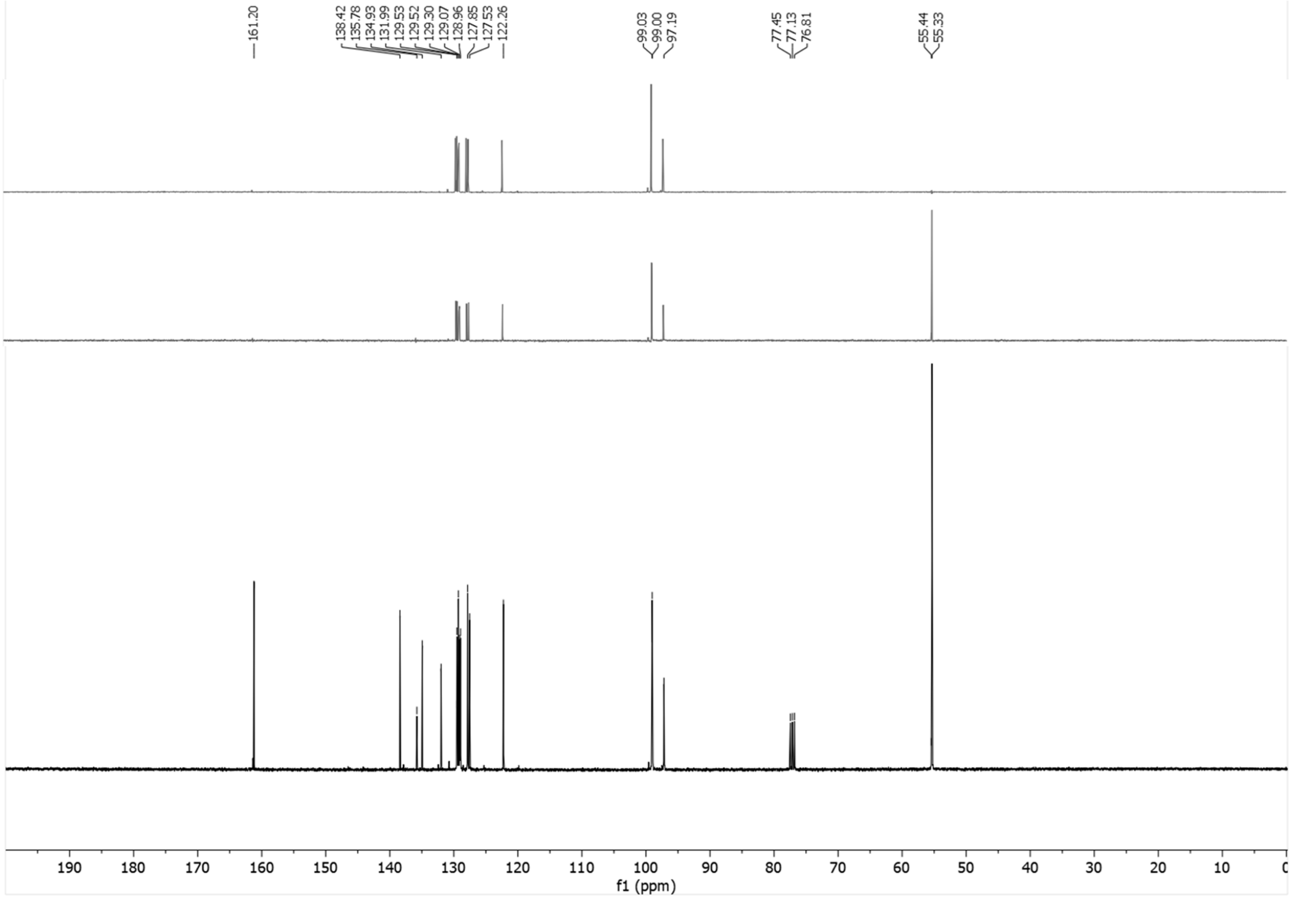
161.20
138.42 135.78 134.93 131.99 129.53 129.52 129.30 129.07 128.96 127.85 127.53 122.26
99.03 99.00 97.19
77.45 77.13 76.81
55.44 55.33
190 180 170 160 150 140 130 120 110 100 90 80 70 60 50 40 30 20 10
f1 (ppm)

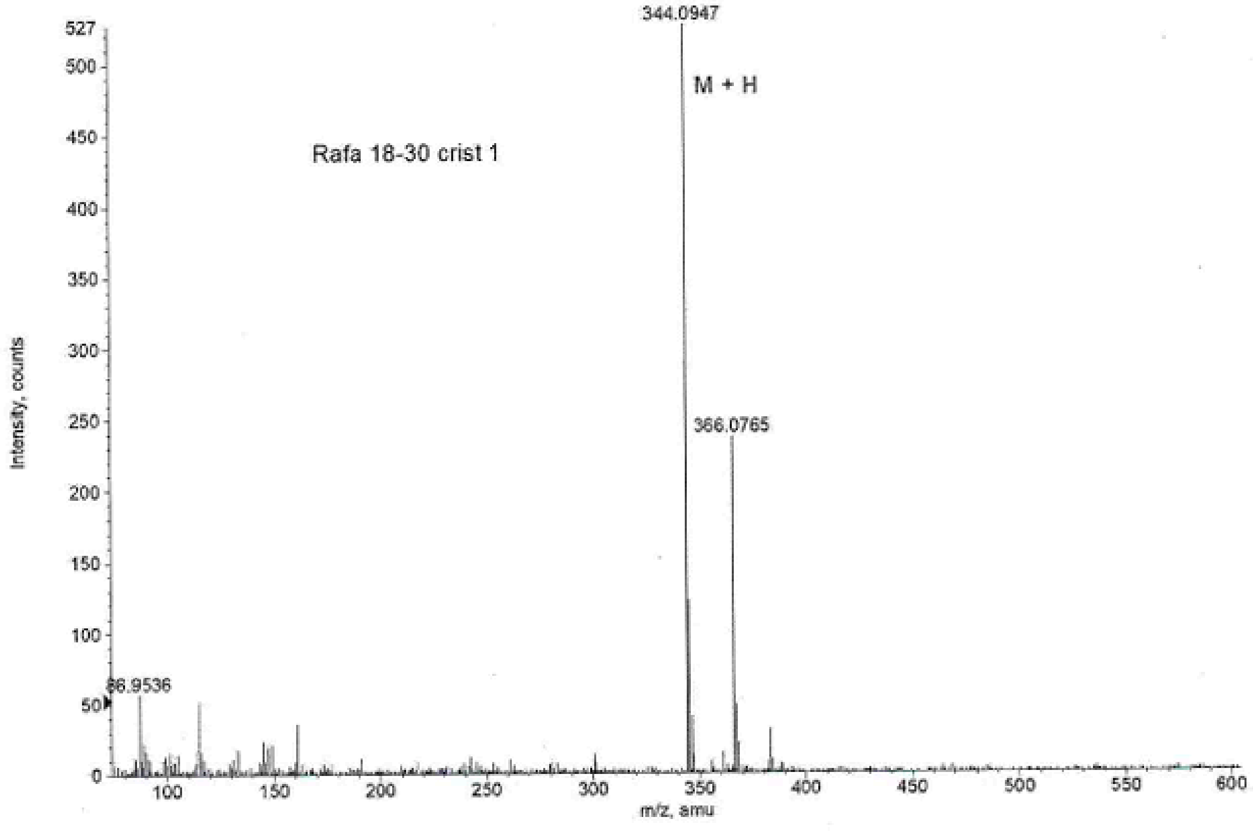

^{1}H, ^{13}C NMR and HRMS spectra of ***N*-(3,5-dimethoxyphenyl)naphthalene-2-sulfonamide (11)**

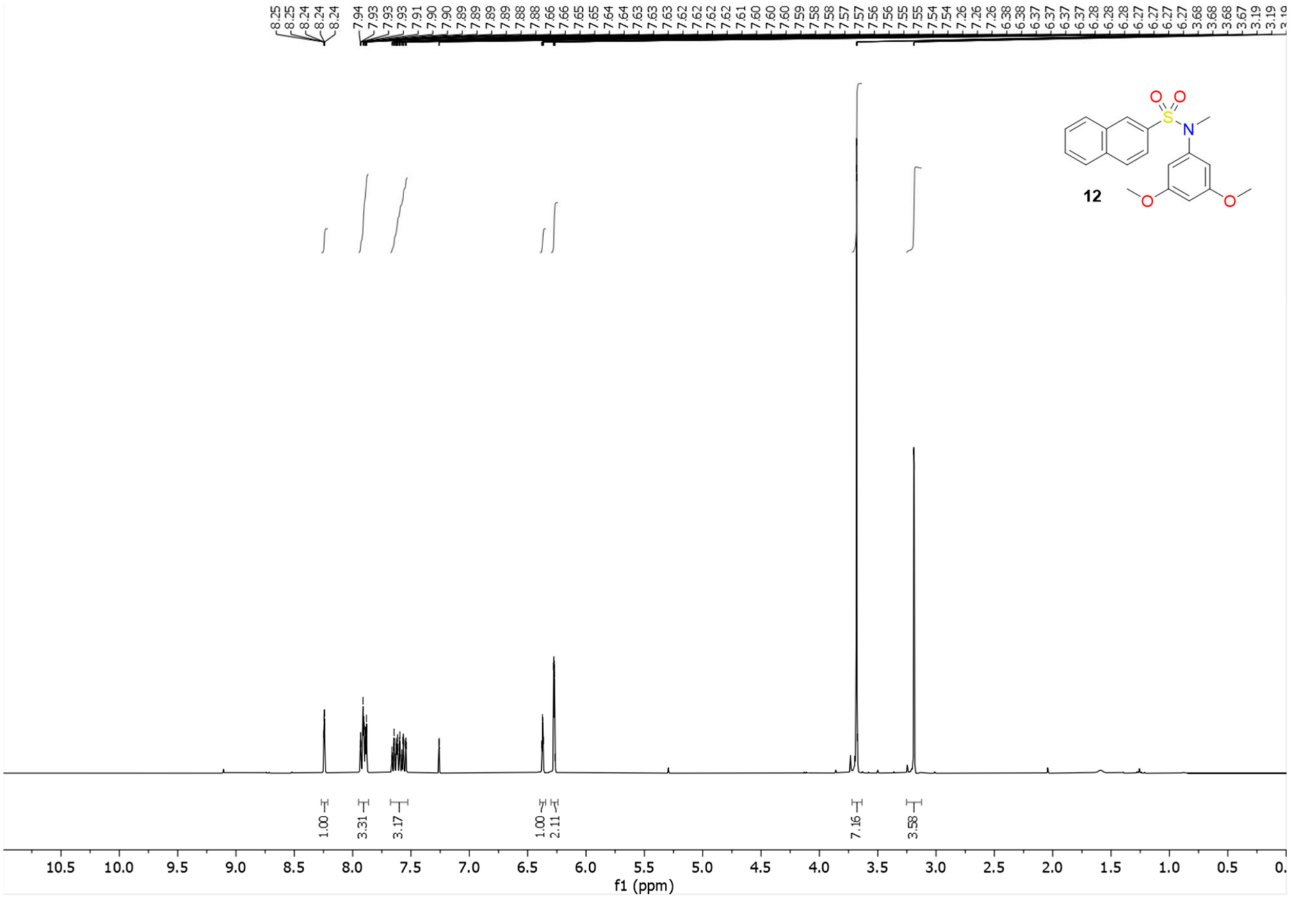
8.25
8.25
8.24
8.24
8.24
7.94
7.93
7.93
7.93
7.91
7.90
7.90
7.89
7.89
7.89
7.89
7.88
7.66
7.66
7.65
7.65
7.64
7.64
7.63
7.63
7.63
7.62
7.62
7.62
7.62
7.61
7.60
7.60
7.60
7.59
7.58
7.58
7.57
7.57
7.56
7.56
7.55
7.55
7.54
7.54
7.54
7.26
7.26
7.26
7.26
6.38
6.38
6.37
6.37
6.37
6.28
6.28
6.28
6.27
6.27
6.27
6.27
3.68
3.68
3.68
3.67
3.19
[illegible]
12
1.00
3.31
3.17
1.00
2.11
7.16
3.58
10.5
10.0
9.5
9.0
8.5
8.0
7.5
7.0
6.5
6.0
5.5
5.0
4.5
4.0
3.5
3.0
2.5
2.0
1.5
1.0
0.5
f1 (ppm)

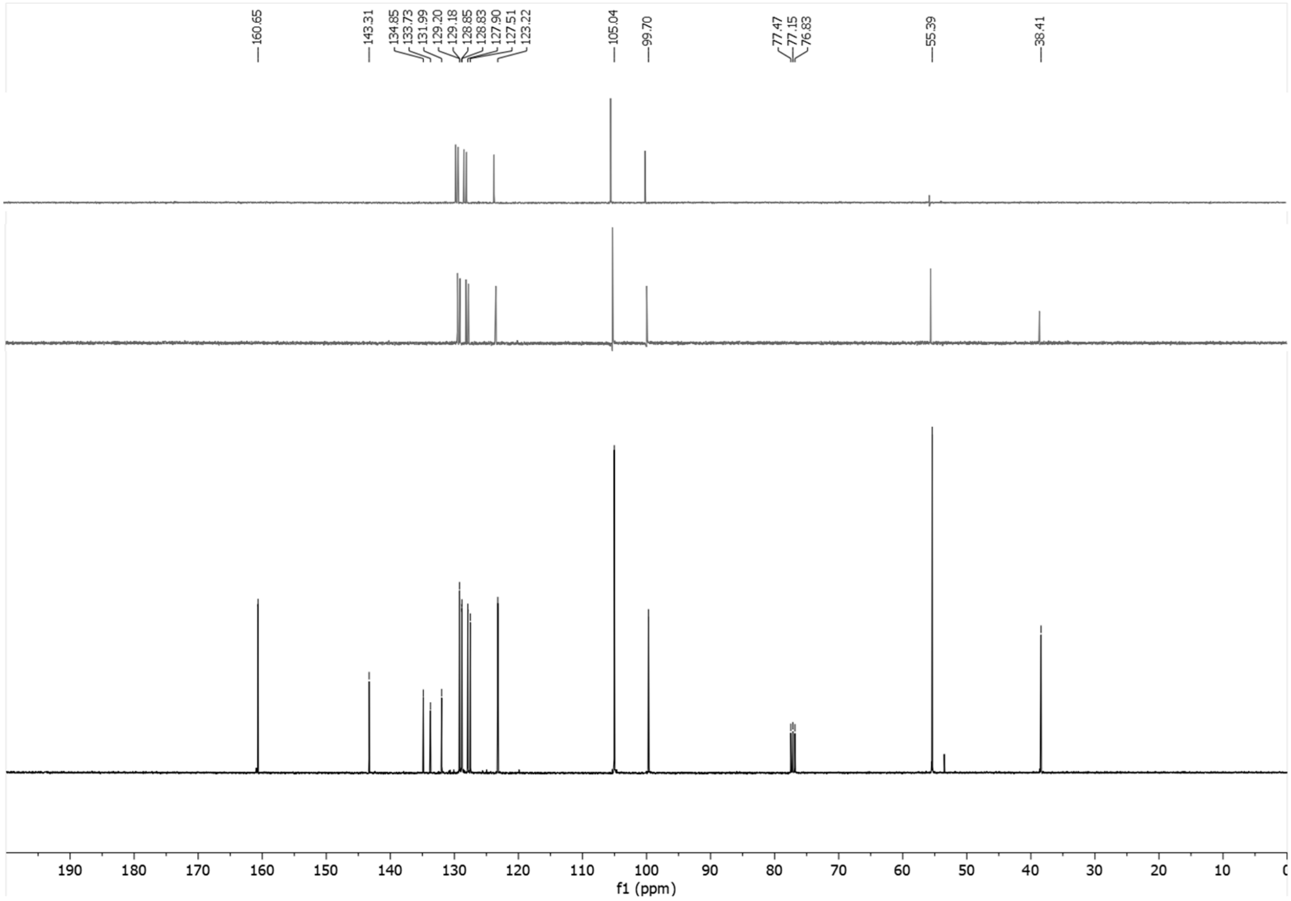
160.65
143.31
134.85
133.73
131.99
129.20
129.18
128.85
128.83
127.90
127.51
123.22
105.04
99.70
77.47
77.15
76.83
55.39
38.41
190
180
170
160
150
140
130
120
110
100
90
80
70
60
50
40
30
20
10
f1 (ppm)

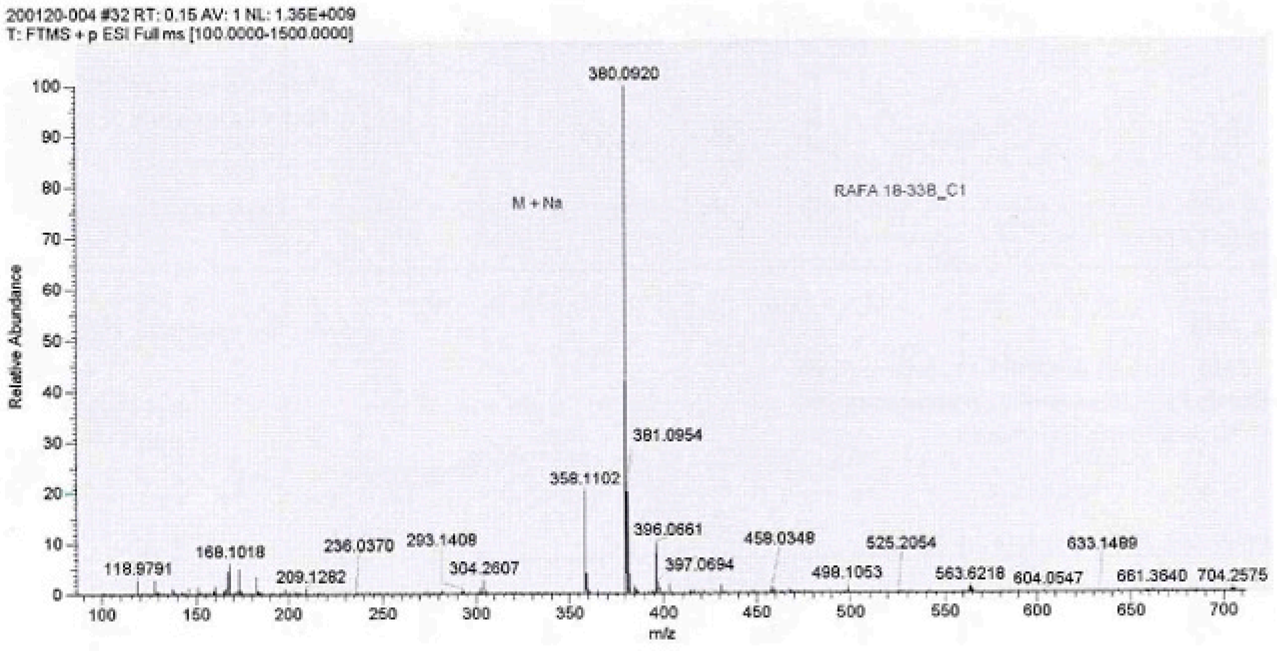

^{1}H, ^{13}C NMR and HRMS spectra of ***N*-(3,5-dimethoxyphenyl)-*N*-methylnaphthalene-2-sulfonamide (12)**

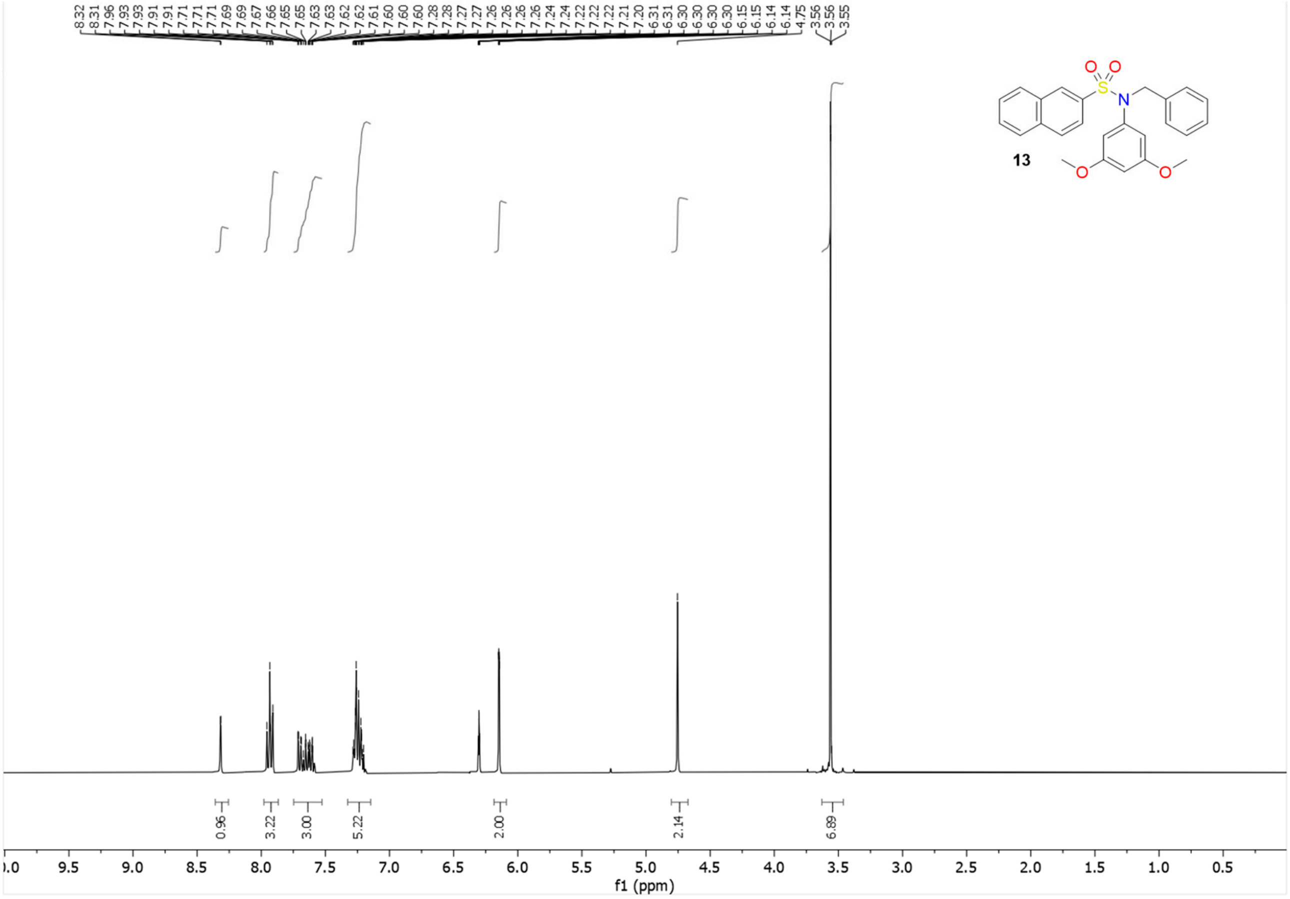
8.32
8.31
7.96
7.93
7.93
7.91
7.91
7.71
7.71
7.71
7.69
7.69
7.67
7.66
7.65
7.65
7.63
7.63
7.62
7.62
7.61
7.60
7.60
7.60
7.28
7.28
7.27
7.27
7.26
7.26
7.26
7.26
7.24
7.24
7.22
7.22
7.22
7.21
7.20
6.31
6.31
6.30
6.30
6.30
6.30
6.15
6.15
6.14
4.75
3.56
3.56
3.55
13
0.96
3.22
3.00
5.22
2.00
2.14
6.89
f1 (ppm)

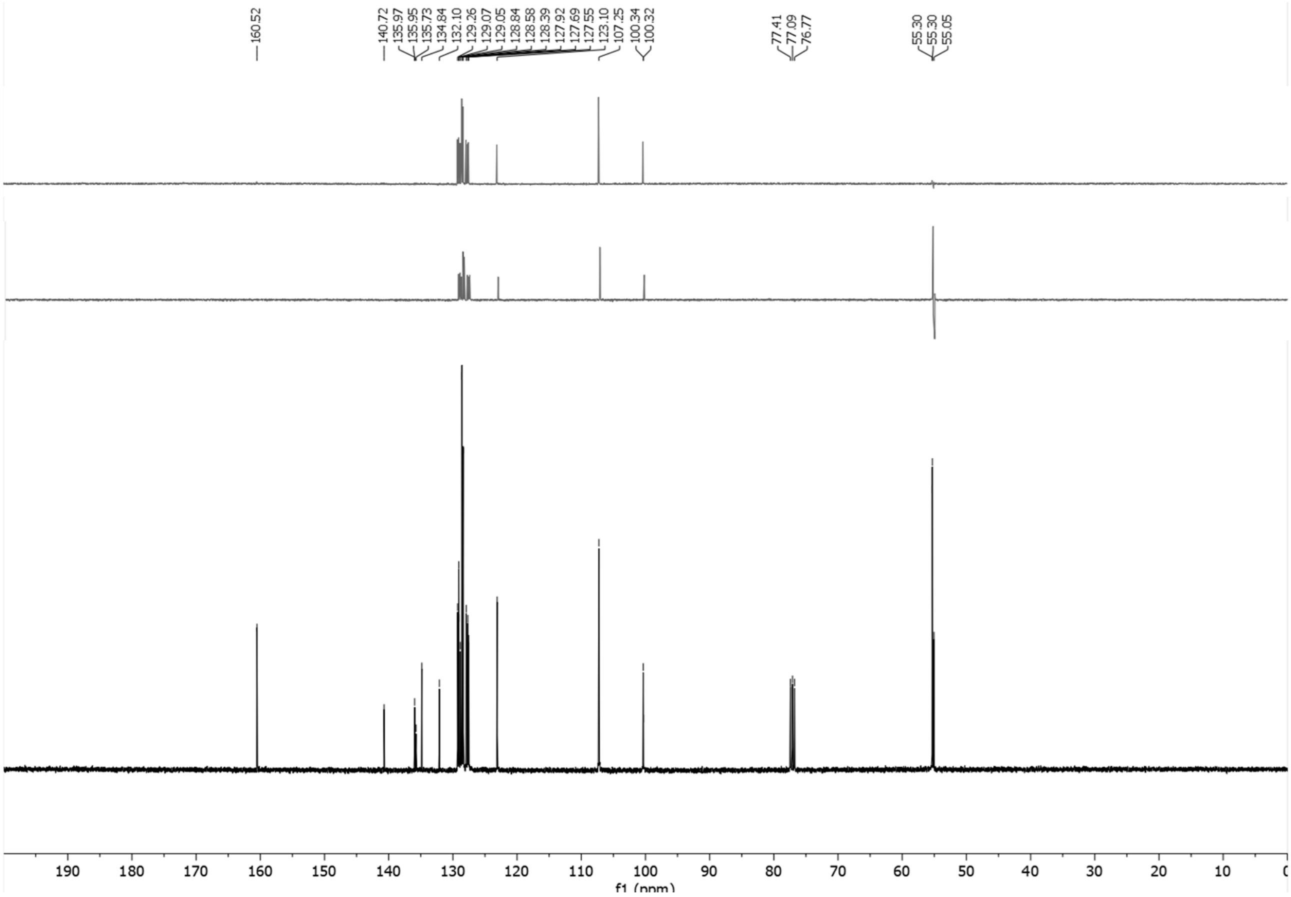
160.52
140.72
135.97
135.95
135.73
134.84
132.10
129.26
129.07
129.05
128.84
128.58
128.39
127.92
127.69
127.55
123.10
107.25
100.34
100.32
77.41
77.09
76.77
55.30
55.30
55.05
f1 (ppm)

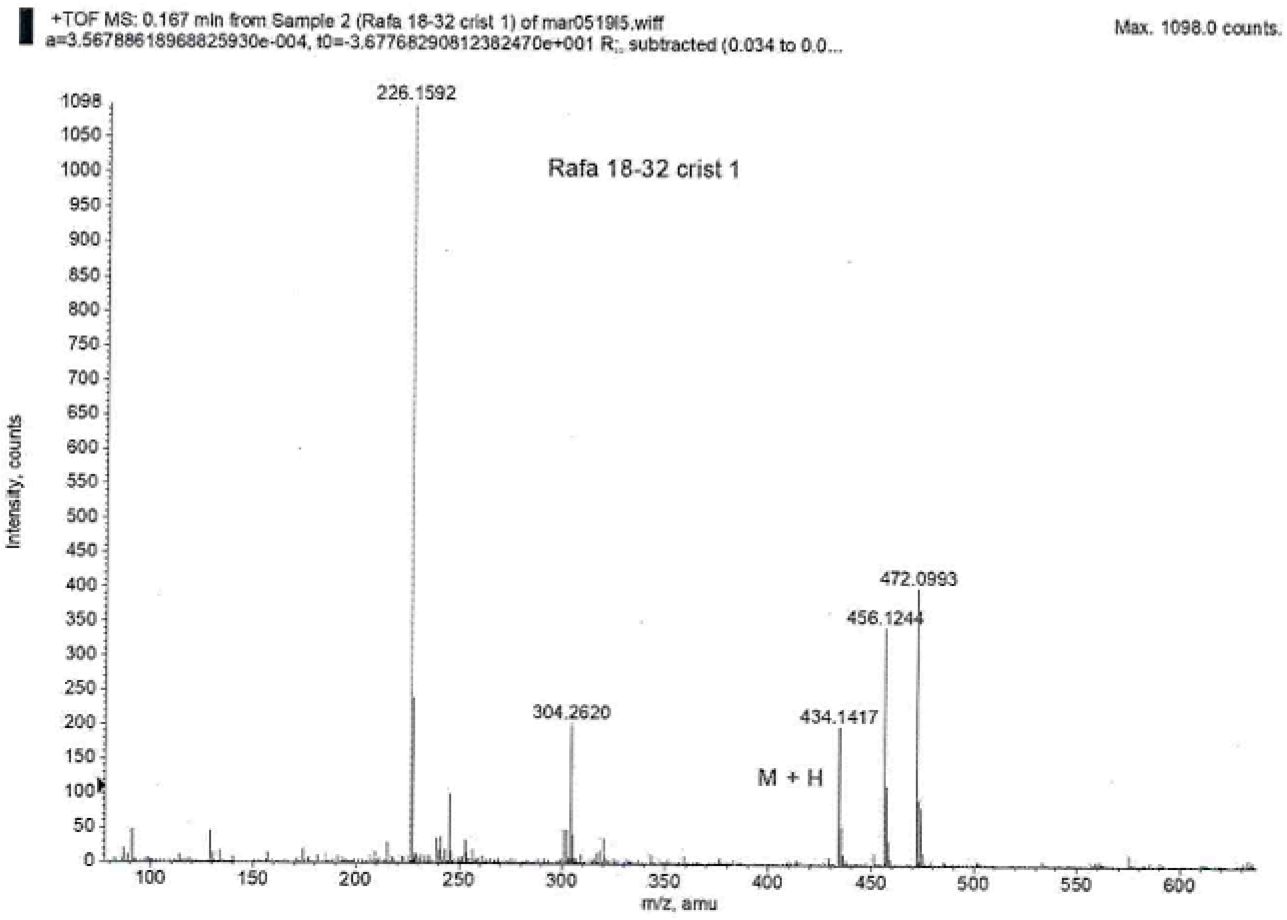

^{1}H, ^{13}C NMR and HRMS spectra of ***N*-benzyl-*N*-(3,5-dimethoxyphenyl)naphthalene-2-sulfonamide (13)**

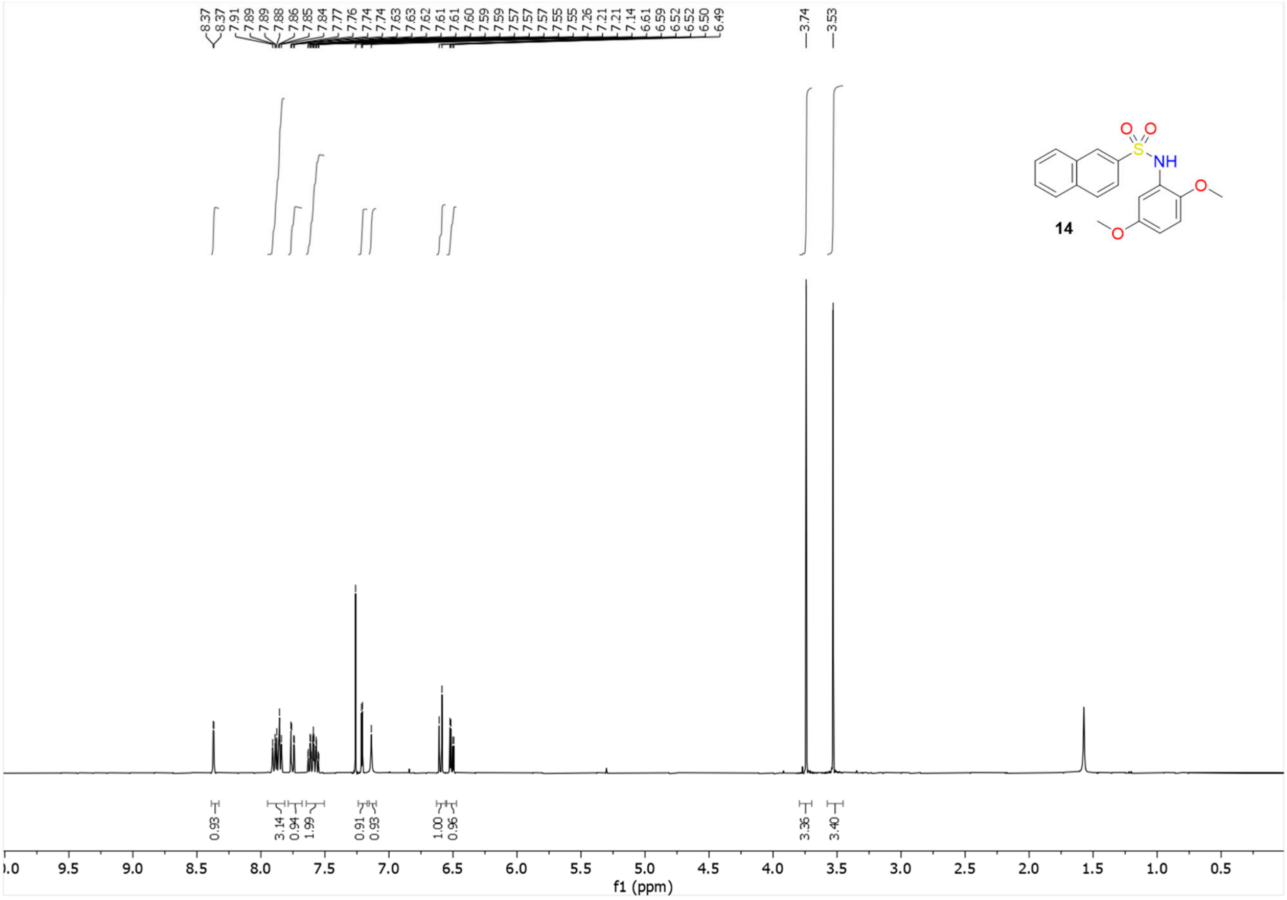
8.37
8.37
7.91
7.89
7.89
7.88
7.86
7.85
7.84
7.77
7.76
7.74
7.74
7.63
7.63
7.62
7.61
7.61
7.60
7.59
7.59
7.57
7.57
7.57
7.55
7.55
7.26
7.21
7.21
7.14
6.61
6.59
6.52
6.52
6.50
6.49
3.74
3.53
O
O
S
NH
O
O
14
0.93
3.14
0.94
1.99
0.91
0.93
1.00
0.96
3.36
3.40
9.5
9.0
8.5
8.0
7.5
7.0
6.5
6.0
5.5
5.0
4.5
4.0
3.5
3.0
2.5
2.0
1.5
1.0
0.5
f1 (ppm)

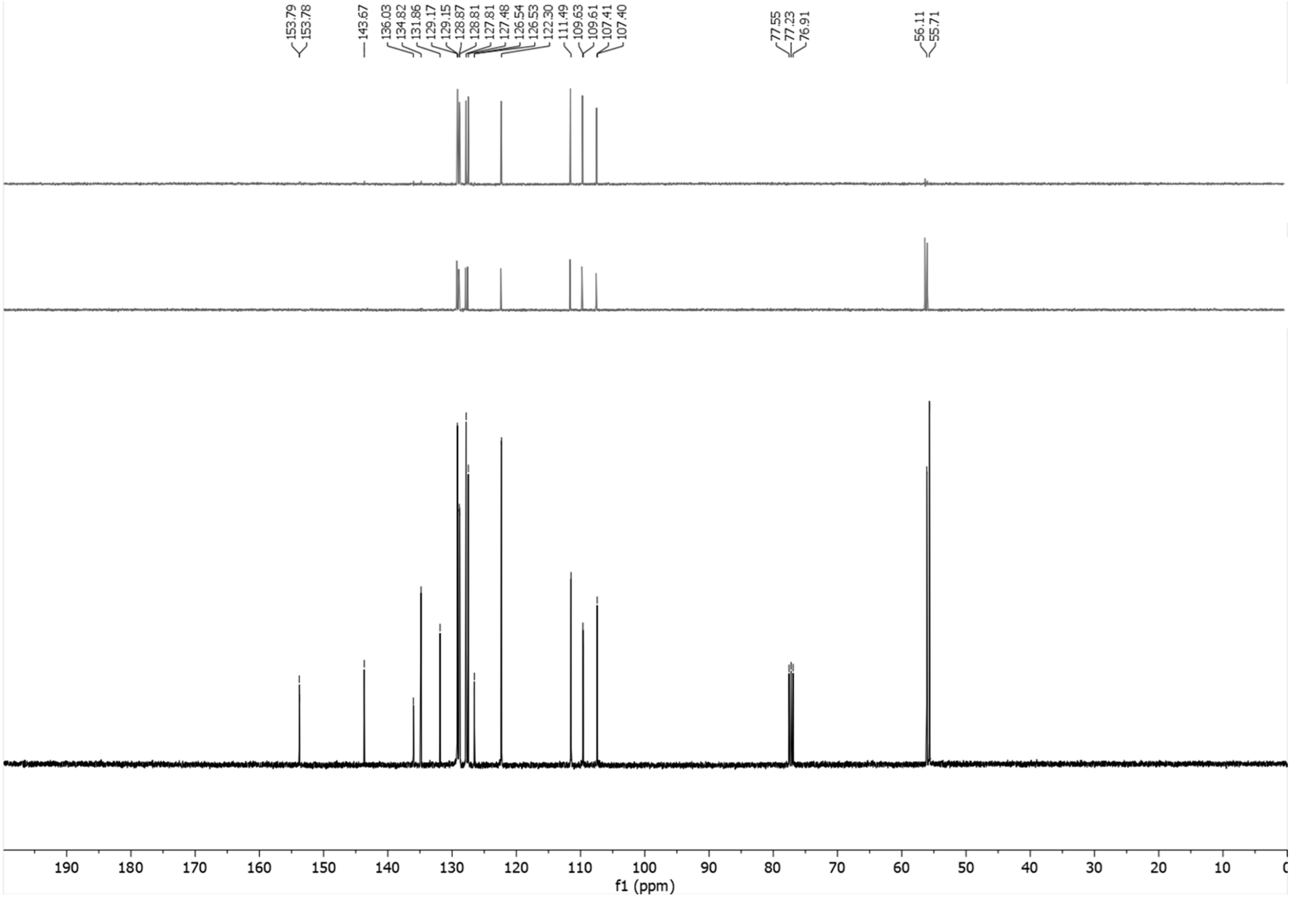
153.79
153.78
143.67
136.03
134.82
131.86
129.17
129.15
128.87
128.81
127.81
127.48
126.54
126.53
122.30
111.49
109.63
109.61
107.41
107.40
77.55
77.23
76.91
56.11
55.71
190
180
170
160
150
140
130
120
110
100
90
80
70
60
50
40
30
20
10
f1 (ppm)

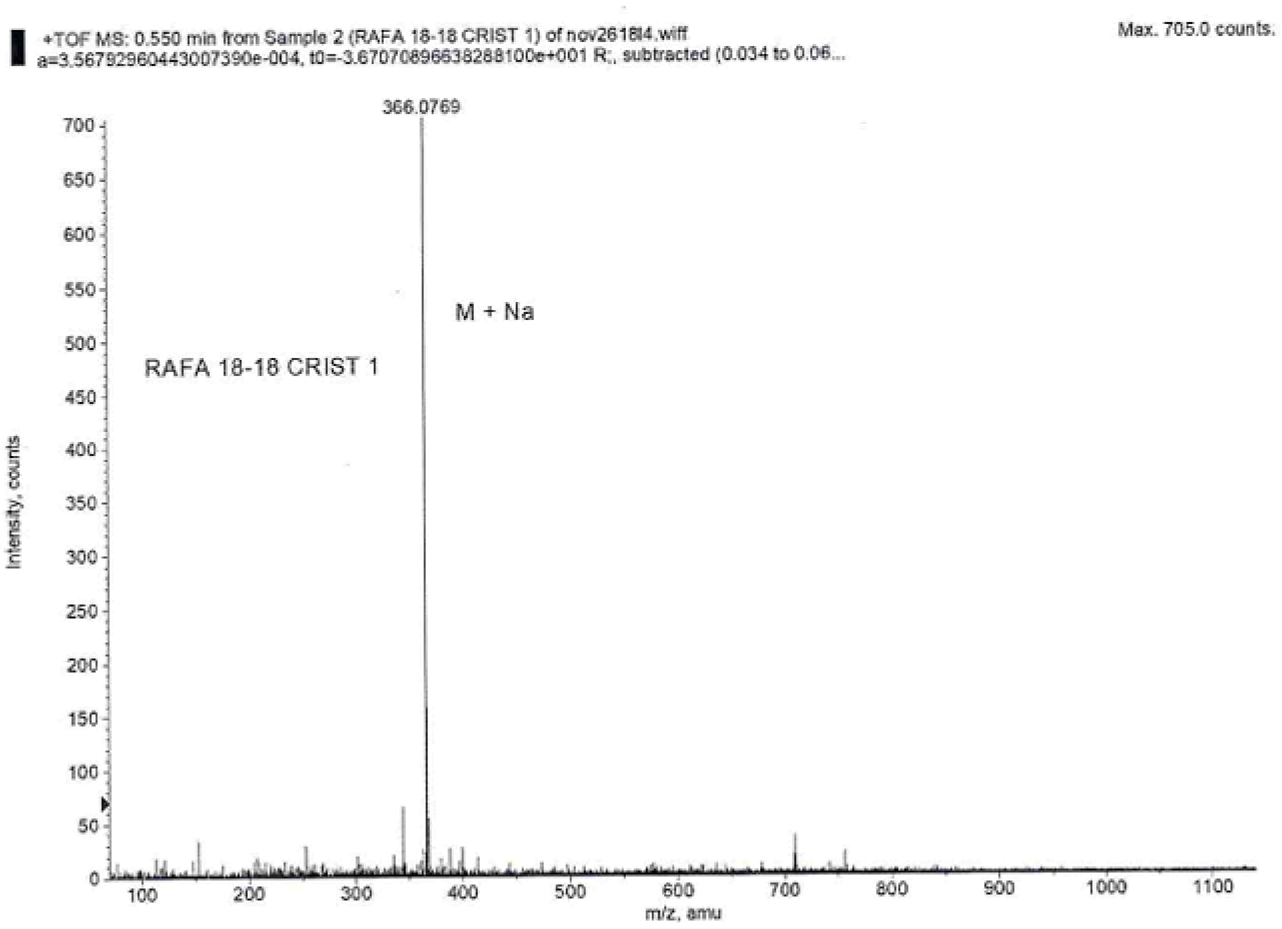

^{1}H, ^{13}C NMR and HRMS spectra of ***N*-(2,5-dimethoxyphenyl)naphthalene-2-sulfonamide (14)**

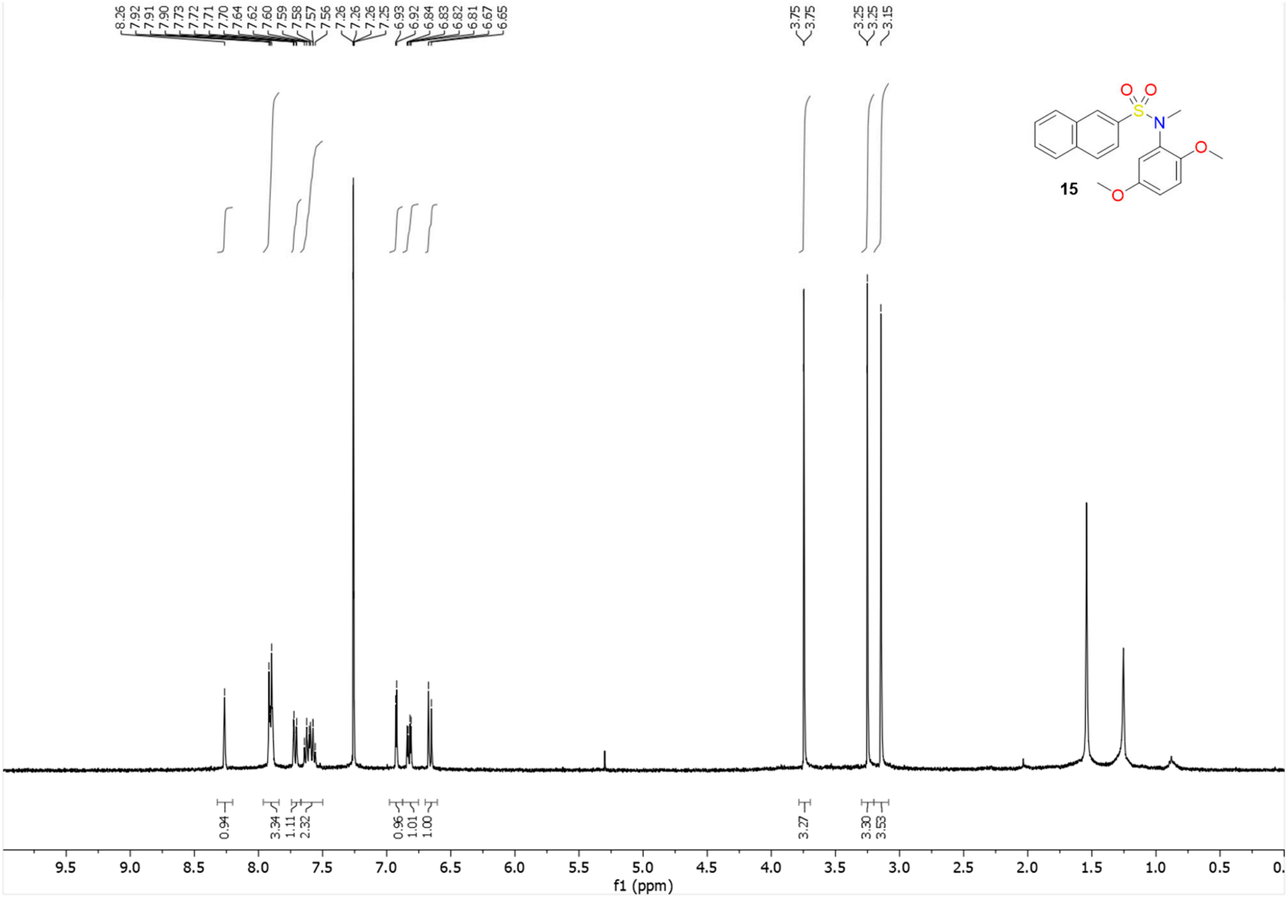
8.26 7.92 7.91 7.90 7.73 7.72 7.71 7.70 7.64 7.62 7.60 7.59 7.58 7.57 7.56 7.26 7.26 7.26 7.25 6.93 6.92 6.84 6.83 6.82 6.81 6.67 6.65
3.75 3.75
3.25 3.25 3.15
15
0.94 3.34 1.11 2.32 0.96 1.01 1.00 3.27 3.30 3.53
f1 (ppm)

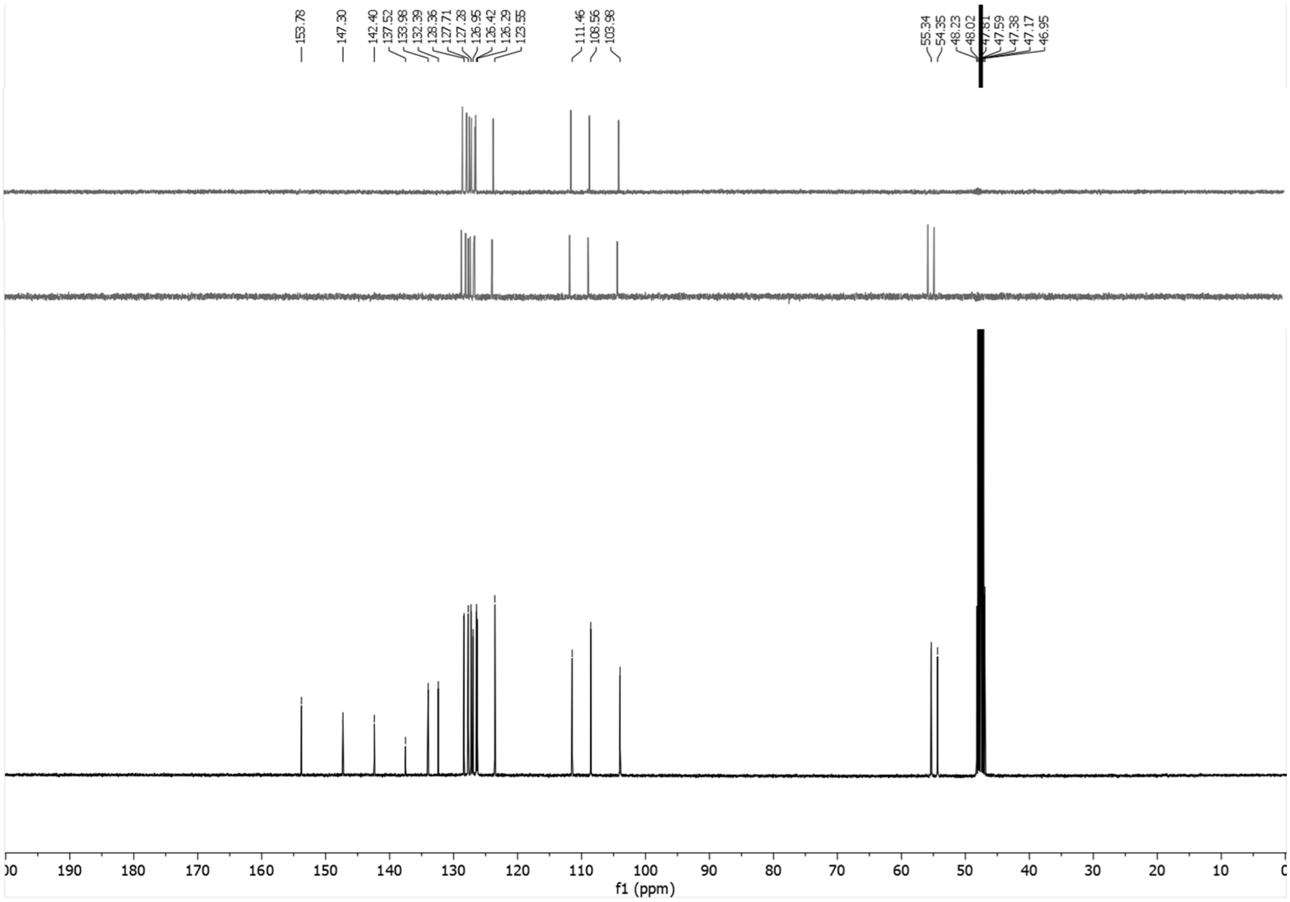
153.78 147.30 142.40 137.52 133.98 132.39 128.36 127.71 127.28 126.95 126.42 126.29 123.55 111.46 108.56 103.98
55.34 54.35 48.23 48.02 47.81 47.59 47.38 47.17 46.95
f1 (ppm)

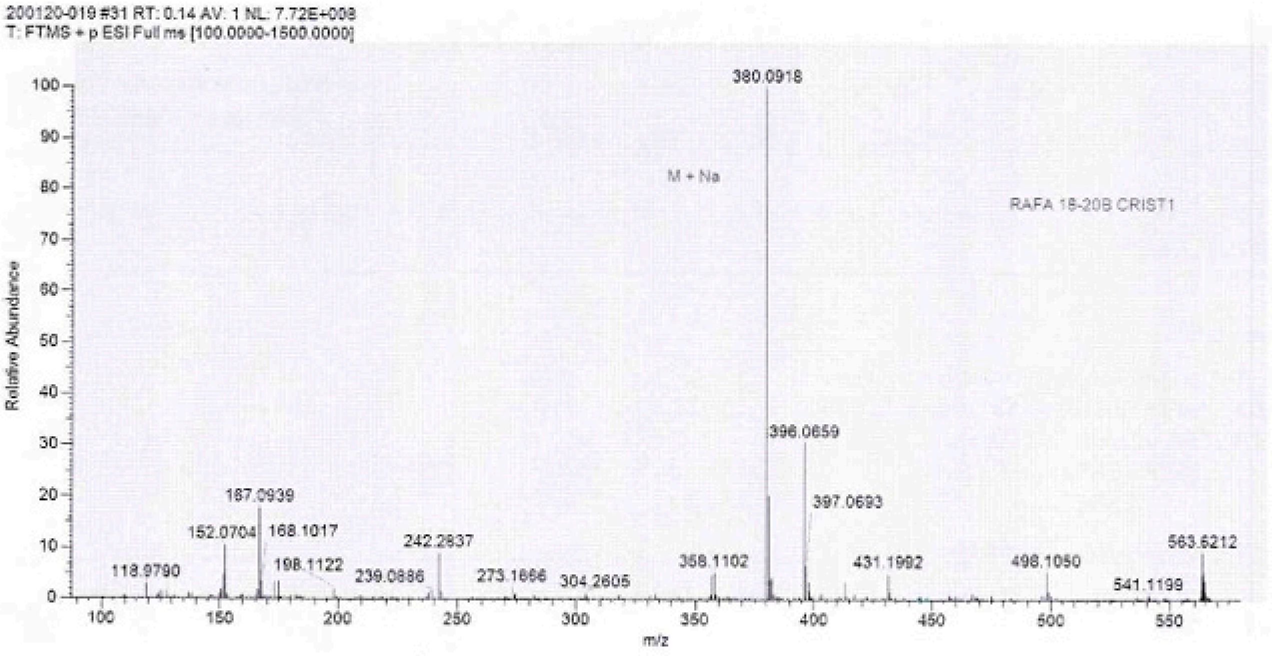

^{1}H, ^{13}C NMR and HRMS spectra of ***N*-(2,5-dimethoxyphenyl)-*N*-methylnaphthalene-2-sulfonamide (15)**

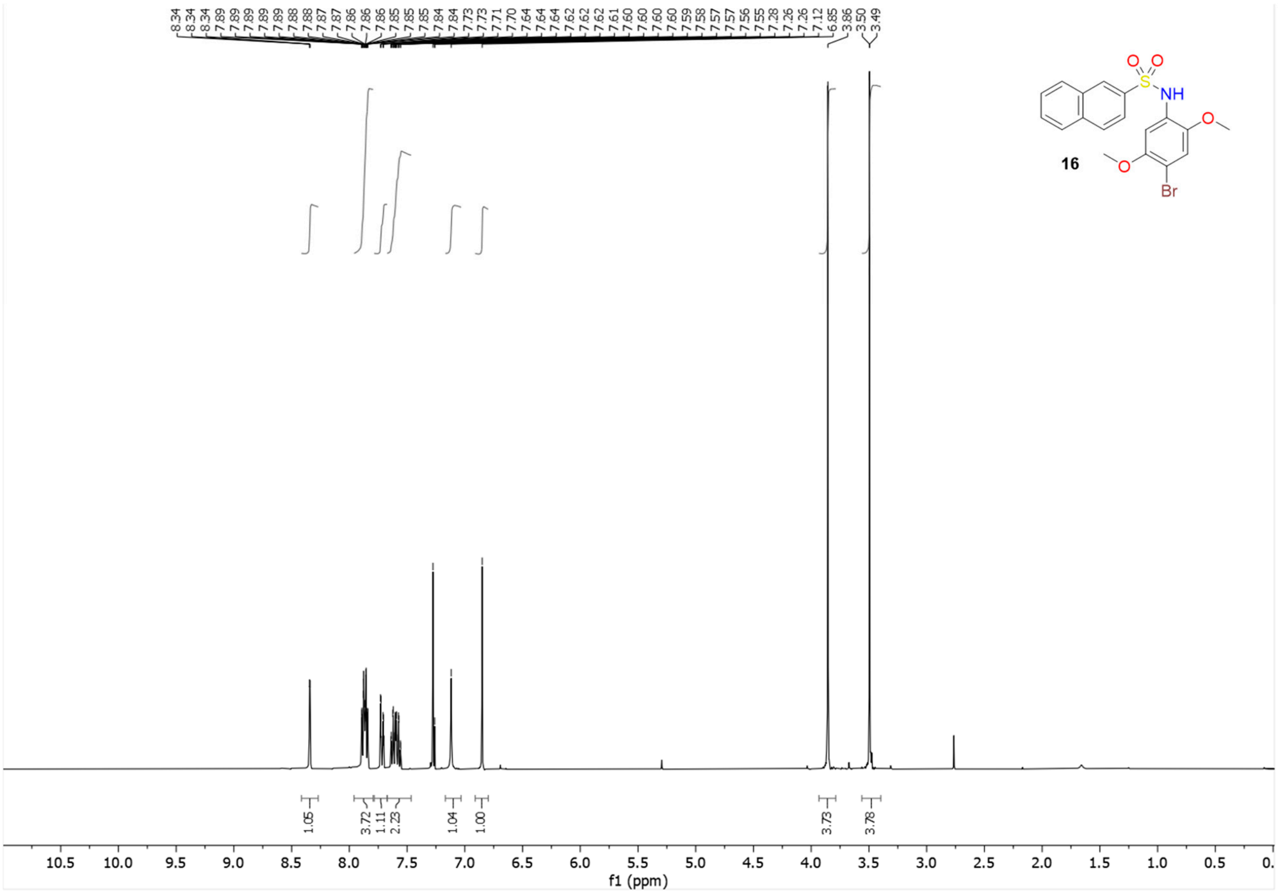

16
NH
Br
f1 (ppm)

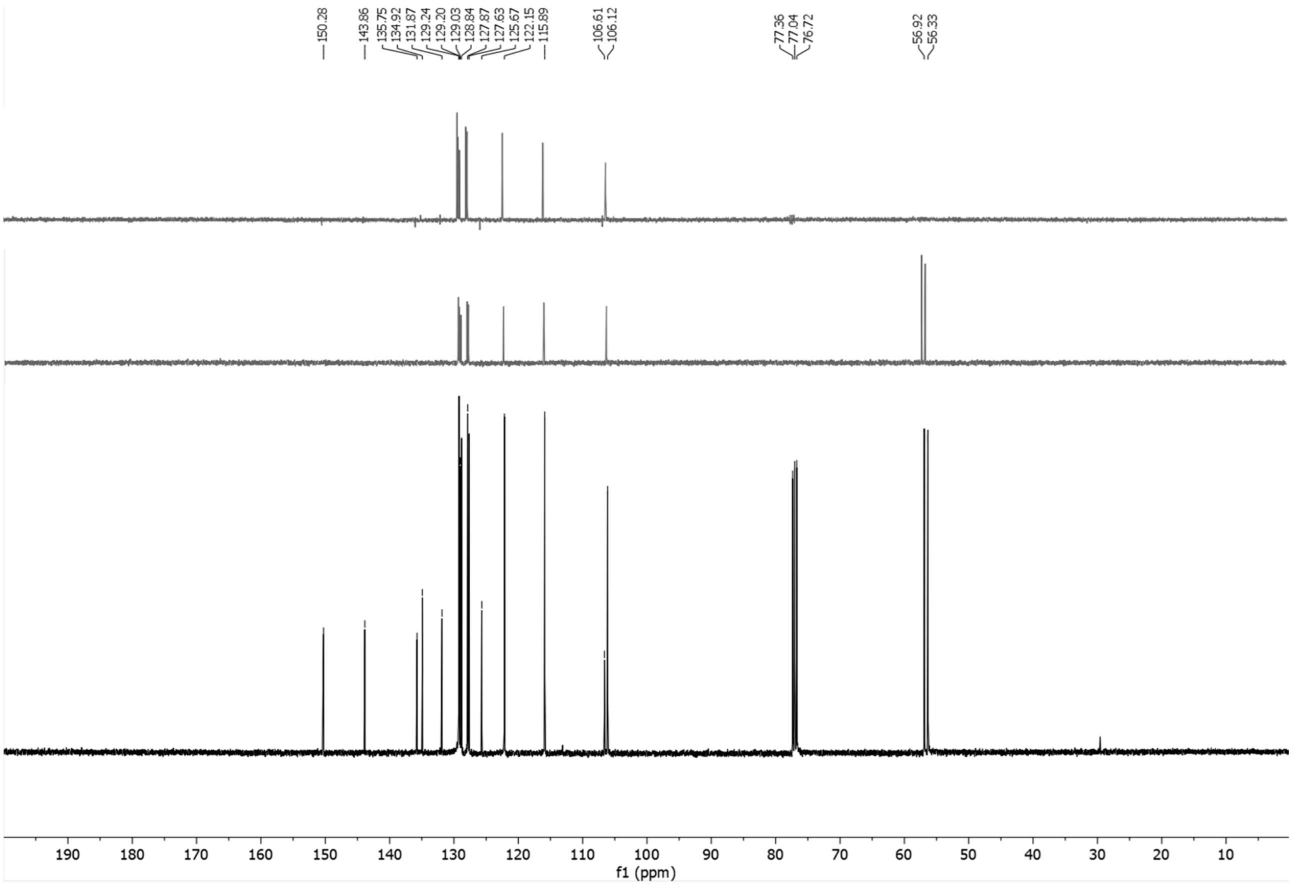

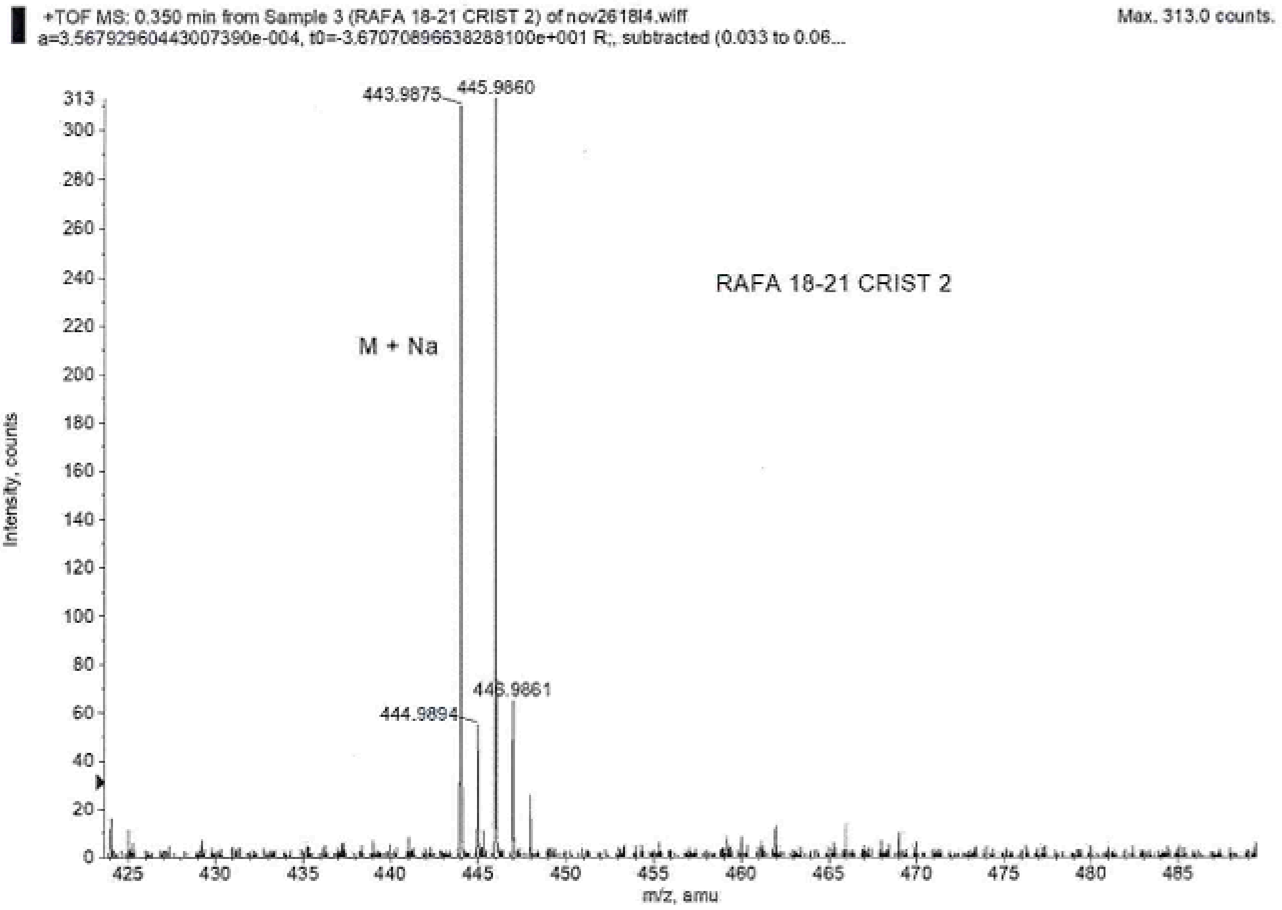

^{1}H, ^{13}C NMR and HRMS spectra of ***N*-(4-bromo-2,5-dimethoxyphenyl)naphthalene-2-sulfonamide (16)**

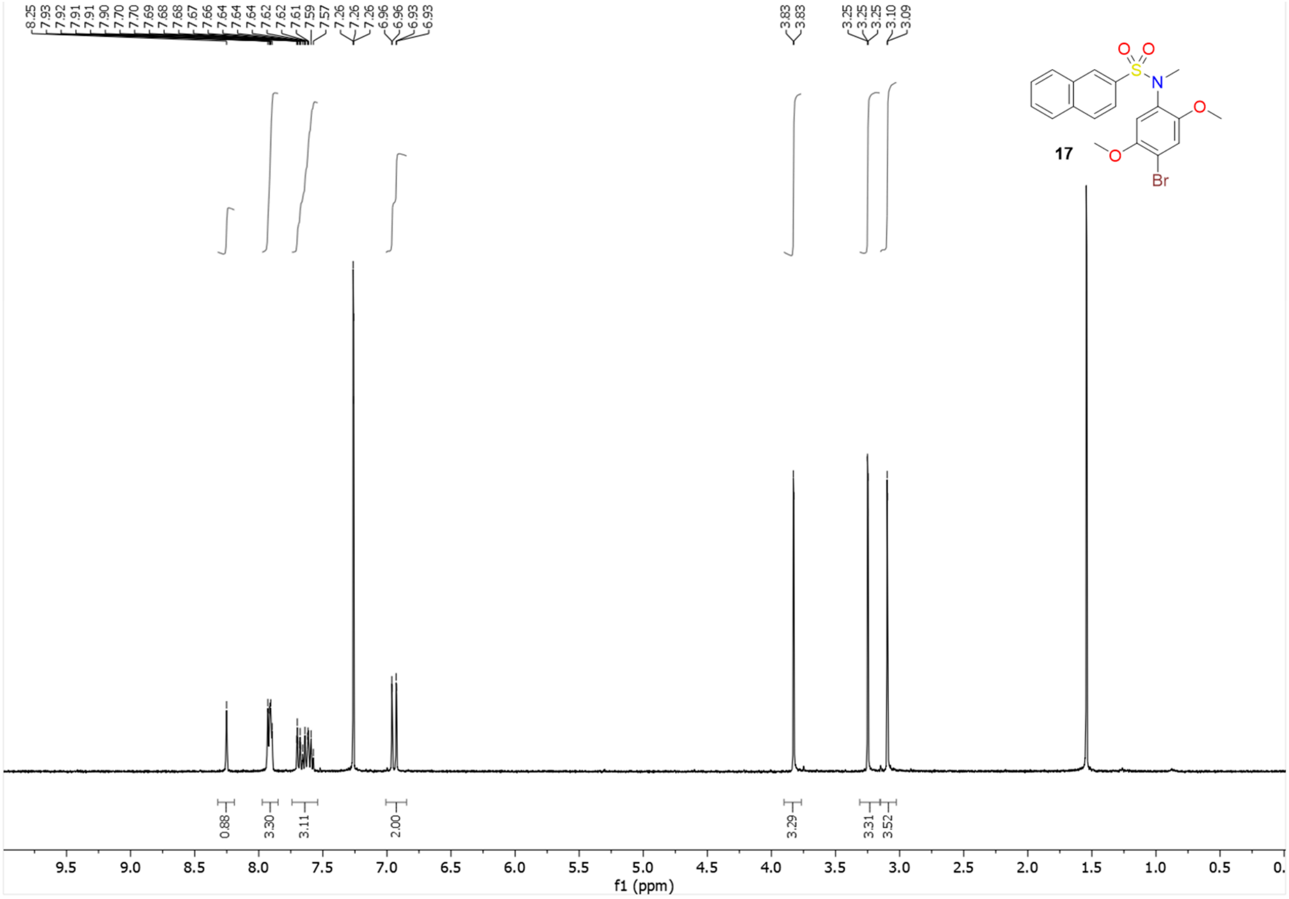
8.25 7.93 7.92 7.91 7.91 7.90 7.70 7.70 7.69 7.68 7.68 7.67 7.66 7.64 7.64 7.64 7.62 7.62 7.61 7.59 7.57 7.26 7.26 7.26 6.96 6.96 6.93 6.93
3.83 3.83
3.25 3.25 3.25 3.10 3.09
17
Br
0.88 3.30 3.11 2.00 3.29 3.31 3.52
f1 (ppm)

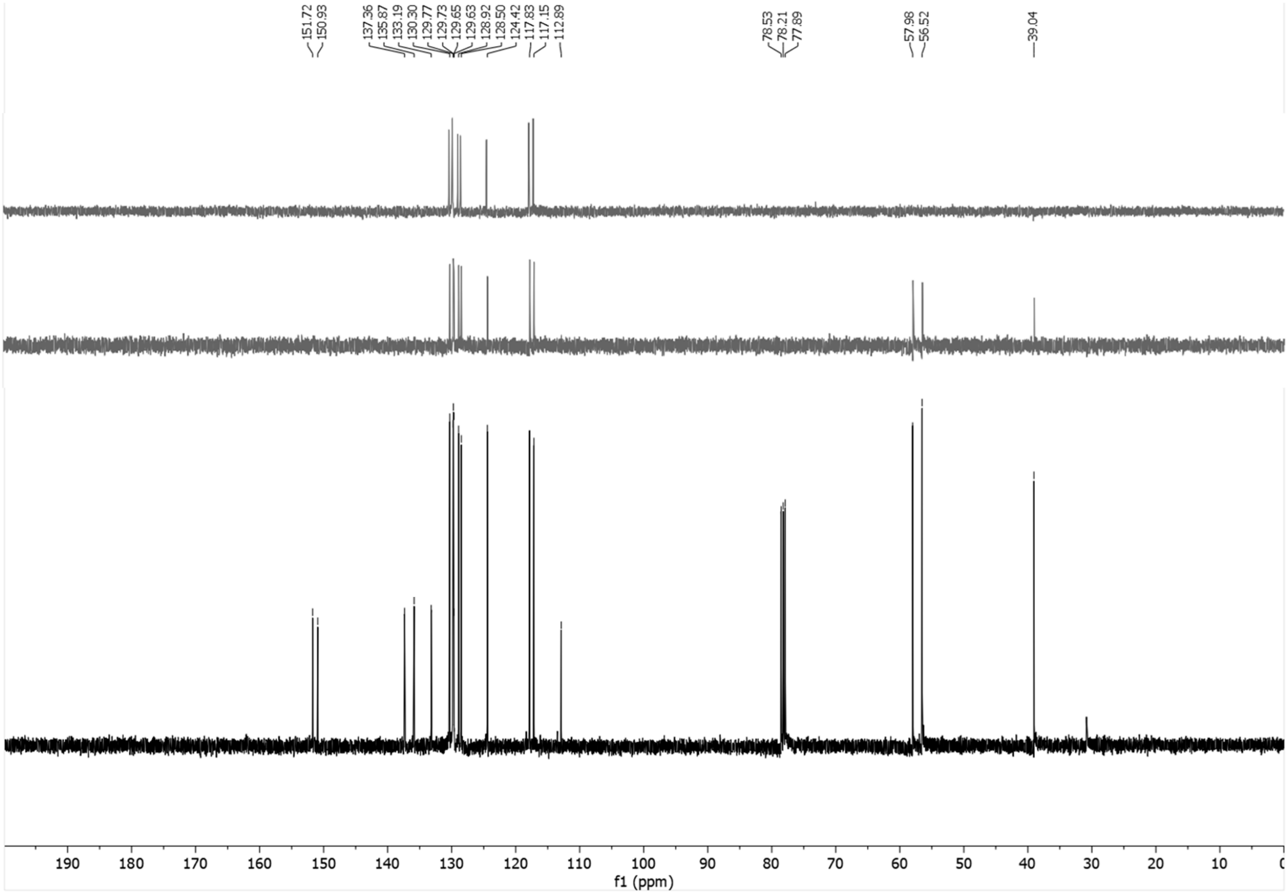
151.72 150.93
137.36 135.87 133.19 130.30 129.77 129.73 129.65 129.63 128.92 128.50 124.42 117.83 117.15 112.89
78.53 78.21 77.89
57.98 56.52
39.04
f1 (ppm)

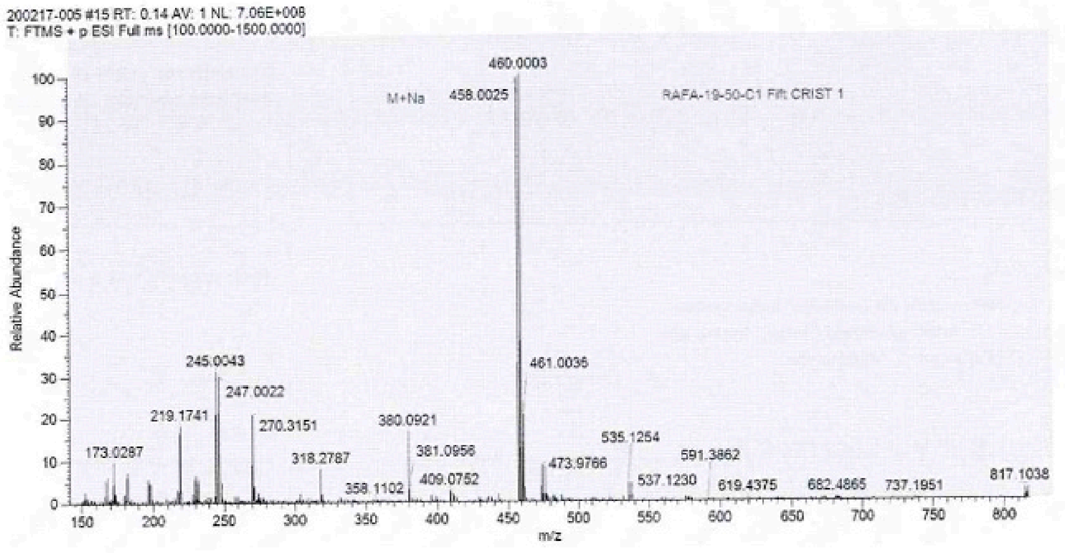

^{1}H, ^{13}C NMR and HRMS spectra of ***N*-(4-bromo-2,5-dimethoxyphenyl)-*N*-methylnaphthalene-2-sulfonamide (17)**

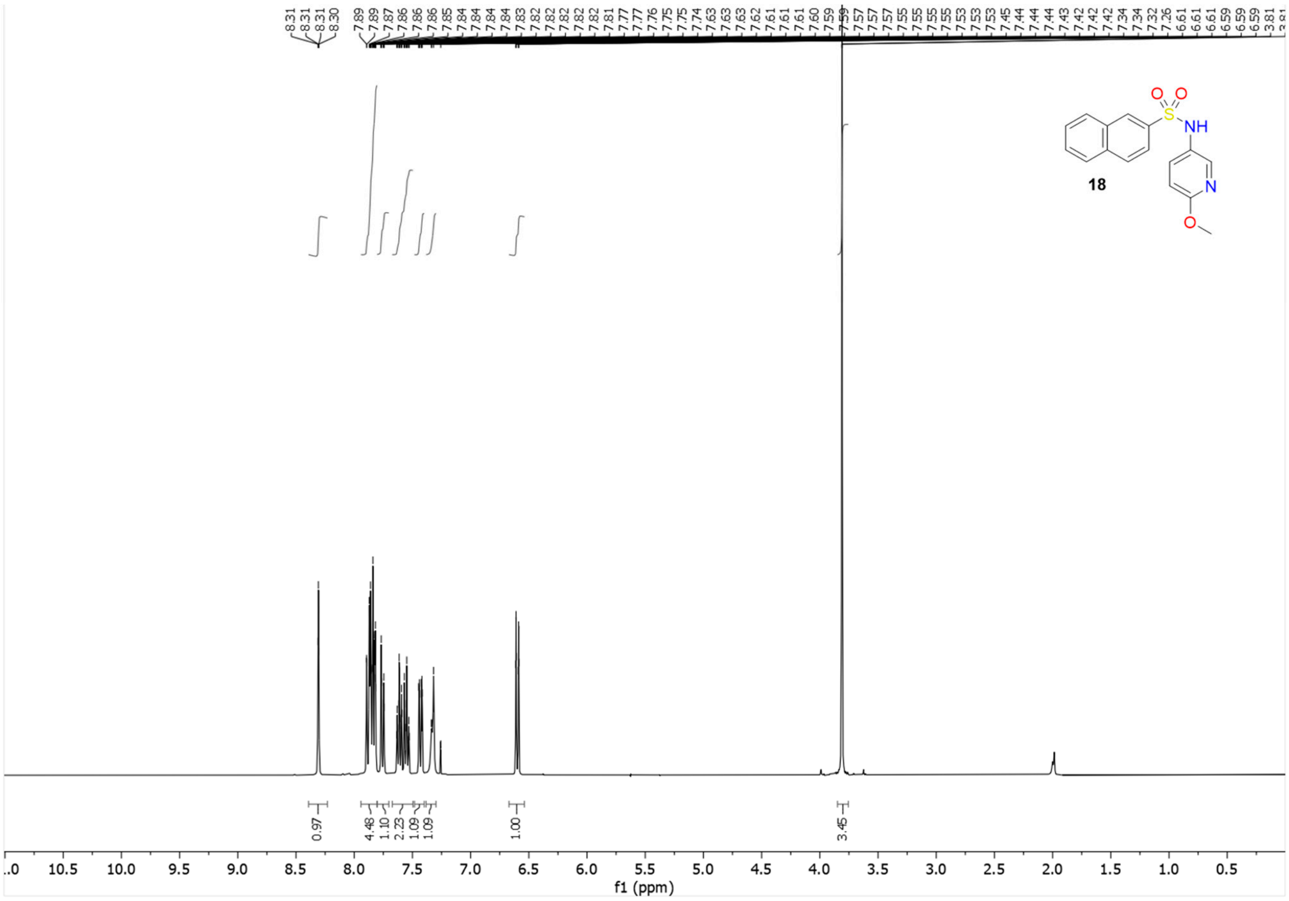

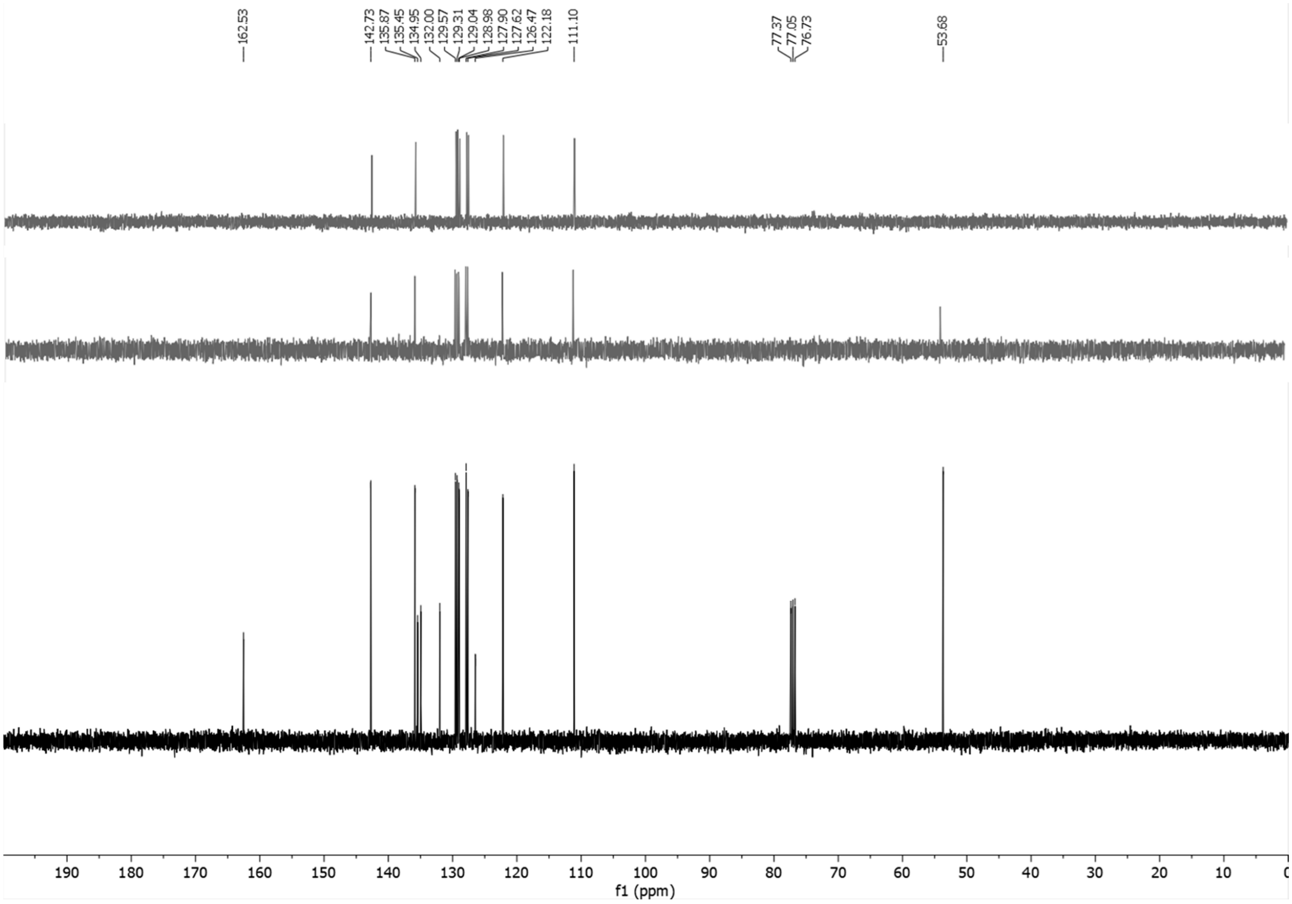

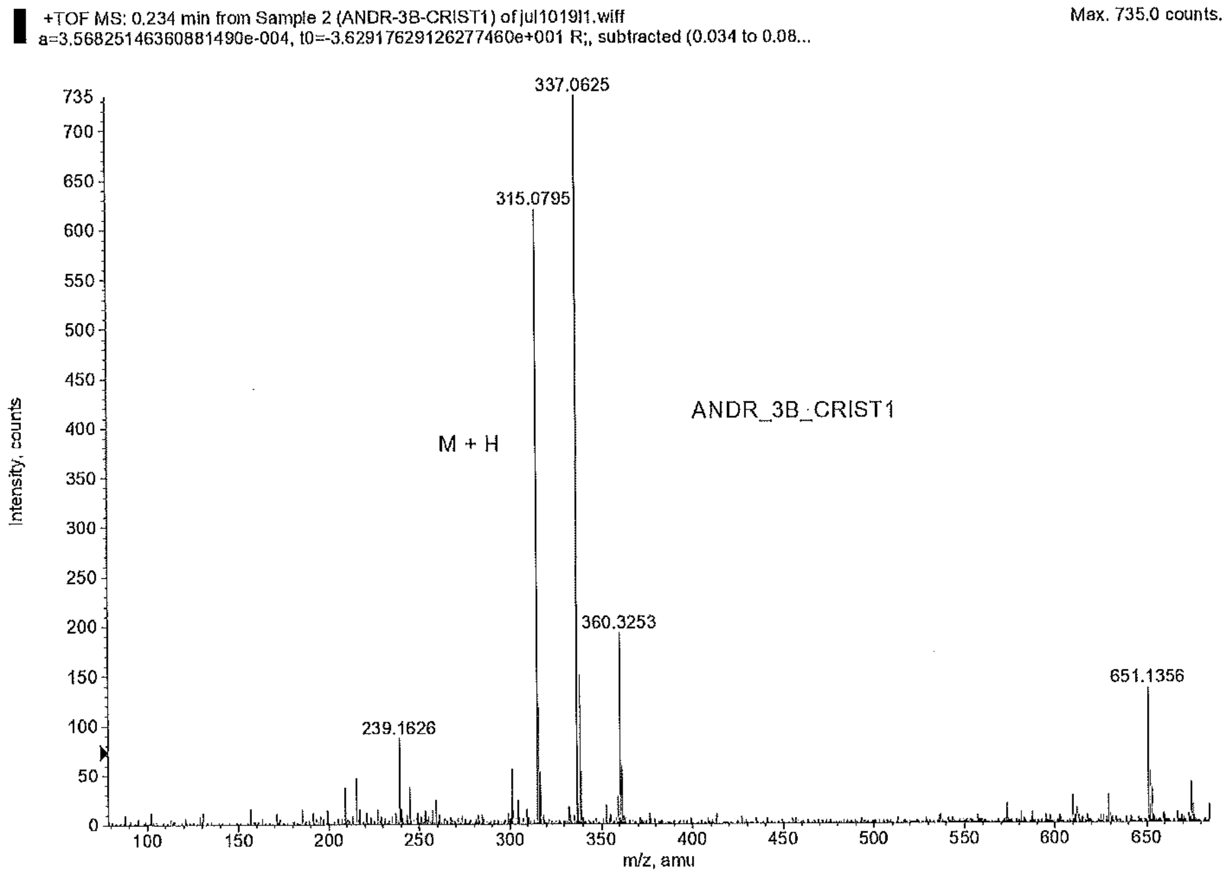

^{1}H, ^{13}C NMR and HRMS spectra of ***N*-(6-methoxypyridin-3-yl)naphthalene-2-sulfonamide (18)**

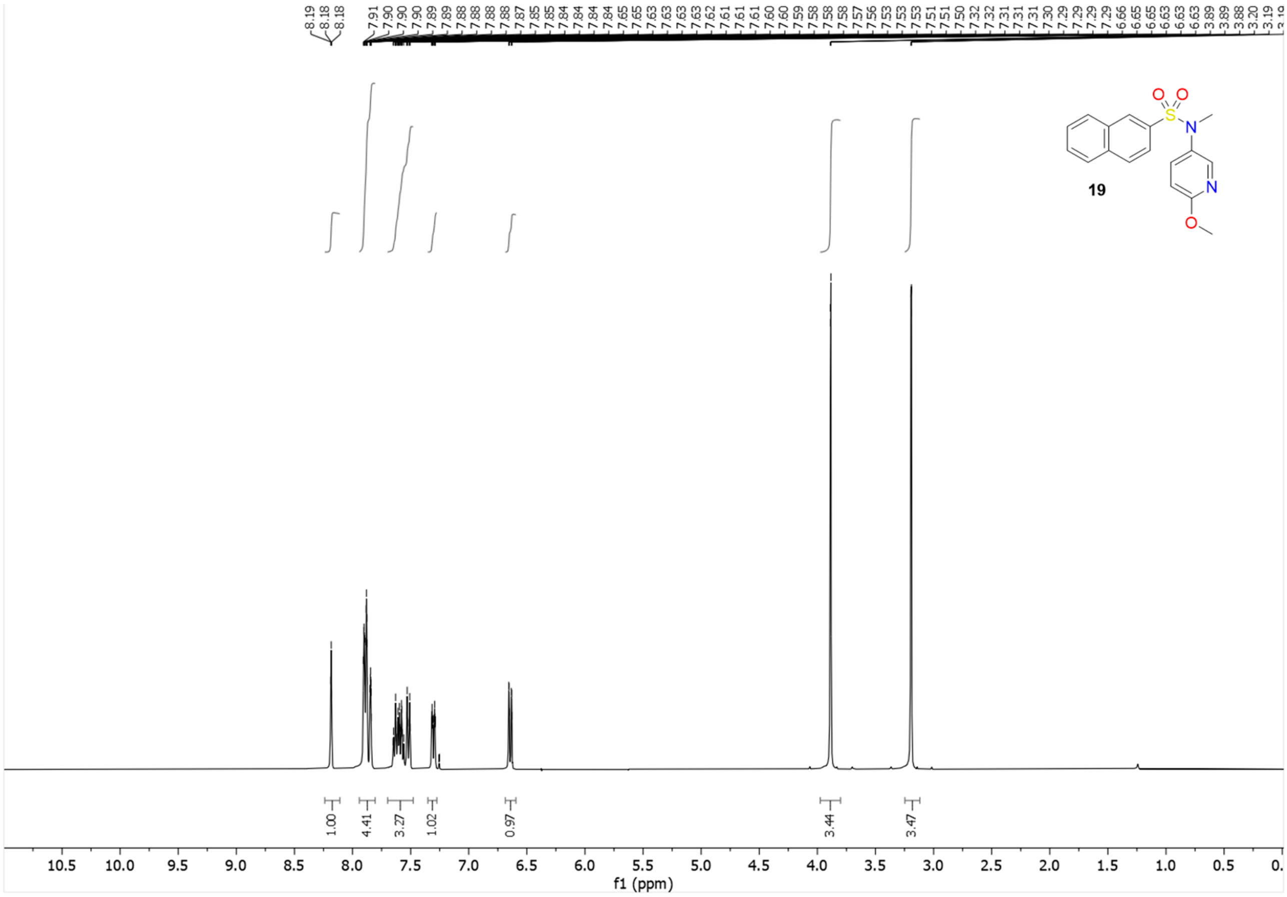
19
1.00
4.41
3.27
1.02
0.97
3.44
3.47
f1 (ppm)

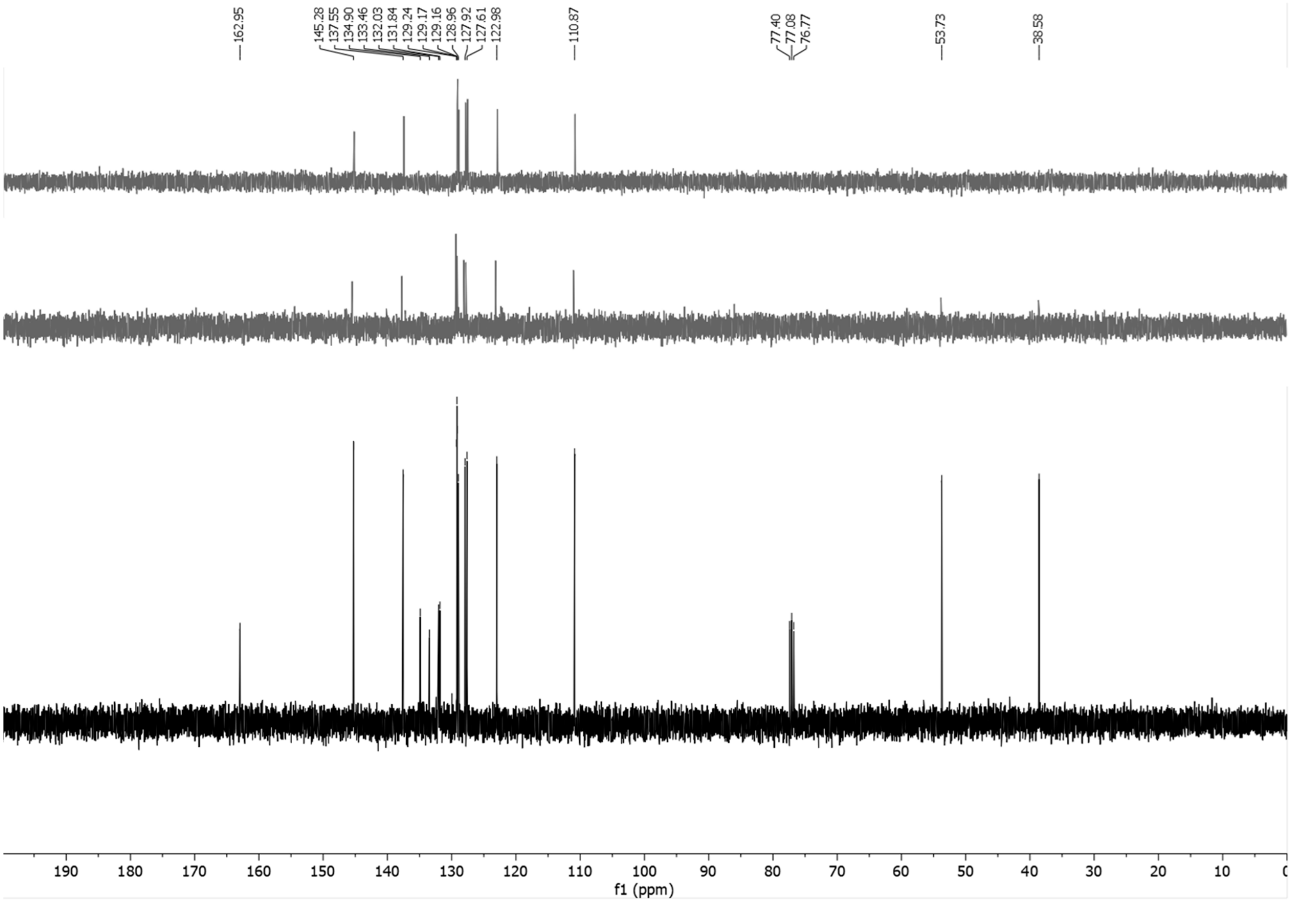
162.95
145.28
137.55
134.90
133.46
132.03
131.84
129.24
129.17
129.16
128.96
127.92
127.61
122.98
110.87
77.40
77.08
76.77
53.73
38.58
f1 (ppm)

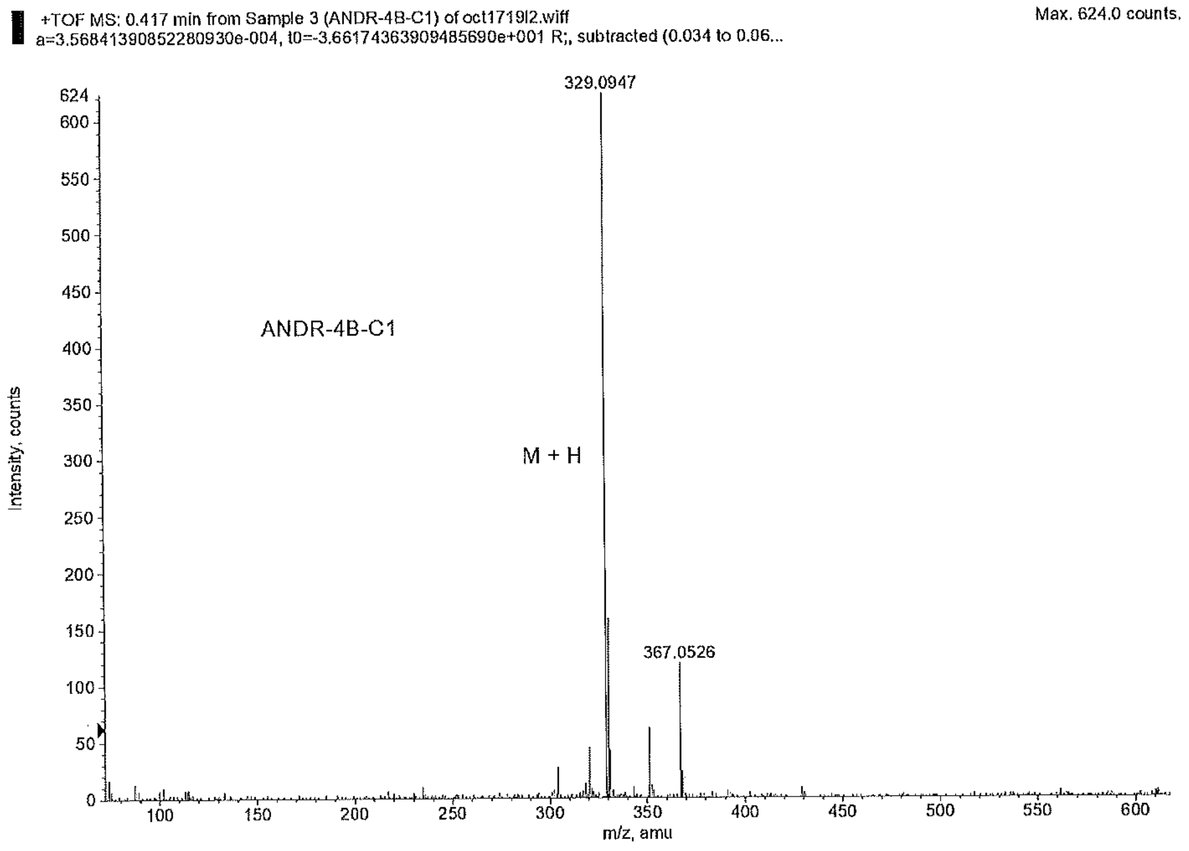

^{1}H, ^{13}C NMR and HRMS spectra of ***N*-(6-methoxypyridin-3-yl)-*N*-methylnaphthalene-2-sulfonamide (19)**

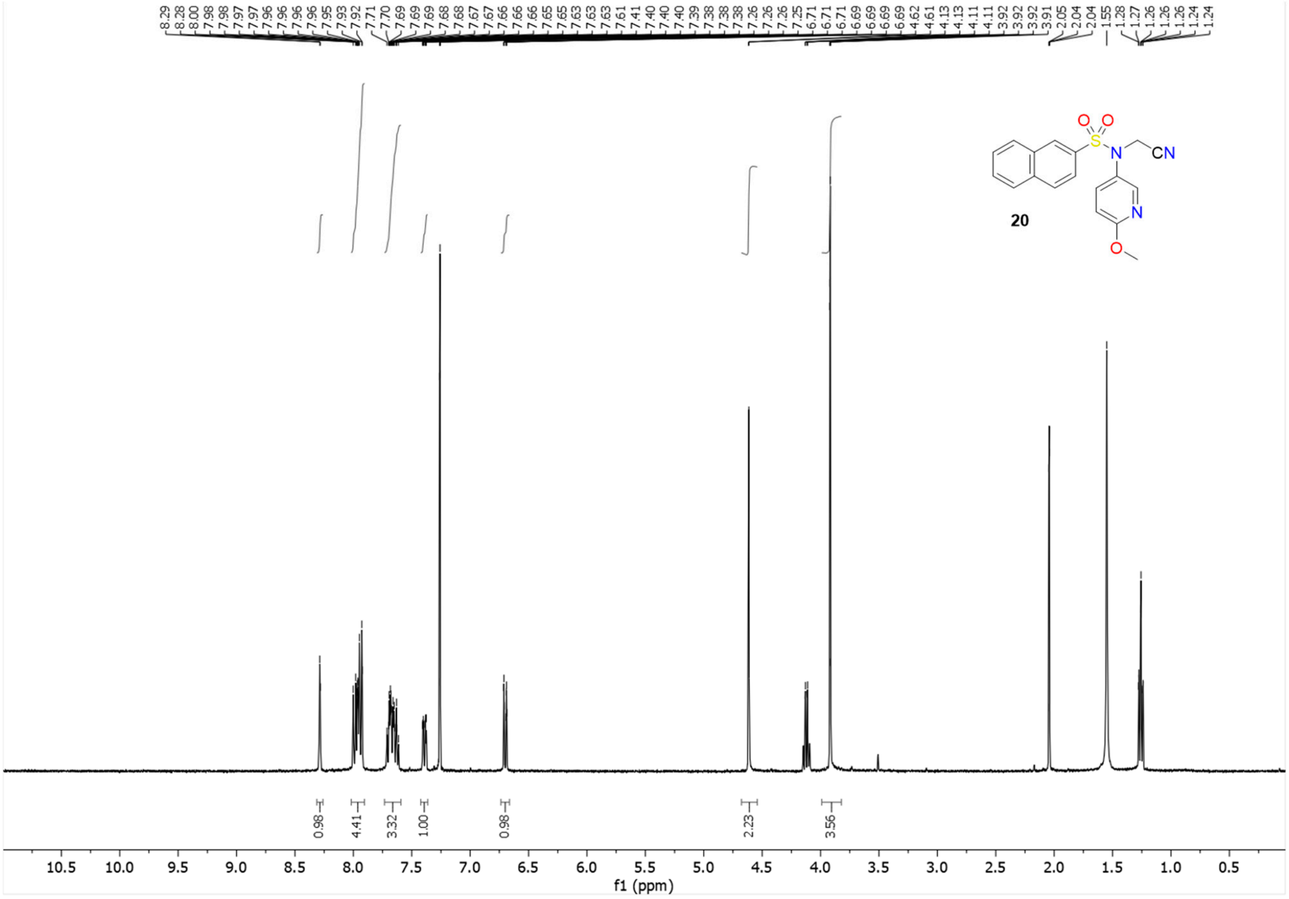
8.29 8.28 8.00 7.98 7.98 7.97 7.97 7.96 7.96 7.96 7.96 7.95 7.93 7.92 7.71 7.70 7.69 7.69 7.69 7.68 7.68 7.67 7.66 7.66 7.66 7.65 7.65 7.63 7.63 7.63 7.61 7.41 7.40 7.40 7.39 7.38 7.38 7.38 7.26 7.26 7.26 7.25 6.71 6.71 6.69 6.69 6.69 4.62 4.61 4.13 4.13 4.11 3.92 3.92 3.92 3.91 2.05 2.04 2.04 1.55 1.28 1.27 1.26 1.26 1.26 1.24 1.24
CN
N
O
S
O
N
O
20
0.98
4.41
3.32
1.00
0.98
2.23
3.56
10.5 10.0 9.5 9.0 8.5 8.0 7.5 7.0 6.5 6.0 5.5 5.0 4.5 4.0 3.5 3.0 2.5 2.0 1.5 1.0 0.5
f1 (ppm)

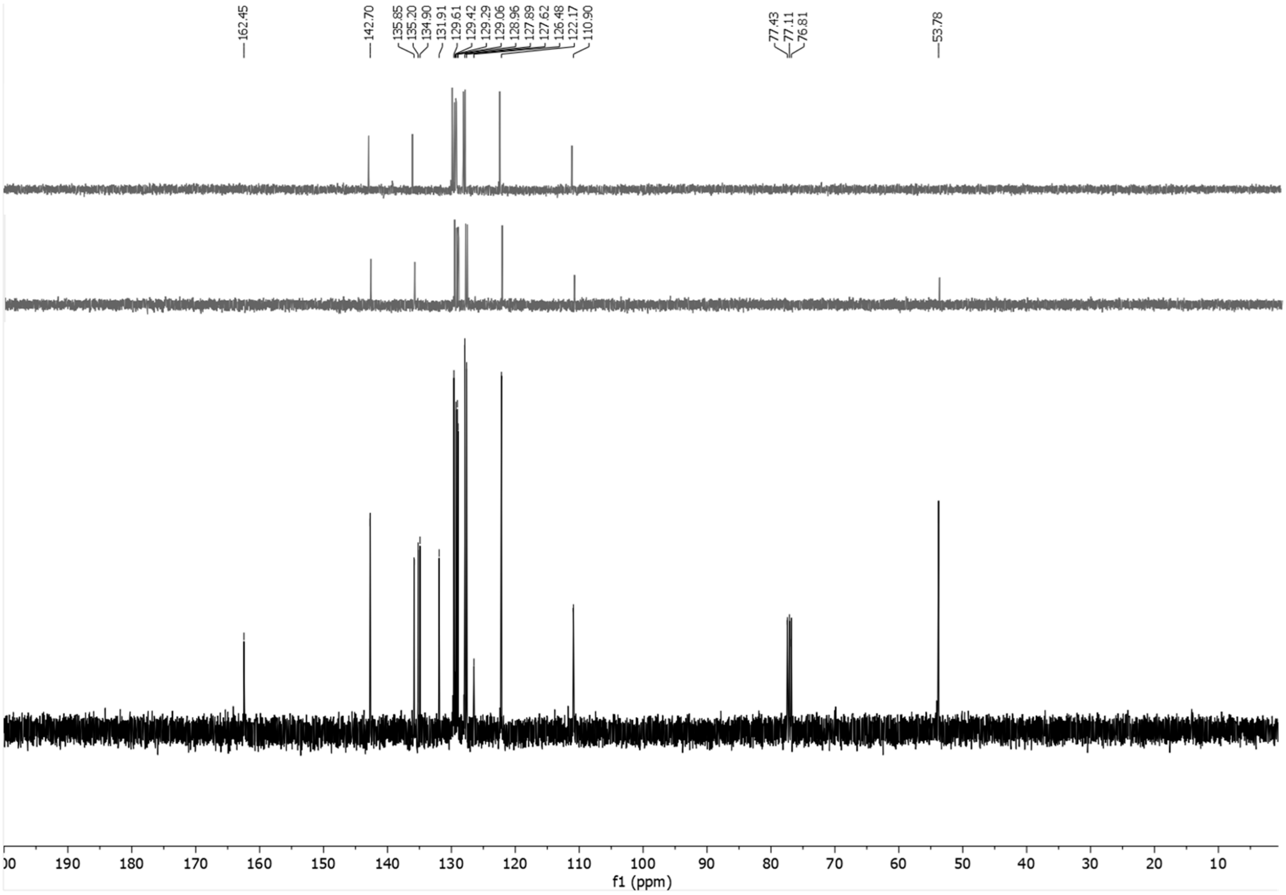
162.45
142.70
135.85 135.20 134.90 131.91 129.61 129.42 129.29 129.06 128.96 127.89 127.62 126.48 122.17 110.90
77.43 77.11 76.81
53.78
190 180 170 160 150 140 130 120 110 100 90 80 70 60 50 40 30 20 10
f1 (ppm)

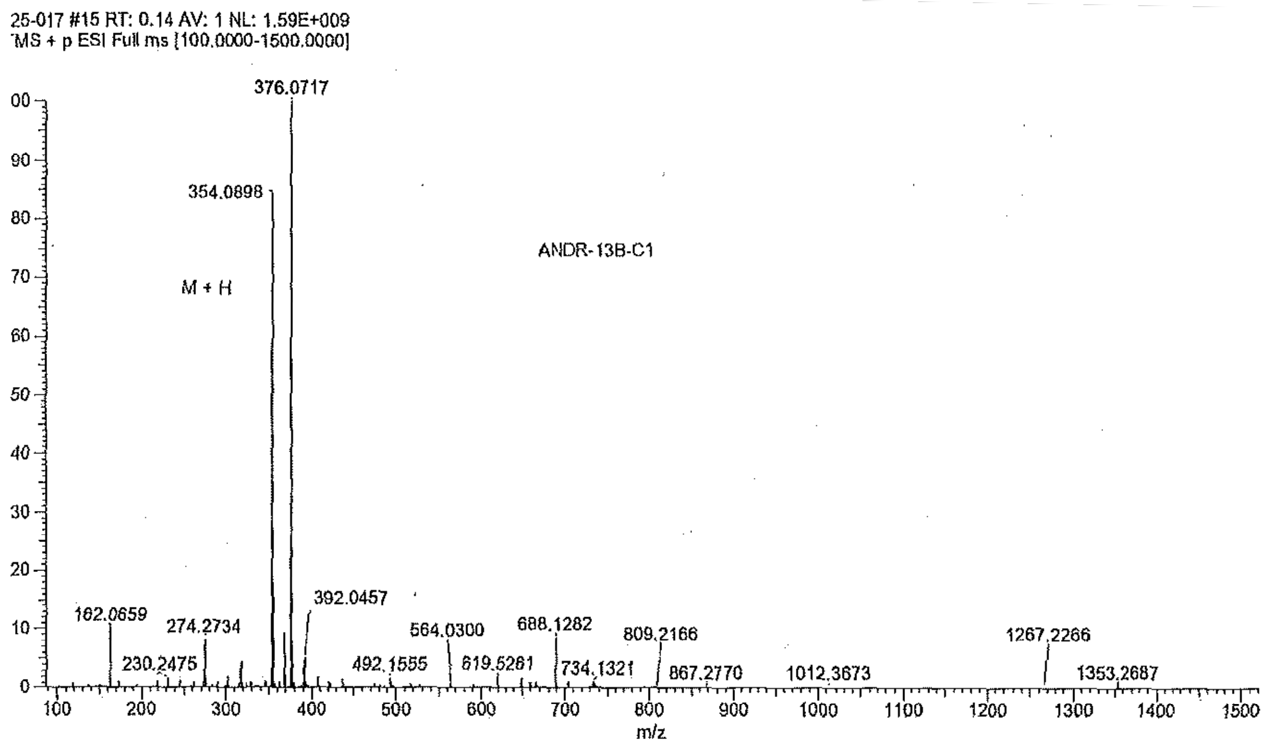

^{1}H, ^{13}C NMR and HRMS spectra of ***N*-(cyanomethyl)-*N*-(6-methoxypyridin-3-yl)naphthalene-2-sulfonamide (20)**

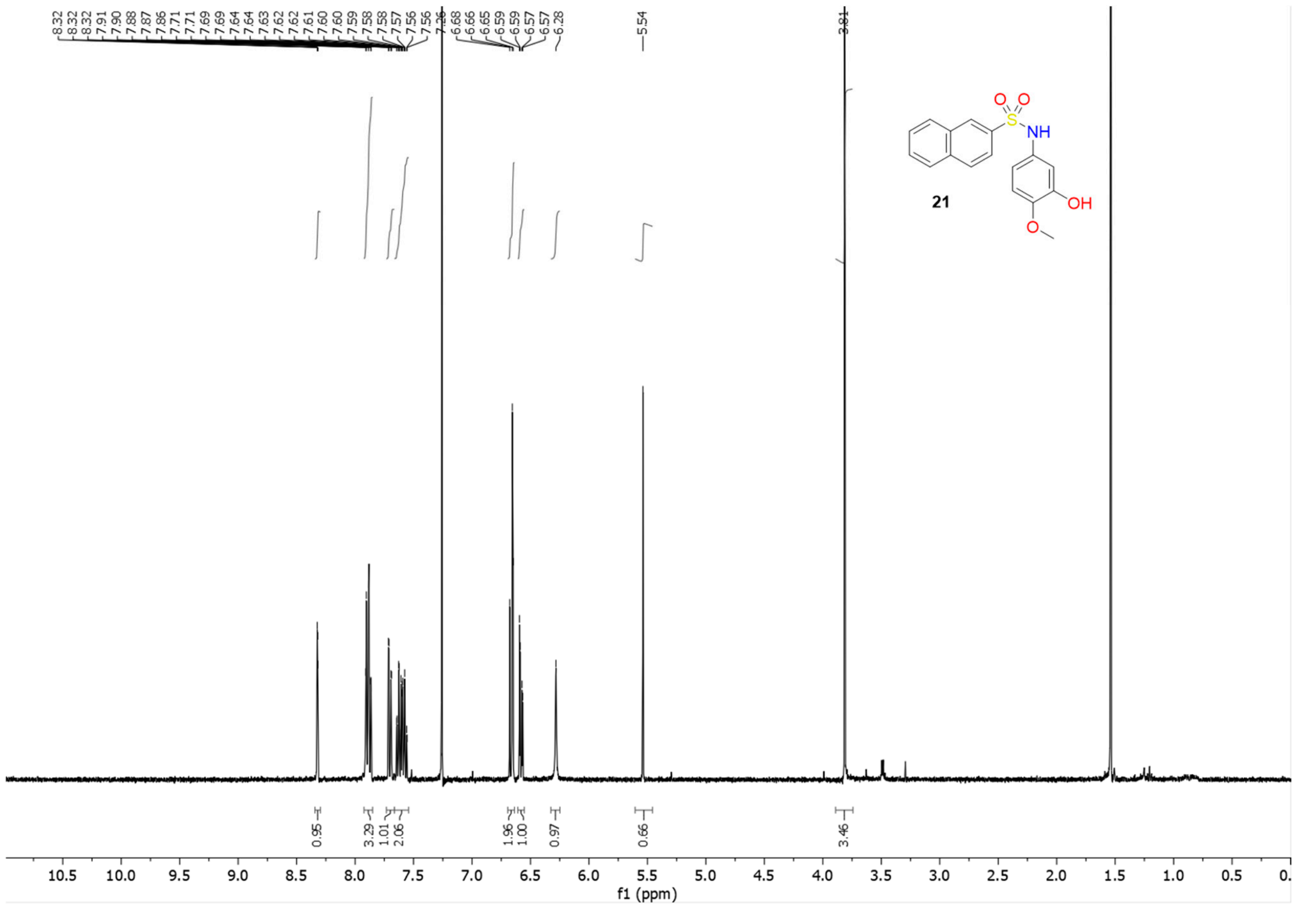
8.32 8.32 8.32 7.91 7.90 7.88 7.87 7.86 7.71 7.71 7.69 7.69 7.64 7.64 7.63 7.62 7.62 7.61 7.60 7.60 7.59 7.58 7.58 7.58 7.57 7.56 7.56 7.26 6.68 6.66 6.65 6.59 6.59 6.57 6.57 6.28
5.54
3.81
O O
S
NH
21
OH
O
0.95
3.29
1.01
2.06
1.96
1.00
0.97
0.66
3.46
10.5 10.0 9.5 9.0 8.5 8.0 7.5 7.0 6.5 6.0 5.5 5.0 4.5 4.0 3.5 3.0 2.5 2.0 1.5 1.0 0.5
f1 (ppm)

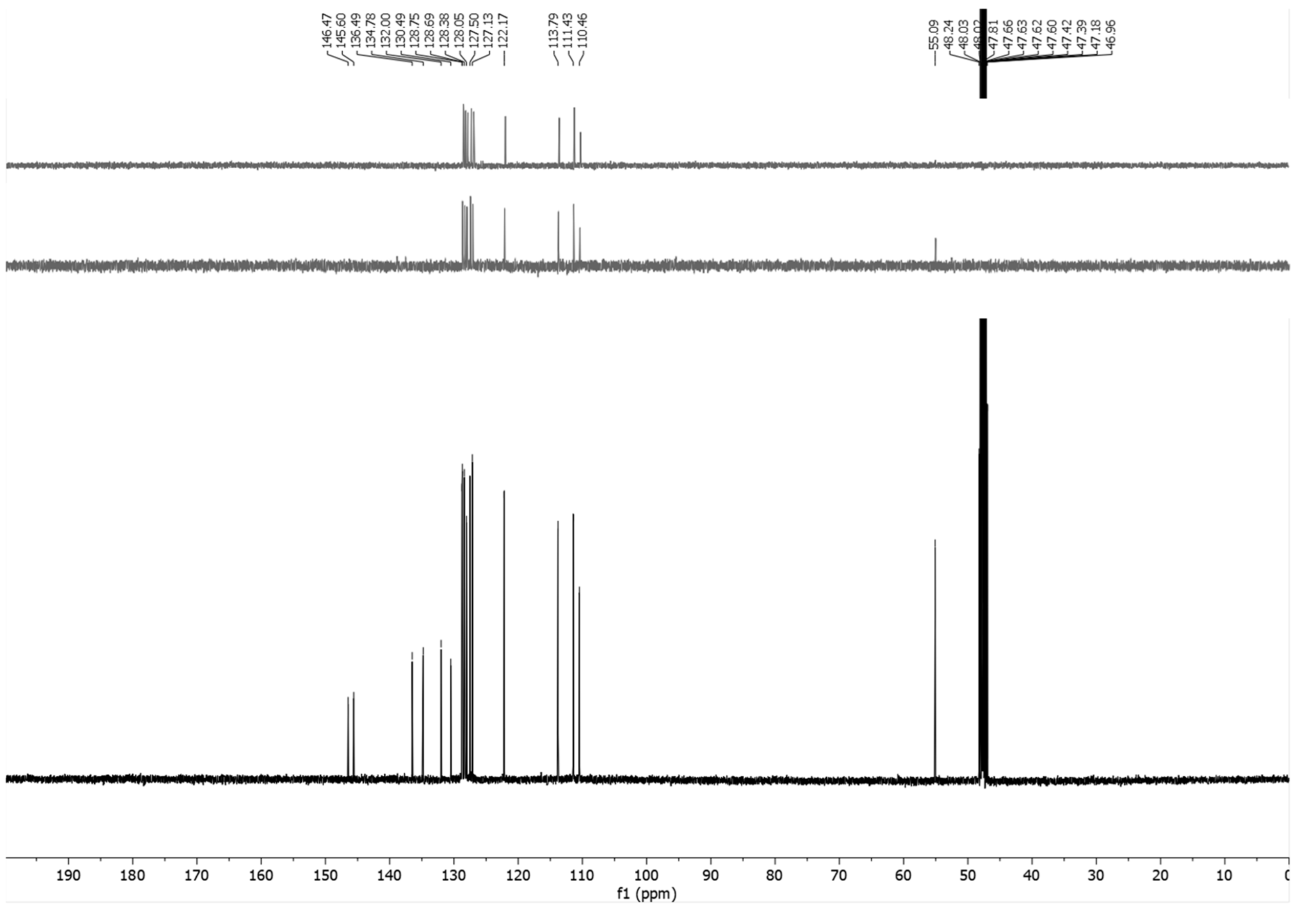
146.47 145.60 136.49 134.78 132.00 130.49 128.75 128.69 128.38 128.05 127.50 127.13 122.17
113.79 111.43 110.46
55.09 48.24 48.03 48.02 47.81 47.66 47.63 47.62 47.60 47.42 47.39 47.18 46.96
190 180 170 160 150 140 130 120 110 100 90 80 70 60 50 40 30 20 10
f1 (ppm)

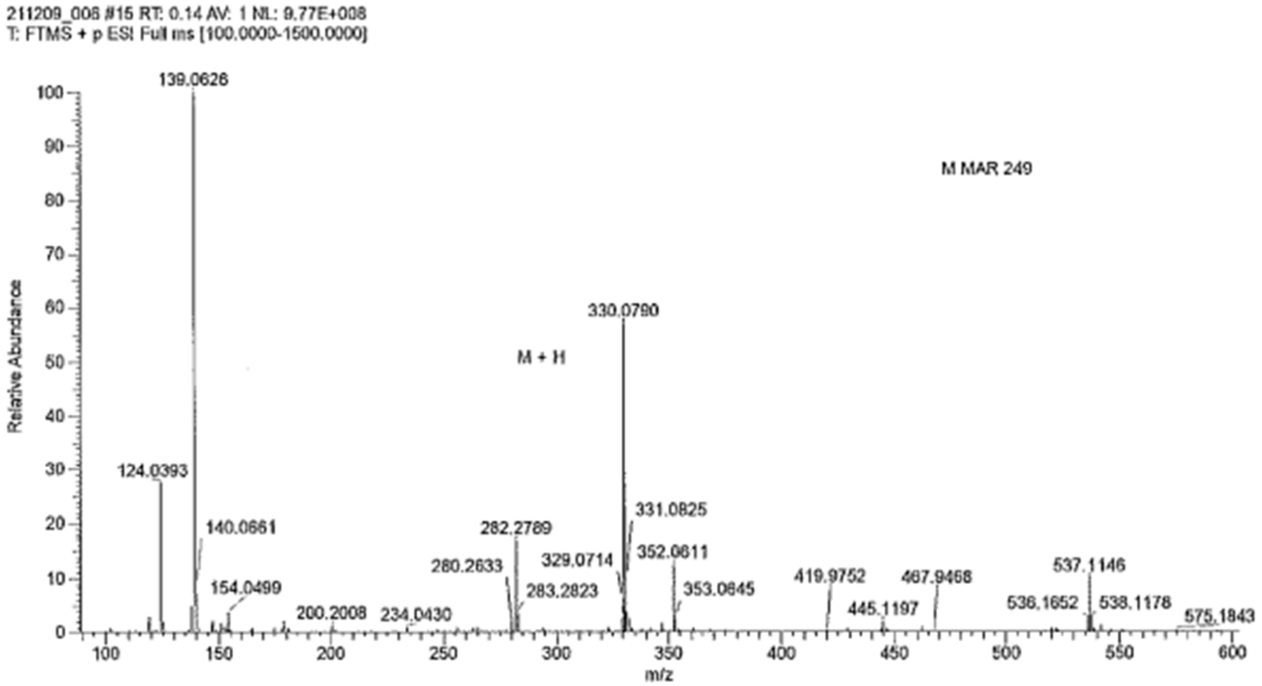

¹H, ¹³C NMR and HRMS spectra of ***N*-(3-hydroxy-4-methoxyphenyl)naphthalene-2-sulfonamide (21)**

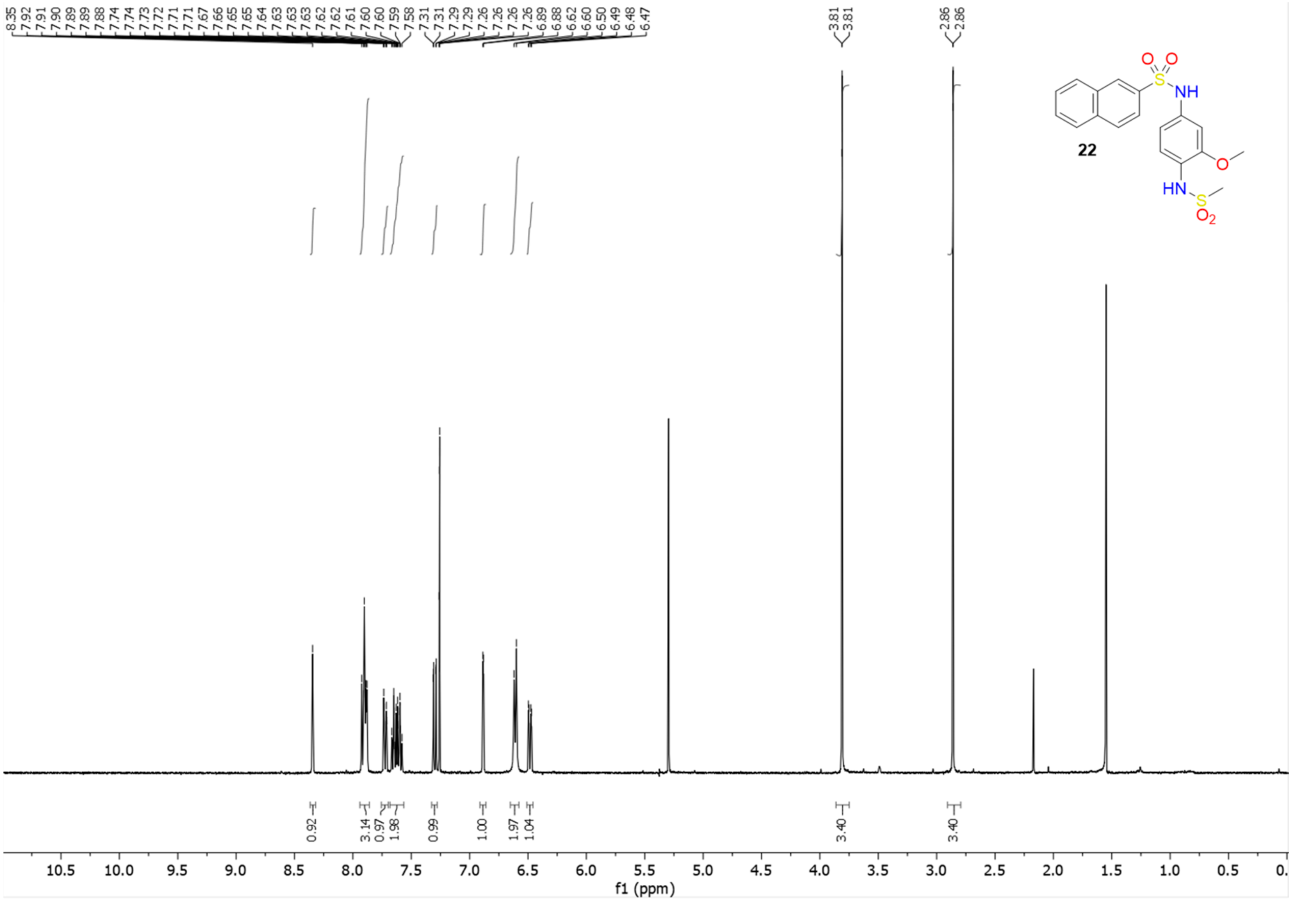
8.35
7.92
7.91
7.90
7.89
7.89
7.88
7.74
7.74
7.73
7.72
7.71
7.67
7.66
7.65
7.65
7.64
7.63
7.63
7.62
7.62
7.61
7.60
7.60
7.59
7.58
7.31
7.31
7.29
7.29
7.26
7.26
7.26
7.26
6.89
6.88
6.62
6.60
6.50
6.49
6.48
6.47
3.81
3.81
2.86
2.86
O
O
S
NH
O
HN
S
O2
22
0.92
3.14
0.97
1.98
0.99
1.00
1.97
1.04
3.40
3.40
10.5
10.0
9.5
9.0
8.5
8.0
7.5
7.0
6.5
6.0
5.5
5.0
4.5
4.0
3.5
3.0
2.5
2.0
1.5
1.0
0.5
f1 (ppm)

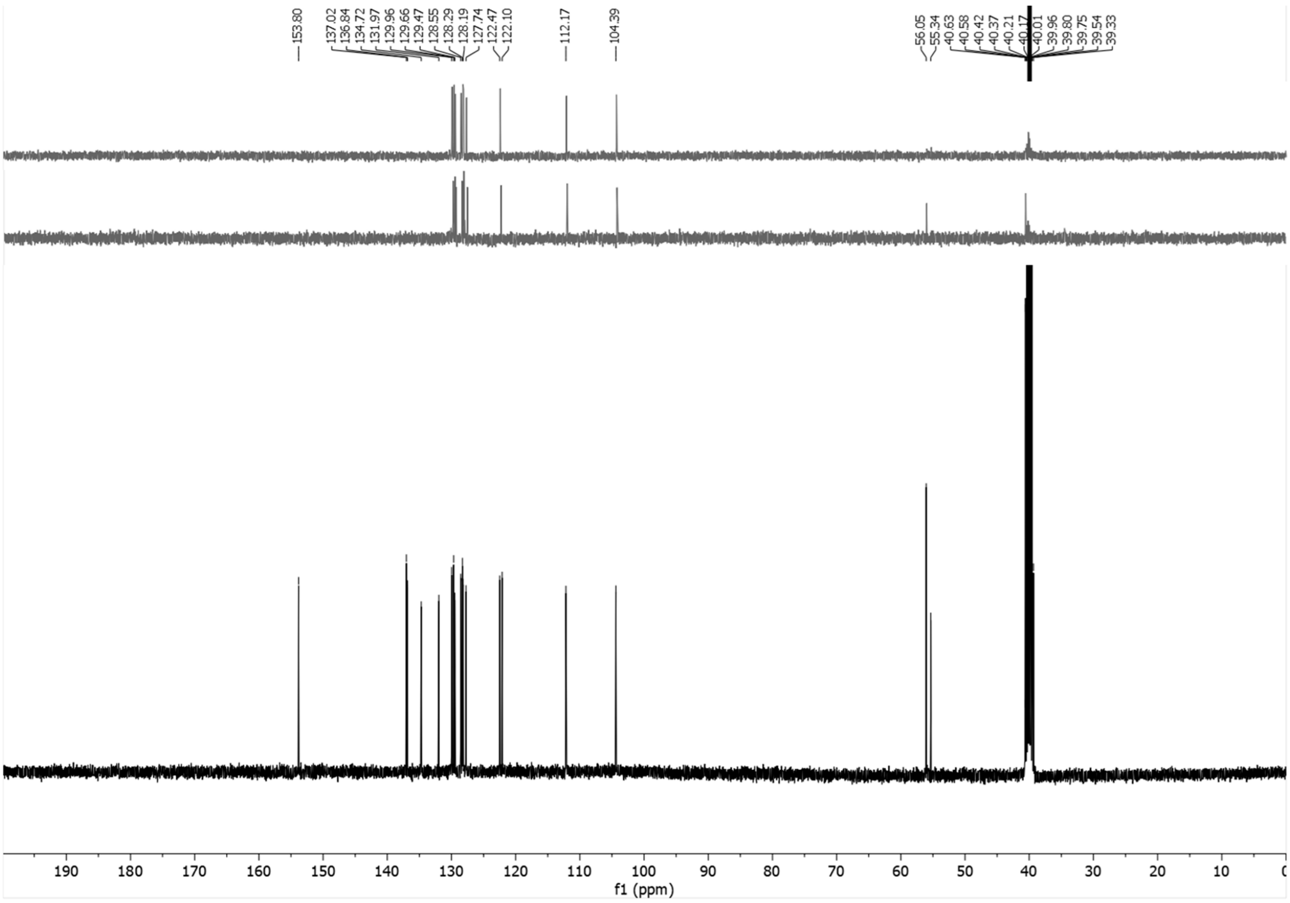
153.80
137.02
136.84
134.72
131.97
129.96
129.66
129.47
128.55
128.29
128.19
127.74
122.47
122.10
112.17
104.39
56.05
55.34
40.63
40.58
40.42
40.37
40.21
40.17
40.01
39.96
39.80
39.75
39.54
39.33
190
180
170
160
150
140
130
120
110
100
90
80
70
60
50
40
30
20
10
f1 (ppm)

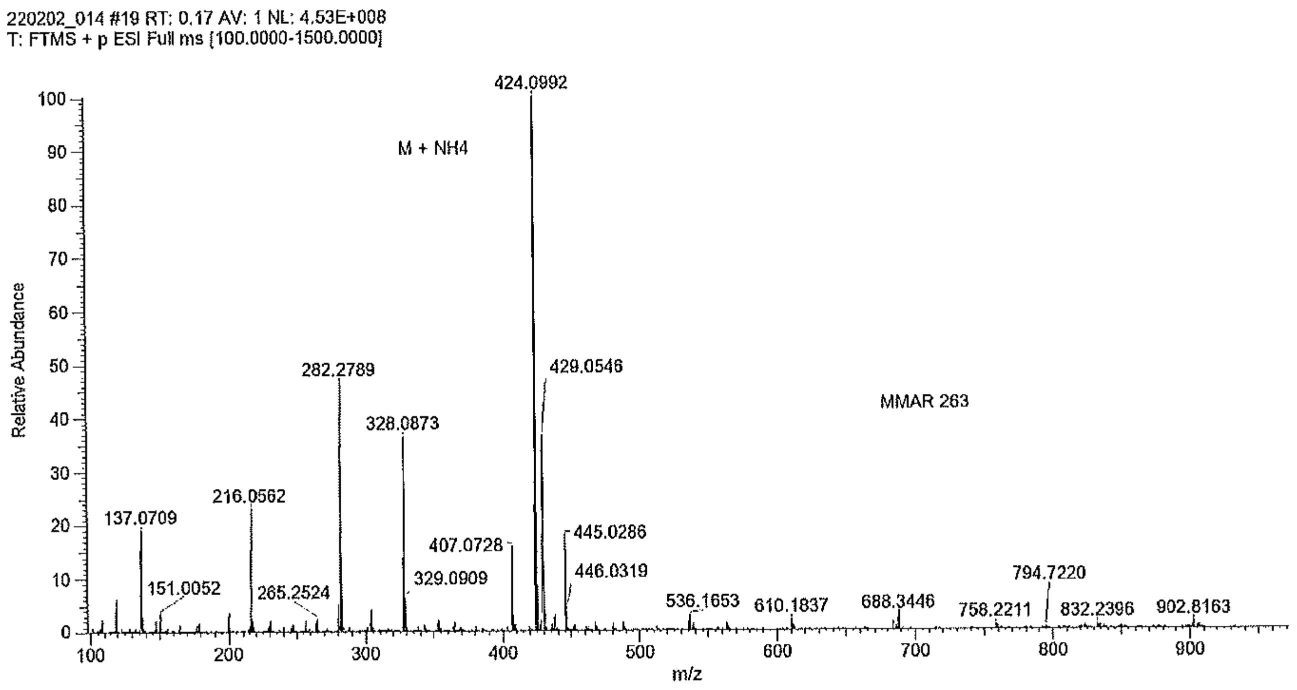

^{1}H, ^{13}C NMR and HRMS spectra of *N*-**(3-methoxy-4-(methylsulfonamido)phenyl)naphthalene-2-sulfonamide (22)**

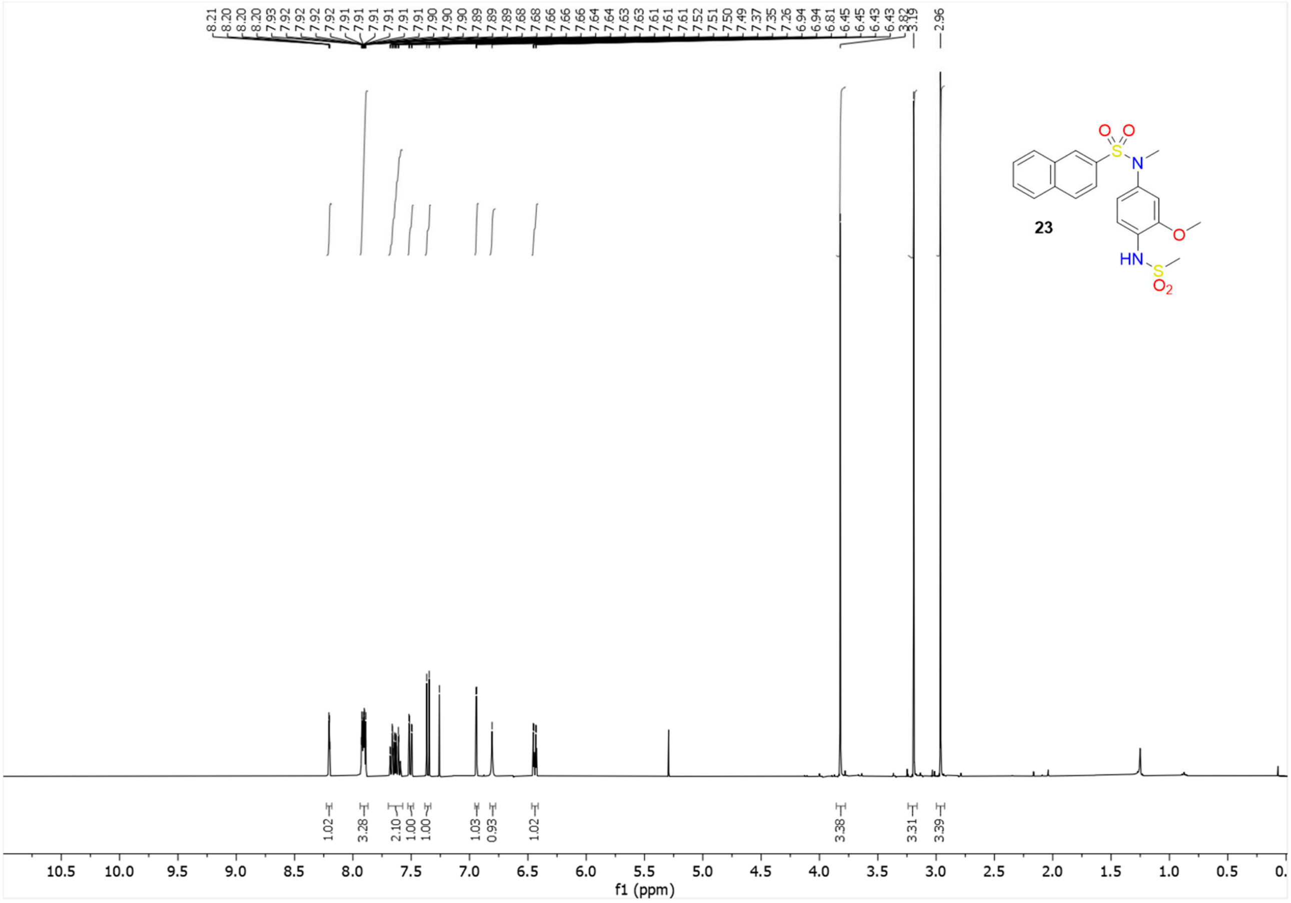

8.21
8.20
8.20
8.20
7.93
7.92
7.92
7.92
7.92
7.91
7.91
7.91
7.91
7.91
7.91
7.90
7.90
7.89
7.89
7.89
7.68
7.68
7.66
7.66
7.66
7.64
7.64
7.63
7.61
7.61
7.61
7.52
7.51
7.50
7.49
7.37
7.35
7.26
6.94
6.94
6.81
6.45
6.45
6.43
6.43
3.82
3.19
2.96
23
HN
O
S
N
O2
1.02
3.28
2.10
1.00
1.00
1.03
0.93
1.02
3.38
3.31
3.39
10.5 10.0 9.5 9.0 8.5 8.0 7.5 7.0 6.5 6.0 5.5 5.0 4.5 4.0 3.5 3.0 2.5 2.0 1.5 1.0 0.5
f1 (ppm)

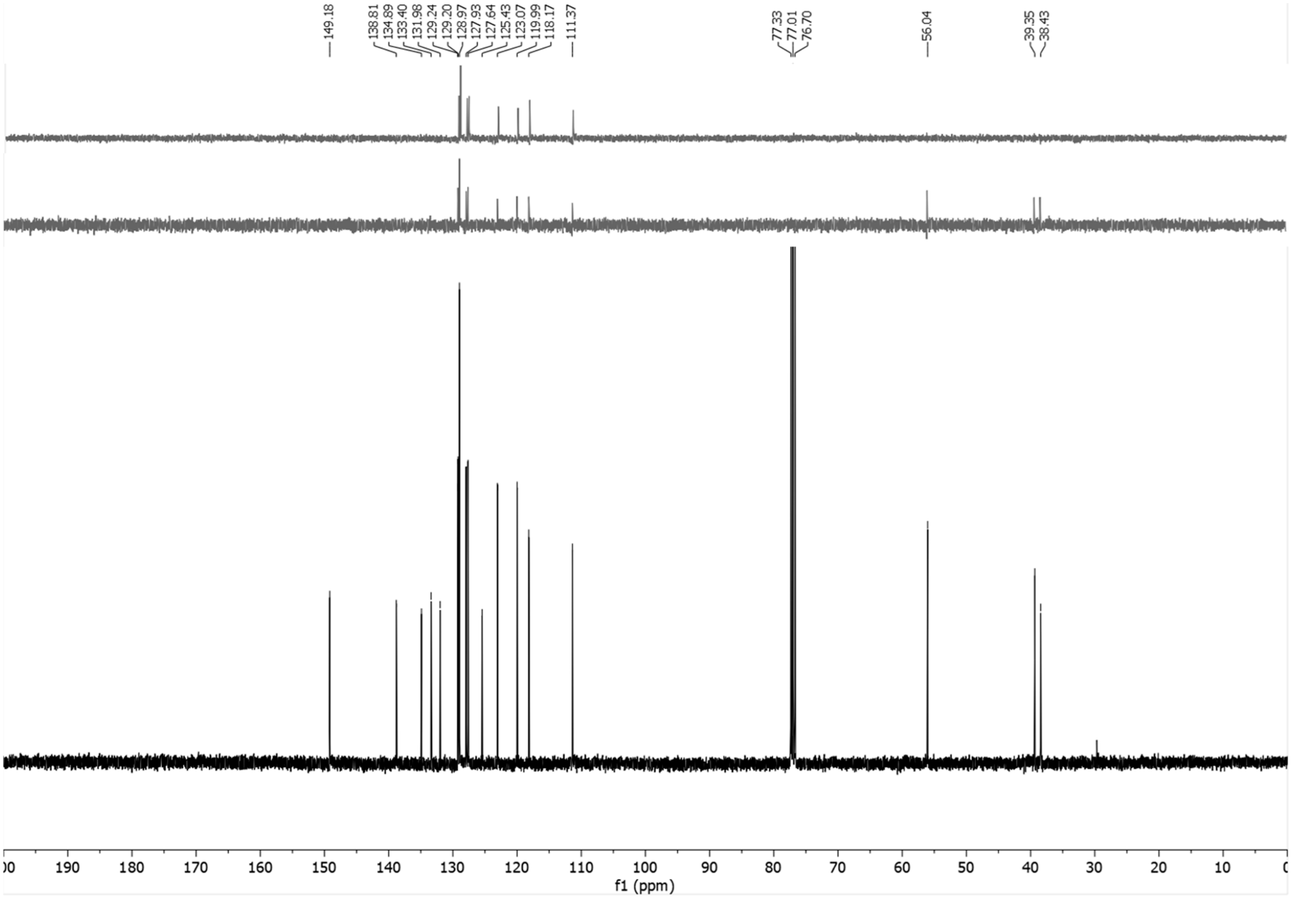

149.18
138.81
134.89
133.40
131.98
129.24
129.20
128.97
127.93
127.64
125.43
123.07
119.99
118.17
111.37
77.33
77.01
76.70
56.04
39.35
38.43
190 180 170 160 150 140 130 120 110 100 90 80 70 60 50 40 30 20 10
f1 (ppm)

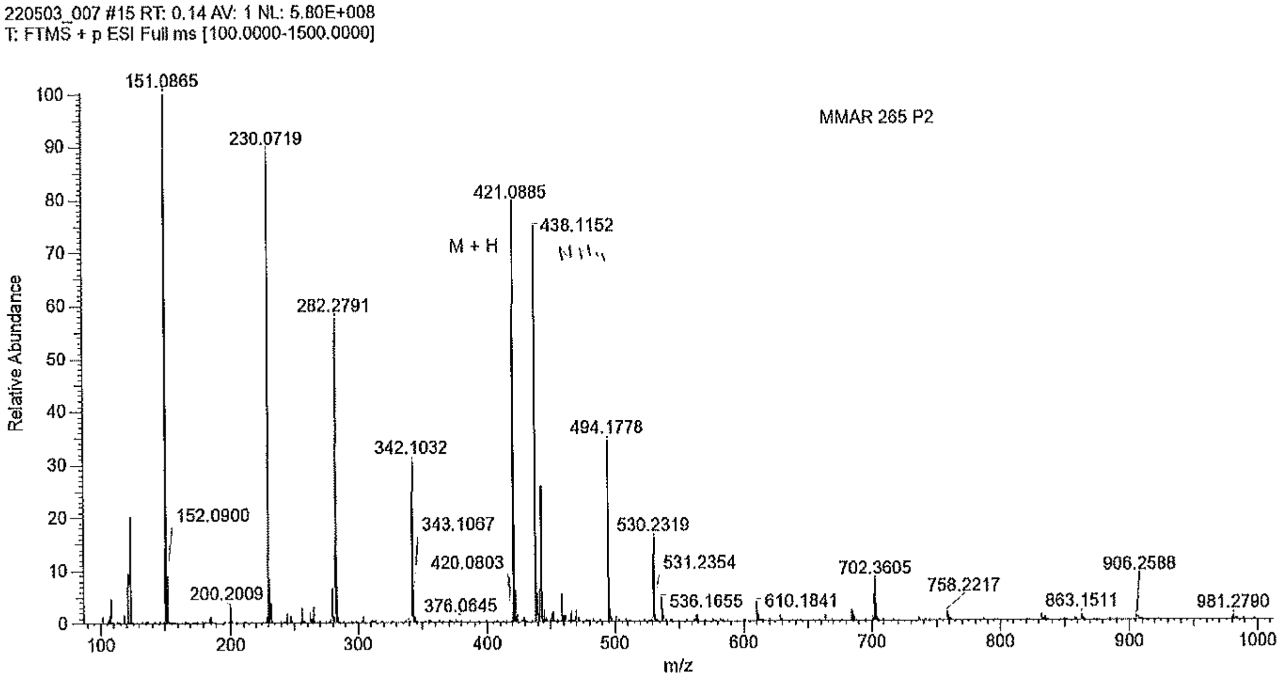

^1H, ^{13}C NMR and HRMS spectra of ***N*-(3-methoxy-4-(methylsulfonamido)phenyl)-*N*-methylnaphthalene-2-sulfonamide (23)**

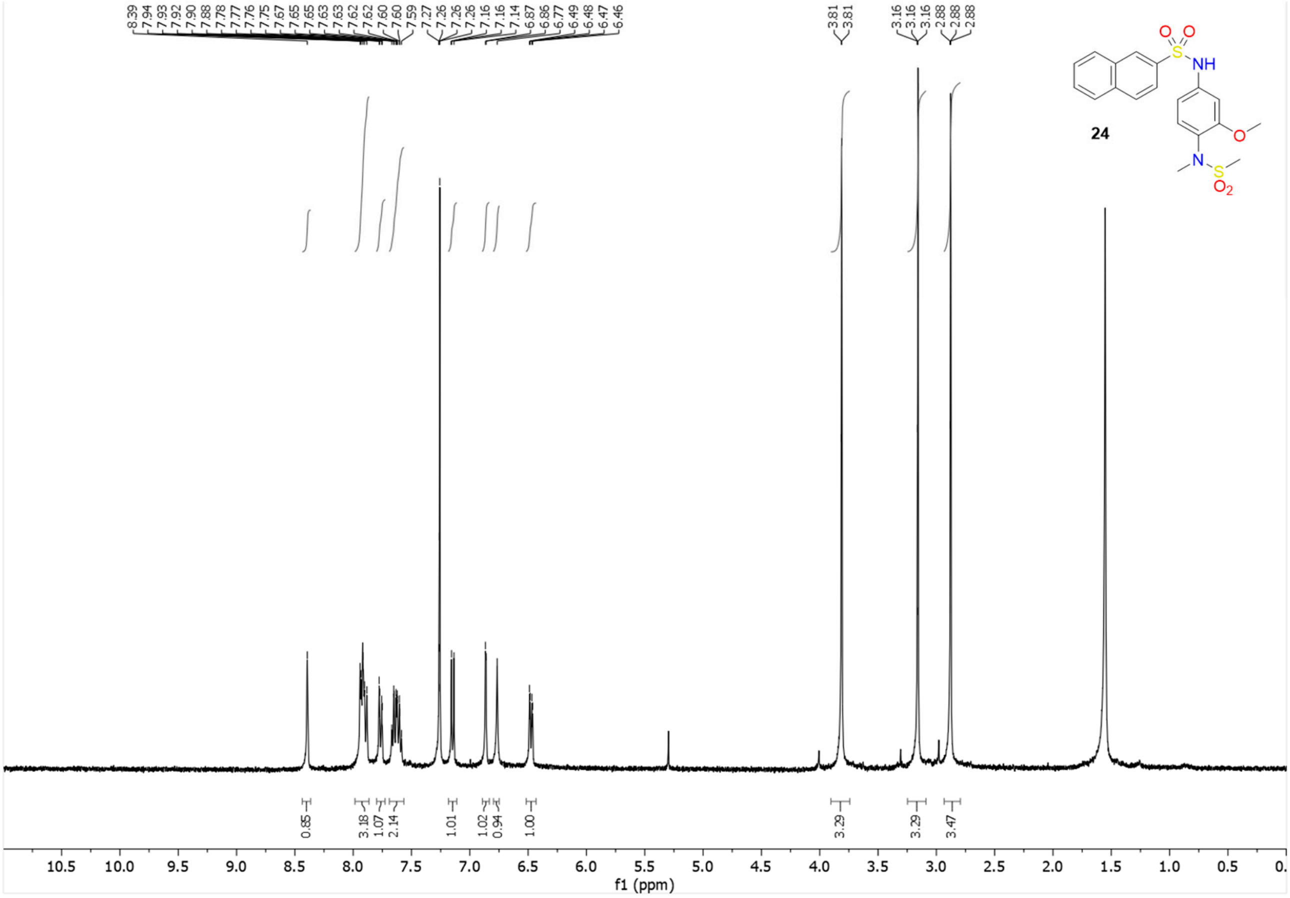

24
f1 (ppm)

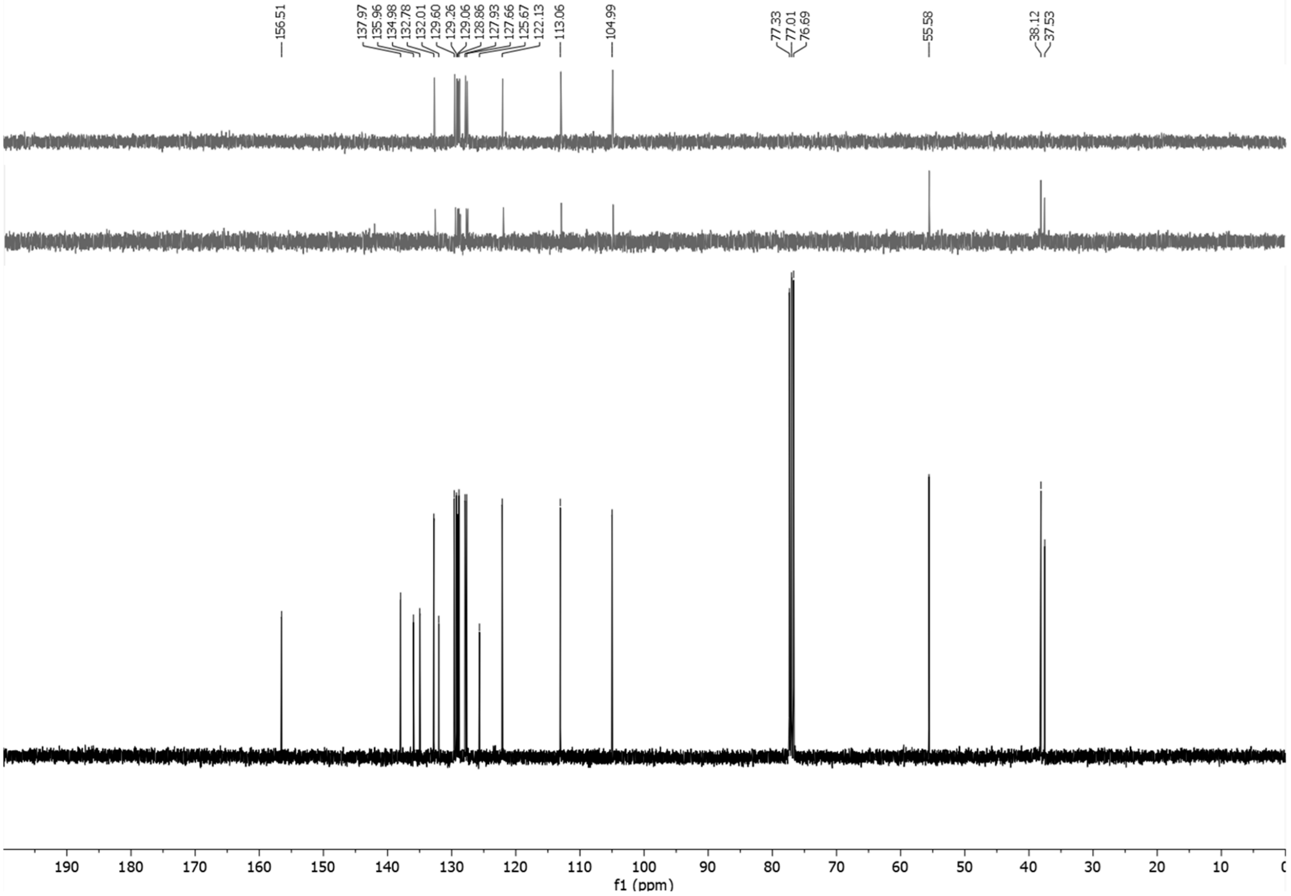

f1 (ppm)

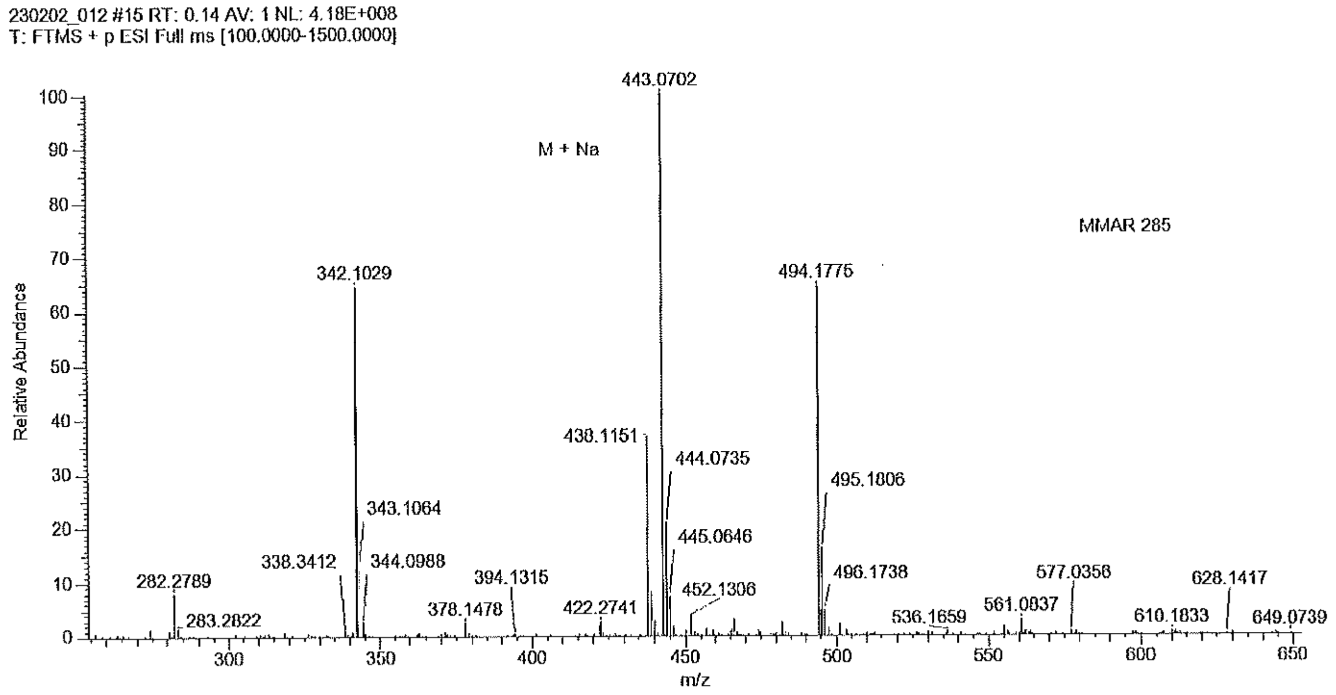

^{1}H, ^{13}C NMR and HRMS spectra of ***N*-(3-methoxy-4-(*N*-methylmethylsulfonamido)phenyl)naphthalene-2-sulfonamide (24)**

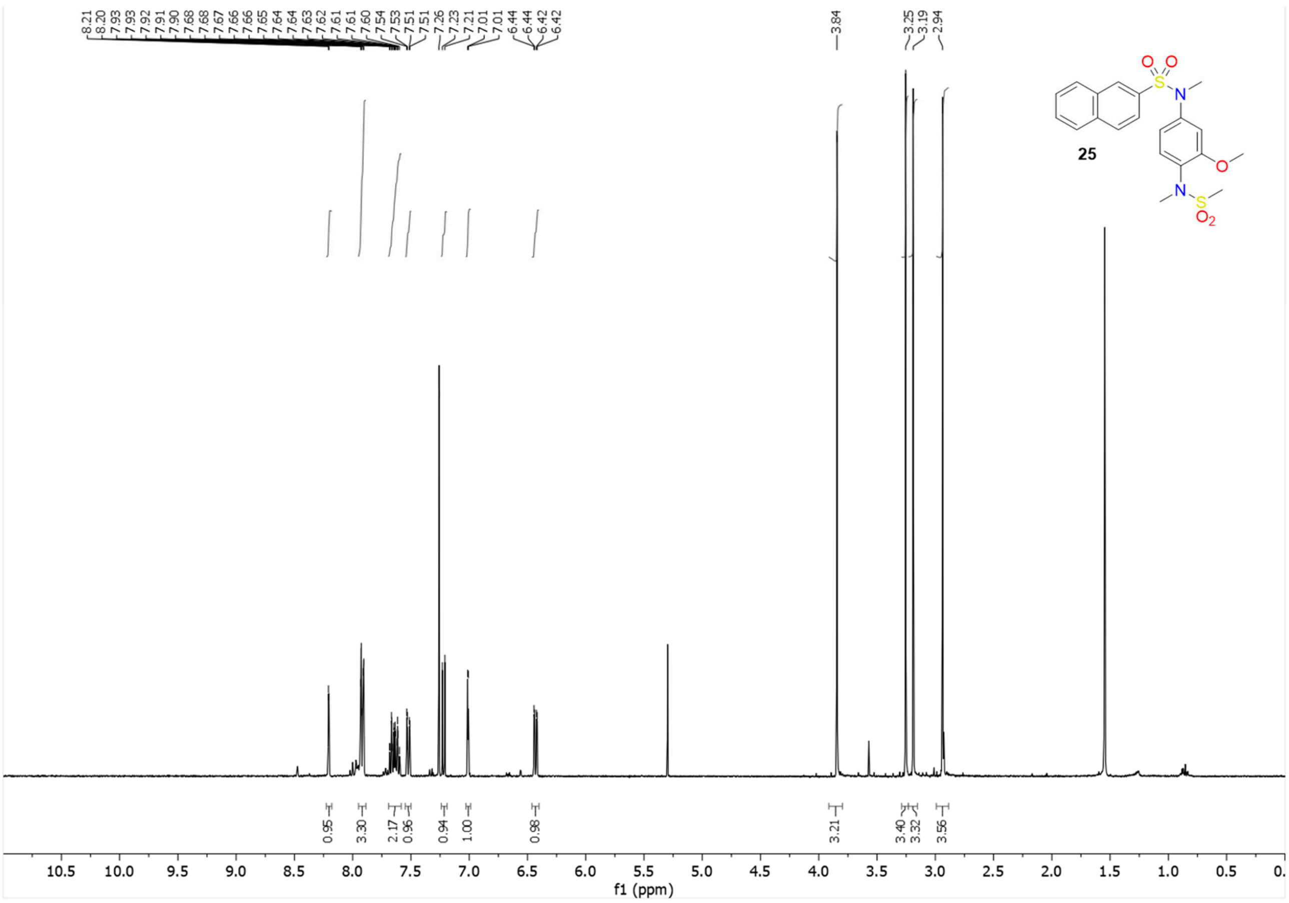
8.21 8.20 7.93 7.93 7.92 7.91 7.90 7.68 7.68 7.67 7.66 7.66 7.65 7.64 7.64 7.63 7.62 7.61 7.61 7.60 7.54 7.53 7.51 7.51 7.26 7.23 7.21 7.01 7.01 6.44 6.44 6.42 6.42
3.84
3.25 3.19 2.94
25
0.95 3.30 2.17 0.96 0.94 1.00 0.98 3.21 3.40 3.32 3.56
f1 (ppm)

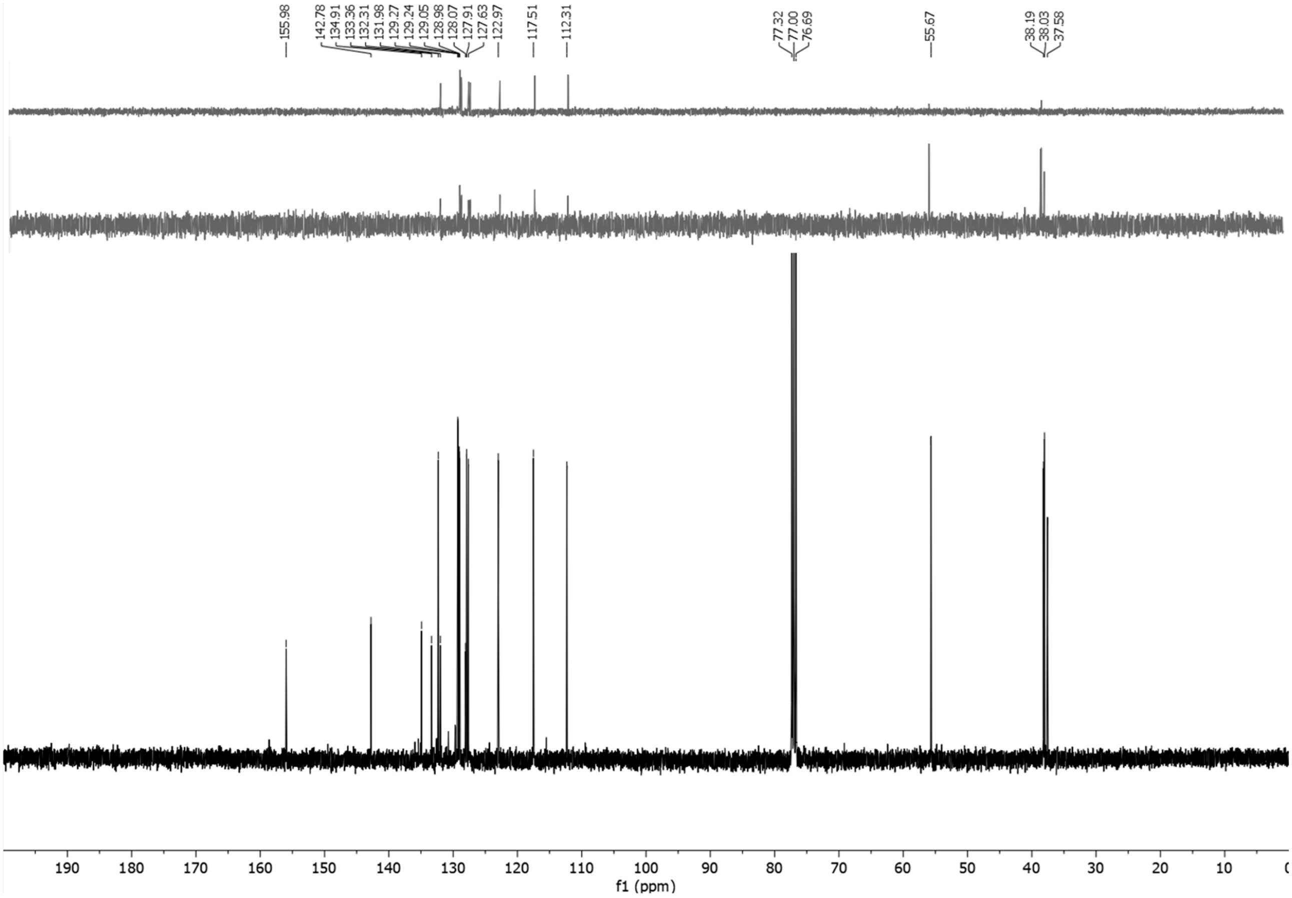
155.98
142.78 134.91 133.36 132.31 131.98 129.27 129.24 129.05 128.98 128.07 127.91 127.63 122.97 117.51 112.31
77.32 77.00 76.69
55.67
38.19 38.03 37.58
f1 (ppm)

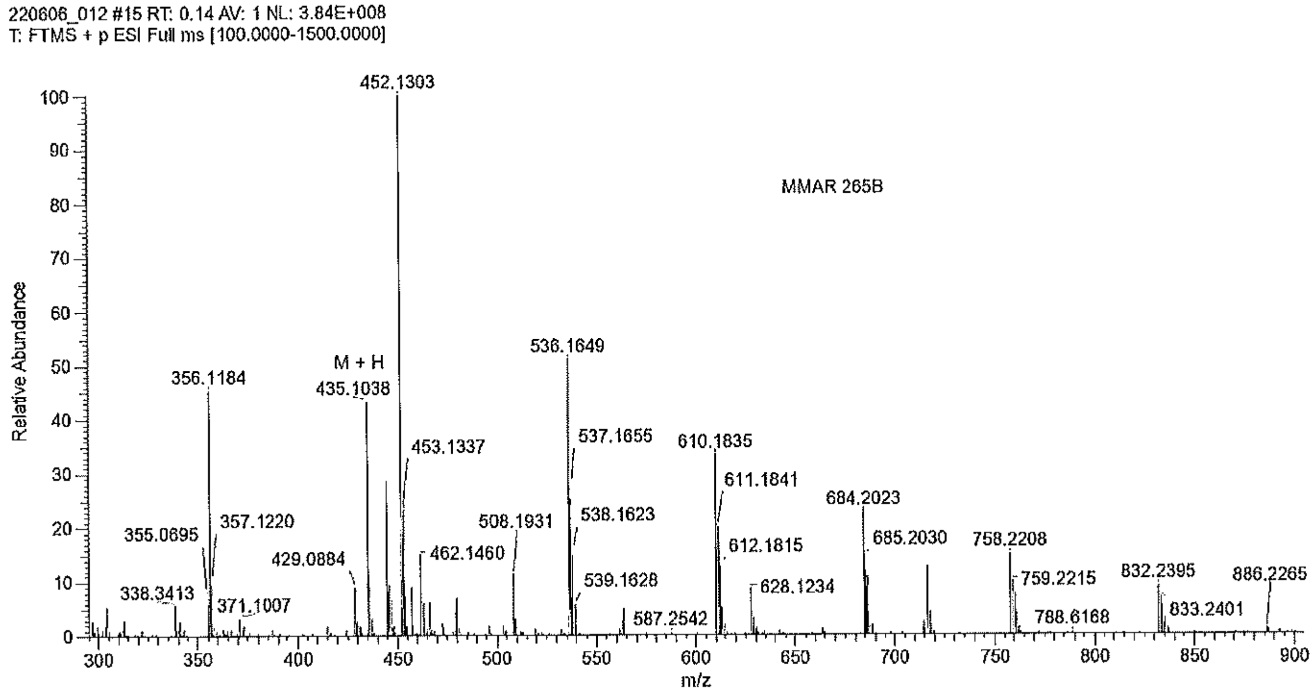

^{1}H, ^{13}C NMR and HRMS spectra of ***N*-(3-methoxy-4-(*N*-methylmethylsulfonamido)phenyl)-*N*-methylnaphthalene-2-sulfonamide (25)**

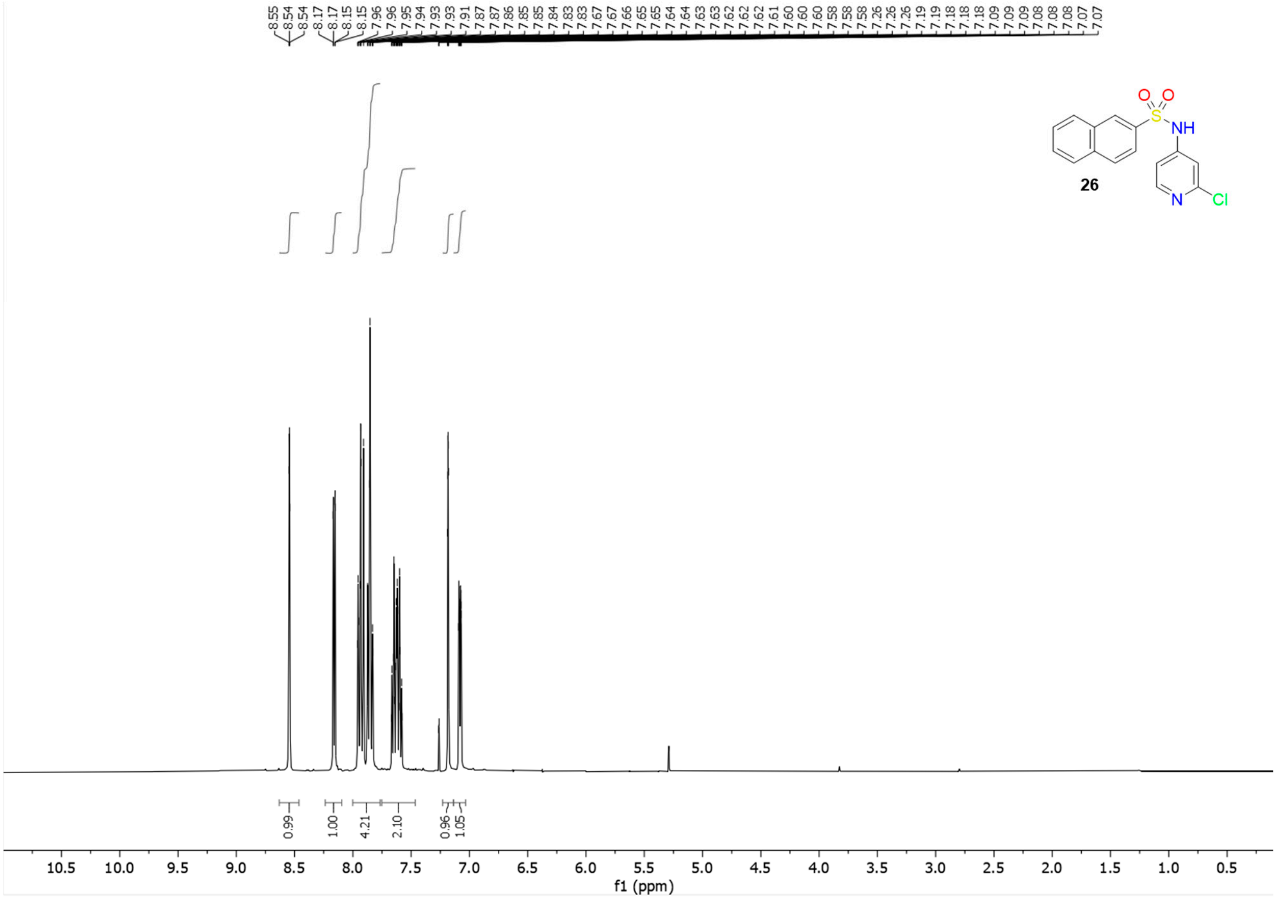

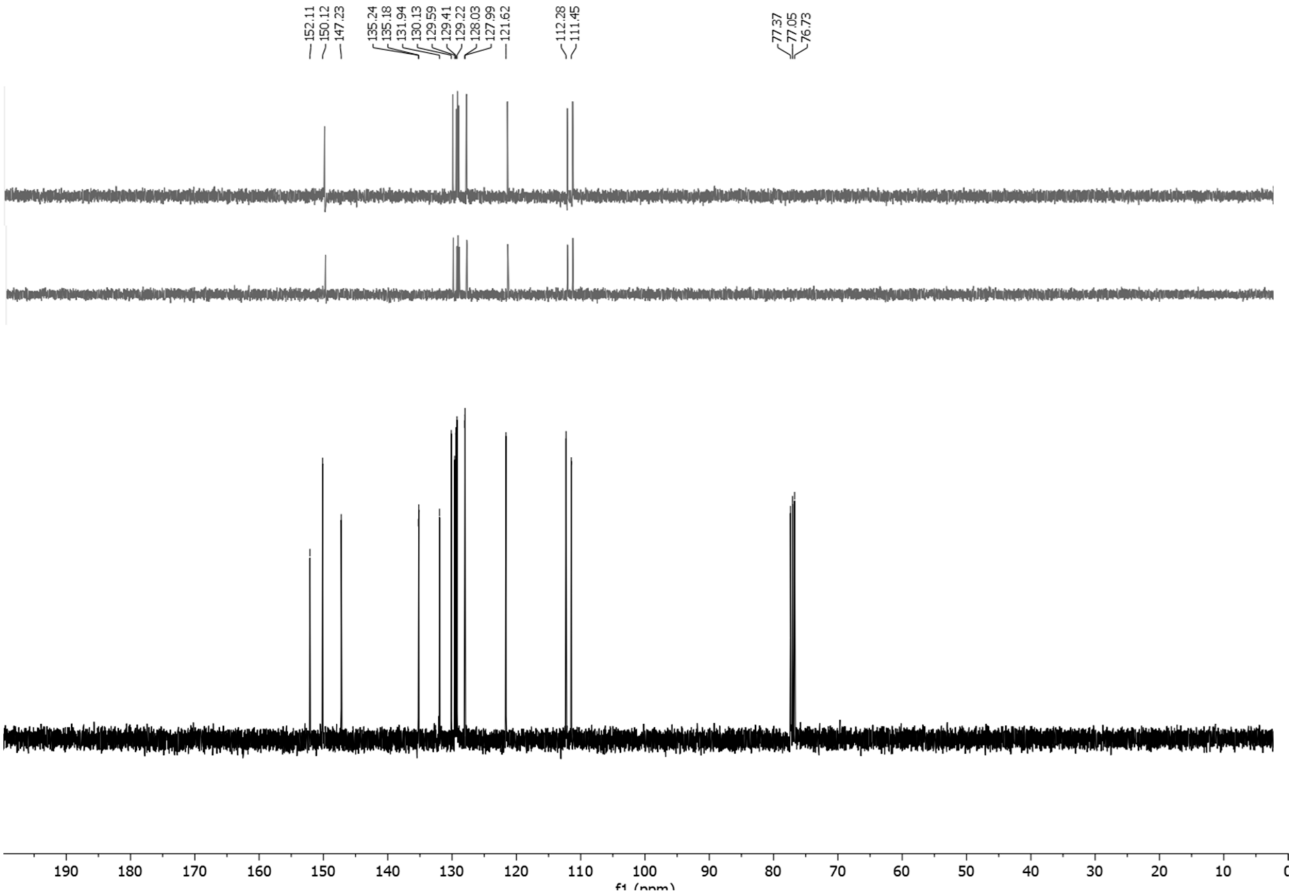

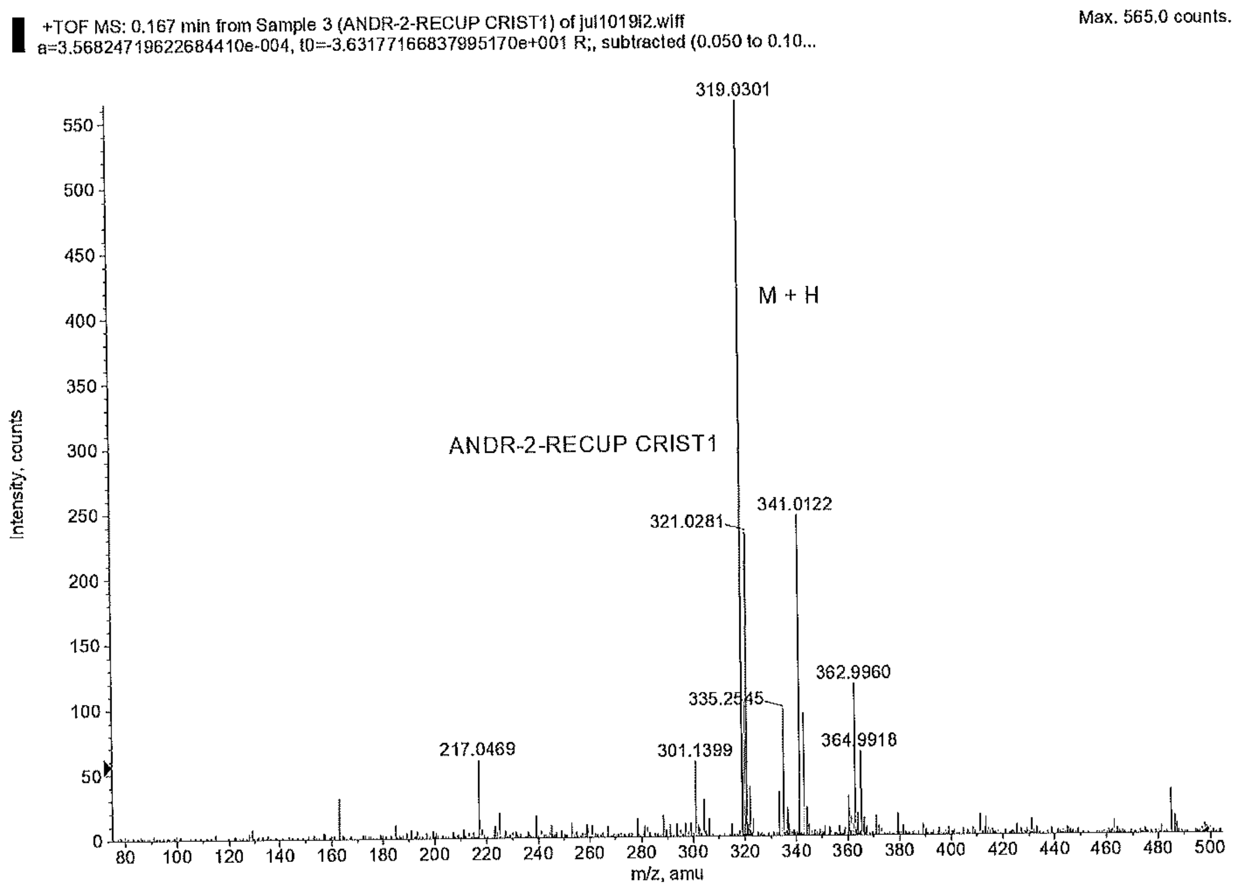

^{1}H, ^{13}C NMR and HRMS spectra of ***N*-(2-chloropyridin-4-yl)naphthalene-2-sulfonamide (26)**

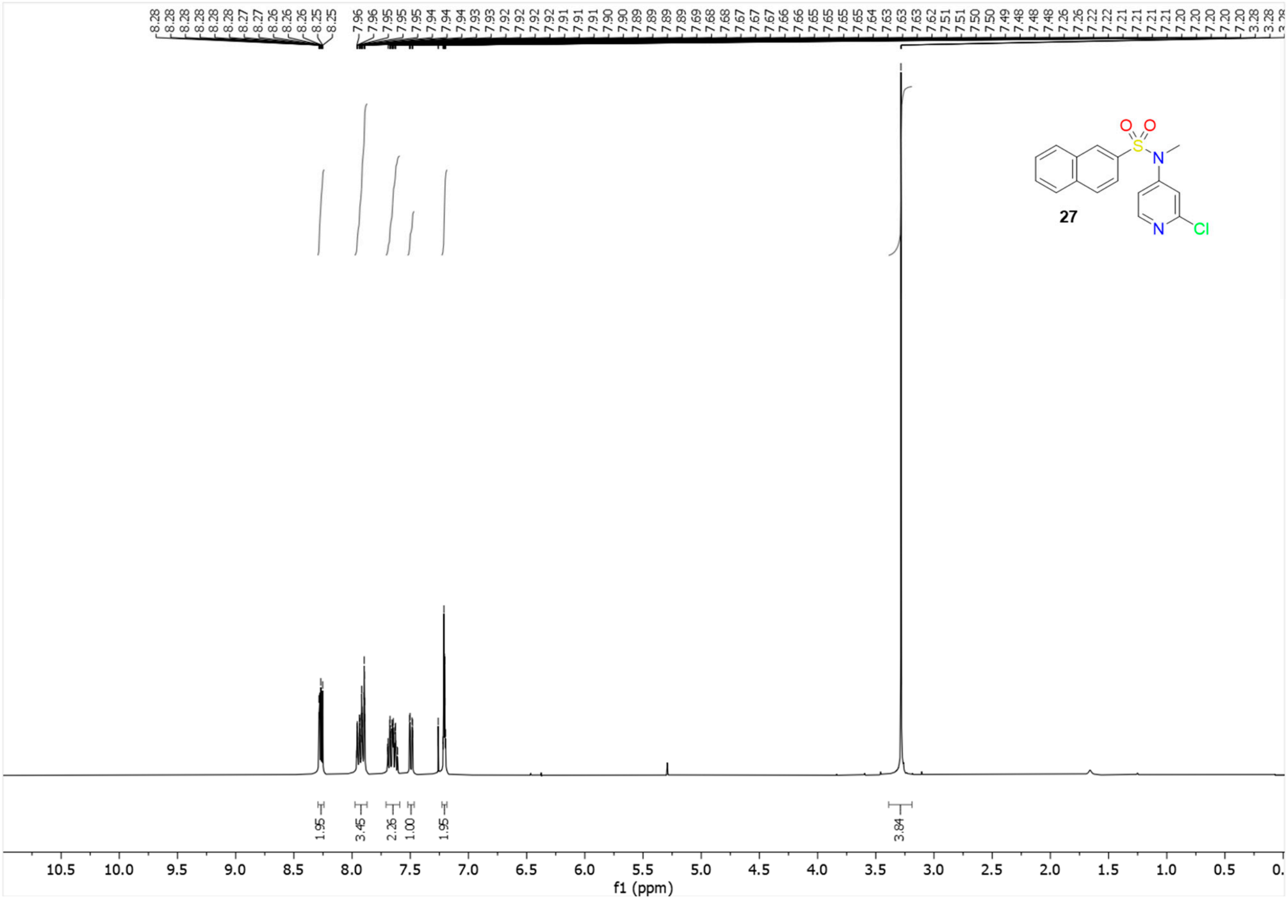
27
O O
S
N
N
Cl
1.95
3.45
2.26
1.00
1.95
3.84
f1 (ppm)

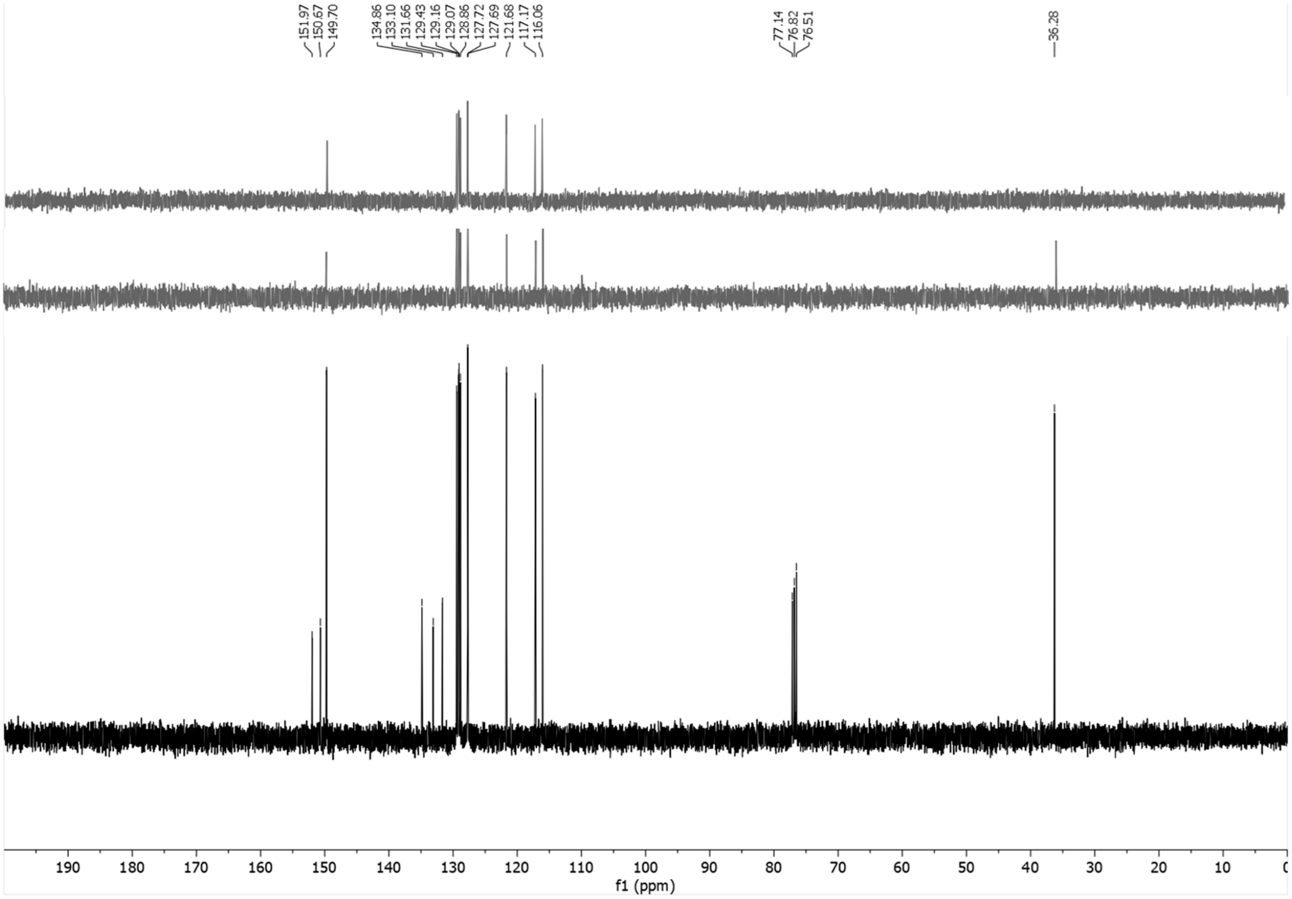
151.97
150.67
149.70
134.86
133.10
131.66
129.43
129.16
129.07
128.86
127.72
127.69
121.68
117.17
116.06
77.14
76.82
76.51
36.28
f1 (ppm)

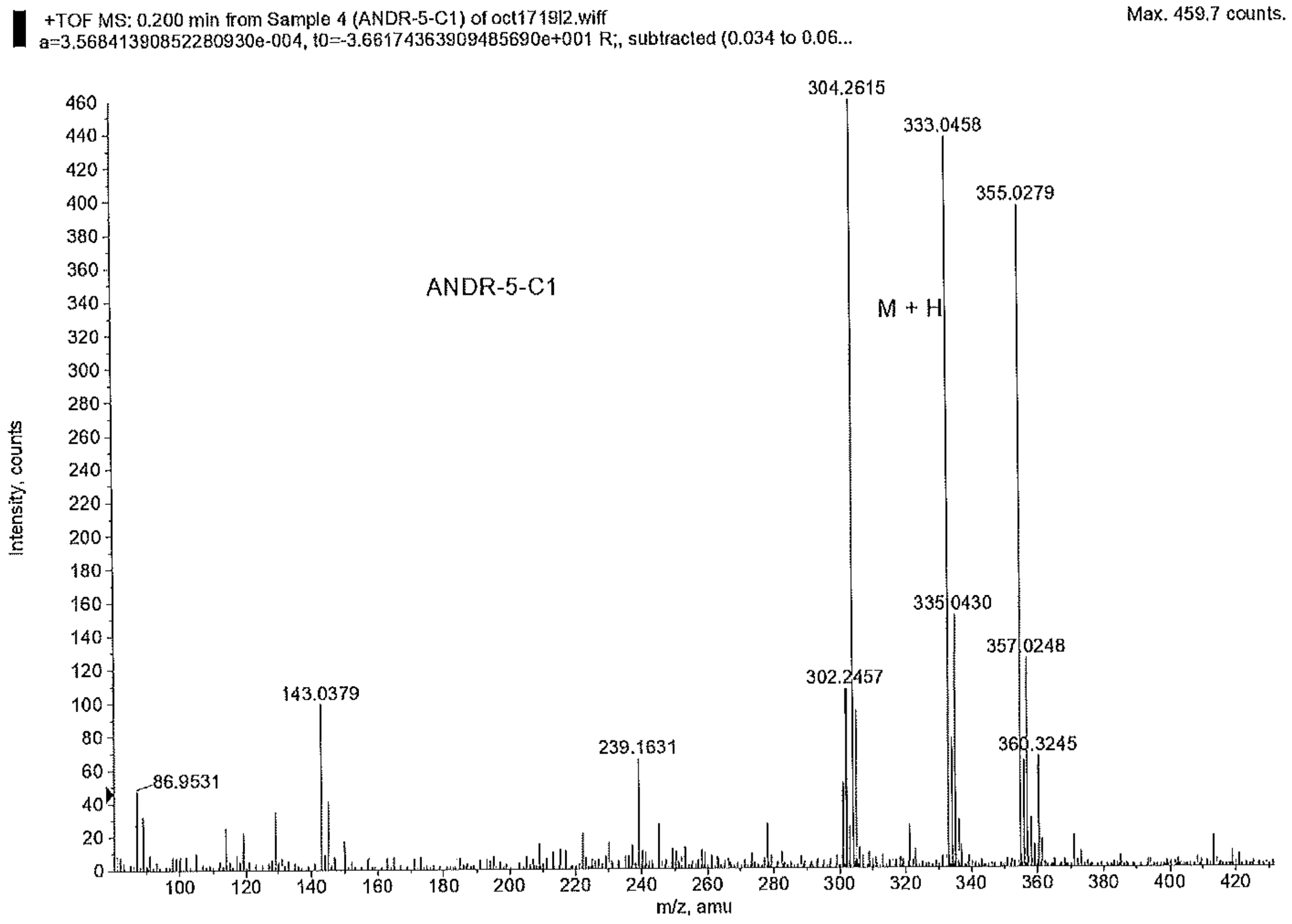

^{1}H, ^{13}C NMR and HRMS spectra of ***N*-(2-chloropyridin-4-yl)-*N*-methylnaphthalene-2-sulfonamide (27)**

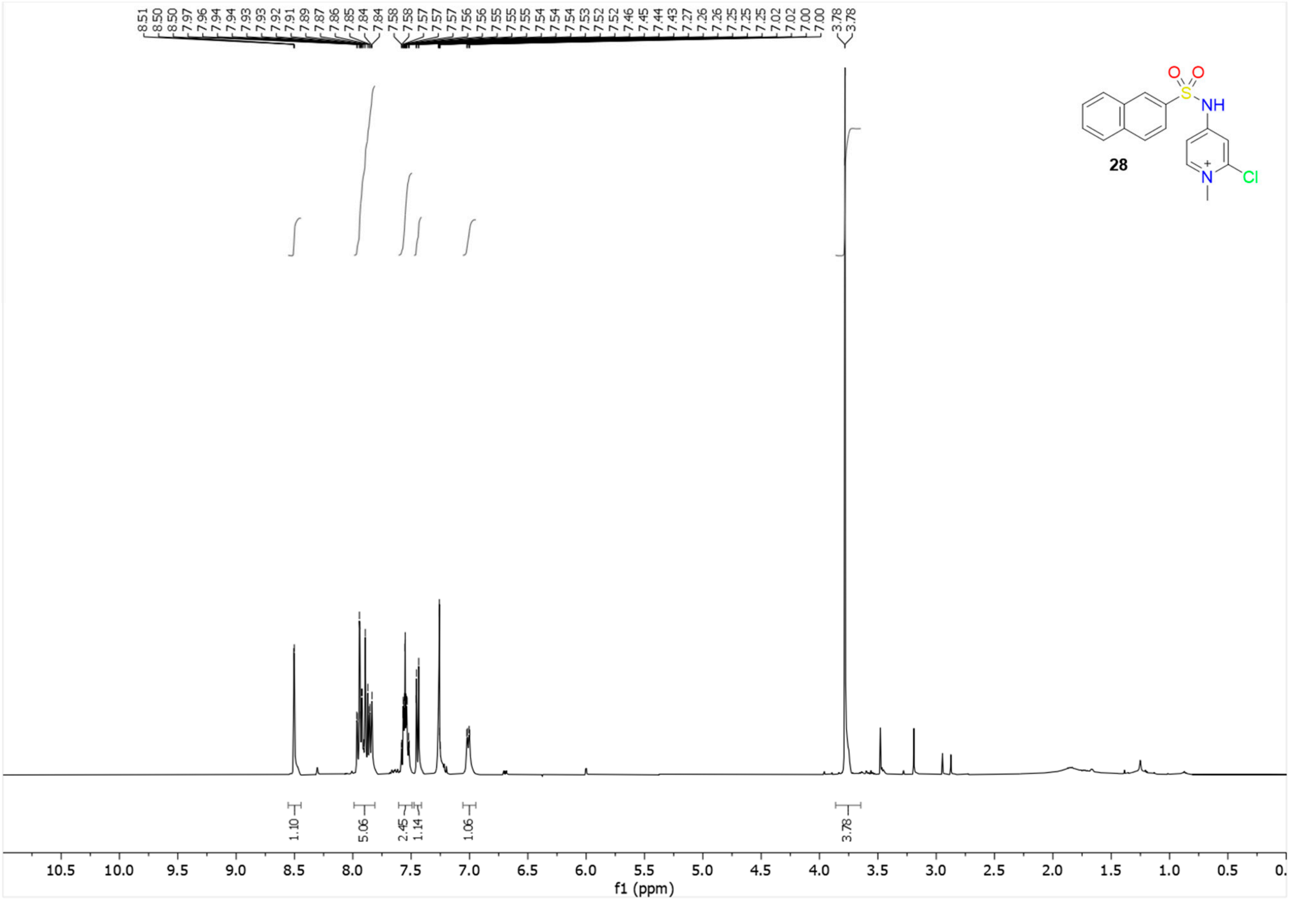

O
O
S
NH
+
N
Cl
28
1.10
5.06
2.45
1.14
1.06
3.78
f1 (ppm)

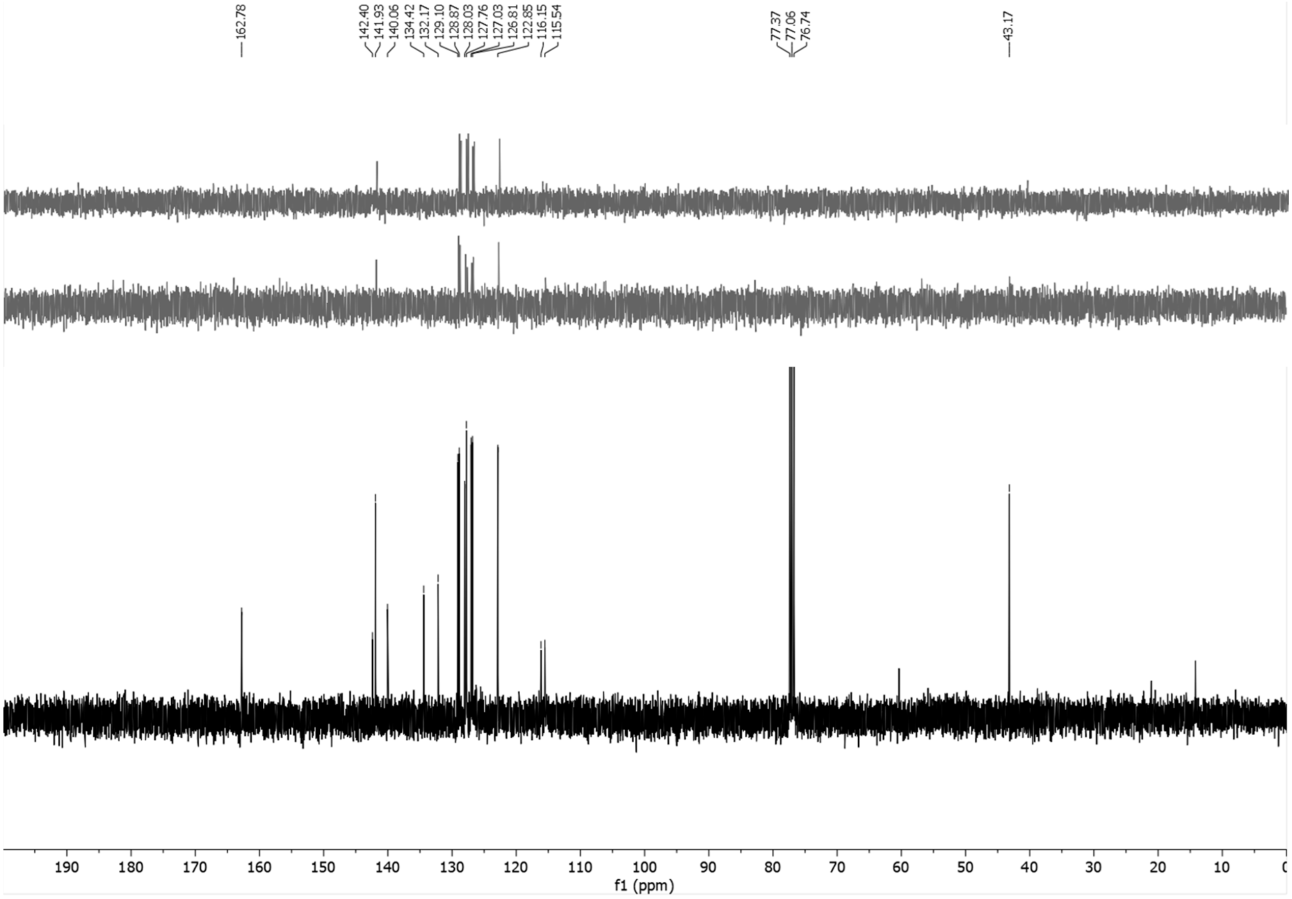

162.78
142.40
141.93
140.06
134.42
132.17
129.10
128.87
128.03
127.76
127.03
126.81
122.85
116.15
115.54
77.37
77.06
76.74
43.17
f1 (ppm)

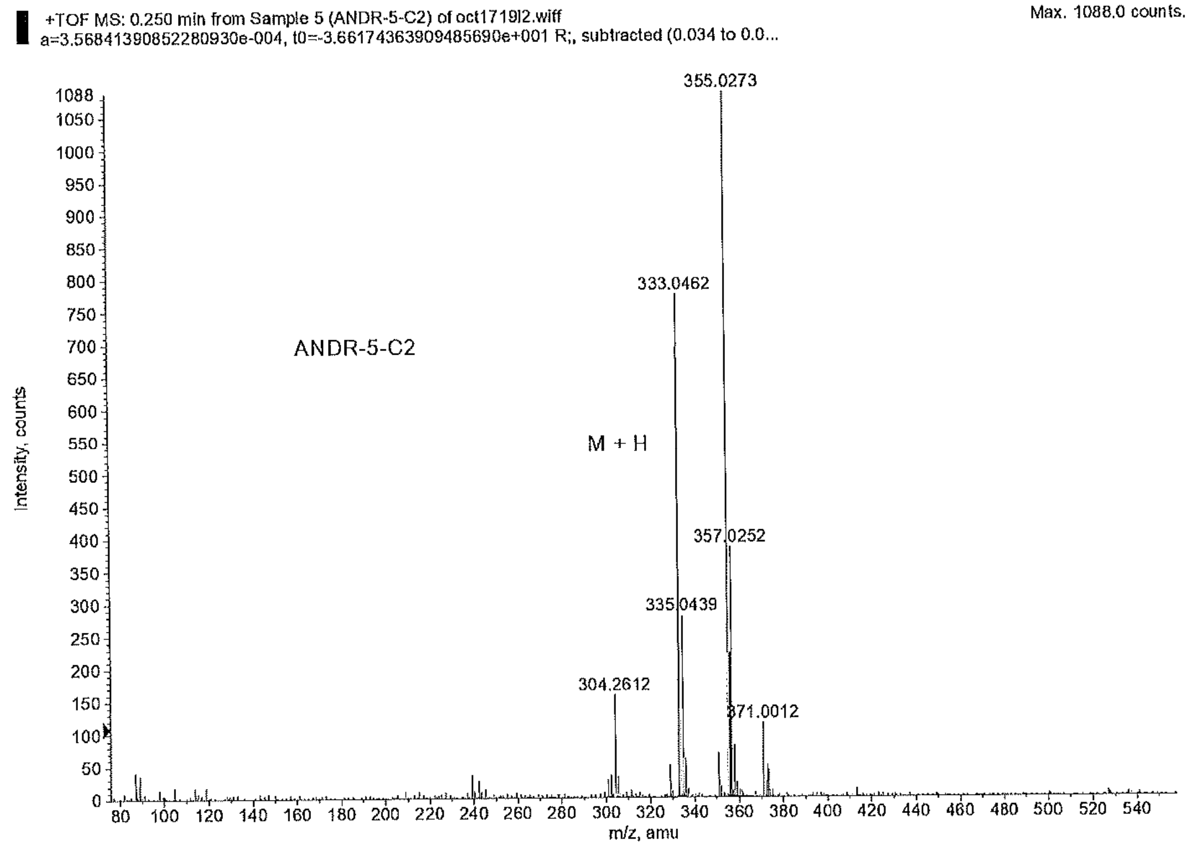

¹H, ¹³C NMR and HRMS spectra of **2-chloro-1-methyl-4-(naphthalene-2-sulfonamido)pyridin-1-ium (28)**

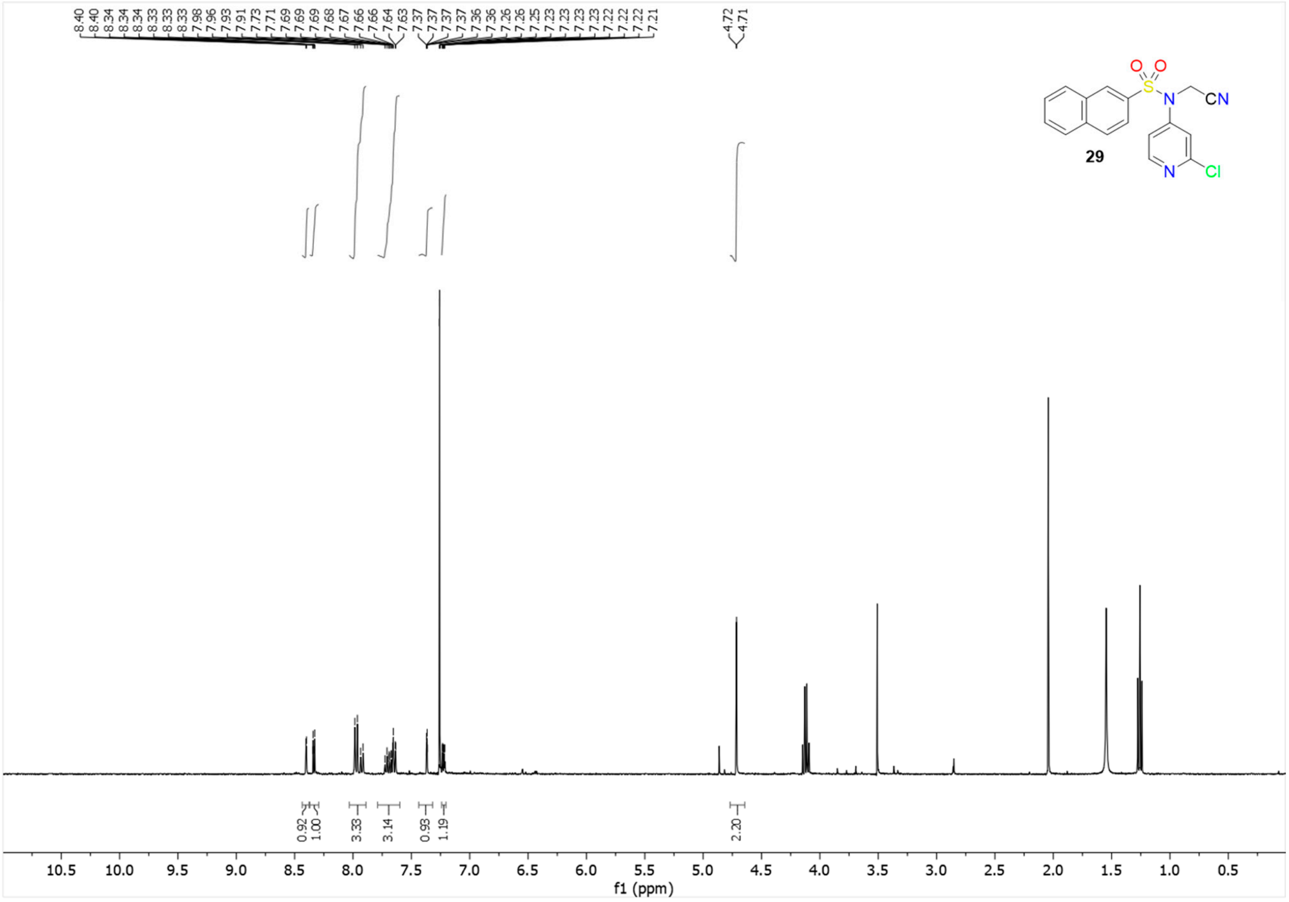

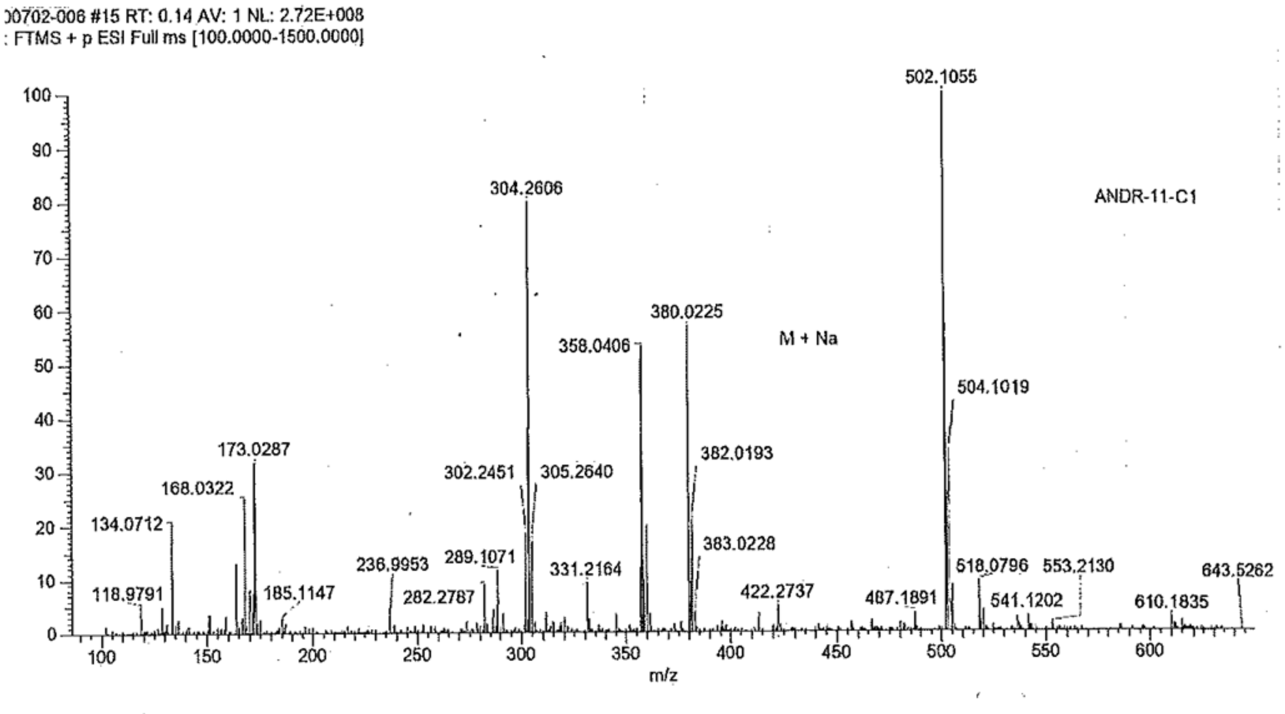

^{1}H and HRMS spectra of ***N*-(2-chloropyridin-4-yl)-*N*-(cyanomethyl)naphthalene-2-sulfonamide (29)**

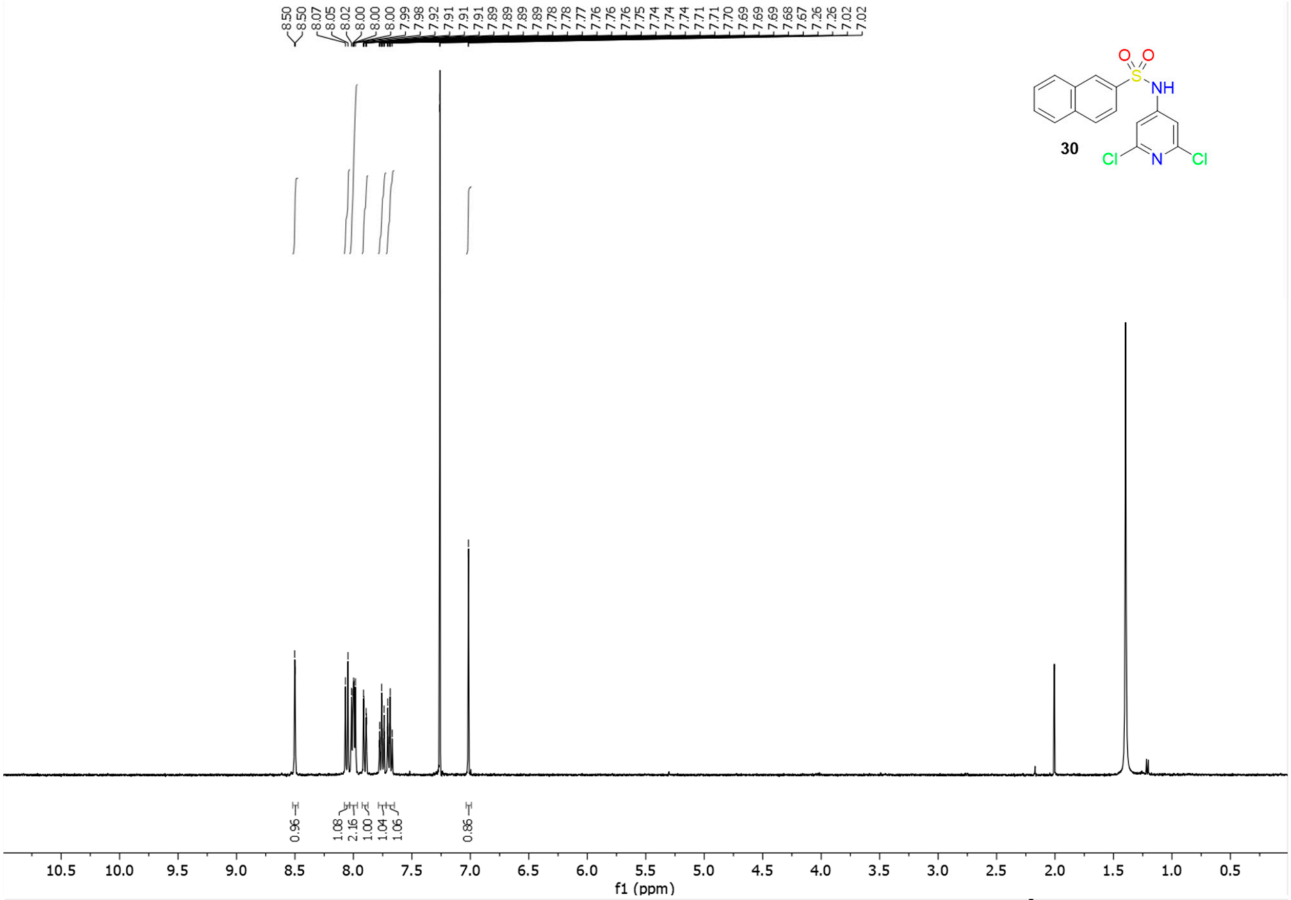
8.50
8.50
8.07
8.05
8.02
8.00
8.00
8.00
7.99
7.98
7.92
7.91
7.91
7.91
7.89
7.89
7.89
7.89
7.78
7.78
7.77
7.76
7.76
7.76
7.75
7.74
7.74
7.74
7.71
7.71
7.70
7.69
7.69
7.69
7.68
7.67
7.26
7.02
7.02
O O
S
NH
N
Cl
Cl
30
0.96
1.08
2.16
1.00
1.04
1.06
0.86
10.5 10.0 9.5 9.0 8.5 8.0 7.5 7.0 6.5 6.0 5.5 5.0 4.5 4.0 3.5 3.0 2.5 2.0 1.5 1.0 0.5
f1 (ppm)

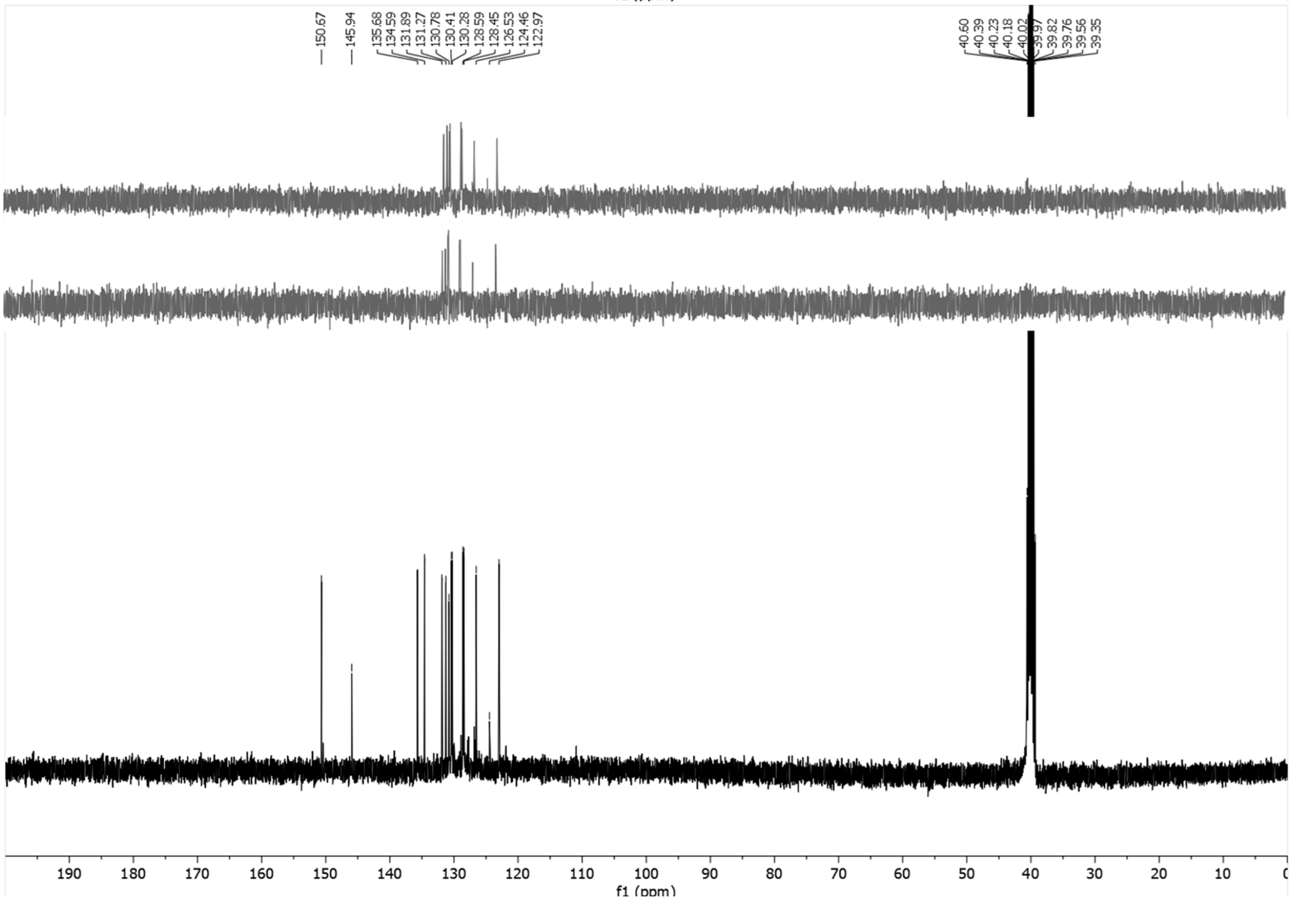
150.67
145.94
135.68
134.59
131.89
131.27
130.78
130.41
130.28
128.59
128.45
126.53
124.46
122.97
40.60
40.39
40.23
40.18
40.02
39.97
39.82
39.76
39.56
39.35
190 180 170 160 150 140 130 120 110 100 90 80 70 60 50 40 30 20 10
f1 (ppm)

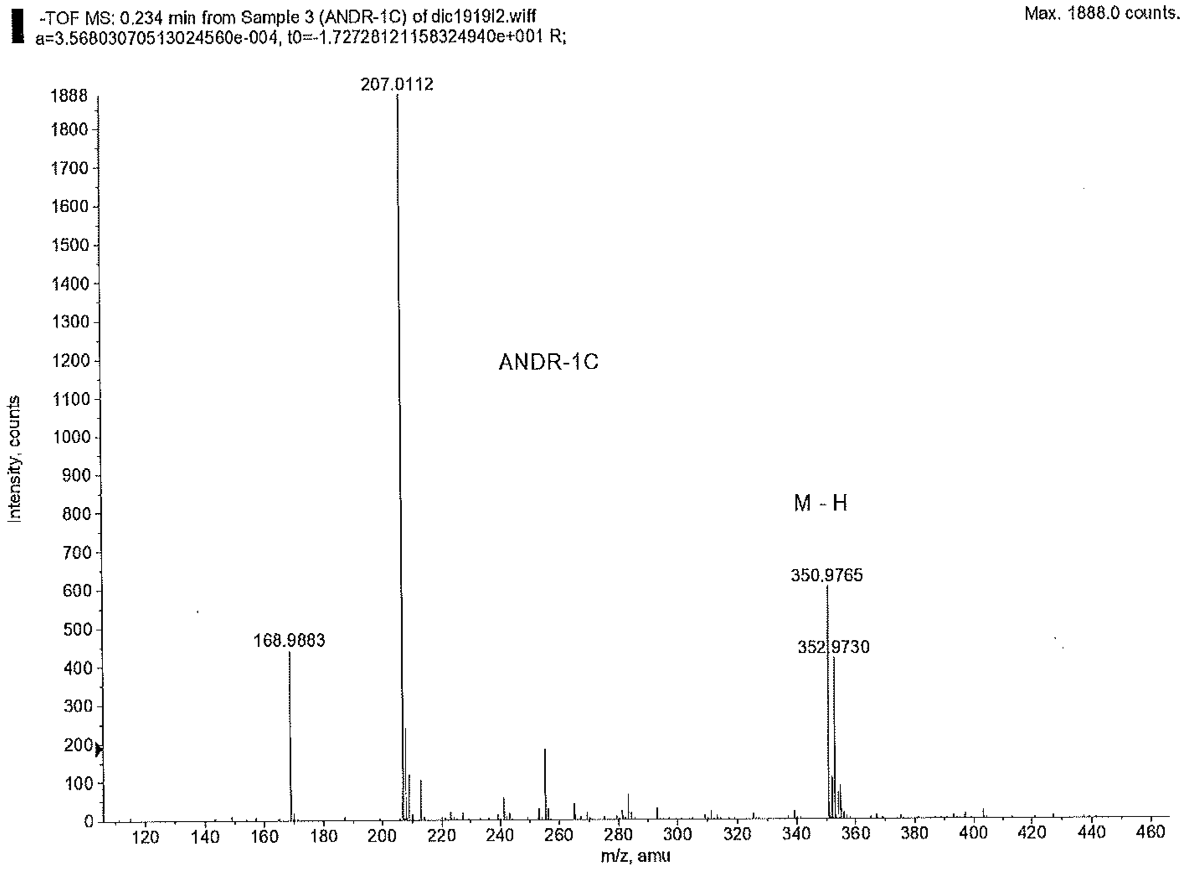

^{1}H, ^{13}C NMR and HRMS spectra of ***N*-(2,6-dichloropyridin-4-yl)naphthalene-2-sulfonamide (30)**

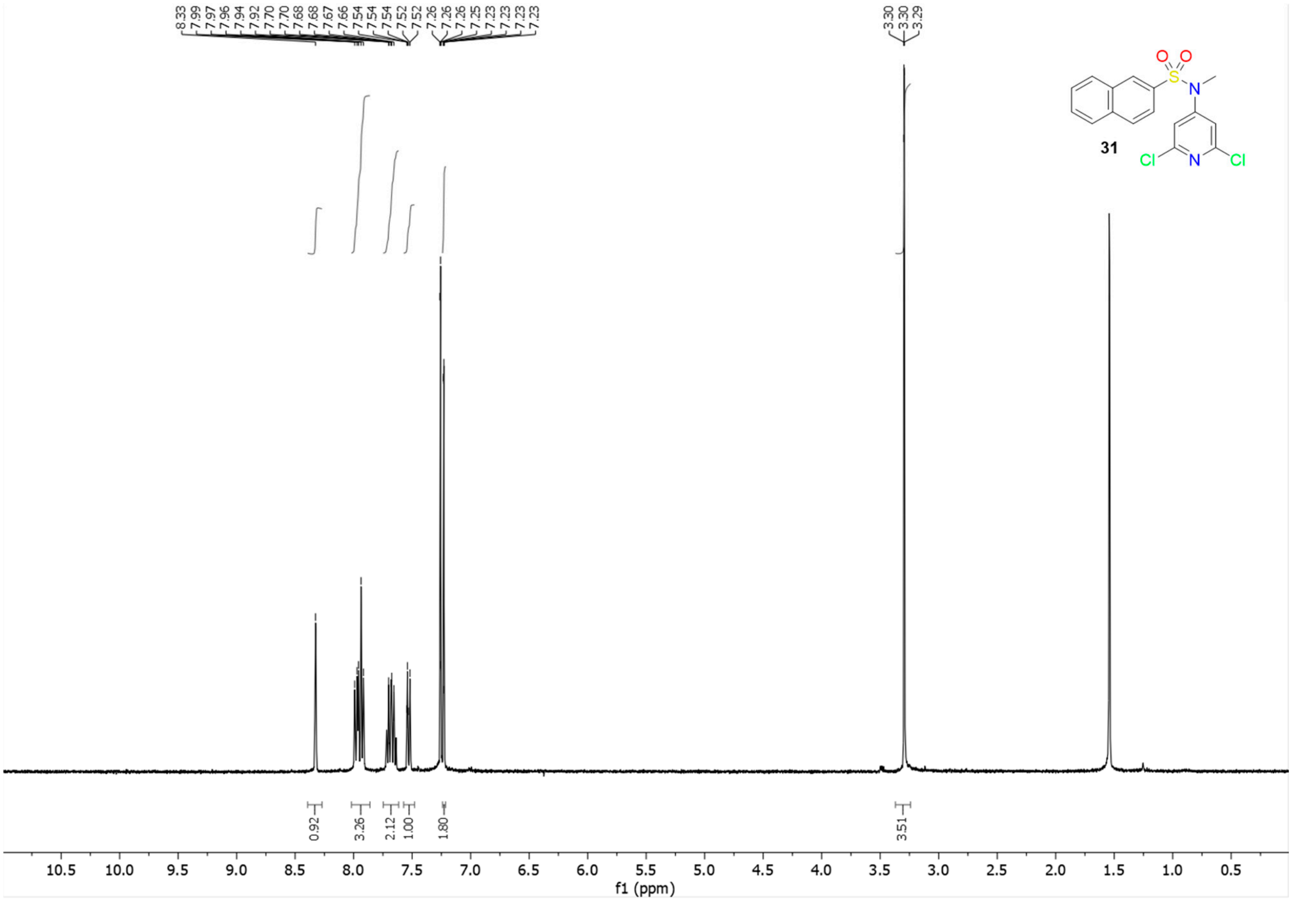
8.33 7.99 7.97 7.96 7.94 7.92 7.70 7.70 7.68 7.68 7.67 7.66 7.54 7.54 7.54 7.54 7.52 7.52 7.26 7.26 7.26 7.25 7.23 7.23 7.23 7.23
3.30 3.30 3.29
31
Cl
N
Cl
O
O
S
N
0.92
3.26
2.12
1.00
1.80
3.51
f1 (ppm)

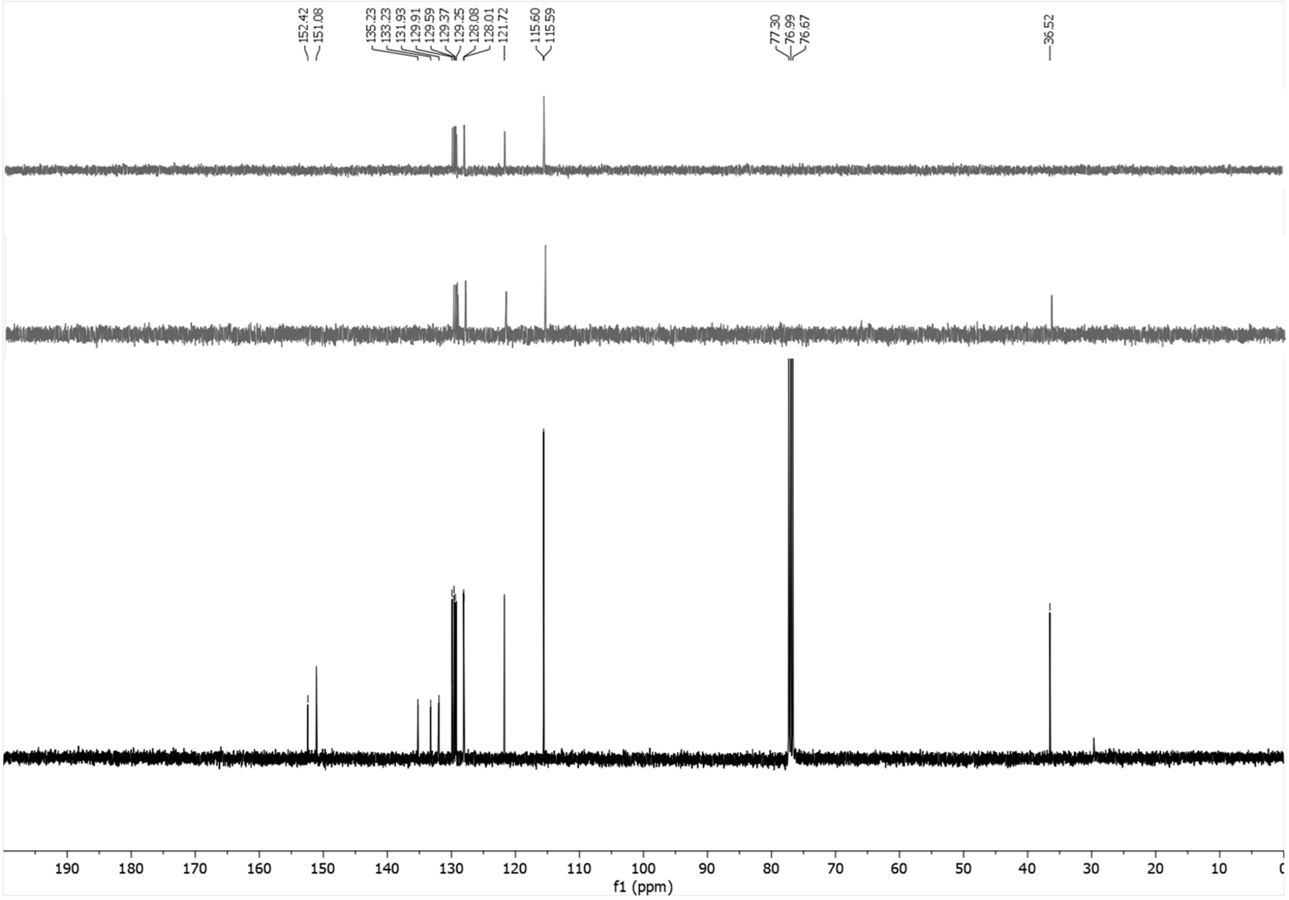
152.42 151.08
135.23 133.23 131.93 129.91 129.59 129.37 129.25 128.08 128.01 121.72
115.60 115.59
77.30 76.99 76.67
36.52
f1 (ppm)

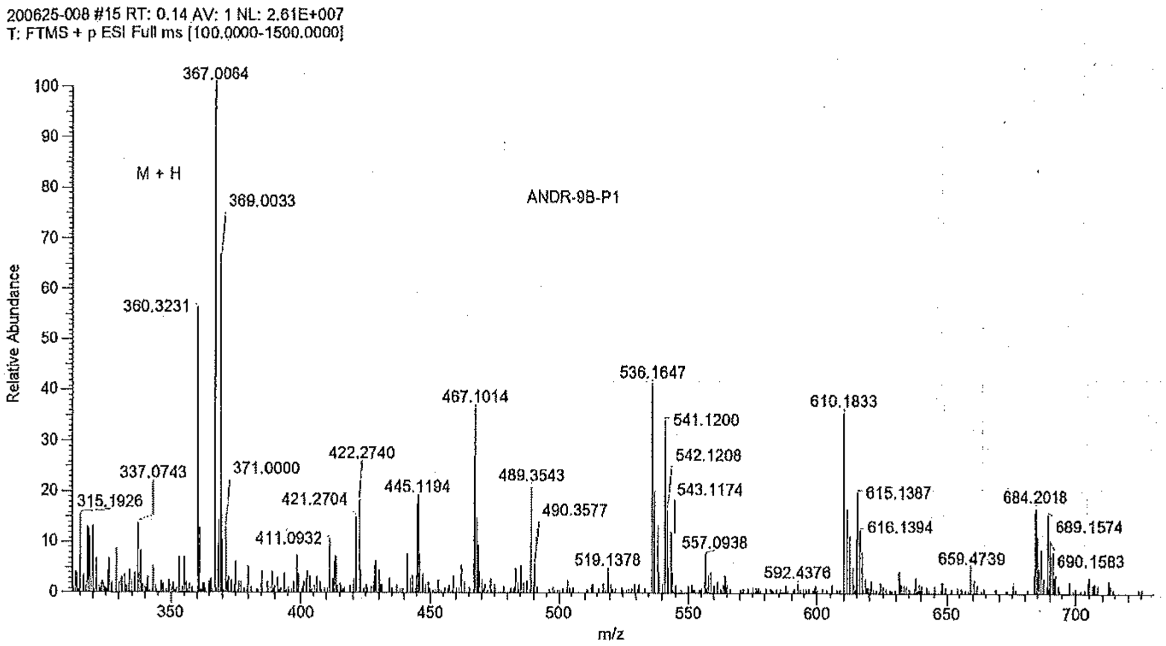

^{1}H, ^{13}C NMR and HRMS spectra of *N*-**(2,6-dichloropyridin-4-yl)**-*N*-**methylnaphthalene-2-sulfonamide (31)**

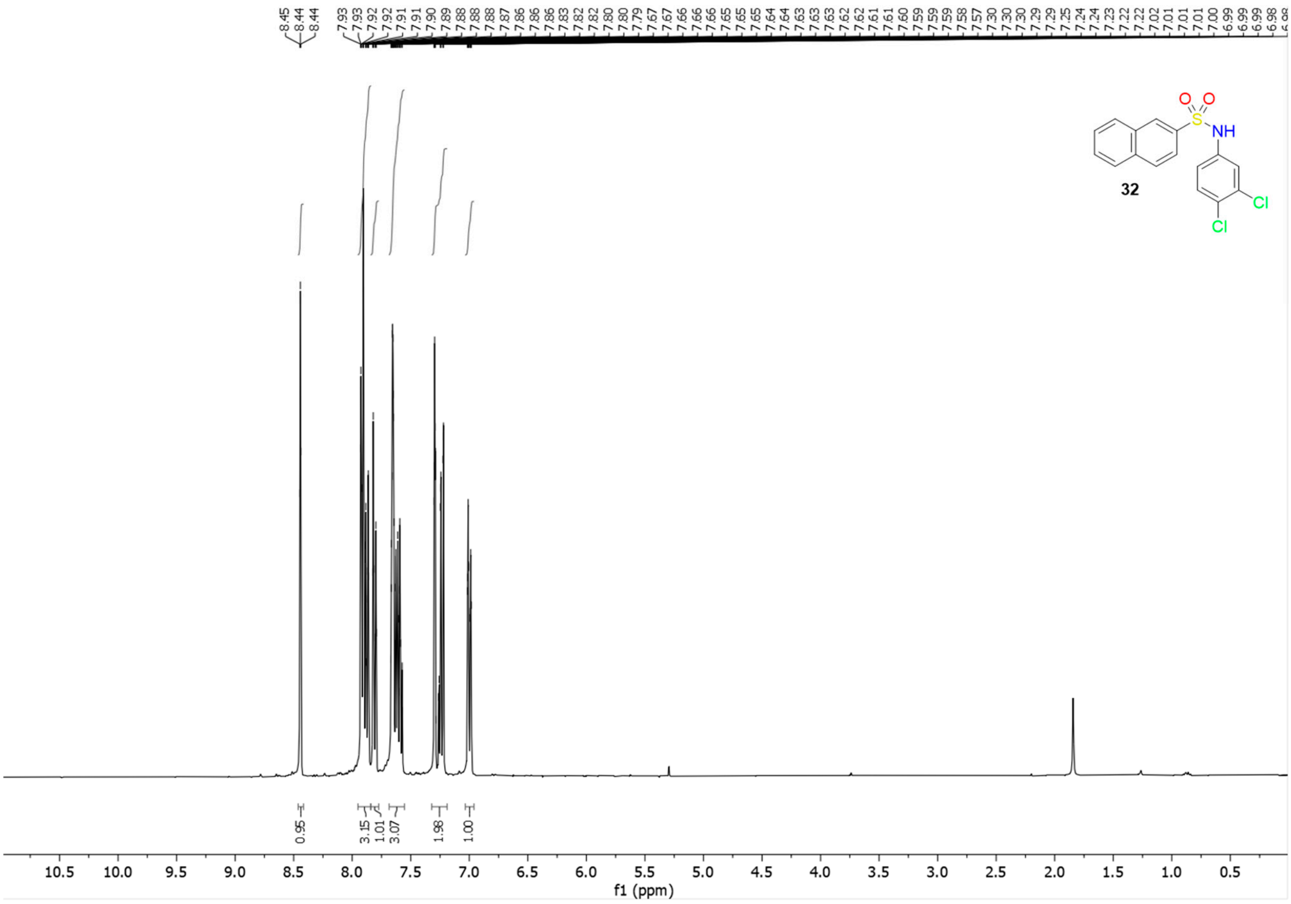
32
NH
Cl
Cl
f1 (ppm)

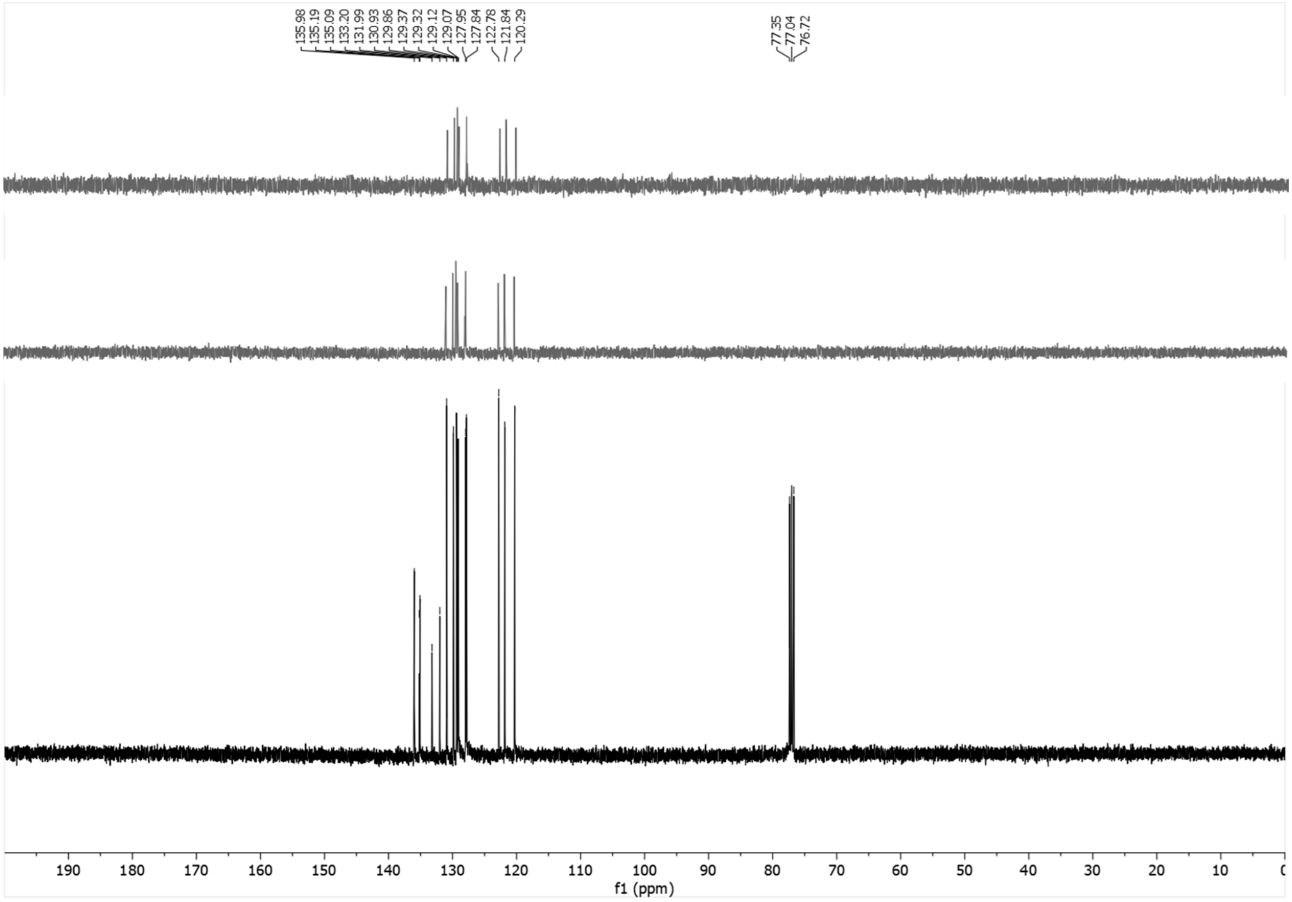
f1 (ppm)

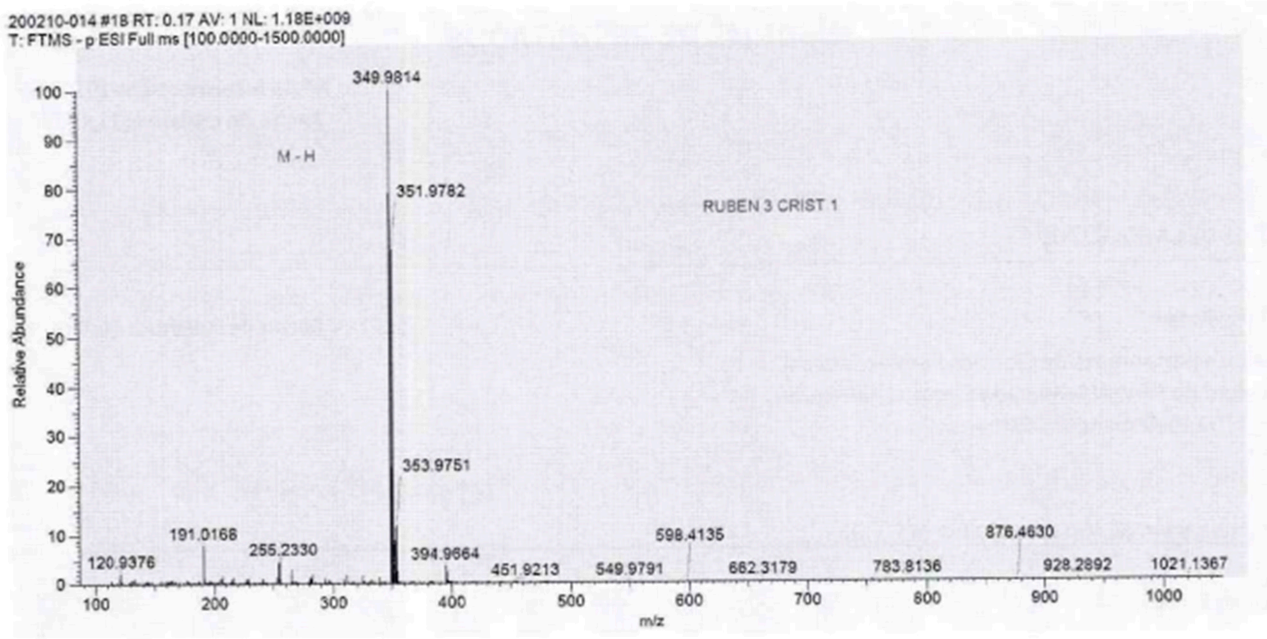

^{1}H, ^{13}C NMR and HRMS spectra of ***N*-(3,4-dichlorophenyl)naphthalene-2-sulfonamide (32)**

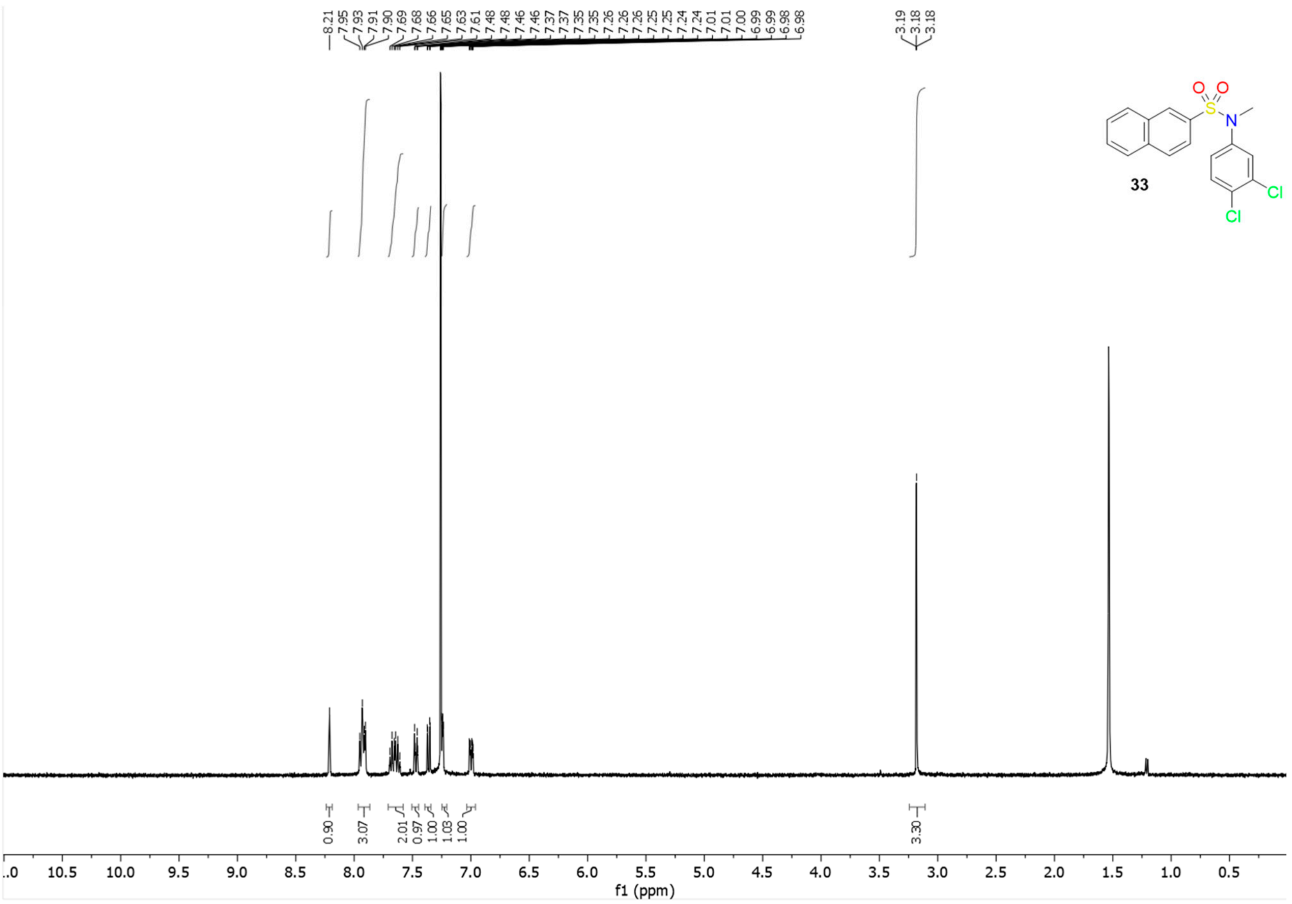
8.21
7.95
7.93
7.91
7.90
7.69
7.68
7.66
7.65
7.63
7.61
7.48
7.46
7.37
7.37
7.35
7.35
7.26
7.26
7.25
7.25
7.24
7.24
7.01
7.01
7.00
6.99
6.98
6.98
3.19
3.18
3.18
33
Cl
Cl
0.90
3.07
2.01
0.97
1.00
1.03
1.00
3.30
f1 (ppm)

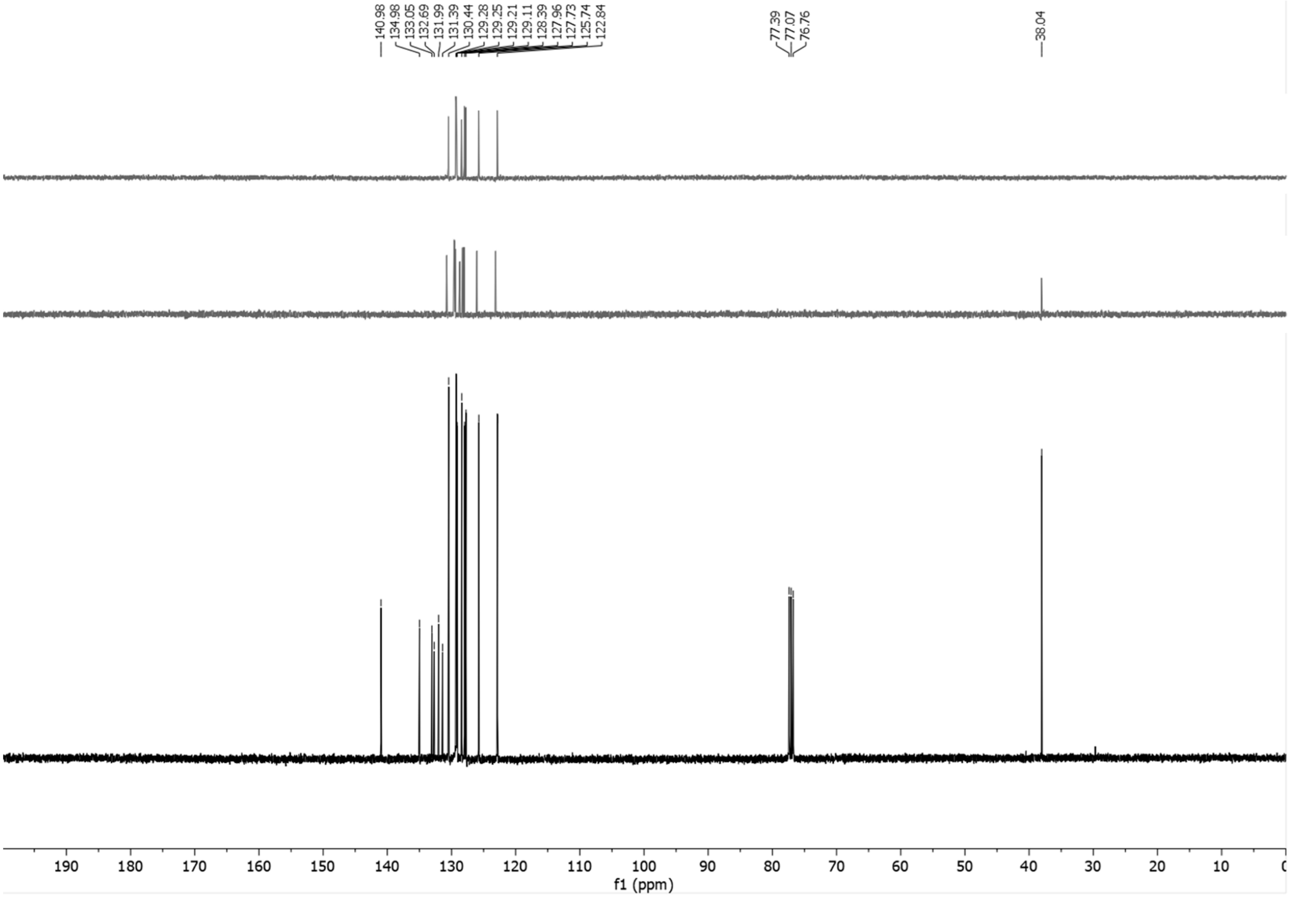
140.98
134.98
133.05
132.69
131.99
131.39
130.44
129.28
129.25
129.21
129.11
128.39
127.96
127.73
125.74
122.84
77.39
77.07
76.76
38.04
f1 (ppm)

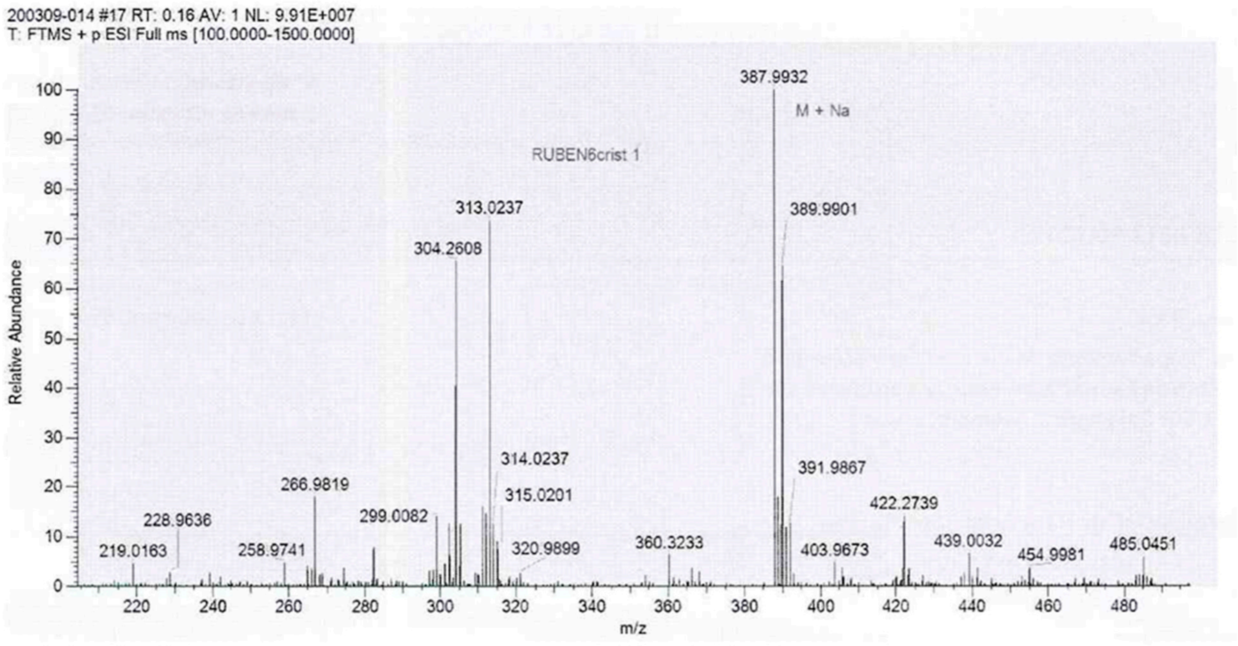

^{1}H, ^{13}C NMR and HRMS spectra of ***N*-(3,4-dichlorophenyl)-*N*-methylnaphthalene-2-sulfonamide (33)**

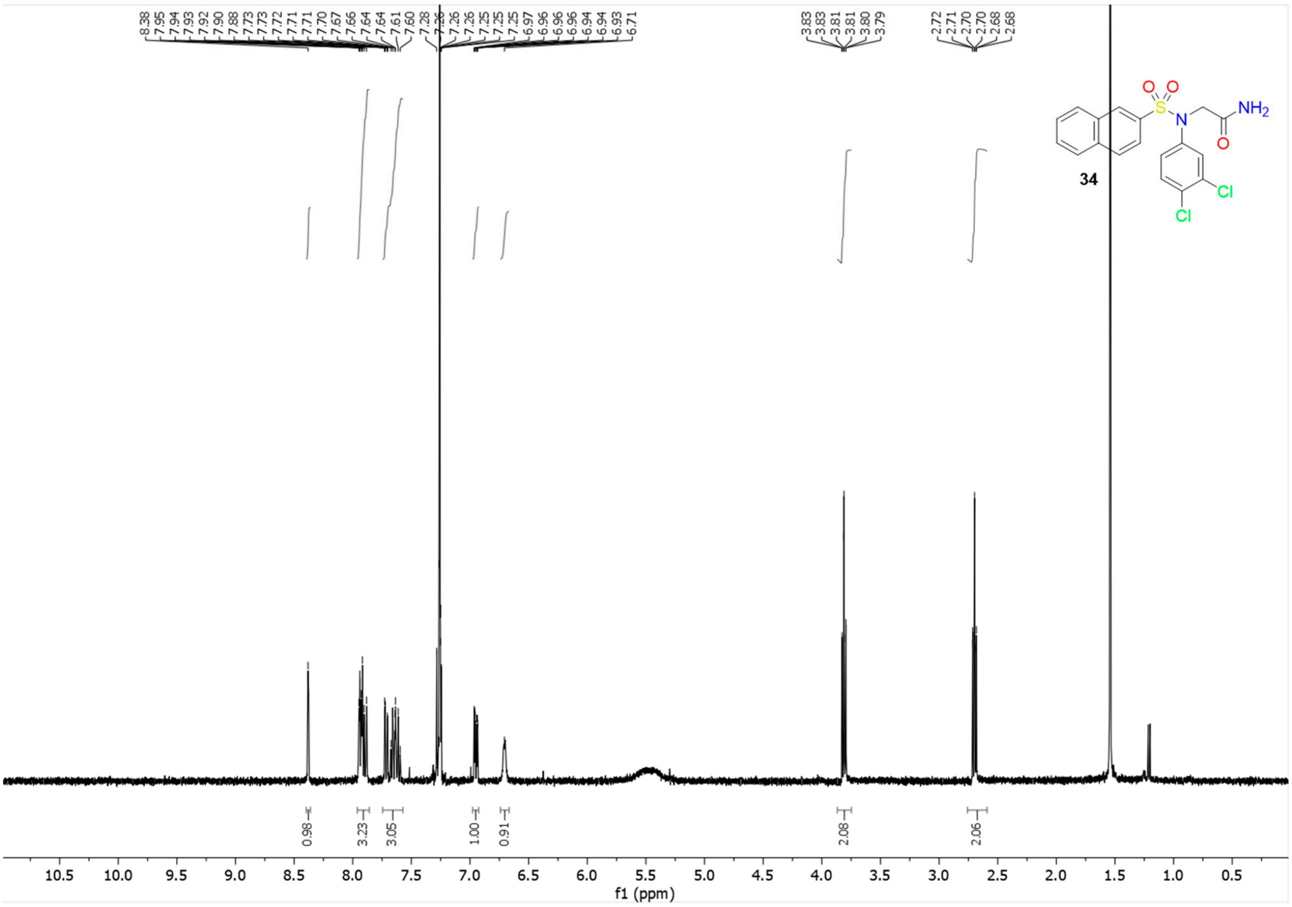

8.38 7.95 7.94 7.93 7.92 7.90 7.88 7.73 7.73 7.72 7.71 7.71 7.70 7.67 7.66 7.64 7.64 7.61 7.60 7.28 7.26 7.26 7.26 7.25 7.25 7.25 6.97 6.96 6.96 6.96 6.94 6.94 6.93 6.71
3.83 3.83 3.81 3.81 3.80 3.79
2.72 2.71 2.70 2.70 2.68 2.68
0.98 3.23 3.05 1.00 0.91 2.08 2.06
NH2
Cl
Cl
34
10.5 10.0 9.5 9.0 8.5 8.0 7.5 7.0 6.5 6.0 5.5 5.0 4.5 4.0 3.5 3.0 2.5 2.0 1.5 1.0 0.5
f1 (ppm)

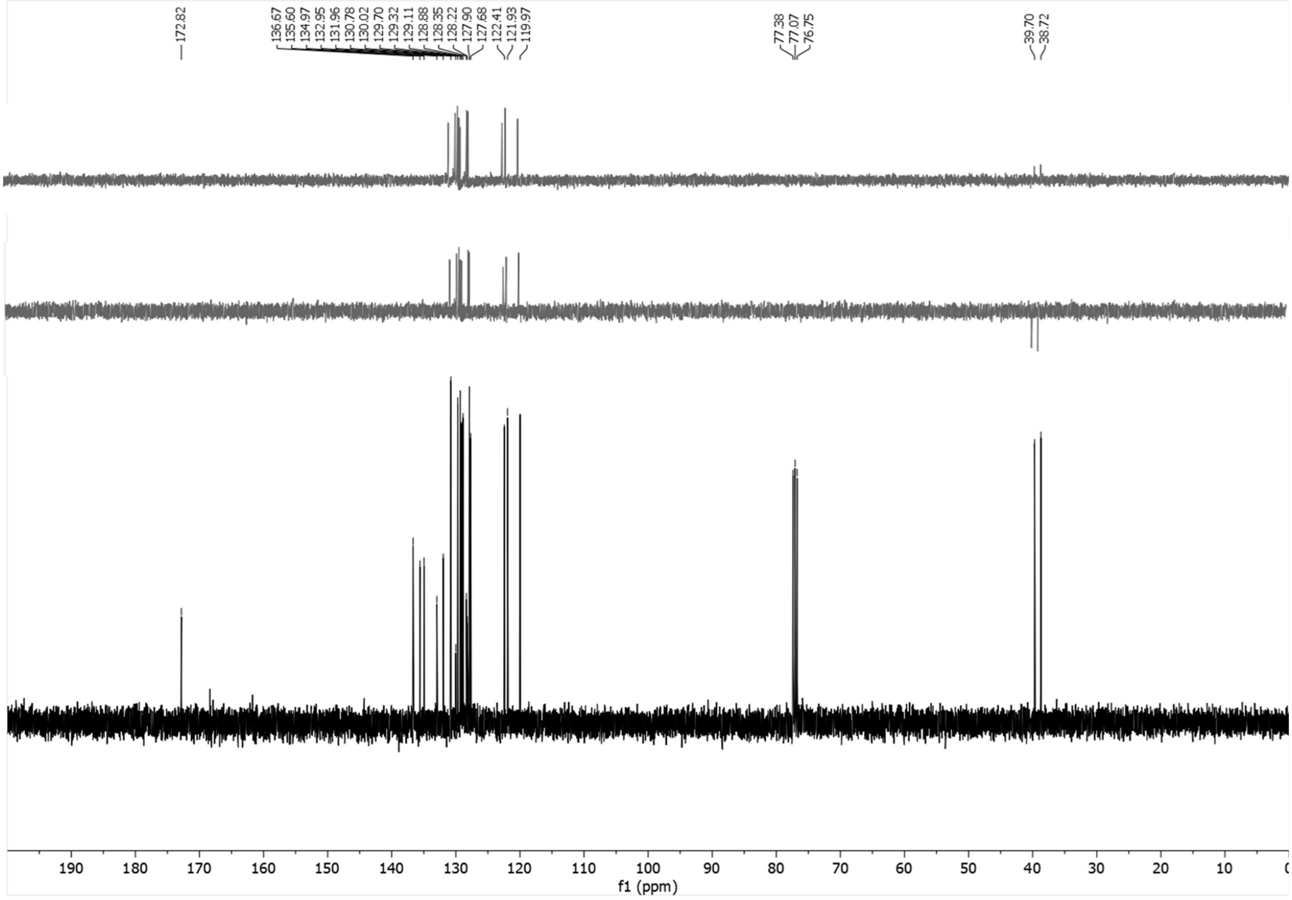

172.82
136.67 135.60 134.97 132.95 131.96 130.78 130.02 129.70 129.32 129.11 128.88 128.35 128.22 127.90 127.68 122.41 121.93 119.97
77.38 77.07 76.75
39.70 38.72
190 180 170 160 150 140 130 120 110 100 90 80 70 60 50 40 30 20 10
f1 (ppm)

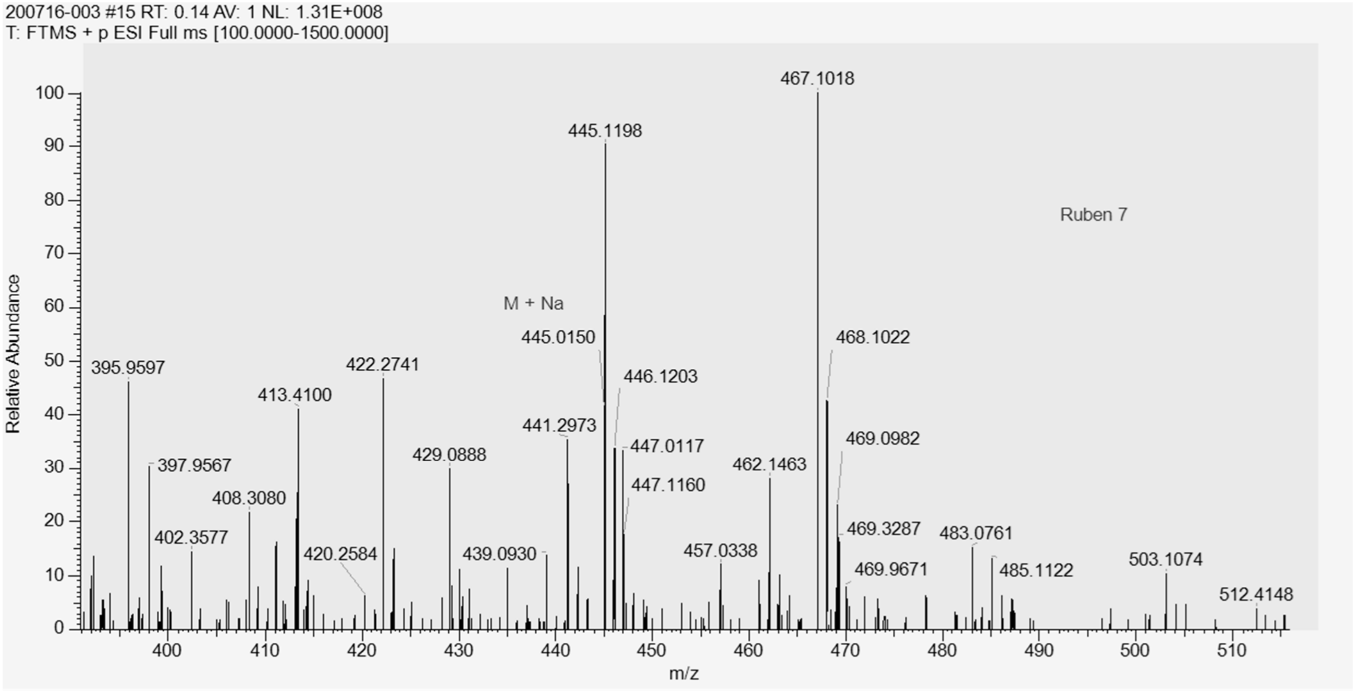

^{1}H, ^{13}C NMR and HRMS spectra of **3-(*N*-(3,4-dichlorophenyl)naphthalene-2-sulfonamido)propanamide (34)**

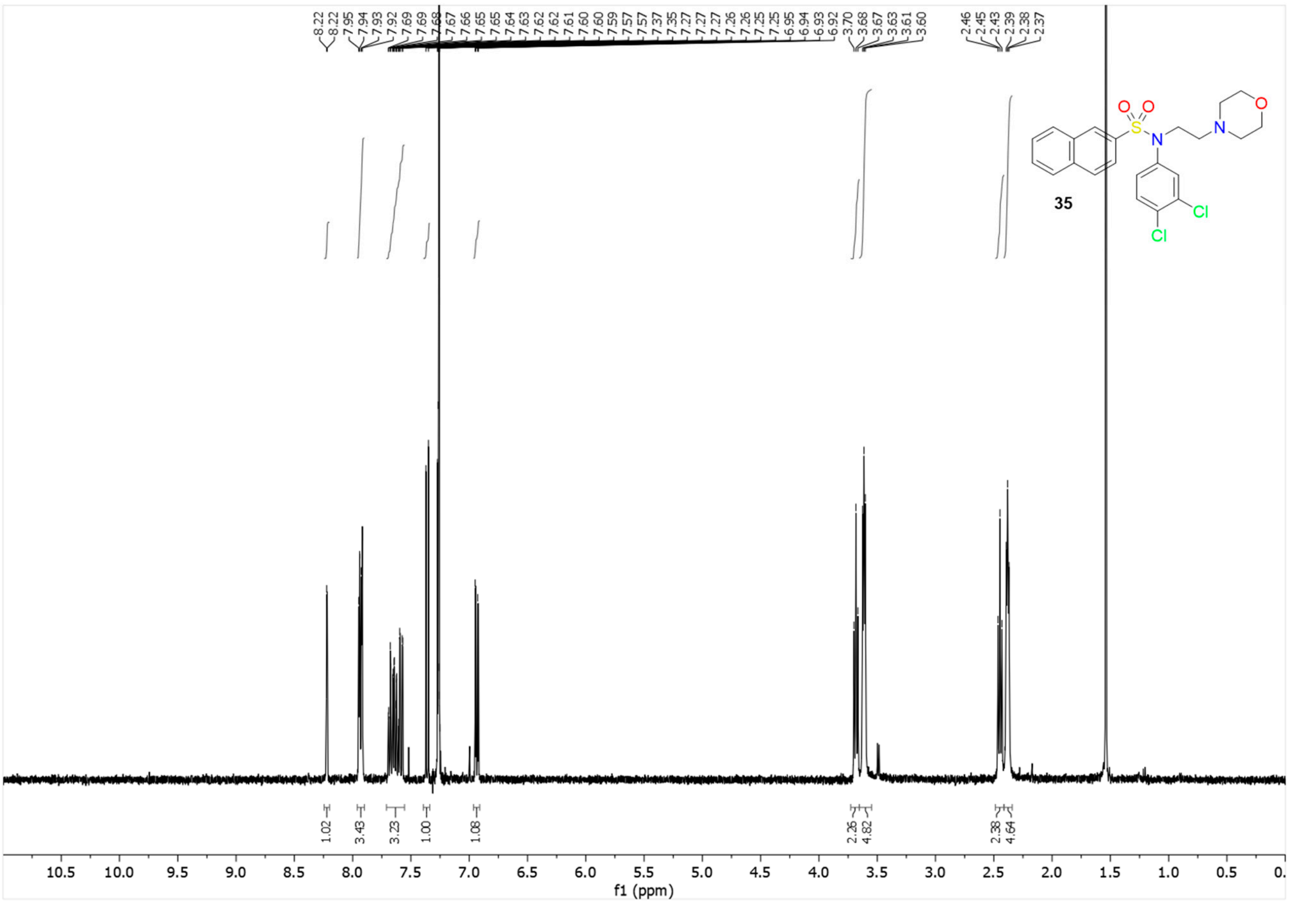
8.22
8.22
7.95
7.94
7.93
7.92
7.69
7.69
7.68
7.67
7.66
7.65
7.65
7.64
7.63
7.62
7.62
7.61
7.60
7.60
7.59
7.57
7.57
7.37
7.35
7.27
7.27
7.27
7.26
7.26
7.25
7.25
6.95
6.94
6.93
6.92
3.70
3.68
3.67
3.63
3.61
3.60
2.46
2.45
2.43
2.39
2.38
2.37
O
O
S
N
N
O
Cl
Cl
35
1.02
3.43
3.29
1.00
1.08
2.26
4.82
2.38
4.64
10.5
10.0
9.5
9.0
8.5
8.0
7.5
7.0
6.5
6.0
5.5
5.0
4.5
4.0
3.5
3.0
2.5
2.0
1.5
1.0
0.5
f1 (ppm)

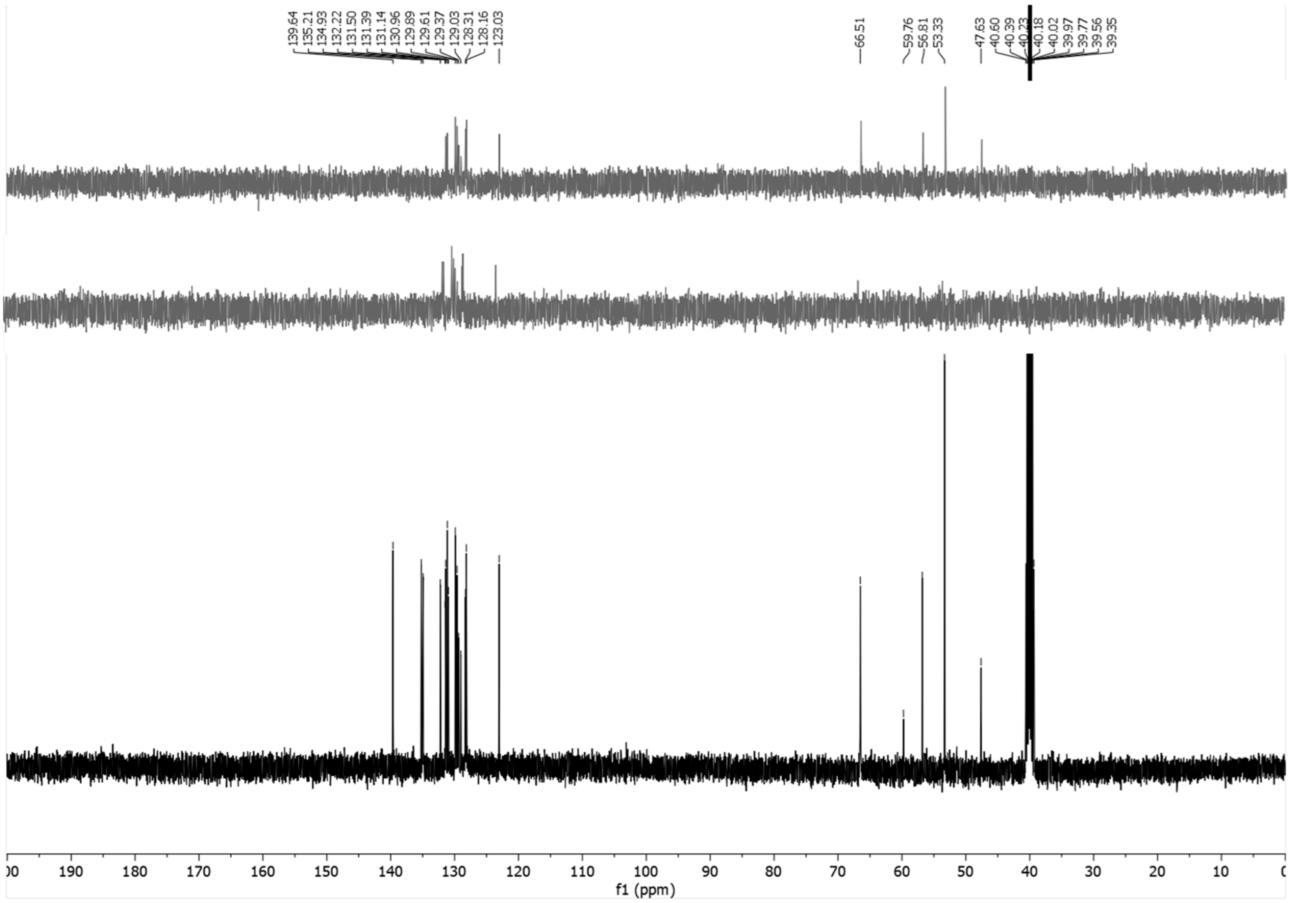
139.64
135.21
134.93
132.22
131.50
131.39
131.14
130.96
129.89
129.61
129.37
129.03
128.31
128.16
123.03
66.51
59.76
56.81
53.33
47.63
40.60
40.39
40.23
40.18
40.02
39.97
39.77
39.56
39.35
190
180
170
160
150
140
130
120
110
100
90
80
70
60
50
40
30
20
10
f1 (ppm)

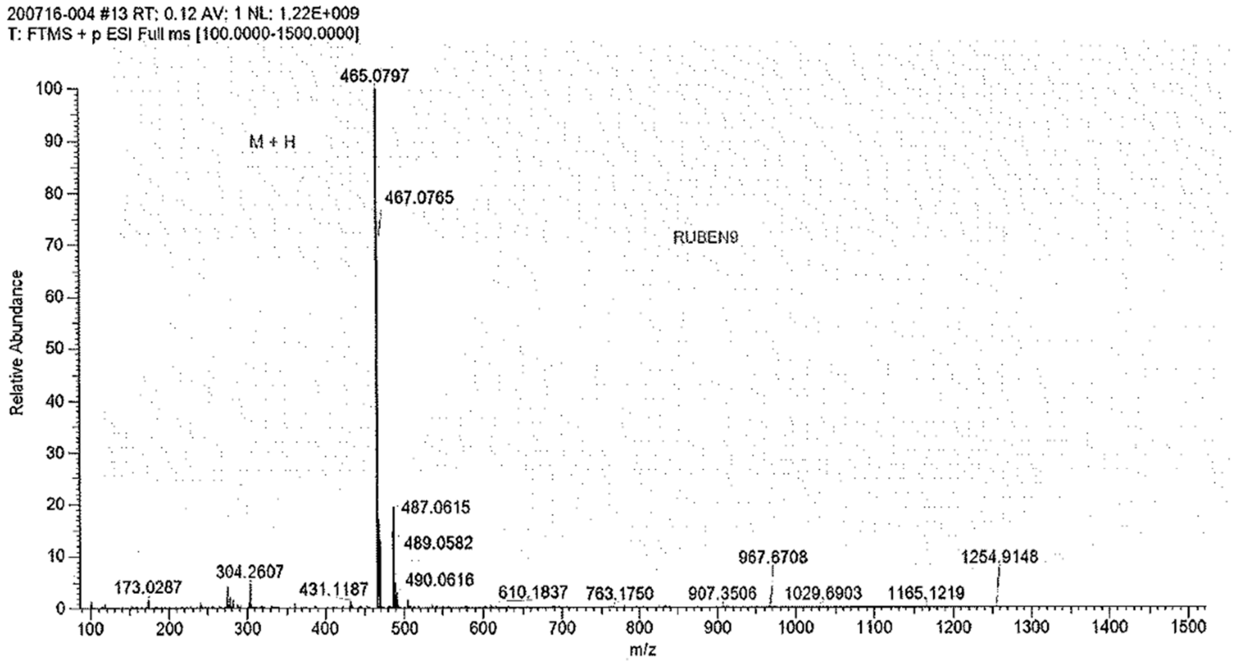

^{1}H, ^{13}C NMR and HRMS spectra of ***N*-(3,4-dichlorophenyl)-*N*-(2-morpholinoethyl)naphthalene-2-sulfonamide (35)**

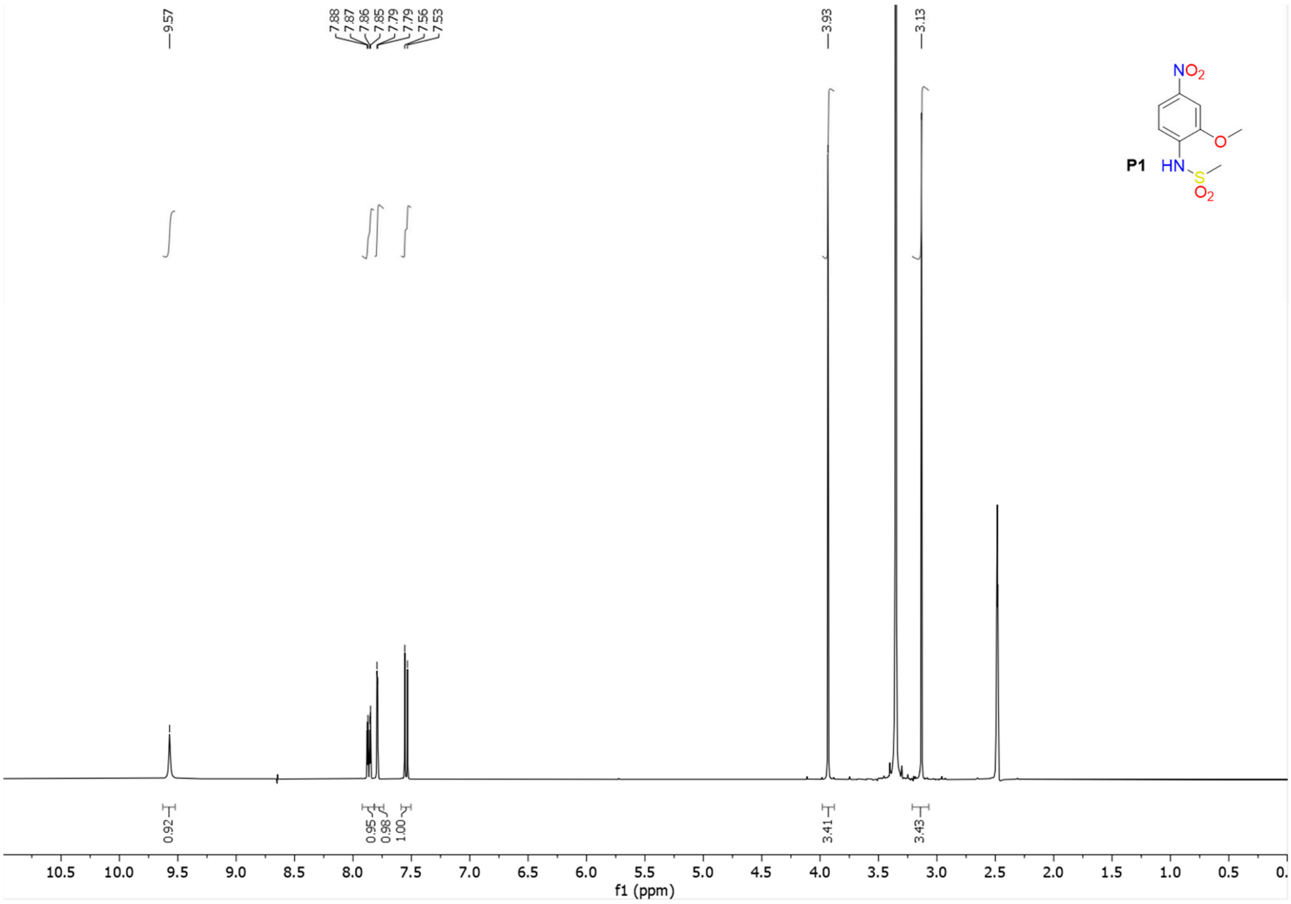

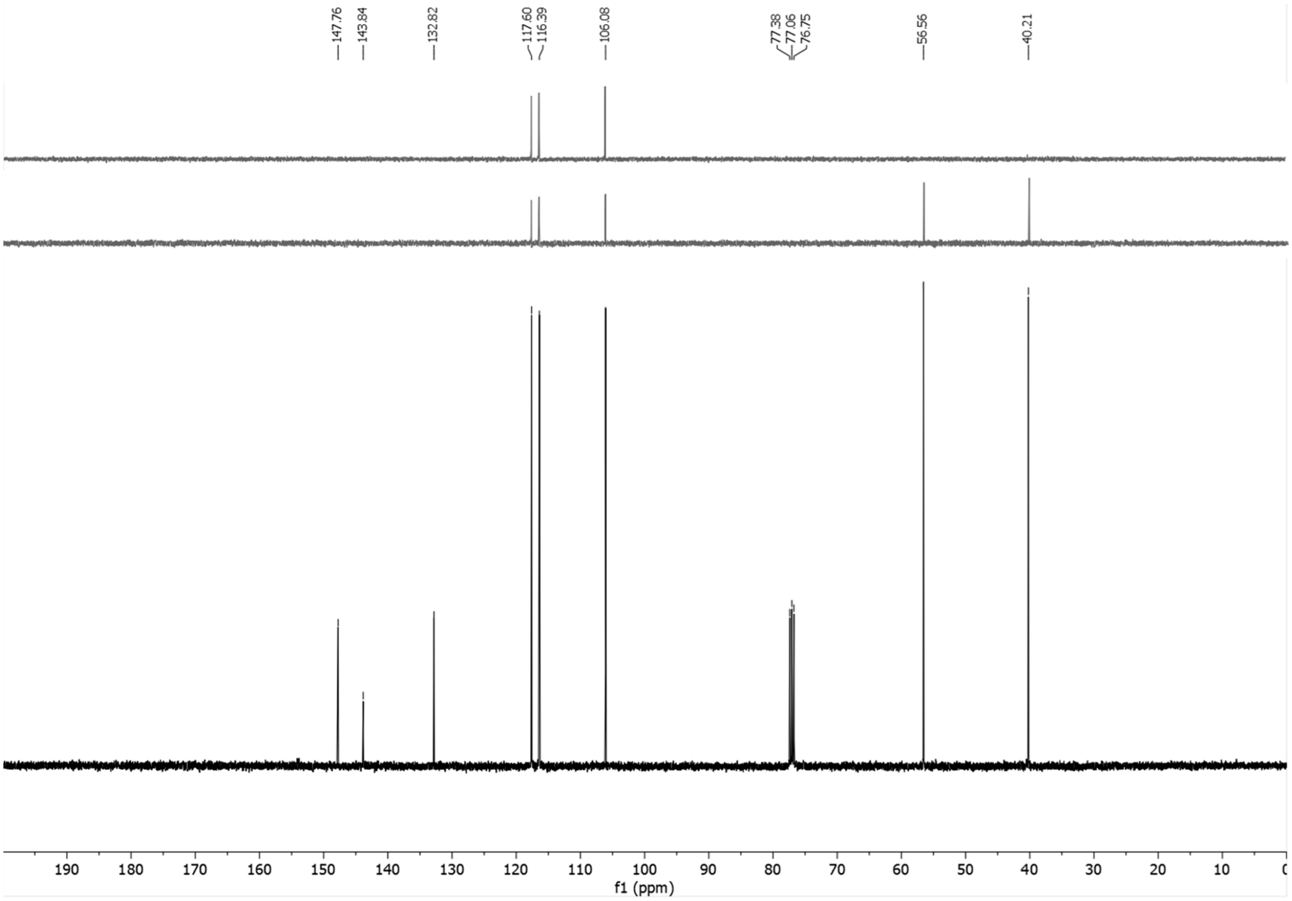

^{1}H and ^{13}C NMR spectra of *N*-(2-methoxy-4-nitrophenyl)methanesulfonamide (**P1**)

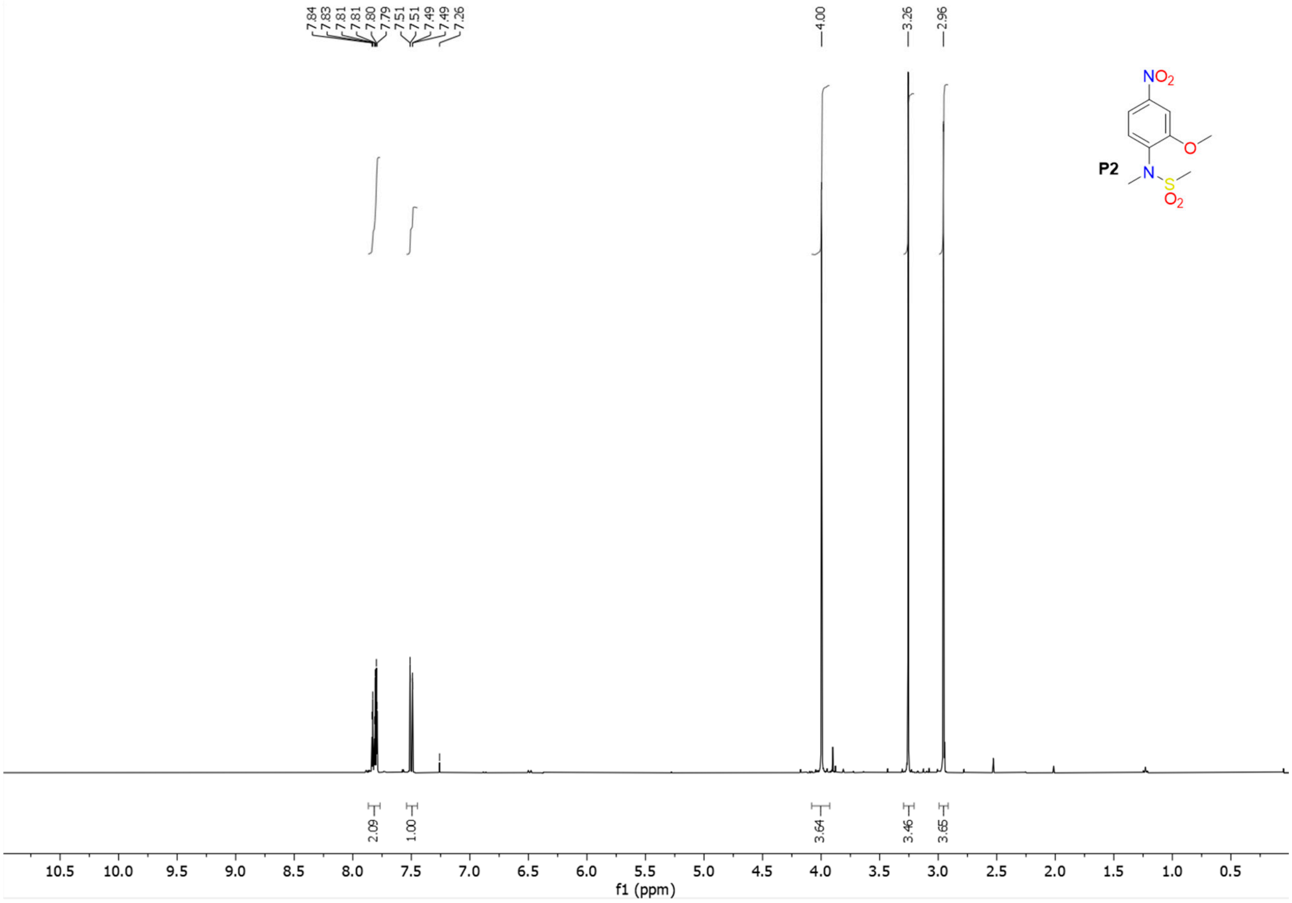

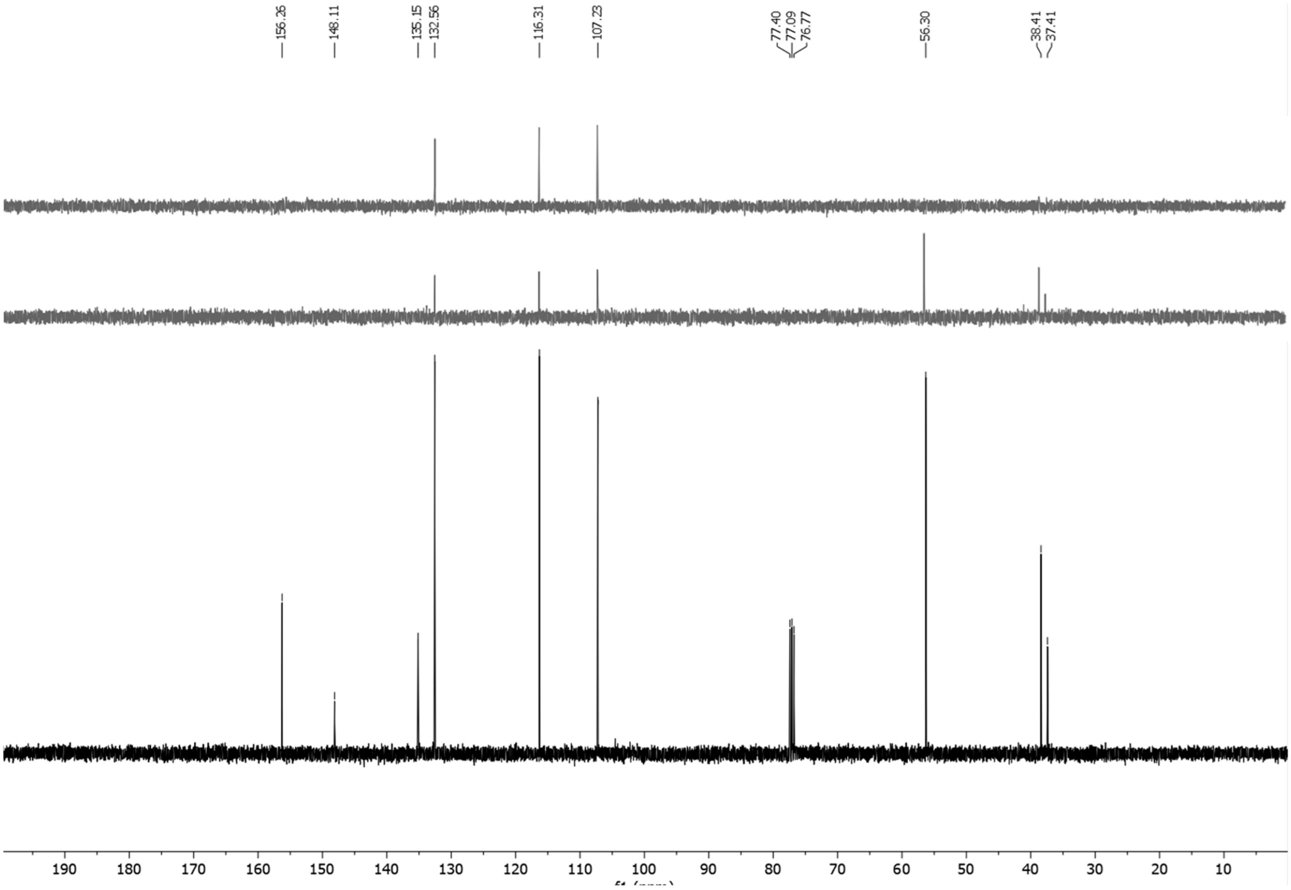

^{1}H and ^{13}C NMR spectra of ***N*-(2-methoxy-4-nitrophenyl)-*N*-methylmethanesulfonamide (P2)**

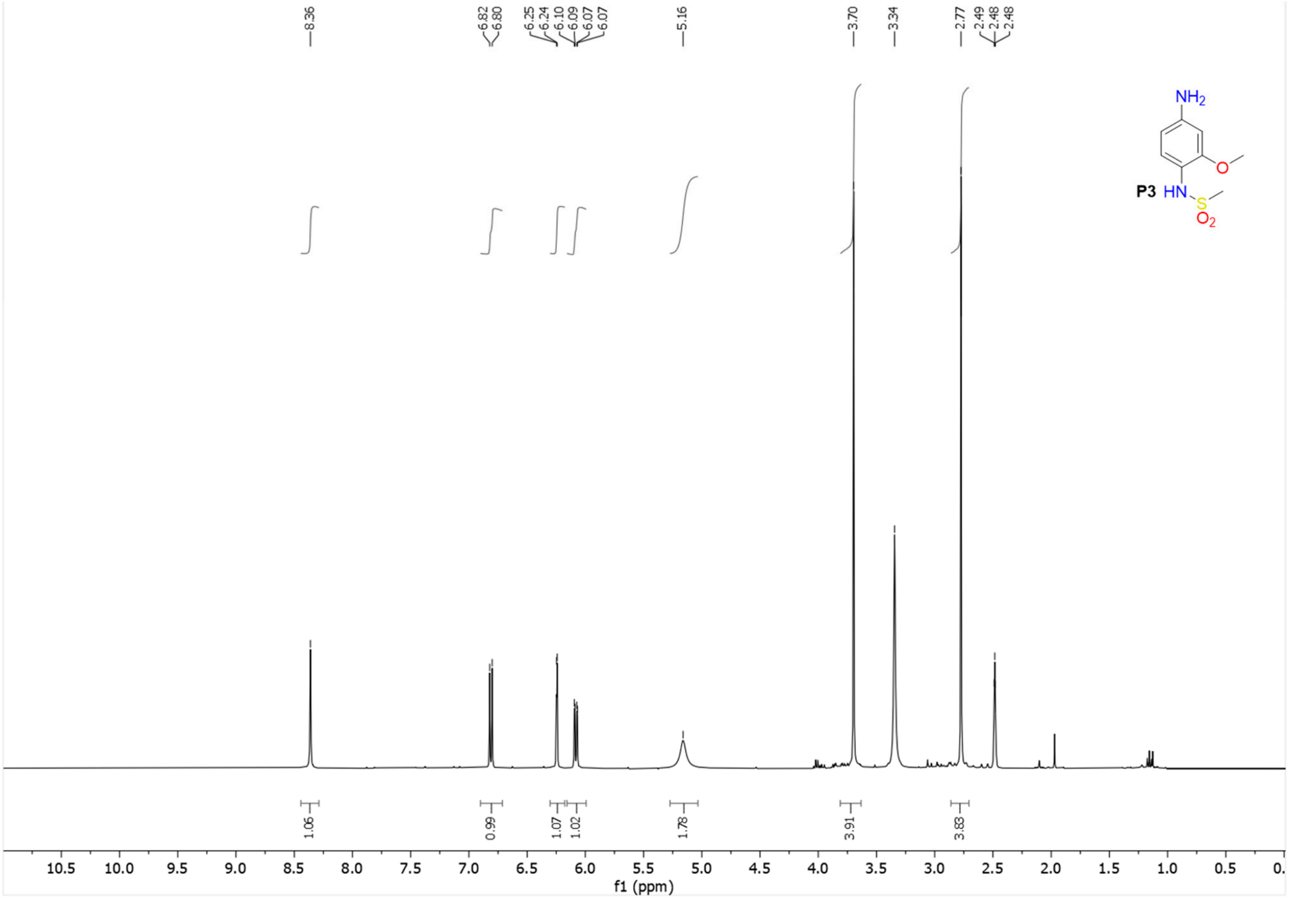

8.36
6.82
6.80
6.25
6.24
6.10
6.09
6.07
6.07
5.16
3.70
3.34
2.77
2.49
2.48
2.48
NH2
O
P3
HN
S
O2
1.06
0.99
1.07
1.02
1.78
3.91
3.83
f1 (ppm)

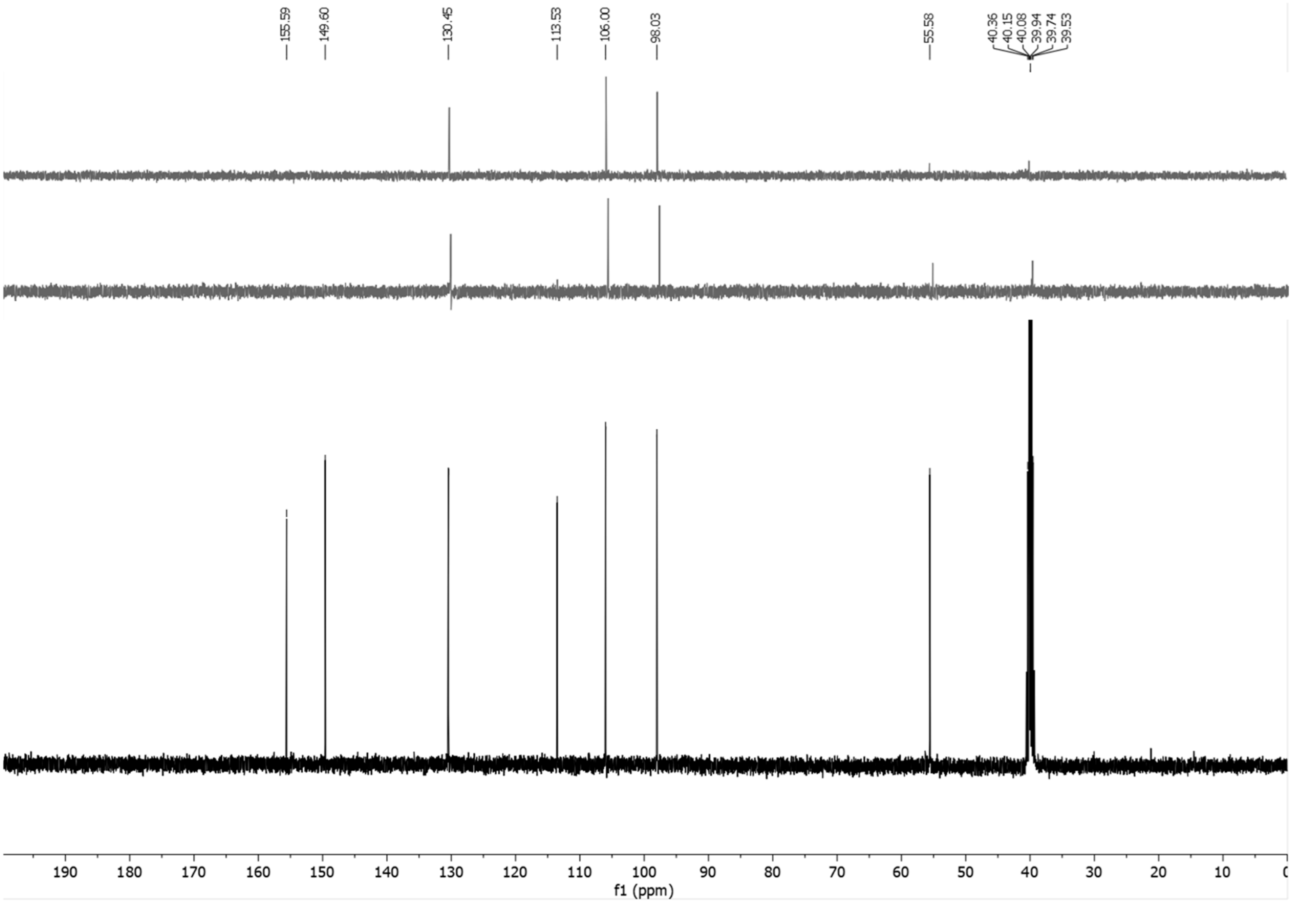

155.59
149.60
130.45
113.53
106.00
98.03
55.58
40.36
40.15
40.08
39.94
39.74
39.53
f1 (ppm)

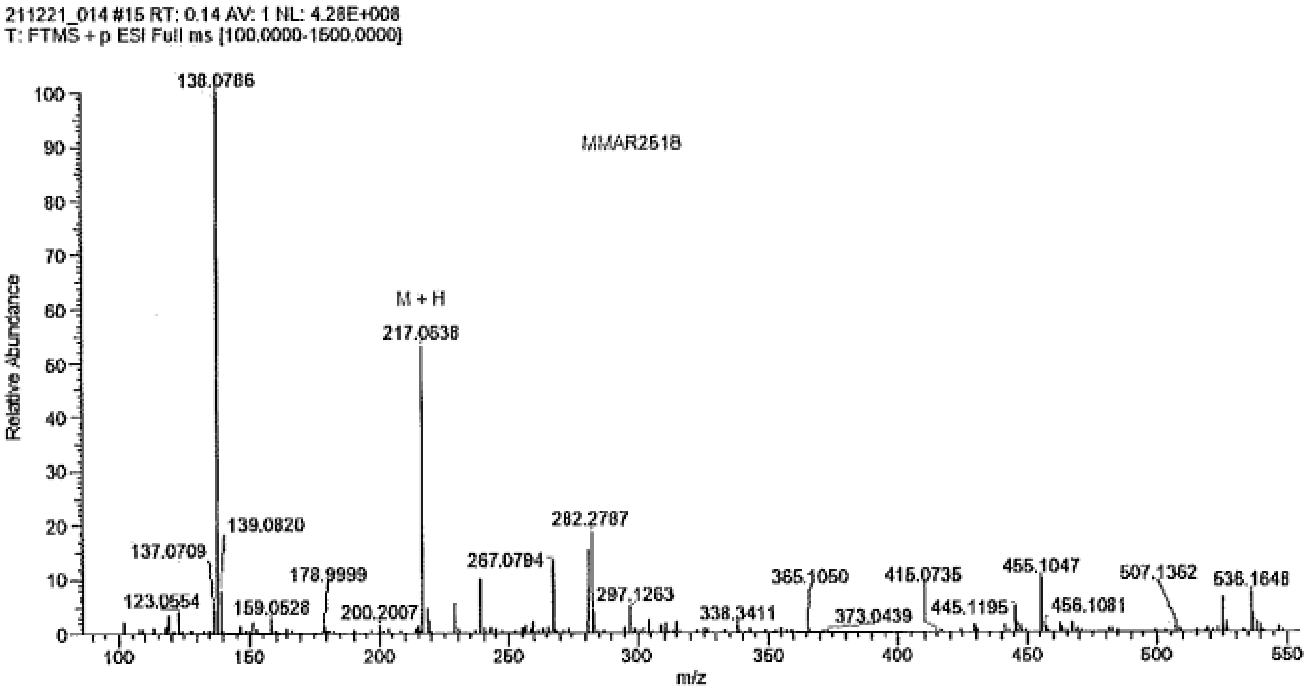

^{1}H, ^{13}C NMR and HRMS spectra of ***N*-(4-amino-2-methoxyphenyl)methanesulfonamide (P3)**

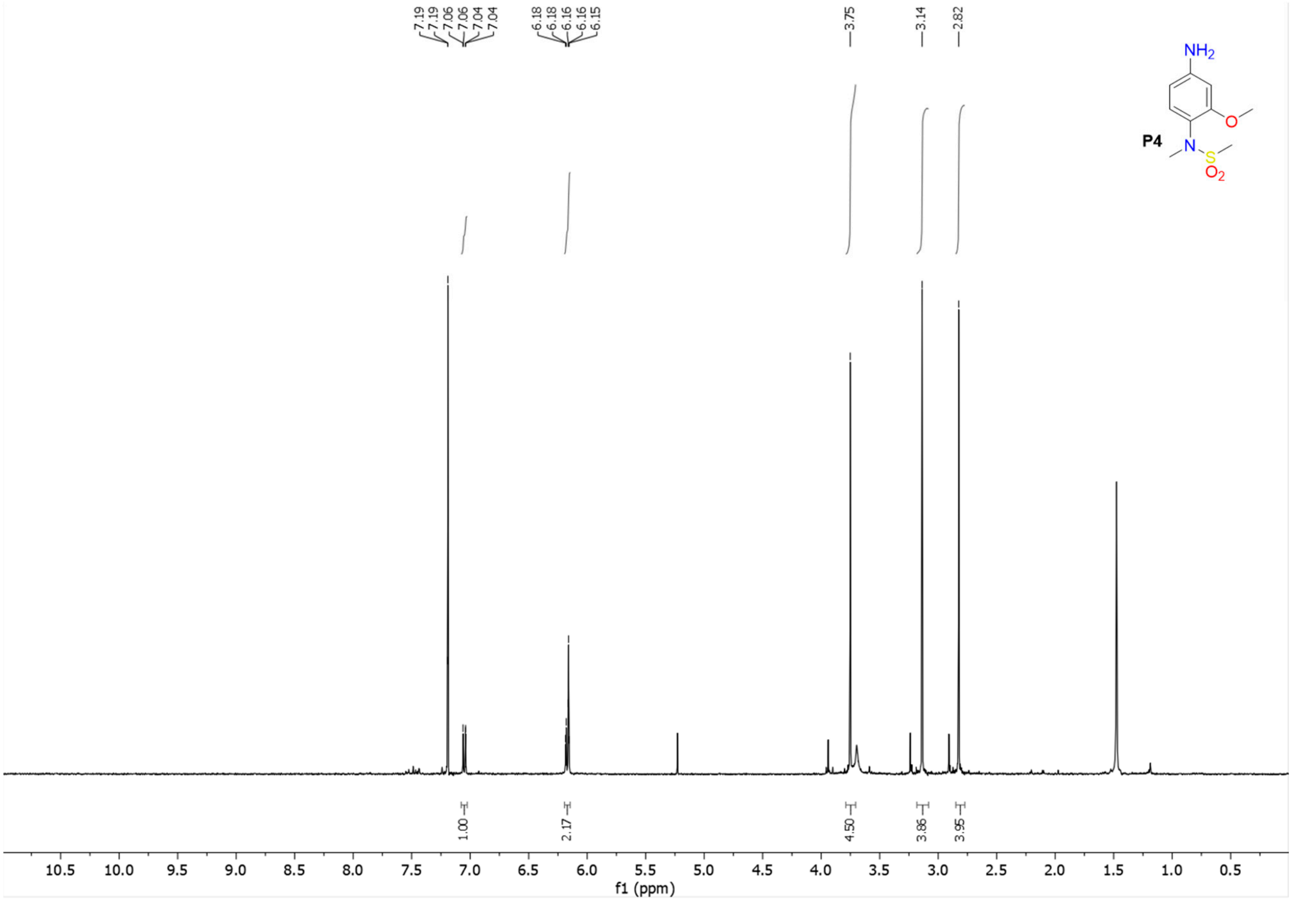

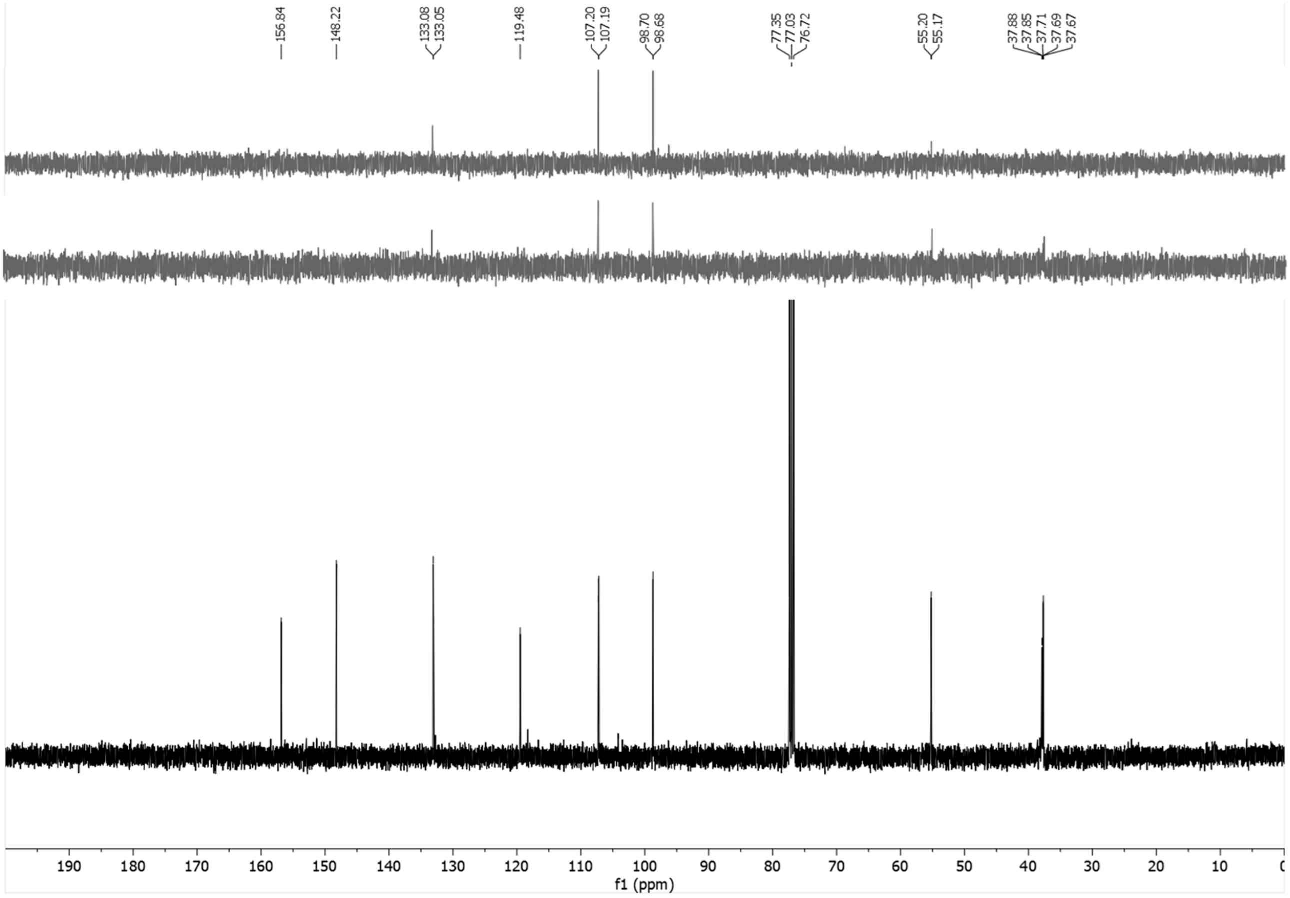

^{1}H and ^{13}C NMR spectra of ***N*-(4-amino-2-methoxyphenyl)-*N*-methylmethanesulfonamide (P4)**
